# Broad-spectrum fungal resistance in sorghum is conferred through the complex regulation of an immune receptor gene embedded in a natural antisense transcript

**DOI:** 10.1093/plcell/koab305

**Published:** 2022-01-09

**Authors:** Sanghun Lee, Fuyou Fu, Chao-Jan Liao, Demeke B Mewa, Adedayo Adeyanju, Gebisa Ejeta, Damon Lisch, Tesfaye Mengiste

**Affiliations:** Department of Botany and Plant Pathology, Purdue University, West Lafayette, Indiana 47907, USA; Department of Botany and Plant Pathology, Purdue University, West Lafayette, Indiana 47907, USA; Department of Botany and Plant Pathology, Purdue University, West Lafayette, Indiana 47907, USA; Department of Botany and Plant Pathology, Purdue University, West Lafayette, Indiana 47907, USA; Department of Agronomy, Purdue University, West Lafayette, Indiana 47907, USA; Department of Agronomy, Purdue University, West Lafayette, Indiana 47907, USA; Department of Botany and Plant Pathology, Purdue University, West Lafayette, Indiana 47907, USA; Department of Botany and Plant Pathology, Purdue University, West Lafayette, Indiana 47907, USA

## Abstract

Sorghum (*Sorghum bicolor*), the fifth most widely grown cereal crop globally, provides food security for millions of people. Anthracnose caused by the fungus *Colletotrichum sublineola* is a major disease of sorghum worldwide. We discovered a major fungal resistance locus in sorghum composed of the nucleotide-binding leucine-rich repeat receptor gene *ANTHRACNOSE RESISTANCE GENE1* (*ARG1*) that is completely nested in an intron of a cis-natural antisense transcript (NAT) gene designated *CARRIER OF ARG1* (*CARG*). Susceptible genotypes express *CARG* and two alternatively spliced *ARG1* transcripts encoding truncated proteins lacking the leucine-rich repeat domains. In resistant genotypes, elevated expression of an intact allele of *ARG1*, attributed to the loss of *CARG* transcription and the presence of miniature inverted-repeat transposable element sequences, resulted in broad-spectrum resistance to fungal pathogens with distinct virulence strategies. Increased *ARG1* expression in resistant genotypes is also associated with higher histone H3K4 and H3K36 methylation. In susceptible genotypes, lower *ARG1* expression is associated with reduced H3K4 and H3K36 methylation and increased expression of NATs of *CARG*. The repressive chromatin state associated with H3K9me2 is low in *CARG*-expressing genotypes within the *CARG* exon and higher in genotypes with low *CARG* expression. Thus, *ARG1* is regulated by multiple mechanisms and confers broad-spectrum, strong resistance to fungal pathogens.

## Teaser

Broad-spectrum fungal resistance in sorghum is conferred by an immune receptor locus regulated by miniature inverted-repeat transposable elements, antisense transcripts, and histone methylation.

##  

**Figure:**
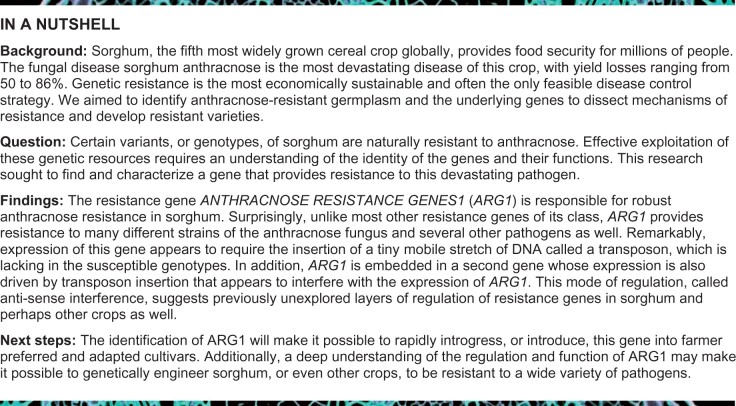


## Introduction

Plant pathogens account for 15%–30% of crop losses globally (Chakraborty and Newton, 2011; Savary et al., 2019). Genetic resistance has been successful in limiting losses to diseases in some crops. This approach has largely relied on leveraging the natural variation in the plant innate immune system, which is effective in restricting infection or inhibiting the progress of disease symptoms after infection. According to the current paradigm, the two primary branches of the plant immune system are pathogen-associated molecular pattern (PAMP)-triggered immunity (PTI) and effector-triggered immunity (ETI; Jones and Dangl, 2006). PTI is a form of disease resistance initiated upon the recognition of PAMPs by plasma membrane-anchored pattern recognition receptors (PRRs) located on the surface of plant cell membranes. Perception of PAMPs by PRRs activates a battery of immune responses, including the production of antimicrobial defense compounds and pathogenesis-related proteins and the accumulation of reactive oxygen species (Nicaise et al., 2009). This form of resistance is usually quantitative and often independent of the strain of the pathogen (Boller and Felix, 2009).

Plant pathogens often suppress PTI by deploying specific virulence effectors that interfere with PTI. In response, plants have evolved ETI, which is based on the recognition of effectors by structurally related but polymorphic intracellular immune receptors known as nucleotide-binding site leucine-rich repeat (NLR) proteins. The core of NLRs is the nucleotide-binding (NB) and leucine-rich repeat (LRR) domains with N-terminal coiled-coil (CC) or Toll/interleukin-1 receptor domains (Belkhadir et al., 2004). ETI activates stronger plant immune responses that confer resistance to strains of the pathogen that express particular effectors. The strong selective pressure on pathogens imposed by ETI results in frequent defeat of resistance by pathogens that acquire or lose new virulence effectors and thus escape recognition by NLRs (Jones and Dangl, 2006). Recent observations suggest that PTI and ETI represent a continuum of overlapping responses rather than being categorically distinct (Thomma et al., 2011).

Natural antisense transcripts (NATs) are noncoding RNAs that regulate gene expression in animal and plant cells through several mechanisms (Pelechano and Steinmetz, 2013). Transcription interference between the expression of protein-coding sense transcripts and the corresponding NAT (Silverman et al., 1992; Shearwin et al., 2005), chromatin modification (Csorba et al., 2014), RNA interference, and DNA methylation have all been implicated in regulating the expression of the sense transcripts (Faghihi and Wahlestedt, 2009; Magistri et al., 2012; Rinn and Chang, 2012). In plants, NATs regulate phosphate homeostasis in rice (*Oryza sativa*; Jabnoune et al., 2013), cell wall biosynthesis in barley (Held et al., 2008), cytokinin regulation in petunia (Zubko and Meyer, 2007), salt tolerance and fertilization in Arabidopsis (Borsani et al., 2005), fiber development in cotton (Wan et al., 2016), and drought tolerance in maize (Xu et al., 2017).

Sorghum (*Sorghum bicolor*) is an important food crop that also serves as a source of animal feed, biofuel, and other industrial products. Sorghum anthracnose, caused by the fungal pathogen *Colletotrichum sublineola* (*Cs*), is the most devastating foliar disease of this crop (Ali and Warren, 1992). Although anthracnose-resistant germplasm is available, the specific resistance regulators and their mechanisms of function have been unclear. In the current study, by screening a collection of sorghum natural variants, we found that the sorghum genotype SC283 displays a high level of broad-spectrum resistance to several different *Cs* strains, whereas the genotype TAM428 is susceptible to many different strains of the fungus. Recombinant inbred lines (RILs) generated by crossing SC283 with TAM428 displayed clear-cut resistance or susceptible disease responses similar to the parental lines. Whole-genome resequencing of DNA from resistant and susceptible RILs defined a major anthracnose resistance locus in SC283 that also confers resistance to other fungal pathogens. The resistance locus is composed of *ANTHRACNOSE RESISTANCE GENE 1* (*ARG1*) encoding a canonical NLR that is nested in an intron of a unique NAT designated *CARRIER OF ARG1* (*CARG*). DNA- and RNA-seq analysis revealed that in resistant RILs, the loss of *CARG* transcripts and a miniature inverted-repeat transposable element (MITE) insertion in the *ARG1* promoter region were associated with significantly enhanced expression of the full-length nested *ARG1* gene. In contrast, susceptible RILs produced two *ARG1* transcripts encoding truncated NLR proteins concomitant with an increase in NAT expression. The identity of the resistance gene and the relationship between the loss of *CARG* and enhancement of *ARG1* expression were validated in distinct sorghum natural variants that carry independent resistant and susceptible allele of the *CARG* and *ARG1* genes. In addition, histone H3K4 and H3K36 methylation at the region of overlap between *CARG* and *ARG1* and in the *ARG1* exon is enriched in resistant alleles but reduced in susceptible alleles. The repressive chromatin state associated with H3K9me2 is low within the *CARG* exon in *CARG*-expressing genotypes and higher in genotypes with low *CARG* expression. In summary, we discovered an immune receptor gene residing in an intron of a noncoding RNA gene that is regulated by MITE elements and confers complete and broad-spectrum fungal resistance.

## Results

### The sorghum line SC283 displays broad-spectrum resistance to sorghum anthracnose caused by *Cs*

**Figure 1:**
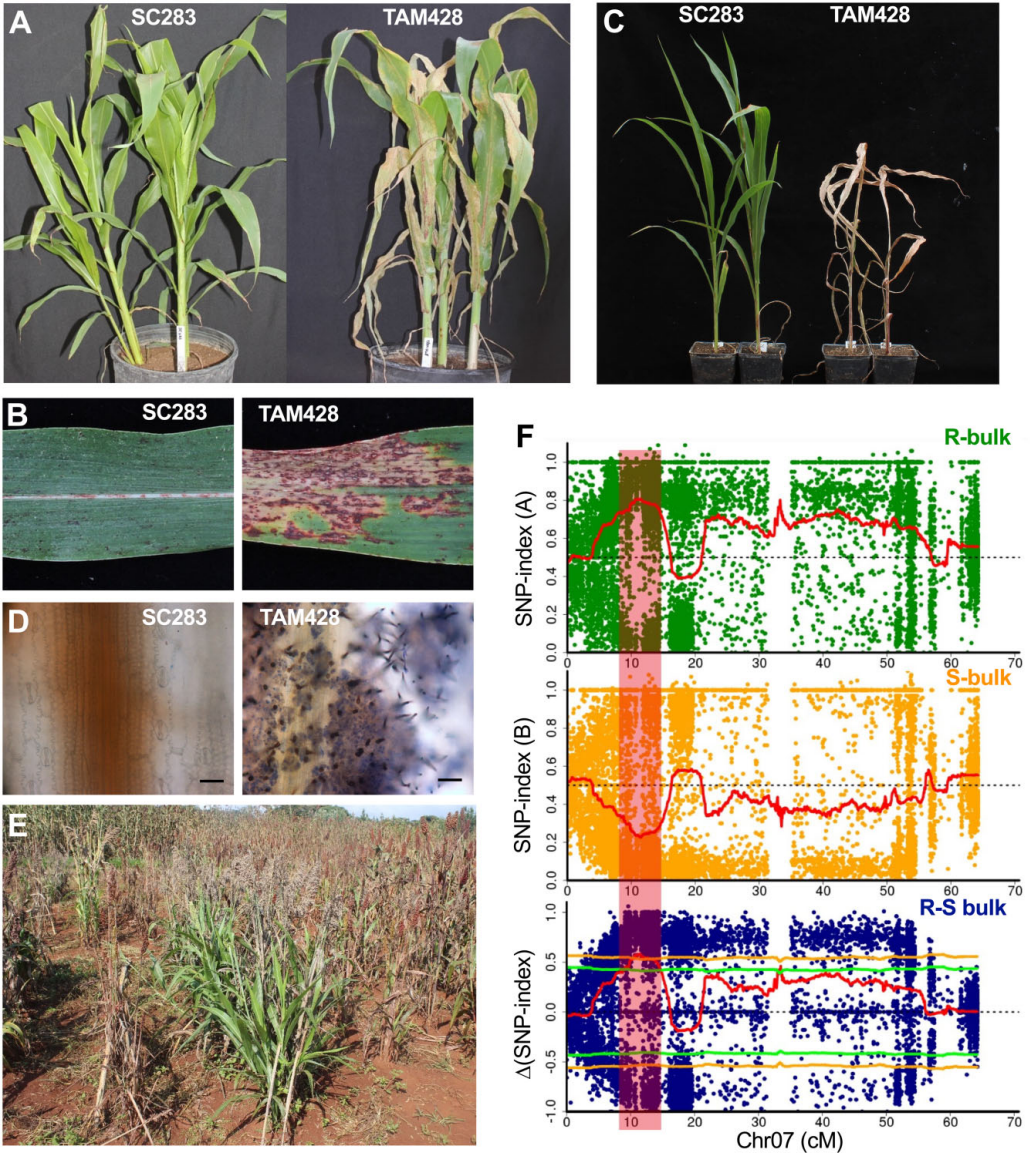
Disease responses of sorghum lines SC283 and TAM428 to *Cs* and identification of the resistance locus. A, C, Disease response phenotypes at 7-day post inoculation (dpi) (A), and 14 dpi (C). B, Disease symptoms on infected leaves at 10 dpi, (D) Trypan blue staining of *Cs* inoculated leaves showing fungal structures on TAM428 but a lack of fungal growth in SC283. Inoculated leaf tissues were stained with trypan blue, and samples were examined under a microscope to visualize fungal material. Scale bars represent 20 μm. E, Resistance of SC283 to foliar pathogens in the field under natural infestation. The experiments were repeated at least three times with similar results. In all the disease response data shown in (A)–(D), the *Cs* strain Csgl2 was used. F, Identification of an anthracnose resistance QTL in SC283 though QTL-seq analysis of recombinant inbred lines. SNP-index plots of R-bulk (top) and S-bulk (middle), and Δ (SNP-index) plot (bottom) of chromosome 7 with statistical confidence intervals under the null hypothesis of no QTLs (green, *P* < 0.05; yellow, *P* < 0.01). The Δ (SNP-index) plot was obtained by subtracting the of S-bulk SNP-index from the R-bulk SNP-index for the RILs. Plants were scored as resistant or susceptible based on their disease symptom or resistance response phenotypes. The DNAs from resistant or susceptible plants were bulked to make separate resistant (R) and susceptible (S) DNA bulks. S-bulk, DNA from the susceptible plants, R-bulk, DNA from the resistant plants. SNP index and Δ (SNP-index) was determined as described.

We screened diverse sorghum natural variants collected from different regions of the world for resistance to the hemibiotrophic fungal pathogen *Cs* by inoculation with a high concentration of fungal spore suspension and incubation under conditions that favor disease in the greenhouse (Supplemental Data Set S1). The sorghum genotype SC283 was resistant to 11 different *Cs* isolates from the USA and Africa, suggesting broad-spectrum resistance (Figure 1; Supplemental Data Set S2). The inoculated SC283 leaves remained healthy and displayed resistance with hypersensitive response (HR) after inoculation with the *Cs* strain Csgl2 (Figure 1, A and B). In contrast, the widely known susceptible line TAM428 lacked any apparent resistance response and showed extensive disease lesions, massively chlorotic leaf areas, and complete tissue collapse (Figure 1, A and B). At 2 weeks after inoculation, SC283 remained healthy with no symptoms of infection, whereas TAM428 plants were killed by the fungus (Figure 1C). Microscopic analysis of inoculated tissue after trypan blue staining revealed restricted fungal growth in SC283 but extensive growth in TAM428 (Figure 1D). Interestingly, SC283 also manifested enhanced resistance to pathogens in a field experiment in Western Ethiopia, where anthracnose is the most prevalent disease. Figure 1E presents fungal resistance of SC283 via natural infestation at a specific location.

### Identification of fungal resistance locus through whole-genome resequencing

RILs generated by crossing SC283 and TAM428 were used to identify the resistance locus in SC283 using an approach that combined bulked segregant analysis (BSA), whole-genome sequencing, and genetic mapping. We tested the responses of 209 RILs after *Cs* inoculation in the greenhouse, which identified 109 resistant and 100 susceptible RILs; these results are consistent with the expected 1:1 segregation ratio (χ^2^ = 0.387, *P *>* *0.05; Supplemental Data Set S3). The responses to the fungus in these resistant and susceptible RILs were similar to those of the parental lines SC283 or TAM428. We selected 50 resistant and 50 susceptible individual plants based on at least six rounds of independent disease assays (Supplemental Data Set S3). A pair of DNA bulks was constructed by pooling DNA from the 50 resistant and 50 susceptible RILs that were then sequenced using Illumina HiSeq 2500. More than one billion paired-end reads were obtained, including 494 million resistant bulk (RB) reads and 513 million susceptible bulk (SB) reads (Supplemental Data Set S4). These paired-end short reads covered the sorghum genome at an average depth of 66× and 68× in the RB and SB pools, respectively. In parallel, reference sequences were built by sequencing eight sorghum cultivars, including the two parental lines of the RILs used in this study (Supplemental Data Set S4).

**Figure 2:**
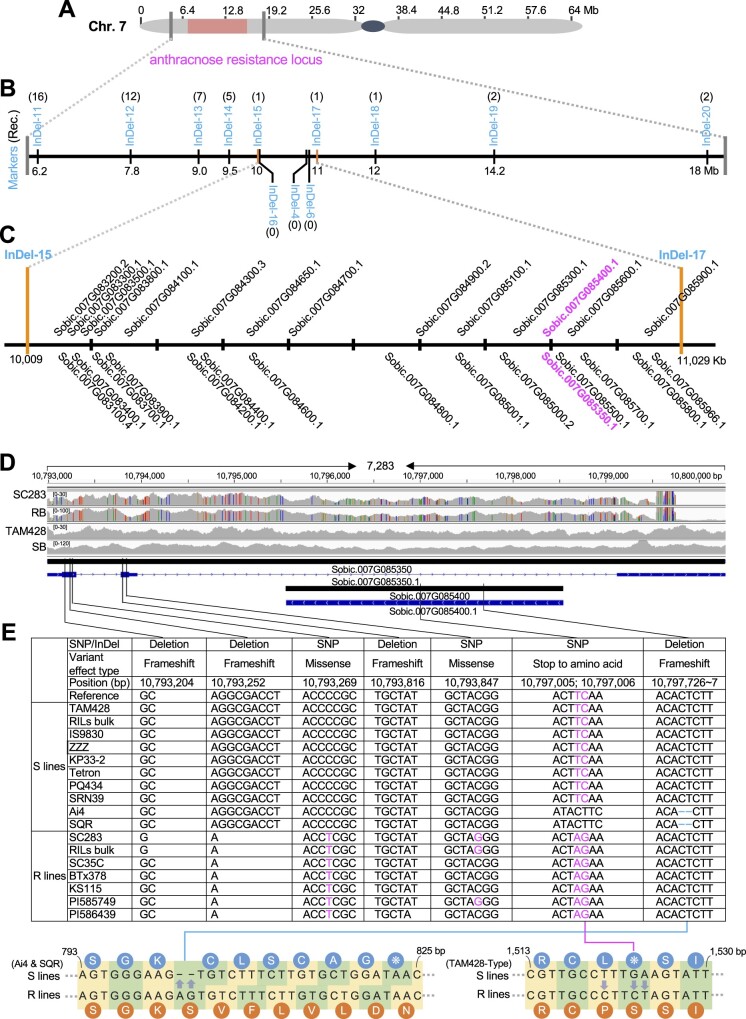
Fine mapping and sequence comparisons in mapped genomic region identifies *ANTHRACNOSE RESISTANCE GENE1*. A, Genomic region harboring the anthracnose resistance locus on Chromosome 7 (7.15–15.80 Mb). B, Fine mapping of the anthracnose resistance locus based on analyses of molecular markers shown on the physical map. The anthracnose resistance locus was fine mapped to a ∼780-kb genomic region flanked by InDel-16 and InDel-6 markers, which showed no recombination. InDel-16, InDel-4, and InDel-6 markers co-segregated with disease responses in the RILs. The numbers in brackets indicate the number of recombinants recovered. C, Gene models in the mapping region flanked by InDel-15 and InDel-17 markers (10,009–11,029 kb). The two candidate genes are shown in magenta. D, Comparisons of DNA sequence reads of the candidate genes Sobic.007G085350 and Sobic.007G085400 from RB and SB and parental lines SC283 and TAM428 using Integrative Genomics Viewer (IGV). The DNA sequences were compared with the reference genome BTx623, and SNPs were marked as color bars. The sequences from the SB and TAM428 show no sequence polymorphism relative to the reference genome in the mapping region. The sequence of RB is identical to that of SC283, and these sequences are different from those of both SB and TAM428. The genomic structure of the resistance locus showing Sobic.007G085350 (*CARG*) and Sobic.007G085400 (*ARG1*), which is nested in Sobic.007G085350. The coding and genomic regions with introns are based on predictions in the database (Phytozome V10; Sbicolor 313 V3.1), which was confirmed by our sequencing. Black bars indicate genomic region, blue bars indicate coding sequence, thin blue bars indicate UTRs, lines indicate introns, and arrows indicate the direction of transcription. E, Sequence variants in the Sobic.007G085350 (*CARG*) and Sobic.007G085400 (*ARG1*) genes between susceptible and resistant genotypes. The Sobic.007G085350 and Sobic.007G085400 genomic regions were amplified from different genotypes and sequenced to determine sequence polymorphisms and to identify additional alleles. The lower shows a sequence comparison between susceptible and resistant lines in Sobic.007G085400. Lower left, a 2-bp (AG) deletion at 10,797,728 was observed only in the susceptible Ai4 and SQR genotypes, which created a frameshift. Lower right, two SNPs at 10,797,005 and 10,797,006 in resistant lines create synonymous mutations that correct a stop codon in the Sobic.007G085400 gene that is in all the sensitive lines except for Ai4 and SQR. The codons and the corresponding amino acids are shown for resistant and susceptible lines. Dash indicates the deletion unique to Ai4 and SQR.

To determine the genomic region associated with resistance, we conducted BSA in the quantitative trait locus (QTL)-seq pipeline (Takagi et al., 2013), an approach combining BSA with whole genome resequencing that is often used to identify genes underlying both qualitative traits and QTLs. QTL-seq relies on an estimation of the single-nucleotide polymorphism (SNP) index in the RB and SB sequences in order to identify genomic region harboring the major QTL. More than 3 million SNPs were identified based on mapped reads for QTL analysis; these SNPs were unevenly distributed in the genome. We determined the SNP-index of each SNP using the QTL-seq pipeline (Supplemental Figure S1; Takagi et al., 2013) and calculated the Δ(SNP-index) by subtracting the SNP-index of SB from that of RB (Supplemental Figure S1). As expected, the Δ(SNP-index) was zero in most genomic regions, but a few regions exhibited positive or negative values, indicating differences from the sorghum BTx623 reference genome (Paterson et al., 2009; Supplemental Figure S1E). A Δ(SNP-index) higher than 0.44 was observed in the region from 7.15 to 15.80 Mb on chromosome 7 with *P *<* *0.05 under the null hypothesis. This contrasting pattern of the SNP-index for RB and SB defined a major *Cs* resistance locus within the 7.15–15.80 Mb genomic region on chromosome 7 (Figures 1, F and 2, A; Supplemental Figure S1E).

### Identification of candidate resistance gene(s) in the mapped anthracnose resistance locus

To further narrow the genomic region carrying the resistance locus, we developed 13 molecular markers that were polymorphic between the parental lines spanning the 6.2–18 Mb genomic interval that was defined to contain the anthracnose resistance locus. Phenotypic analysis identified recombination events between markers, which further narrowed the candidate genomic region (Figure 2, A and B; Supplemental Data Set S5). Based on these results, the anthracnose resistance locus was mapped to ∼780-kb genomic region flanked by InDel-16 and InDel-6 markers that showed a complete co-segregation with the disease phenotype (Figure 2B).

Next, to identify the specific *Cs* resistance gene, we annotated SNPs, insertions, and deletions in the ∼1 Mb mapping interval containing 29 genes (see “Materials and methods”) after filtering out low-quality sequences and SNPs with no polymorphisms in the parental lines (Cingolani et al., 2012). In all, sequence variants in 15 genes were closely analyzed, and most predicted genes were excluded based on a lack of significant polymorphisms (Supplemental Data Set S6). Importantly, sequence polymorphisms that have potential effects on gene function or the integrity of encoded proteins were mapped to two candidate genes: Sobic.007G085400 and Sobic.007G085350 (Figure 2C;  Supplemental Data Set S6). The susceptible TAM428 genotype contained a sequence polymorphism that introduced a premature stop codon in the Sobic.007G085400 coding sequence. However, in the resistant SC283 genotype, two SNPs replaced the stop codon with a serine codon in this gene, restoring the open reading frame (ORF; Supplemental Data Set S6).

Sobic.007G085400 encodes a canonical NLR with N-terminal CC domain, NB site, and C-terminal LRR domains (Supplemental Data Set S7), making it an excellent candidate, and is hence designated *ANTHRACNOSE RESISTANCE GENE1* (*ARG1*). This class of proteins function as intracellular receptors for effector proteins and are key determinants of ETI (Jones and Dangl, 2006). The sequence polymorphism in *ARG1* is consistent between the bulks and parental lines (Figure 2D). The susceptible lines and SB carry the stop codon and the resistant lines and the RB harbor the intact *ARG1* ORF (Figure 2E). Interestingly, Sobic.007G085400 (*ARG1*) is nested in the intron of the second candidate gene, Sobic.007G085350 (Figure 2D). In addition, an 8-bp sequence deletion (GGCGACCT) in the first exon of Sobic.007G085350 at position 10,793,252 on chromosome 7 was identified in the resistant parent SC283 that was not present in the susceptible parent TAM428 (Figure 2E;  Supplemental Data Set S6). The deletion in Sobic.007G085350 in SC283 was also present in RB but absent in the SB sequence (Figure 2E). Based on these genetic data, the polymorphism at Sobic.007G085350 is also considered to be the candidate causal sequence change that co-segregated with the resistance phenotype in SC283. The likely noncoding corresponding gene (Sobic.007G085350) is hereafter designated as *CARRIER OF ANTHRACNOSE RESISTANCE GENE* (*CARG*).

### The *ARG1* gene embedded in the *NAT* gene encodes an NLR receptor

**Figure 3:**
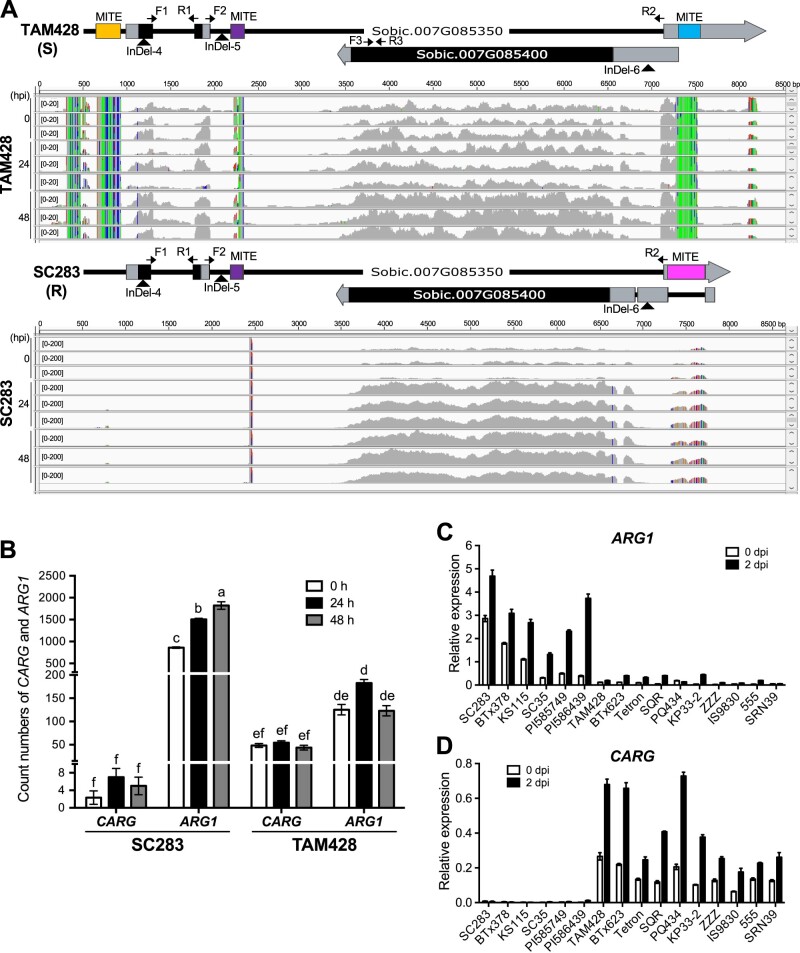
Genomic structure and RNA-seq analysis of the *CARG* and *ARG1* genes and upstream regulatory elements. A, Genomic structure of the *CARG–ARG1* locus. The lower panel shows RNA-seq analysis of the *CARG–ARG1* locus. RNA-seq was conducted at 0, 24, and 48 h after inoculation with *Cs*. Sequence reads were viewed by IGV and mapped to the TAM428 and SC283 genomic sequences of *CARG* and *ARG1*. The transcript count data are shown at 0–200 scale for SC283 and 0*–*20 scale for TAM428 due to the elevated levels of the transcripts for *ARG1* in SC283. The mapped transcripts, 5′- and 3′-RACE, qRT-PCR, and genomic sequence data were used to determine the exon, introns, and UTRs of *CARG* and *ARG1*. B, Expression of *CARG* and *ARG1* in SC283 and TAM428 based on RNA-seq transcript count data. Error bars indicate the standard deviation of three libraries (error bars ± sd [*n =* 3]). Different letters indicate significant difference based on the least significant difference (LSD, *P* < 0.05). C *ARG1* and D *CARG* expression in different sorghum lines. In (C) and (D), expression levels were analyzed by qRT-PCR in independent sorghum genotypes at 0 and 2 days after *Cs* inoculation. Data were normalized by the comparative cycle threshold method with *Actin* as the internal control and presented as relative expression. The data represent mean ± sd (*n* = 3 biological replicates per genotype, collected from one independent experiment). Similar results were obtained in two independent experiments.

Analyses of the genomic organization of the *CARG–ARG1* locus revealed that *CARG* has two exons, interrupted by two introns, the second of which is quite large (Figure 3A). The *ARG1* coding region is embedded in this large second intron. To delineate the boundaries of the *CARG–ARG1* genomic and transcript sequences, we conducted 5′- and 3′-rapid amplification of complementary DNA ends (RACE). The 5′-untranslated regions (UTRs) of the *CARG* gene are 148 bp in TAM428 and 139 bp in SC283, and the DNA sequences in these UTRs are identical except for a 9-bp size difference due to an Insertion/Deletion (InDel). SC283 carries a shorter *CARG* gene 3′-UTR (867 bp) than that of TAM428 (1,254 bp; Figure 3A;  Supplemental File S1). Both TAM428 and SC283 carry 740-bp *ARG1* 5′-UTRs with very high sequence similarity. However, in SC283, the 5′-UTR carries a 423-bp intron from positions −662 to −1,084 relative to the *ARG1* start codon and a second, 33-bp intron between positions −288 to −320, which are lacking in TAM428. TAM428 and SC283 both have 151-bp *ARG1* gene 3′-UTRs (Figure 3A;  Supplemental File S1). To confirm that the UTR sequences were not artifacts from cDNA synthesis, we performed reverse transcription polymerase chain reaction (RT-PCR) using one primer in the UTRs and one in the coding regions of *ARG1* or *CARG*. The PCR products were cloned and at least three individual clones were sequenced, revealing that the 5′-UTR of *ARG1* and the 3′-UTR of *CARG* partially overlap in both genotypes (Figure 3A;  Supplemental File S1).

Interestingly, the *CARG–ARG1* genomic region was found to harbor MITE-related sequences that showed significant differences between the resistant and susceptible genotypes (Figure 3A;  Supplemental File S1 and Supplemental Figure S2). The susceptible genotypes carry MITEs of 275 bp in the 5′-UTR, 151 bp in the second intron, and 248 bp in the 3′-UTR of *CARG*. The resistant lines carry the same 151-bp MITE in the second intron of *CARG*, as well as a different 420-bp MITE insertion in the 3′-UTR in this gene, which is also located in the first intron of *ARG1* in this allele. Sequence analysis of the 5′-UTR of this allele suggested that the MITE insertion introduced a splice junction that results in splicing of the MITE from the transcript. The locations of the MITEs relative to the *CARG–ARG1* genes are shown in Figure 3A. The 5′- and 3′-MITE sequences flanking the *CARG–ARG1* genes show very limited sequence identity with each other, and the MITEs in the 5′-UTR of the CARG in the two alleles were also inserted into different positions and thus likely represent two independent insertion events (Supplemental Figure S2).

We mapped RNA-seq data that we generated from healthy and pathogen-inoculated TAM428 and SC283 lines to genomic sequences of the corresponding lines to determine the transcript boundaries of *CARG* and *ARG1*. The *ARG1* transcripts were detected in both TAM428 and SC283, but the *CARG* transcript was observed only in TAM428 (Figure 3A). The RNA-seq further revealed that the basal expression level of *ARG1* was significantly higher in SC283, with further increased after *Cs* inoculation, while *CARG* expression was significantly lower both before and after infection (Figure 3A). Significantly different transcript read counts were observed for *CARG* and *ARG1* between TAM428 and SC283 (Figure 3B). In contrast, TAM428 exhibited higher *CARG* expression and much lower *ARG1* expression than SC283 (Figure 3B). The ratio of expression of *ARG1* and *CARG* was only two-fold different in the susceptible TAM428 line, compared to at least a 250-fold difference in SC283 based on RNA-seq data (Figure 3B), further supporting the hypothesis that the loss of *CARG* transcript in SC283 is correlated with the enhanced expression of *ARG1*.

The gene expression pattern observed from the RNA-seq data was confirmed using quantitative RT-PCR (qRT-PCR) with primers flanking introns in both the *CARG* and *ARG1* genes. The expression level of *ARG1* was significantly higher in six resistant genotypes and three resistant RILs carrying the *CARG* deletions than in lines where *CARG* is normally expressed (Figure 3C;  Supplemental Figure S3A). qRT-PCR using primers flanking the second *CARG* intron confirmed *CARG* expression in the susceptible genotypes and the susceptible RILs SSD50, SSD61, and SSD65, all of which exhibited significantly higher levels of *CARG* expression than in the resistant genotypes (Figure 3D;  Supplemental Figure S3B). A second primer pair flanking the first intron of *CARG* gave similar results (Supplemental Figure S3C). In every case, alleles that abrogate *CARG* gene expression were associated with both high levels of *ARG1* expression and resistance to anthracnose. Both *ARG1* and *CARG* were induced by fungal infection, with only *ARG1* showing a significantly larger induction in both genotypes.

### Validation of *ARG1* through characterization of independent alleles

To provide further genetic evidence for a link between the observed phenotypes and sequence variation in the candidate genes, we searched whole genome sequences of several sorghum genotypes to identify additional alleles of the *CARG* and *ARG1* genes. Sorghum lines carrying independent deletions and/or SNPs in the *CARG* and *ARG1* genes were identified from analysis of 46 deep sequenced cultivars and land race genotypes available in public databases and inhouse generated sequences that have been tested for anthracnose disease resistance (Supplemental Data Set S1). Among these, five resistant sorghum lines, SC35C, BTx378, KS115, PI585749, and PI586439, carried the same 8-bp deletion in the *CARG* gene and an intact *ARG1* gene, as was observed in SC283. The resistant lines also carried additional sequence alterations in the *CARG* gene that are distinct from those of SC283, providing additional genetic evidence for variation in these genetic backgrounds (Figure 2E;  Supplemental Figure S4). On the other hand, in all 40 susceptible lines examined, mutations that disrupt *ARG1* were linked to intact *CARG* genes. These susceptible lines each carried one of two distinct susceptible *ARG1* alleles, one with a premature stop codon identical to TAM428 at position 508 amino acid (aa) or a second, independent *ARG1* allele that introduced a stop codon at position 275 aa in Ai4 and SQR (Figure 2E;  Supplemental Figure S4 and Supplemental Data Set S1). These *ARG1* sequence variations between the susceptible and resistant genotypes are linked to polymorphisms observed in the 5′-upstream region and within the *CARG* gene sequences in all the genotypes studied. The tight linkage between two independently derived mutations that result in stop codons that are both associated with susceptibility provides strong evidence that a functional *ARG1* gene is required for resistance.

**Figure 4:**
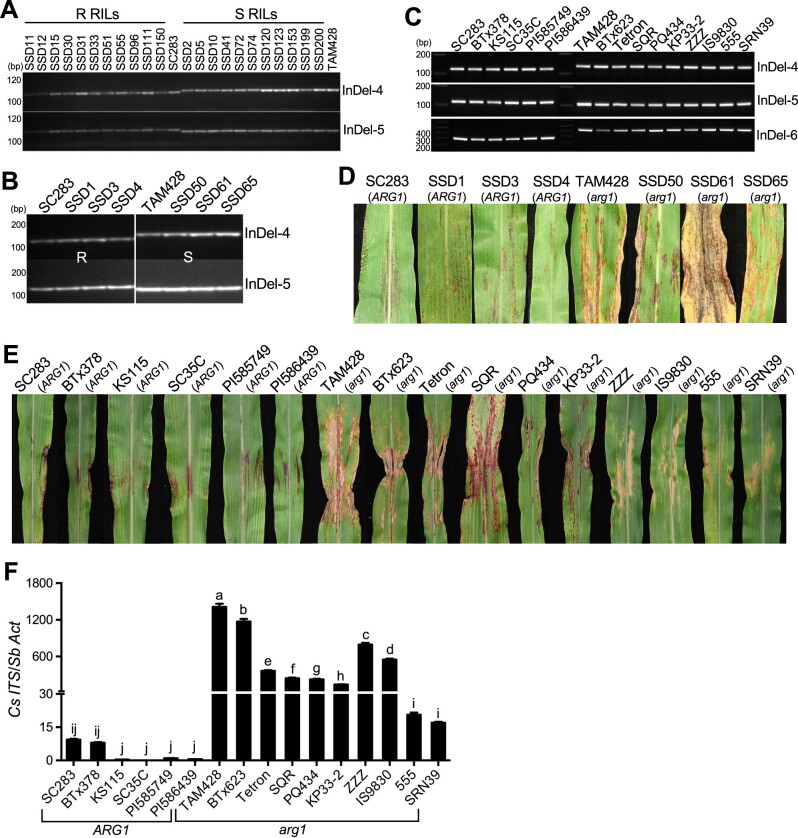
Co-segregation of molecular markers, and pathogen responses of recombinant inbred lines and distinct sorghum lines. A–C, Genotyping of the resistant and susceptible recombinant inbred lines (A and B) and different sorghum lines with distinct origins (C) using Indel molecular markers in Sobic.007G085350 and Sobic.007G085400. InDel4, InDel5, and InDel6 represent three deletion polymorphisms, which were used to design PCR markers in the Sobic.007G085350 and Sobic.007G085400 genes and are polymorphic between resistant lines and susceptible lines. R RILs, resistant recombinant inbred lines; S RILs, susceptible recombinant inbred lines; SSD, single seed decent. D, E, Disease response phenotypes of sorghum lines carrying different *CARG* and *ARG1* alleles after inoculation with *Cs* strain Csgl2. SSD1, SSD3, and SSD4 are resistant RILs, while SSD50, SSD61, and SSD65 are susceptible RILs. SC283, BTx378, KS115, SC35, PI585749, and PI586439 are resistant genotypes that carry the *CARG* deletion and intact *ARG1*. TAM428, BTx623, Tetron, SQR, PQ434, KP33-2, ZZZ, IS9830, 555, and SRN39 are susceptible genotypes that have intact *CARG* gene and mutated *arg1*. F, Quantification of fungal growth. Fungal growth in infected leaves was determined by qPCR amplification of the *Cs* Internal Transcribed Spacer rDNA (*Cs ITS*). Relative DNA levels were calculated using *SbActin* (*Sb Act*) as a reference gene. Data represent mean ±sd from three technical replicates. Different letters indicate statistically significant differences (*P* < 0.05, Student’s *t* test). Similar results were obtained in three different independent experiments.

The sequence variations among resistant and susceptible RILs and other independent sorghum genotypes were confirmed using molecular markers (Figure 4, A–C). The InDel-4 marker flanking the sequence deletion in the *CARG* (Sobic.007G085350) exon and InDel-5 within the *CARG* intron co-segregated with the resistance phenotype, which confirmed that the polymorphism in *CARG* is tightly linked to resistance on the same region of chromosome 7 (Figure 4, A–C). InDel-6, located in the *ARG1* promoter region, also co-segregated with the resistant and susceptible phenotypes. Resistance was invariably observed in RILs and five other genotypes with different origins that carried intact *ARG1* and the linked molecular markers (Figure 4, C–E).

The genotypes that carried the *ARG1* mutant allele and the associated polymorphisms showed typical disease symptoms, such as chlorotic and necrotic lesions and black spots caused by fungal acervuli (fungal reproductive structures) to a greater or lesser degree (Figure 4, D and E; Supplemental Figure S5). The susceptible genotypes TAM428, BTx623, Tetron, SQR, PQ434, KP33-2, ZZZ, IS9830, and Ai4 showed more severe and advanced disease symptoms, while 555 and SRN39 showed mild disease symptoms. These results are consistent with the presence of additional modifiers of disease resistance in these lines (Figure 4E;  Supplemental Figure S5).

To determine the relationship between disease symptoms and fungal growth, we quantified fungal growth in inoculated plants using quantitative PCR (qPCR) amplification of the internal transcribed spacer (ITS) region of the fungal ribosomal DNA (rDNA). Overall, the fungal growth correlated well with disease symptoms (Figure 4F). These analyses confirmed the sequence data, and the disease responses were consistent in all genotypes tested. Thus, among the genes that map to the QTL region, only the *CARG–ARG1* gene pair showed consistent sequence polymorphism between the two parental lines and between resistant and susceptible RILs, and this genetic association was confirmed using independent sorghum genotypes.

### Genetic inheritance, function, and the expression of *CARG* and *ARG1*

To determine the genetic inheritance of disease resistance with respect to the *CARG–ARG1* locus, we examined the F1 and selfed progenies from the TAM428 x SC283 cross. All 10 F1 plants tested were resistant. Of the 409 F2 single plants examined, 114 individuals were susceptible and 295 were resistant, with the *CARG* sequence deletion co-segregating with resistance. The values obtained from the analysis of segregation in the F2 population do not differ significantly from a 3 resistant: 1 susceptible segregation ratio (χ^2^ = 1.18, *P *>* *0.05), pointing to the monogenic and dominant nature of the allele linked to *Cs* resistance. These results demonstrate that *CARG–arg1* is a recessive allele and that the *carg–ARG1* allele is dominant for disease resistance. Thus, resistance to *Cs* is inherited as a dominant trait that is correlated with an upregulated and intact *ARG1* allele that is also closely linked the loss of the *CARG* transcript.

**Figure 5:**
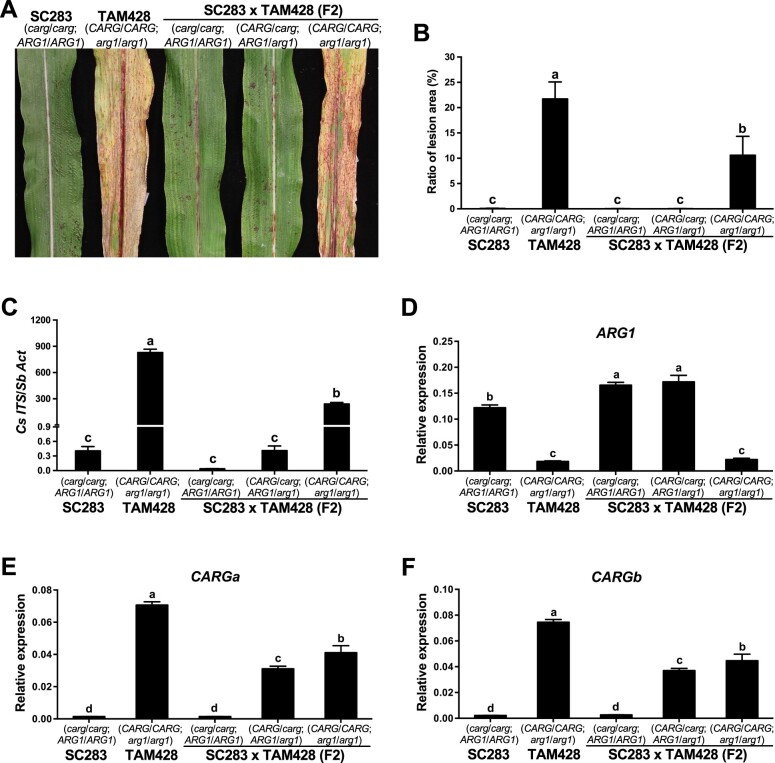
*ARG1* and *CARG* show contrasting expression patterns and inheritance. A Disease responses, (B) area of disease lesions, and (C) quantification of fungal growth in F2 plants with different *CARG* and *ARG1* alleles. In (B), the ratio of lesion area (%) is presented as mean ± sd obtained from five inoculated leaves. Different letters indicate statistically significant differences (*P* < 0.05, Student’s *t* test). In (C), fungal growth in infected leaves was determined by qPCR amplification of the *Cs* Internal Transcribed Spacer rDNA (*Cs ITS*). Relative DNA levels were calculated using *SbActin* (*Sb Act*) as a reference gene. Data represent mean ±se from three independent biological replicates. Data from each biological replicate consisted of nine technical replicates. Different letters indicate statistically significant differences (*P* < 0.05, Student’s *t* test). D, *ARG1* and (E and F) *CARG* expression in SC283, TAM428 and F2 plants. The *CARGa* primer set flanks the second *CARG* intron, and the *CARGb* primer pair flank the first intron of *CARG*. In (D–F), the expression levels were analyzed by qRT-PCR in SC283, TAM428, and F2 plants. Data were normalized by the comparative cycle threshold method with *Actin* as the internal control and presented as relative expression. The data represent at least four biological repeats with three technical replicates. Error bars show ± se (*n* ≥ 24). Different letters indicate significant differences among genotypes (*P* < 0.05, Student’s *t* test). Similar results were obtained in three independent experiments.

We genotyped individual F2 plants from the cross between SC283 and TAM428 and identified plants carrying different *CARG* and *ARG1* alleles. We evaluated plants carrying the homozygous *CARG* deletion (*carg/carg;ARG1/ARG1*), *CARG* homozygous wild-type (*CARG/CARG;arg1/arg1*), and heterozygous plants (*CARG/carg;ARG1/arg1*) for *Cs* resistance by assessing disease symptoms and fungal growth (Figure 5, A–C). Both TAM428 and F2 *CARG/CARG;arg1/arg1* plants displayed disease symptoms, including microscopic dark spots indicative of fungal acervuli and chlorotic leaves, which were quantified by measuring the area of the disease lesion relative to the total leaf area (Figure 5, A and B).

We also quantified fungal growth based on qPCR amplification of the ITS region of the fungal rDNA. The F2 *carg/carg;ARG1/ARG1* and *CARG/carg;ARG1/arg1* plants were equally resistant based on both fungal growth and quantification of disease symptoms and shared comparable levels of resistance with SC283 plants (*carg/carg;ARG1/ARG1*; Figure 5, B and C). The F2 *CARG/CARG;arg1/arg1* plants were significantly more susceptible than the F2 *carg/carg;ARG1/ARG1* and *CARG/carg;ARG1/arg1* plants, but clearly less susceptible than the TAM428 plants carrying the same *CARG/CARG;arg1/arg1* alleles (Figure 5, A–C). Similar differences were observed in disease symptoms and fungal growth when the various genotypes were drop inoculated in detached leaf assays (Supplemental Figure S6). These results suggest the presence of other factors in the SC283 background that modulate resistance. Overall, however, resistance was associated with the presence of ARG1 and susceptibility was attributed to the loss of the wild-type *ARG1* allele in all genotypes evaluated. The above data show that the *CARG–ARG1* locus determines resistance in SC283 and likely in other resistant sorghum genotypes as well.

We conducted gene expression analyses in the F2 plants. Of these plants, *carg/carg;ARG1/ARG1* and *CARG/carg;ARG1/arg1* plants showed comparable levels of resistance, having only the HR, which is consistent with their similar levels of *ARG1* expression (Figure 5, A and D). In all of these genotypes, *ARG1* gene expression levels were negatively correlated with *CARG* expression (Figure 5, D–F). In *CARG/carg;ARG1*/*arg1* plants, the level of *ARG1* expression was comparable to that in *carg/carg;ARG1/ARG1* plants, despite an intermediate level of *CARG* transcript. These results suggest that the expression of only one copy of *CARG* is not sufficient to affect overall *ARG1* transcript levels. However, the inverse correlation between *CARG* and *ARG1* expression suggests that enhanced resistance may partially result from a loss of cis-NAT *CARG* transcript, perhaps permitting a concomitant increase in expression of an intact *ARG1* allele in the resistant genotypes.

### The *ARG1* allele in susceptible genotypes expresses alternatively spliced transcripts encoding truncated NLRs

**Figure 6:**
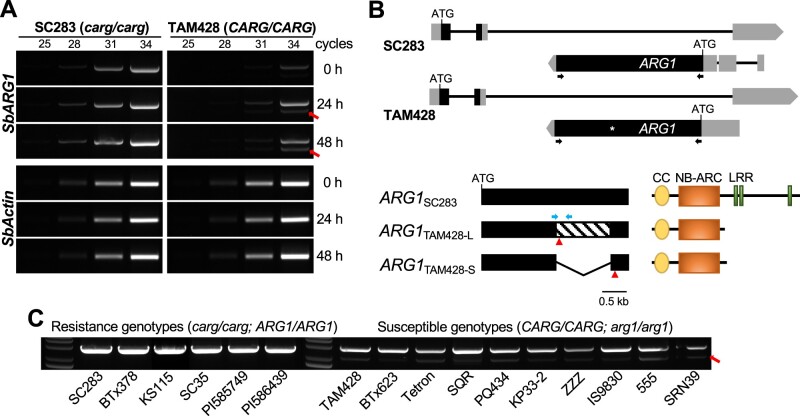
*ARG1* alleles in susceptible genotypes produce two differentially spliced transcripts. A, RT-PCR showing the expression of *ARG1*. The spliced *ARG1* variant is indicated by a red arrow. *Actin* shows equal amounts of cDNA input. Similar results were obtained in two independent experiments. B, A schematic drawing of *CARG* and *ARG1* genes (top). Exons and UTRs are shown as black and gray bars, respectively. ATG indicates translational start site and black arrows indicate the positions of primers used to amplify *ARG1* transcripts. The full-length and alternatively spliced *ARG1* transcripts are shown schematically. TAM428-U, full-length *ARG1* in TAM428; TAM428-D, splice variant of *ARG1* in TAM428. The red triangles indicate the positions of stop codon in the full-length and spliced *ARG1* transcripts and TAM428. The skipped exon in the spliced second variant transcript is represented by a diagonal pattern in the exon. The major domains in ARG1 proteins are shown in the bottom right panel. CC-NB-ARC, coiled coil, nucleotide binding site. C, RT-PCR showing *ARG1* transcripts in independent sorghum lines. The spliced *ARG1* variant is indicated by a red arrow. Similar results were obtained in two independent experiments.

To further confirm the *ARG1* expression patterns in response to *Cs* infection identified by RNA-seq, we performed RT-PCR analysis using primers permitting amplification of full-length *ARG1*. The transcript levels of *ARG1* in SC283 and TAM428 appeared to display good correlation with the RNA-seq data. A single pathogen-inducible *ARG1* transcript was observed in SC283, which harbored *CARG* with an 8-bp deletion and lacked the MITE in the 5′-region of this transcript. However, TAM428, which expresses the *CARG* NAT, produced two variant *ARG1* transcripts, both of which were pathogen inducible (Figure 6A). We sequenced all *ARG1* transcripts from SC283 and TAM428 to determine the nature of the splice variants of the *ARG1* transcript. Interestingly, the larger variant was comparable in size to the *ARG1* transcript in the resistant genotypes but carried a stop codon in the middle of the *ARG1* gene, as shown in Figure 2E. The second transcript was much smaller, skipped the LRR domain, and retained only the CC and NBs-ARC domains (Figure 6B;  Supplemental Figure S7). We analyzed alternative splicing of *ARG1* across all genotypes tested by our disease assay to determine whether this occurs in other susceptible genotypes. Two different *ARG1* transcripts were observed in the 10 susceptible lines tested, while all six resistant lines produced a single *ARG1* transcript (Figure 6C).

### The *CARG* 3′-UTR in the susceptible genotype produces small RNAs

The *CARG–ARG1* locus has an interesting genomic structure. The entire coding sequence of *ARG1* is embedded in an intron of *CARG*. The 5′-UTR of *ARG1* overlaps with the 3′-UTR of *CARG* (Figure 3A;  Supplemental Figure S8A), raising the possibility of an interaction between the two complementary transcripts. Due to the overlapping *CARG* and *ARG1* transcripts, there is a potential for formation of double-stranded RNA (dsRNA) and small RNAs (sRNA). We therefore conducted small RNA profiling of healthy and infected SC283 and TAM428 to identify sRNA sequences that map to the *CARG–ARG1* region that may regulate gene expression. A comparison of sRNAs in the two genotypes identified a cluster of sRNAs from a portion of the 3′-UTR of *CARG* of TAM428 but not of SC283. These sRNAs correspond to a MITE that is present in TAM428 but is missing from SC283, which has a different MITE that is spliced out of the SC283 *ARG1* 5′-UTR (Supplemental Figure S8A). Given that the MITE in TAM428 is present in very high copy numbers in the sorghum genome (Supplemental Figure S2), it is possible that these small RNAs are derived from transcripts that include this MITE elsewhere in the genome. Interestingly, however, the sRNAs were present in TAM428, but there was no sRNA accumulation in SC283, suggesting that at least some of these sRNAs are correlated with *CARG* expression (Supplemental Figure S8B). Interestingly, this MITE shows similarity to a hairpin variant of the MITE that expresses a putative pre-miRNA that is processed into sbi-miR6225 (miRBase, Version 21), which is similar to the small RNAs present at *CARG* (Supplemental Figure S9). The significance of these sRNAs needs to be determined in future studies.

### MITE sequences regulate *ARG1* gene expression

**Figure 7:**
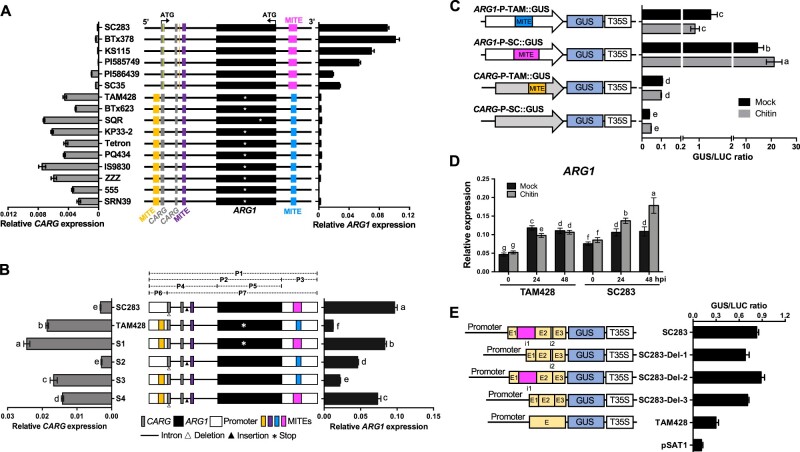
Regulation of *CARG* and *ARG1* genes by MITE and 5′-UTR intronic sequences. A, Genomic distribution of MITE insertions in the *CARG–ARG1* locus (middle). The *ARG1* gene is in the second intron of *CARG*. Black boxes denote the exon of *ARG1*, and gray boxes denote exons of *CARG*. The two genes are in the opposite orientation. The promoter regions (upstream from the ATG) are represented as a thick horizontal line; the yellow and orange vertical lines are indel; the gold, violet, blue, and magenta boxes indicate 275-, 151-, 248-, and 420-bp MITE insertions, respectively. The stop codon in the middle of *ARG1* in the susceptible genotypes is indicated by the white asterisk. *CARG* (left) and *ARG1* (right) transcript levels were quantified by qRT-PCR. Data were normalized by the comparative cycle threshold method with *Actin* as the internal control and presented as relative expression. The data in both panels represent the mean ± sd, *n* = 6. B, Regulation of the *CARG–ARG1* locus by the flanking MITE sequences. Schematics of the swapping constructs for the expression of the wild-type and chimeric *CARG–ARG1* loci in Arabidopsis are shown in the middle. The full (P1) and partial (P2*–*P7) fragments of *CARG–ARG1* loci amplified by PCR are indicated as dashed lines above the diagram of the wild-type and chimeric *CARG–ARG1* genomic region. All swapped constructs were expressed in Arabidopsis under the control of their native promoters. The expression levels of *CARG* (left) and *ARG1* (right) were quantified by qRT-PCR. Data were normalized by the comparative cycle threshold method with Arabidopsis *ACTIN* as the internal control and presented as relative expression. The data in both parts represent the mean ± sd, *n* = 6. Different letters indicate statistically significant differences (*P* < 0.05, Student’s *t* test). C, *ARG1* MITE from the SC283 allele regulates basal and chitin-induced *ARG1* expression. Schematics of the *ARG1* and *CARG* promoter GUS reporter construct on the left and their GUS activities with or without chitin treatment in Arabidopsis protoplasts. White and gray arrows indicate the promoter of *ARG1* and *CARG*, respectively. T35S: CaMV 35S terminator. Each construct was co-transfected with a CaMV 35S:luciferase construct into Arabidopsis protoplasts. Transfection efficiency was normalized relative to the expression of the Luciferase gene. Data are mean ± sd from triplicate experiments. Different letters show significant differences (*P* < 0.05, Student’s *t* test). The transfection assays were performed in triplicate and repeated three times. D, Chitin induces *ARG1* expression in the resistant genotype. Sorghum plants were treated with 2-nM chitin (β-1,4-linked *N*-acetylglucosamine) before RNA extraction. Data were normalized by the comparative cycle threshold method with *Actin* as the internal control and presented as relative expression. Data show mean ± sd from two independent tissues with three technical replicates (*P* < 0.05, Student’s *t* test). E, Regulation of the gene expression by introns in the *ARG1* 5′-UTR. Schematics of constructs derived from the *ARG1* 5′-UTR promoter driving GUS expression, and their GUS activities in Arabidopsis protoplasts. The gold boxes represent the 5′-UTRs; the magenta and white boxes represent the introns with i1 and i2; the promoters are shown by thick bars; T35S:CaMV 35S terminator. Each construct was co-transfected with a CaMV 35S:luciferase construct into Arabidopsis protoplasts. Transfection efficiency was normalized using the expression of the Luciferase gene. Data are mean ± sd from triplicate experiments. Different letters show significant differences (*P* < 0.05, Student’s *t* test). The transfection assays were performed at least in triplicate and repeated three times.

We studied the association between the MITE insertions and the expression of the *CARG* and *ARG1* genes via qRT-PCR (Figure 7A). A higher level of *CARG* expression was observed in the susceptible lines harboring a 275-bp MITE insertion immediately upstream of the *CARG* transcript than in the resistant lines lacking this MITE, suggesting that this MITE may be driving *CARG* expression. Similarly, in lines that carry the 420-bp MITE in the 5′-UTR intron of *ARG1*, higher expression of this gene was observed. In contrast, the 248-bp MITE insertion in the 3′-UTR of *CARG* found in the susceptible genotypes did not correlate with any significant induction of *ARG1* expression (Figure 7A). These results suggest that the 420-bp MITE in the 5′-UTR of *ARG1* positively regulates *ARG1* expression in the resistant genotypes, whereas the 248-bp MITE negatively regulates *ARG1* expression in the susceptible genotypes, perhaps due to induction of expression of the *CARG* NAT.

To further evaluate the roles of MITEs and the *CARG* NAT in regulating gene expression, we generated a series of constructs derived from the *CARG–ARG1* locus with swapped sequences between SC283 and TAM428 (Figure 7B). First, the *ARG1* promoter region in TAM428 was replaced with that from SC283 and vice versa (S1 and S2). Second, the *ARG1* coding region in TAM428 was replaced with the *ARG1* from SC283 (S3). Third, the *CARG* promoter region in SC283 was replaced with the *CARG* promoter from TAM428 (S4). The resulting constructs were individually transformed into Arabidopsis in parallel with constructs representing the native *CARG–ARG1* genes in SC283 and TAM428. We selected single copy transgenic plants for all six Arabidopsis lines and measured *CARG* and *ARG1* expression using qRT-PCR (Figure 7B).

The expression of *CARG* and *ARG1* in Arabidopsis lines carrying wild-type *CARG–ARG1* genes (SC283 and TAM428) recapitulated the patterns in the sorghum genotypes, indicating that the transgenes function properly in Arabidopsis (Figure 7B). As a rule, the expression of each gene reflected the promoter associated with it in each construct. Thus, constructs S1, S3, and S4, which all had the native *CARG* promoter from TAM428 driving *CARG* expression, had levels of expression of this gene similar to that of the native TAM428 construct. Similarly, S1 and S4, which had the native promoter of *ARG1* from SC283, showed similar levels of expression of this gene to that of the SC283 native construct (Figure 7B). Thus, each promoter appears to function largely independently. However, *ARG1* revealed contrasting expression tendencies to *CARG* in S2 and S3 (Figure 7B), suggesting that at the relatively low levels of expression of *ARG1* driven by the TAM428 promoter, *CARG* expression can in fact repress *ARG1* expression.

### The exogenous application of chitin and the 5′-UTR intron regulate *ARG1* expression

We tested the ability of the promoter sequences of *ARG1* from TAM428 and SC283 carrying distinct MITEs to regulate gene expression. The *CARG* and *ARG1* promoters were cloned into β-glucuronidase (GUS) reporter constructs, and each construct was transfected together with a CaMV 35S:luciferase (LUC) construct into Arabidopsis protoplasts. GUS activity was normalized to the fluorescence of luciferase, which was used to determine the relative transcriptional efficiency. The *ARG1* promoter from SC283 (*ARG1*-P-SC:GUS) significantly increased GUS activity, but the *ARG1* promoter from TAM428 (*ARG1*-P-TAM:GUS) yielded lower GUS activity (Figure 7C). A lower level of GUS activity was generally observed using the *CARG* promoters, but GUS activity was higher using the TAM428 construct compared to the SC283 construct. Interestingly, *ARG1*-P-SC:GUS activity increased in response to chitin (β-1,4 linked *N*-acetylglucosamine) treatment relative to the mock-treated samples, while *ARG1*-P-TAM:GUS activity showed no significant change in response to chitin treatment (Figure 7C). The *CARG* promoter GUS constructs showed no altered response to chitin treatment in all assays tested.

Chitin fragments are PAMPs generated by plant chitinase activity during fungal infection that are subsequently perceived by plant cells to activate immune responses (Gong et al., 2020). *Cs* significantly induced *ARG1* expression in SC283, which may partially be attributed to recognition of chitin fragments (Figure 3C). To confirm the responses to chitin observed in protoplasts, we treated detached TAM428 and SC283 leaves with 2-nM chitin and analyzed *ARG1* expression. *ARG1* expression increased at 24 h after mock or chitin treatment in both TAM428 and SC283 (Figure 7D). However, in both genotypes, the levels of *ARG1* expression did not differ after 24 h or 48 h of mock treatment, while the expression of *ARG1* was significantly induced by 24 h and 48 h of chitin treatment in SC283 (Figure 7D). Thus, *ARG1* is differentially regulated in response to chitin in the SC283 and TAM428 backgrounds, likely due to differences in upstream sequences.

The resistant allele of *ARG1* contains two introns in the 5′-UTR that are missing in *ARG1* from the susceptible allele. One of the two introns in SC283 *ARG1* is likely spliced due to the presence of its unique MITE (Supplemental Figure S2). To examine the roles of these introns in regulating gene expression, we generated constructs with and without these sequences (Figure 7E). Three constructs with various deletions of the introns in the 5′-UTR of *ARG1* from SC283 were fused to the GUS reporter gene in the pSAT1 vector. SC283-Del-1 contains the native *ARG1* promoter and the 5′-UTR but lacks the 423-bp intron that includes the MITE element. SC283-Del-2 carries the native promoter and 5′-UTR but lacks the 33-bp intron, and SC283-Del-3 contains the native promoter and the 5′-UTR but lacks all introns. We transfected Arabidopsis protoplasts with these constructs, with the CaMV 35S:LUC construct used as an internal control. Protoplasts harboring all of the constructs showed a significant increase in GUS activity compared to the negative control, i.e., the pSAT1 vector, which contains the Nopaline synthase (Nos) promoter without the GUS reporter gene. SC283-Del-1 and SC283-Del-3 exhibited lower GUS expression compared to SC283, whereas GUS expression in SC283-Del-2 was similar to that of SC283. These results indicate that the 423-bp intron including the MITE in the 5′-UTR functioned as transcriptional activator in the protoplast transient expression assays (Figure 7E).

### Permissive chromatin at the *ARG1* locus is correlated with fungal resistance

The higher expression of *ARG1* in resistant genotypes is linked to a loss of the *CARG* NAT. This raised the possibility that *ARG1* is regulated by *CARG* noncoding RNA, which could result in DNA methylation, histone methylation, or transcriptional interference due to the opposite orientation of the two transcripts. We first examined DNA methylation status in the coding regions of *ARG1* using bisulfite sequencing analyses. In general, there was no significant difference in DNA methylation between SC283 and TAM428 within the *ARG1* exon (Supplemental Figure S10). However, we were not able to analyze DNA methylation in the promoter regions of *ARG1* and *CARG* due to polymorphisms in the promoter sequences of these genes.

**Figure 8:**
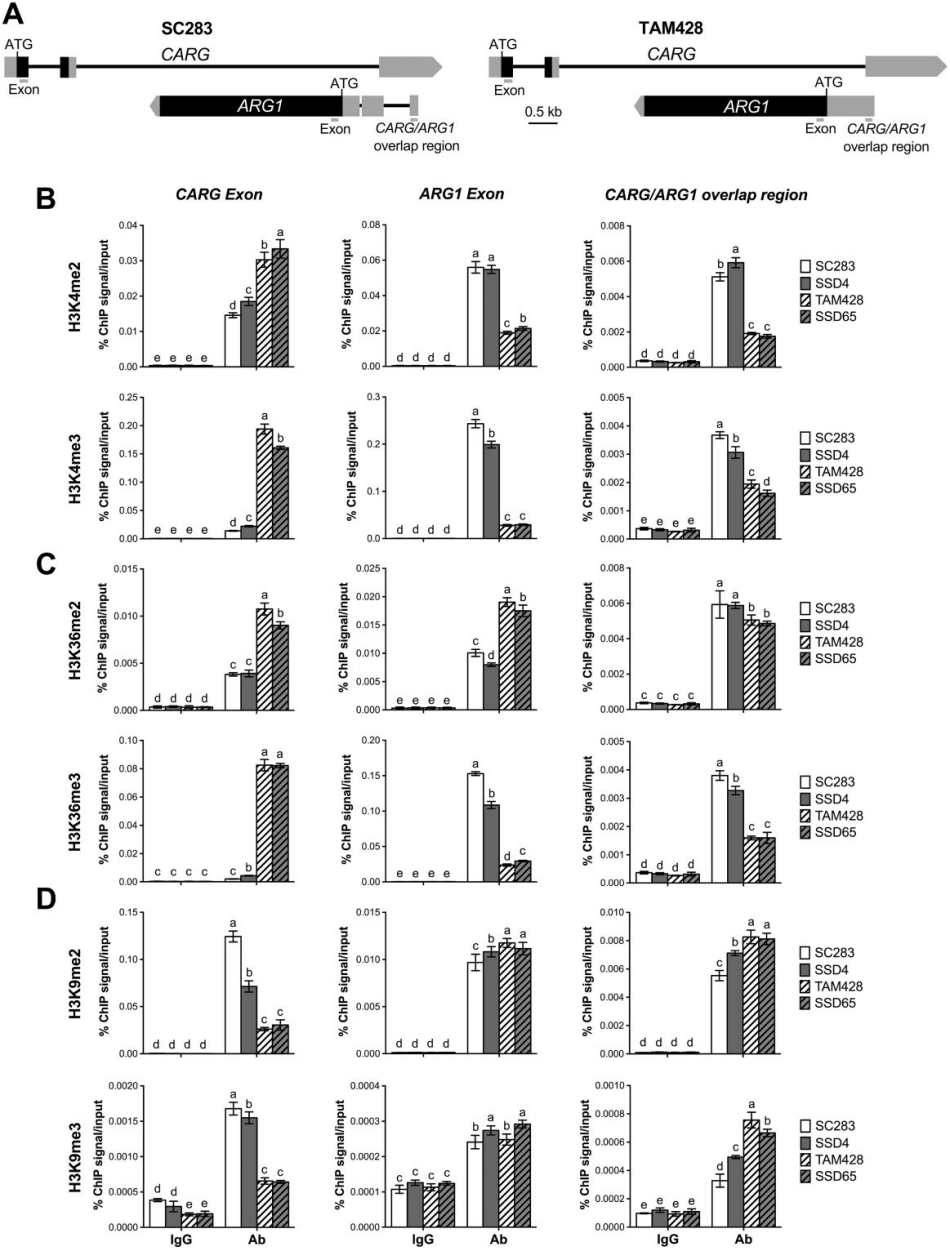
Changes in active and repressive Histone H3 lysine methylation at *CARG–ARG1* chromatin. A, Schematics showing the *CARG* and *ARG1* genomic region. The location of the primers at the coding (Exon) and *CARG/ARG1* overlap region used to analyze the level of H3K4, H3K36, and H3K9 methylation by ChIP assays are indicated by bars. The gray and black boxes indicate UTRs and exons, respectively. B–D, ChIP-qPCR analysis at *CARG–ARG1* locus with antibodies against (B) H3K4me2/3, (C) H3K36me2/3, and (D) H3K9me2/3. ChIP was conducted on chromatin extracts with antibodies that recognize different histone H3 lysine methylation marks, and IgG was used as a background control. Precipitated DNA was quantified by qPCR, and the DNA enrichment is shown as a percentage of IP/input. Multiple comparisons of mean values were performed using Tukey–Kramer honest significant difference test (*P* < 0.05), and different letters indicate significant differences. Two independent experiments were performed with similar results. Ab, Antibody. SSD4 and SSD65 are resistant and susceptible recombinant inbred lines, respectively.

To further understand how *ARG1* gene expression is regulated, we examined histone H3 lysine methylation (H3Kme) patterns within the *CARG* and *ARG1* exons, a region upstream of *CARG*, as well as the region shared by the *CARG* and *ARG1* transcripts (Figure 8A). H3K4 and H3K36 methylation marks are generally associated with active transcription, whereas H3K9 methylation is a repressive mark associated with transcriptional silencing (Kouzarides, 2007) and is often linked to both DNA methylation and NAT-mediated regulation of gene expression (Li et al., 2012; Bohmdorfer and Wierzbicki, 2015). In general, H3K9me2 is more prevalent in facultative heterochromatin in gene-rich regions and H3K9me3 is often associated with constitutive heterochromatin (Peters et al., 2002, 2003).

Chromatin immunoprecipitation (ChIP) was conducted using antibodies specific to H3K4, H3K36, and H3K9 di- and trimethylation, followed by qPCR designed to amplify precipitated products from the indicated regions of the *ARG1* and *CARG* genes to determine the level of chromatin modifications at these loci. At the 5′-UTR of *ARG1*, which covers the *CARG/ARG1* overlap region, H3K4me2, H3K4me3, and H3K36me3 levels were dramatically higher in the resistant genotypes SC283 and SSD4 and reduced in the susceptible genotypes TAM428 and SSD65 (Figure 8, B and C), closely tracking with the levels of expression of this *ARG1* gene. The chromatin of the *ARG1* exon was also significantly enriched for H3K4me2, H3K4me3, and H3K36me3 in the resistant genotypes, whereas the levels of these marks were reduced in the susceptible genotypes (Figure 8, B and C), correlating with the loss of *ARG1* expression in these genotypes. In contrast, despite the lower *ARG1* expression, H3K36me2 was enriched in the exon of *ARG1* chromatin of the susceptible genotypes (Figure 8C). Although H3K36 methylation is commonly associated with active transcription, previous studies have reported that it is also implicated in alternative splicing (Luco et al., 2010), which was observed in *ARG1* of the susceptible genotypes (Figure 6). These data suggest that H3K36 methylation plays a role in the alternative splicing of *ARG1* in the susceptible genotypes. Consistent with higher *CARG* gene expression, the *CARG* exon contained much higher levels of H3K4m2, H3K4me3, H3K36me2, and H3K36me3 in the susceptible genotypes versus the resistant genotypes (Figure 8, B and C).

H3K9 methylation is a repressive mark that is often triggered by small RNA (Holoch and Moazed, 2015). In contrast to H3K4 and H3K36 methylation, H3K9me2 and H3K9me3 levels were higher in the *CARG/ARG1* overlap region in the susceptible genotypes, which exhibited lower *ARG1* expression (Figure 8D). However, there were no significant differences in H3K9me2 and H3K9me3 at the *ARG1* exon in either genotype (Figure 8D). H3K9me2 and H3K9me3 levels at the *CARG* exon were significantly higher in the resistant lines in which *CARG* expression was reduced, whereas low levels of H3K9 methylation at the *CARG* exon were observed in the susceptible lines where *CARG* was highly expressed (Figure 8D). In all cases, the control experiment was conducted on the same IP protein DNA complex using the primers at the constitutive sorghum *Actin* gene (Sobic.001G112600), which showed no difference in the level of histone H3 lysine methylation (Supplemental Figure S11).

Due to the extensive polymorphisms of the upstream region of *CARG* in the resistant and susceptible genotypes, histone H3 lysine methylation was not examined in the 5′-upstream region of *CARG*. Overall, however, the patterns of histone lysine methylation that could be assayed correlated well with gene expression patterns, but it is not clear if these are the causes or consequences of the reduced gene expression.

### ARG1 confers resistance to fungal pathogens with distinct pathogenesis strategies

NLR-mediated resistance is often linked to plant immune responses to biotrophic and hemibiotrophic pathogens with race specificity (Jones and Dangl, 2006). To determine the specificity of ARG1, we tested the different genotypes for resistance to target spot, a fungal disease of sorghum caused by the necrotrophic fungus *Bipolaris sorghicola* (Supplemental Figure S12A)*.* Unexpectedly, the plant responses to *B. sorghicola* were similar to those for *Cs*. Similarly, ARG1 conferred resistance to sorghum rust disease caused by the biotrophic fungus *Puccinia purpurea* (Supplemental Figure S12B). This resistance is, therefore, broadly effective against three species of fungal pathogens with three distinct modes of infection and pathogenesis. Resistance to distinct groups of pathogens is unexpected given that NLRs are a class of proteins that are generally linked to race-specific resistance, and, in some cases, known to promote susceptibility to necrotrophic fungi (Coll et al., 2011). Therefore, we identified a single *NLR* gene that causes broad spectrum and complete resistance to multiple unrelated fungal pathogens.

### Comparative analysis of the ARG1 gene reveals distinct evolutionary relationships between resistant and susceptible genotypes

ARG1 encodes a typical NLR protein with N-terminal CC, NB-ARC, and LRR domains (Supplemental Data Set S7). Sequence comparisons revealed that ARG1 shares the highest sequence identify (54.61%) with RPP13-like protein in the wild rice species *Oryza brachyantha* (*Ob*), for which no functional data are available. RPP13 proteins from other plant species are also related to ARG1, with the Arabidopsis RPP13 showing significantly lower sequence identity (27.15%). We conducted phylogenetic analyses including ARG1, *Ob*RPP13, and 84 functionally validated CC-NLRs retrieved from RefPlantNLR (Kourelis et al., 2021) to explore the evolutionary relationships of ARG1 to other CC-NLRs from different species. This comprehensive analysis showed that ARG1 is closely related to *Ob*RPP13 (Supplemental Figure S13), which is consistent with their high sequence identity. Interestingly, Arabidopsis NRG1.2 closely clustered with ARG1. However, ARG1 and NRG1.2 share only 23.73% sequence identity. NRG1 was discovered in *Nicotiana benthamiana* because it was required for N protein-mediated resistance to tobacco mosaic virus (Peart et al., 2005). Arabidopsis RPP13 is a typical NLR that recognizes the *Hyaloperonospora parasitica* effector protein ATR13, which triggers resistance to biotrophic pathogens (Rentel et al., 2008).

A total of 397 NLR-encoding genes were identified from predicted gene models in the sorghum genome (Supplemental Figure S14 and Supplemental File S2), most of which were located on three chromosomes (Chr2, Chr5, and Chr8). In contrast, the CARG-deduced amino acid or DNA sequence is unique to the sorghum genome, with no similarity to other sequences in the database. Proteomic analysis of SC283 and TAM428 lines identified peptides that map to the ARG1 protein in the resistant lines, but no polypeptide was identified that maps to the *CARG* ORF in any of the genotypes. Furthermore, the putative 89 aa polypeptide that would be produced by that ORF would be unique to sorghum. Thus, although we cannot exclude the possibility that the ORF in *CARG* is translated, these data suggest that it is not and that the *CARG* transcript functions as a noncoding RNA.

**Figure 9:**
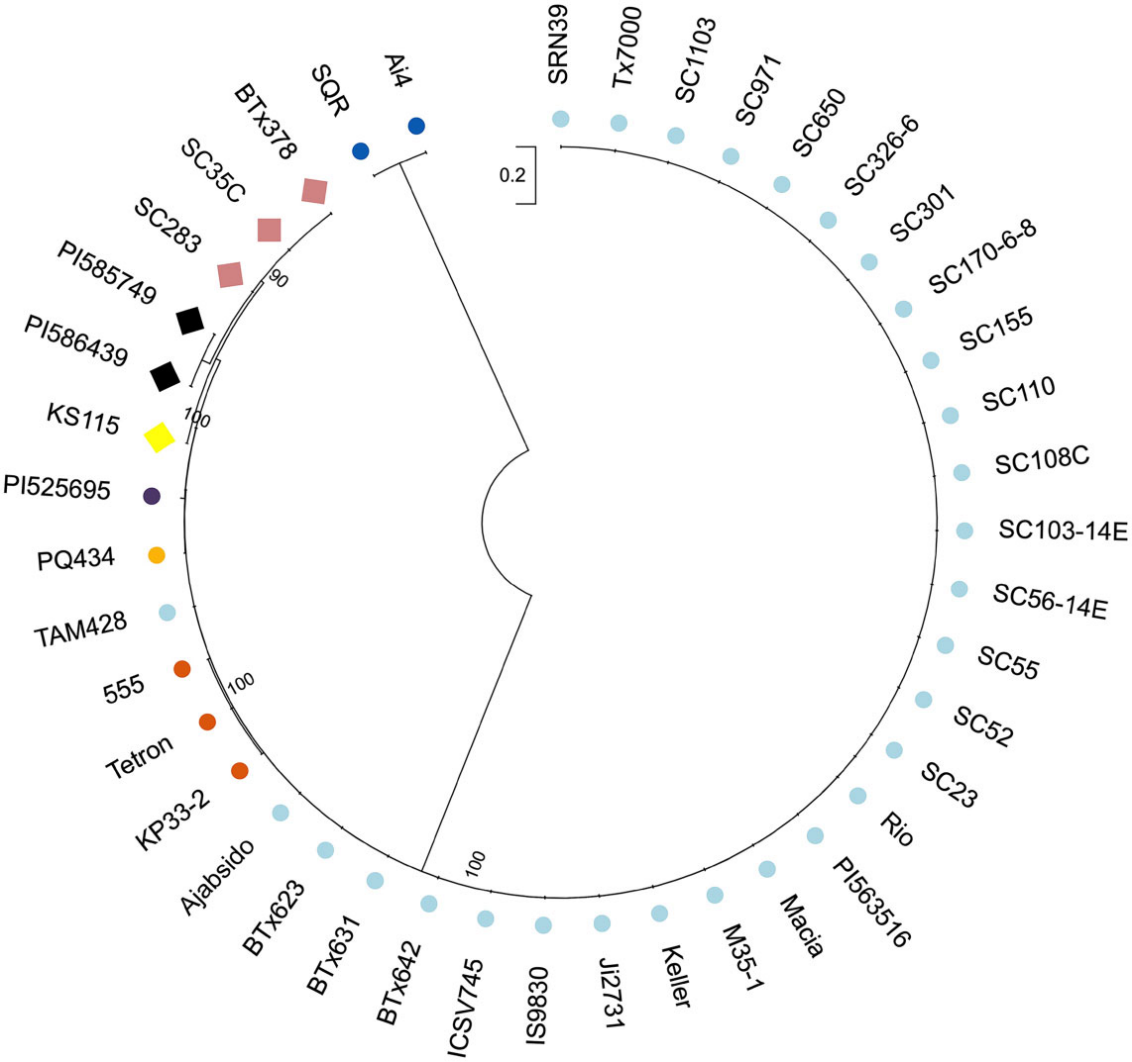
The maximum likelihood method and JTT matrix-based model analysis of the ARG1 amino acid sequences showing the evolutionary relationship of 42 different sorghum genotypes. Diamonds indicate resistance and circles indicate susceptible genotypes to *Cs* strain Csgl2. The ARG1 proteins were divided into eight sub-clusters and represented by different colors. Initial tree(s) for the heuristic search were obtained automatically by applying Neighbor-Join and BioNJ algorithms to a matrix of pairwise distances estimated using the JTT model, and then selecting the topology with superior log likelihood value. The tree is drawn to scale, with branch lengths measured in the number of substitutions per site. There were a total of 953 positions in the final data set. Evolutionary analyses were conducted in MEGA X. Bootstrap values are given at the node as a percentage of 1,000 replicates.

We next assessed the genetic relationship of the *ARG1* gene among many resistant and susceptible lines for which sequences were available from databases and our sequencing data. The phylogenetic relationship inferred from Maximum-likelihood analysis revealed a clear separation between the resistant and susceptible lines (Figure 9; Supplemental File S3). The resistant lines form three sub-clusters; SC283, SC35C, and BTx378 form one sub-cluster, while PI585749 and PI586439 form another sub-cluster. KS115 forms its own separate sub-cluster, which also includes the distantly related ARG1 from SC283, as shown by the large distance in the phylogenetic tree. The susceptible lines fell into five sub-clusters. TAM428 and 28 susceptible genotypes make up one sub-cluster. The second sub-cluster includes PQ434 on its own and a third sub-cluster includes only PI525695, which is not closely related to TAM428. The fourth sub-cluster is composed of 555, Tetron, and KP33-2, while the last sub-cluster consists of SQR and Ai4 (both originally collected from China), which were closely related to each other but were relatively distantly related to the other sub-clusters. These results point to variation within both the resistant and the susceptible sorghum genotypes.

Consistent with the above analyses, alignment of *ARG1* sequences of the 42 genotypes revealed that all resistant lines carry intact *ARG1* and are very similar to SC283, whereas *ARG1* from the susceptible lines were identical or nearly identical to *ARG1* from TAM428 or to SQR and Ai4, which have premature stop codons at different sites from TAM428 (Figure 2; Supplemental Figure S4 and Supplemental File S4). The pattern of sequence variation in the *ARG1* gene confirms the differences between the different genetic backgrounds coming from diverse origins, with the three subclusters in the resistant group showing clear differences in sequences. Despite extensive sequence searches, we identified no genotype that links the intact *CARG* NAT from the resistant *ARG1* allele.

## Discussion

Anthracnose is a major foliar diseases of sorghum that completely kills plants in the absence of resistance genes (Sherriff et al., 1995). Both the molecular mechanisms and the genes that regulate plant immunity to this pathogen are poorly understood. Here, we identified the *ARG1* gene, encoding a plant immune receptor that confers broad spectrum and complete resistance to several distinct fungal pathogens. *ARG1* is nested in an intron of a unique NAT, designated *CARRIER OF ARG1* (*CARG*), and the entire *CARG* and *ARG1* locus is flanked by MITE sequences that regulate the expression of *CARG* and *ARG1* transcripts. A combination of MITE insertions in the 5′- and 3′-*CARG* results in repressed expression of *ARG1*. The antisense NAT shares very limited sequence complementarity with the sense *ARG1* transcript apart from a short segment of 158 nucleotides in TAM428 and 159 nucleotides in SC283. The 3′-UTR of *CARG* in the susceptible genotype produces small RNAs whose functional significance is unclear. *CARG* and *ARG1* are transcribed in opposite orientations and exhibit inverse expression levels. The expression of the *ARG1* allele from susceptible genotypes is associated with an increase in the repressive H3K9 di- and trimethylation marks within the *CARG/ARG1* overlap region, as well as a reduction of these modifications in the *CARG* exon in the susceptible genotype. Abrogation of *CARG* expression is associated with the derepression of *ARG1* expression, which in turn correlates with increased histone H3K4 and H3K36 methylation levels within the single *ARG1* coding exon. In susceptible cultivars, basal transcription of *ARG1* and *CARG* is likely maintained through a combination of mechanisms including interference with transcription, dsRNA, NAT-mediated histone methylation, and repressive chromatin states.

It should be acknowledged that the primary lesion most likely to be responsible for susceptibility is the premature stop codons present in all of the susceptible genotypes. The loss of the conserved LRR domain likely results in a nonfunctional protein, and may also lead to nonsense-mediated decay of the *ARG1* transcript, which could explain its reduced steady-state levels (Lejeune, 2017). The increased level of *CARG* in susceptible lines may thus be a consequence of the loss of transcriptional interference due to reduced levels of *ARG1* in these genotypes. According to this scenario, the changes in expression levels and chromatin modifications would be a consequence rather than a cause of a mutation in *ARG1* that results in a loss of *ARG1* transcript. However, at least in our Arabidopsis transgenic plants, high levels of *ARG1* expression do not reduce the level of *CARG* in cis (Figure 7B).

There are a number of lines of evidence that suggest an alternative hypothesis, in which the NAT is a key player in the differentiation between resistant and susceptible genotypes. First, we note that all susceptible genotypes have both MITE insertions flanking the *CARG–ARG1* locus and point mutations in *ARG1*. The tight association between two genetic lesions in the NAT and the absence of both of them in the *ARG1* gene suggest that both lesions are required for resistance: one permits expression of the resistance gene due to the loss of the NAT, and the other permits high levels of expression of a functional *NBs-LRR* gene. However, because the polymorphisms in the two genes have not been separated, it is not possible at this time to determine whether both of them are required for the production of large quantities of functional ARG1 protein. The most straightforward way to determine this would be to genetically modify a resistant genotype such that *CARG* is expressed at high levels in situ. If this modification results in a susceptible phenotype despite the presence of an intact *ARG1* gene, it would be possible to conclude that the loss of the NAT is required for full resistance.

There are also other scenarios that are worth entertaining. NBs-LRR genes are often found at new locations in different accessions or related species (Luo et al., 2012), and many of these “transposed” genes are not functional, likely due to local sequence context. Indeed, *ARG1* is not present at a syntenic position relative to sorghum in the rice, *Brachypodium distachyon*, barley, teff, or *Setaria italica* genomes and is missing altogether from the maize genome, suggesting movement of this gene at some point in its evolutionary history. It is quite possible that *ARG1* moved to a new position in sorghum and did not provide disease resistance, perhaps due to the absence of a strong, inducible promoter. In some lineages (those with the InDel), a MITE insertion triggered expression of an long non-coding RNAs/natural antisense transcript (lncRNA NAT), which repressed any residual expression of *ARG1*, and point mutations resulted in a loss of functional *ARG1* altogether. In other lineages, relaxed purifying selection could have then resulted in the polymorphisms that may contribute to ARG1’s current unique broad-spectrum resistance. Subsequent strong selective pressure caused by disease could then have led to selection for a MITE insertion into *ARG1*, which both enhanced expression and made it inducible. According to this scenario, providing full resistance to the susceptible lines would require correction of both the *ARG1* and *CARG* lesions.

The nature of *ARG1* exon skipping is also unusual in that the skipping or the production of two transcripts from the same genomic template occurs in the absence of obvious well-defined intronic sequences in the *ARG1* gene. Many resistance genes are regulated by differential splicing where premature stop codons introduced by frame shifts result in variant transcripts encoding proteins lacking LRR repeats (Yang et al., 2014). However, the functions of these transcripts or truncated proteins in the susceptible backgrounds are unknown.

Proteins with canonical NLR protein structure mediate recognition of virulence effectors, which then activate a very strong and race-specific resistance that varies depending on the pathogen strain. *ARG1* encodes a typical NLR, which in SC283 and other resistant genotypes confers resistance to distinct pathogen groups. These include the obligate biotrophic fungus *Puccinia purpurea* (which causes sorghum rust), the hemibiotrophic fungus *Cs*, as well as the necrotrophic fungus *B.* *sorghicola* (which causes target spot in sorghum). Broad-spectrum resistance to multiple distinct pathogenic species with disparate virulence strategies and lifestyles is extremely uncommon. In fact, some NLRs are known to actually promote susceptibility to a variety of other necrotrophic fungi in sorghum and other plants (Lorang et al., 2007, 2012). It is possible that ARG1 recognizes a conserved effector that is common to different plant pathogen lineages. Alternatively, the derepression of ARG1 may activate an immune response that is broadly effective against many pathogens (Thomma et al., 2011). Ligand-independent resistance due to the derepression of ARG1 may also be possible. The broad-spectrum resistance in the sorghum cultivar BK7 was attributed to a QTL that maps to the *ARG1* chromosomal region, supporting a significant role for *ARG1* in existing sorghum cultivars (Felderhoff et al., 2016).

In eukaryotic cells, noncoding RNAs affect gene expression through transcription interference, RNA masking, dsRNA-dependent mechanism, RNA interference, or antisense-mediated methylation (Faghihi and Wahlestedt, 2009; Bohmdorfer and Wierzbicki, 2015). In Arabidopsis, the role of antisense transcripts (*COOLAIR*) in the cold-induced, epigenetic silencing of Arabidopsis *FLOWERING LOCUS C* (*FLC*), a regulator of the transition to reproduction, is linked to switching of chromatin states at *FLC* during vernalization (Csorba et al., 2014). Inference of transcription and consequent changes in chromatin have also been observed in other systems (Xue et al., 2014). Due to the complementarity of parts of the *CARG* 3′-UTR and *ARG1* 5′-UTR and the identification of small RNAs in the 3′-UTR of *CARG*, we suggest that the low levels of expression in susceptible genotypes may be due, at least in part, to sense–antisense interference, and that this process may result in the changes in chromatin modification that we observed in both genes.

H3K4 methylation of *ARG1* is significantly enriched in genotypes that show high levels of the expression of *ARG1*, as are H3K4 and H3K36 in the exon of *CARG* in genotypes that express high levels of that gene. Consistent with this finding, we observed enrichment of the repressive H3K9 methylation mark in the exon of *CARG* in resistant genotypes in which the expression of this gene is low. However, analyzing chromatin changes in the *CARG* promoter is complicated by the fact that the actual promoter region of this gene is poorly defined and is largely composed of transposable elements. Indeed, of the 1,500 bp of sequence upstream of the transcription start site in *CARG* of TAM428, only 26 bp are nontransposon sequences.

Clearly, additional studies are required to determine the degree to which changes in the expression of *CARG* mediate *ARG1* regulation, and the means by which changes in histone methylation caused, or are caused by, changes in gene expression. However, we did find clear evidence that changes in histone methylation are associated with changes in the expression of these two genes, although our comparative analyses of DNA and histone methylation was hampered by high polymorphism in the DNA sequence in resistant and susceptible genotypes.

Genetic studies have defined multiple loci that control resistance to *Cs* (Perumal et al., 2009; Murali Mohan et al., 2010; Felderhoff et al., 2016). However, the identification of specific resistance genes and their mechanisms of action has been slow in coming. Our findings are significant both because of their direct application for controlling widespread and economically significant sorghum diseases and because this pair of genes represents an unusual regulatory mechanism of a known class of immune receptors. Indeed, resistance associated with a loss of the NAT of an immune receptor gene is unique. In addition, the MITE insertion in the 5′-*ARG1* regulatory region confers inducible gene expression, adding to a growing body of evidence that transposable elements can be a significant source of regulatory information (Lisch, 2013).

Regardless of the molecular and cellular mechanisms involved, the *CARG–ARG1* locus provides a unique resistance locus that can be easily introgressed into a variety of sorghum cultivars using *CARG–ARG1*-specific molecular markers. The resistance provided by *ARG1* allele confers strong resistance to at least 10 distinct *Cs* strains tested, as well as 2 other fungal species. Transgenic expression of *ARG1* in susceptible but adapted varieties of crop plants may also provide broad-spectrum resistance. Genome editing of the *CARG* and *ARG1* genes in improved and adapted cultivars in order to generate broad-spectrum resistance will considerably shorten the breeding cycle and will make it possible to more precisely determine the means by which this unusual locus is regulated. A better understanding of the regulatory relationship between the NAT, *ARG1*, and the flanking MITEs will also likely provide important insights into the means by which novel patterns of gene regulation can rapidly evolve in plant genomes in response pathogens.

## Materials and methods

### Plant growth

The sorghum (*S.* *bicolor*) RILs were generated by crossing SC283 and TAM428 and advanced through single seed descent to the F6 generation and were maintained by self-fertilization. A total of 209 RIL lines were evaluated six times consecutively in the greenhouse. Plant growth conditions, methods of inoculation, and disease response assessments were as previously described (Prom et al., 2009). *Arabidopsis thaliana* Columbia-0 (Col-0) wild-type and the *CARG–ARG1* transgenic plants were generated as described previously (Mengiste et al., 1997) and grown in a growth chamber under a 12-h photoperiod with 140–150 μE m^−2^ s^−1^ of fluorescent light (Philips F32T8/ADV835/ALTO-T8) at 22°C and 60% relative humidity.

### Preparation of fungal cultures and plant disease assays

The *Cs* strains Csgl1 and Csgl2 were obtained from Dr Lisa Vaillancourt (University of Kentucky, Lexington). The other *Cs* strains are from different regions in Ethiopia and Nigeria (Supplemental Data Set S1). All strains were cultured on potato dextrose agar plates at 25°C. Fungal spores were harvested from 15- to 20-day-old cultures and suspended in ddH_2_O. The suspension was filtered through two layers of cheesecloth, and the concentration of spores was adjusted to 10^6^ spores·mL^−1^. The spore suspension was uniformly sprayed onto 3- to 4-week-old sorghum plants. Plants were kept in humidity chambers for 2 days and transferred to the greenhouse with a temperature setting of 28°C with a 16-h light duration and with occasional misting to maintain high humidity. Disease responses were scored by visual assessment of disease symptoms or resistance responses, chlorosis, and fungal growth in planta. The detached leaf disease assay for *Cs* was conducted by drop inoculation of spores on leaves placed on wetted absorbent or filter paper and incubated in sealed transparent trays. A drop (20 µL of 10^6^ spores·mL^−1^) of suspension was deposited on each leaf and disease evaluated by measuring lesion area and fungal growth. Total genomic DNA was isolated from *Cs*-inoculated leaves of each genotype using quick DNA extraction buffer (200-mM Tris–Cl, pH 7.5, 250-mM NaCl, 25-mM EDTA, pH 8, and 0.5% SDS). Fungal growth was assessed by qPCR amplification of the fungal rDNA and sorghum *Actin* as an internal control.

Rust (*Puccinia purpurea*)-infected sorghum leaves were collected from the Agronomy Center for Research and Education, West Lafayette, Indiana. The rust inoculum was maintained on rust-susceptible genotypes in the greenhouse. Inoculations and disease assays were conducted as described (White et al., 2014).

The target leaf spot fungus *B.* *sorghicola* isolates were obtained from Dr Burt H. Bluhm (University of Arkansas). The strain was cultured, harvested, and plants inoculated using the same method described for *Cs* strains. The concentration of spores was adjusted to 4 × 10^4^ spores·mL^−1^ and the plants were inoculated as previously described (Borges, 1983).

### Trypan blue staining

Leaf tissue samples from inoculated plants were collected for staining with trypan blue to reveal fungal growth in leaf tissue. The leaves were cleared in acetic acid: ethanol (1:3, v/v) solution overnight, followed by clearing using acetic acid: ethanol: glycerol (1:5:1, v/v/v) solution B for 3 h. The tissue was then stained with trypan blue (0.01% trypan blue in lactophenol) overnight. The stained tissue samples were rinsed multiple times and preserved in 60% glycerol for microscopic observation (Nikon ECLIPSE Ci).

### RNA-seq analysis

TAM428 and SC283 plants were grown in soil for 3 weeks and inoculated with Csgl2 (10^6^ spore·mL^−1^). At 0, 24, and 48 h after inoculation, the fifth leaves were collected from three biological replicates (approximately six plants each). Total RNA isolation was performed as described in the protocol of the Spectrum Plant Total RNA Kit with on-column DNase digestion (Sigma-Aldrich, USA), treated with DNase, and purified using the RNA Clean & Concentration TM-25 (ZYMO RESEARCH). The quality of the total RNA was determined by NanoDrop and an Agilent 2100 Bioanalyzer. For each sample, 3-µg total RNA was used to prepare the mRNA-seq library according to the TrueSeq RNA Sample Prep Kit protocol (Illumina). Library quality control and quantification were performed with an Experion DNA 1K Chip (Bio-Rad) and a Qubit fluorometer (Invitrogen), respectively. A total of 734,963,453 high-quality reads (average length = 99 bp) were generated using an Illumina HiSeq 2500 sequencer (Supplemental Data Set S2). For each library, 75 million 100-bp paired-end sequences were generated using an Illumina HiSeq 2500 sequencer. After removing low-quality sequences containing uncalled bases (Ns), we used Tophat2 (version 2.1.1) software (Kim et al., 2013) to align the RNA-seq reads against the reference genome of BTx623 (PhytozomeV10: Sbicolor_313_v3.1). Tophat2 alignment parameters were set to allow a maximum of two mismatches and to exclude reads mapping to more than one position on the reference genome. Moreover, only reads for which both pairs successfully aligned were considered. The gene counts were extracted using the HTSeq python tool (Anders et al., 2015). Differential expression analyses were performed using the EdgeR package (Robinson et al., 2010) using empirical Bayesian methods. To filter out weakly expressed genes, only those genes with a minimum expression level of 1 RPKM (reads per kilobase per million mapped reads) in three replicates were included in the analysis. Genes with a LogFC > 1 (2-fold change) and false discovery rate < 0.05 and *P* < 0.05 were considered to be differentially expressed between conditions. To assess the variability among samples, we performed hierarchical clustering and dispersion analysis based on biological coefficient of variation. Hierarchical clustering was performed based on Euclidean distances. Dispersion was conducted using top 2,000 values in the EdgeR software package.

### Functional classification analysis

To annotate entire gene sets of the sorghum and *Cs* genomes accurately, all protein sequences were analyzed using InterProScan 5.8-49.0 (Jones et al., 2014). We then used agriGO (http://bioinfo.cau.edu.cn/agriGO/) and ReviGO (http://revigo.irb.hr/) (Du et al., 2010; Supek et al., 2011) to identify the putative biological functions and biochemical pathways for the differentially expressed genes (DEGs) and to find statistically overrepresented gene ontology terms. To expand our functional analysis of the DEGs, we used MapMan software (http://mapman.gabipd.org) for visualization and biochemical pathway overlays as previously described (Lei et al., 2014). For MapMan analysis, all gene identification labels were converted into Sbicolor_79 label based on Sbicolor 3.1 annotation files (PhytozomeV10: Sbicolor_313_v3.1. synonym).

### DNA isolation and whole genome sequencing

Among the RILs, 50 resistant and 50 susceptible plants were selected and used to construct two DNA bulks (RB and SB). To build the reference sequence, eight sorghum cultivars (Supplemental Data Set S4) were sequenced. For DNA extraction, 100-mg fresh leaf was harvested from each selected seedling and DNA was isolated using a DNeasy Plant Mini Kit (Qiagen, USA). Approximately 100-ng DNA from each sample was combined to construct two independent DNA bulks. The two DNA bulks were purified with a DNA clean-up & Concentration Kit (ZYMO Research, USA). A genomic DNA library was prepared for each DNA bulk using an Illumina TruSeq DNA Sample Preparation Kit (Illumina Inc., San Diego, CA, USA) according to the manufacturer’s protocol. Each DNA library was sequenced on the Illumina HiSeq 2500 sequencing platform.

### Bulk DNA sequencing and QTL analysis

The raw DNA-seq reads were trimmed and filtered to remove low-quality sequences using Fastx-tools (Pearson et al., 1997). Reads with a quality threshold lower than 30 and those shorter than 40 bp were discarded. The short reads from the two DNA bulks that passed the quality control were aligned to the reference genome of BTx623 (Phytozome V10: Sbicolor_313_v3.1) using BWA software (Version 0.7.12; Li and Durbin, 2009). Reads that aligned to more than one position in the reference genome were filtered out. The files were converted to BAM files using SAM tools (Li et al., 2009), sorted, and compared to locate duplicate records using Picard software (http://picard.sourceforge.net). Re-alignment (BAQ) was done to avoid false SNP calls near InDels. The resulting files were applied to GATK SNP-calling (version 3.3; McKenna et al., 2010; DePristo et al., 2011). SNP annotation was performed using SnpEff (version 4.1; Cingolani et al., 2012) with the sorghum annotation file (PhytozomeV10: Sbicolor_255_ v2.1.gene.gff3). A total of 11,170 variants, including 9,567 SNPs, 755 insertions, and 848 deletions, were annotated in the QTL region. QTL analysis was performed as previously described (Takagi et al., 2013). The sorghum reference sequence was reconstructed by replacing nucleotides in BTx623 with the 1,826,960 SNP positions identified between the eight cultivars by aligning the short reads to the reference genome of BTx623 (PhytozomeV10: Sbicolor_313_v3.1). SNP-index was calculated at all SNP positions with Coval. All steps were performed using the QTL-seq_framework1.4.4 pipeline (http://genome-e.ibrc.or.jp/home/bioinformatics-team/mutmap;  Takagi et al., 2013). Slide window analysis was applied to SNP-index plots with 2-Mb window size and 50-kb increment.

### Identification of InDels, primer design, and marker analysis

To identify potential InDel markers between SC283 and TAM428, we detected sequence polymorphisms between them using the genome browser Integrative Genomics Viewer. Primer3Plus (http://www.bioinformatics.nl/cgi-bin/primer3plus/primer3plus.cgi) was used to design PCR primers with a length of 18–27 bp, GC content of 40%–60%, and PCR products of 100–700 bp. For marker analysis, genomic DNA was isolated from each selected RIL and parental line using a DNeasy Plant Mini Kit (Qiagen, USA). PCR amplification was performed in a 20-μL reaction containing 1×PCR Buffer (1.5-mM MgCl_2_), 0.1 mM each dNTP, 0.5 μM of forward/reverse primers, 20–40 ng of genomic DNA, and 1.0 U GoTaq DNA polymerase (Promega, USA).

### ChIP-qPCR

ChIP experiments were performed as described previously with minor modifications (Saleh et al., 2008). Leaf tissues (1.5 g) from 3-week-old plants were fixed with 1% (v/v) formaldehyde for 40 min at room temperature, and the chromatin samples were sonicated to yield 200–1,000-bp fragments. After pre-clearing of the chromatin samples with salmon sperm DNA/protein A agarose beads (EMD Millipore), immunoprecipitations were carried out with the appropriate antibodies to histone lysine methylation and reverse cross-linking overnight at 65°C. Immunoprecipitated DNA samples were purified using a silica membrane column (MACHEREY-NAGEL Inc.) and eluted in 60-μL elution buffer. For qPCR, 2 μL of DNA was amplified using SYBR Green Supermix (Bio-Rad) with specific primers, as listed in Supplemental Data Set S8. The data are presented as percentage of input values. The antibodies used for the ChIP experiments were: H3K4me2 (07-030, EMD Millipore), H3K4me3 (07-473, EMD Millipore), H3K9me2 (ab1220, Abcam), H3K9me3 (07-442, EMD Millipore), H3K36me2 (07-369-I, EMD Millipore), H3K36me3 (ab9050, Abcam), and IgG (sc‐2027, Santa Cruz) as a negative control.

### RNA isolation

Total RNA was isolated from *Cs*-inoculated leaves and mock- or 2-nM chitin-treated leaves of each sorghum genotype and from leaves of each Arabidopsis transgenic line using TRI reagent (Molecular Research Center Inc.) according to the manufacturer’s instructions. The total RNA concentration and quality were measured using a NanoDrop 2000c spectrophotometer (Thermo Fisher Scientific).

### RT-PCR and RT-qPCR analysis

After DNase I treatment (NEB), cDNA was synthesized from 2 μg of total RNA using M-MLV Reverse Transcriptase (Promega) according to the manufacturer’s protocol. For RT-PCR, the PCRs were carried out using GoTaq DNA Polymerase (Promega) with the primers listed in Supplemental Data Set S8. The reactions (Applied Biosystems 2720 Thermal Cycler) for the *ARG1* and *Actin* genes consisted of 25, 28, 31, and 34 cycles in three steps: 95°C for 30 s, 57°C for 30 s, and 72°C for 2 min (*ARG1* gene) or 30 s (*Actin* gene). Amplified PCR products were loaded on 1.5%–2.0% agarose gels, and bands were visualized by ethidium bromide staining. For RT-qPCR, reactions were performed using SYBR Green Supermix (BIO-RAD) with the primers listed in Supplemental Data Set S8, following the manufacturer’s instructions.

### Generation of swapping constructs of *CARG–ARG1* loci and plant transformation

A total of 6 *CARG–ARG1* loci constructs were made in the pEarleyGate 104 (pEG104) vector backbone using an In-Fusion HD Cloning Kit (Clontech). These six constructs, SC283, TAM428, S1, S2, S3, and S4, are schematically represented in Figure 7B. The full-length and partial DNA amplification of *CARG–ARG1* loci were carried out by PCR to generate DNA fragments for P1, P2, and P3 from both SC283 and TAM428, P4 from TAM428, P5 from SC283, P6 from TAM428, and P7 from SC283. These DNA fragments contained 15-bp overlapping regions to allow homologous recombination between DNA fragments. The In-Fusion reaction was conducted using mixed appropriate DNA fragments and linearized pEG104 according to the manufacturer’s instructions. The primers used in this work are listed in Supplemental Data Set S8. All six constructs were transformed into *Agrobacterium tumefaciens* GV3101, and subsequently, 5-week-old plants were used for *Agrobacterium*-mediated transformation by the floral dip method (Clough and Bent, 1998).

### Construction of the GUS reporter gene-fusion plasmid, transfection, and GUS activity assay

A *Nae*I/*EcoR*I fragment of pSAT1-Pnos-Venus-N (E4042) modified by introducing the GUS reporter and removing the nos promoter was used as a vector for introducing the *ARG1* and *CARG* promoters: the 1,635-bp TAM428 *ARG1* promoter; the 1,723-bp SC283 *ARG1* promoter; the 1,153-bp TAM428 *CARG* promoter; and the 1,194-bp SC283 *CARG* promoter. Intron-deleted promoter-5′-UTR products were generated by performing a PCR-driven overlap extension method. Each gene segment in the promoter-5′-UTR region was spliced by segment-specific primers. The primers except for ARG1-SC283-NaeI (-1722F) and ARG1-EcoRI (-1R) generated overlapping regions by including nucleotides that span the junction of each segment. The second or third PCR generated the intronless versions of *ARG1* promoter-5′-UTR products, which that were then inserted into the same vector used for promoter-GUS construction. All constructs were verified by sequencing and the plasmid DNA for transfection prepared using a Qiagen Plasmid Midi Prep kit. Primers used for the constructions are listed in Supplemental Data Set S8.

Approximately 1 × 10^5^ Arabidopsis protoplast in 0.2 mL of MMg solution (0.4-M mannitol, 15-mM MgCl_2_, and 4-mM MES, pH 5.7) were mixed with 10 μg of each plasmid DNA and an equal volume of PEG solution (40% [wt/vol] PEG4000; Fluka, 0.2-M mannitol and 0.1-M CaCl_2_) for 5 min at room temperature. After incubation, the protoplasts were washed three times with 1 mL of W5 solution (154-mM NaCl, 125-mM CaCl_2_, 5-mM KCl, 5-mM glucose, and 2-mM MES, pH 5.7). The protoplasts were resuspended gently in 1 mL of W5 and incubated at room temperature for 16 h. Fluorescence intensity (GUS and luciferase activity) was determined using a Tecan Infinite M200 pro microplate reader (Tecan). Co-transfection with a CaMV 35S:luciferase plasmid was used to determine the transfection efficiency. All transfection assays were performed at least in triplicate and repeated three times.

### 5′- and 3′-RACE

A RACE experiment was performed on total RNA samples using a SMARTer RACE 5′/3′ Kit (TaKaRa) according to the manufacturer’s protocol. Briefly, 1-μg RNA was treated with 5′-CDS Primer A or 3′-CDS Primer A at 72°C for 3 min and cooled to 42°C for 2 min. For the 5′-RACE cDNA synthesis reaction, 1 µL of the SMARTer II Oligonucleotide was added to the 5′-CDS Primer A treated RNA sample. The denatured RNAs were reverse transcribed with 8 µL of Master Mix at 42°C for 90 min and at 70°C for 10 min. The cDNAs were amplified by PCR. The PCR products were detected by agarose gel electrophoresis and purified using a PCR purification kit (MACHEREY-NAGEL Inc.). The PCR products were sequenced, and sequencing results were compared with the genomic sequences.

### DNA methylation analysis

Leaves of three plants per line were selected for DNA isolation. DNA was extracted from 4-week-old leaves using a DNeasy Plant Mini Kit (Qiagen), and DNA (200 ng) was used for bisulfite conversion using an EpiTect Bisulfite kit (Qiagen). The converted DNAs were used for PCR to evaluate the methylation status of *ARG1*, *CARG*, and *Actin* genes with specific primer sets. The primers are shown in Supplemental Data Set S8. The amplified products were gel purified (Gel Extraction kit; MACHEREY-NAGEL Inc.), ligated into the pGEM-T Easy Vector (Promega), and transformed into *Escherichia coli*. The plasmid DNAs were isolated and sequenced using the T7 or M13 forward primers.

### Small RNA-seq analysis

We applied an informatics pipeline to filter plant siRNAs and miRNAs from the complete set of small RNAs. A total of 228,228,937 distinct small RNA reads from 12 sorghum libraries with *Cs* or mock-inoculated plants were analyzed using the pipeline. As a first step, the adaptors and low-quality reads were removed using FASTX-Toolkit (Gordon and Hannon, 2010). The next step involved removing structural RNAs such as tRNAs and rRNAs. The third step involved selecting the RNA read sizes between 18 nt and 28 nt. The fourth step was to remove low-abundance small RNAs (retaining only those with less than 10 transcripts per million in at least one of 12 libraries), *Cs* genome reads, as well as highly repetitive small RNAs (those with more than 20 hits to the genome). A total of 121,338 distinct small RNAs were retained. Finally, miRDeep-P (Yang and Li, 2011) was employed to detect predicted miRNAs. To identify consistent miRNAs, all small RNA libraries were separately processed based on the above method. miRNAs were considered to be candidate miRNAs if they could be detected in three libraries with the same treatment in SC283 or TAM428. To further verify our predicted miRNAs, highly similar homologs in miRBase V21 were identified using Segemel (Hoffmann et al., 2009). miRNAs that passed all filter processing steps were identified as novel miRNAs. All small RNA-seq reads were aligned against the BTx623 reference genome (PhytozomeV10: Sbicolor_313_v3.1) using Tophat2 (version 2.1.1) software (Kim et al., 2013).

### Statistical tests

Statistical parameters are presented in the figures and figure legends. Analyses of gene expression, fungal biomass, and ChIP-qPCR data were performed using at least three replicates, and data are shown as mean ± standard deviation (sd). Statistically significant differences were determined by least significant difference, Student’s *t* test, and Turkey’s honest significant difference test. Significance was considered to be *P <* *0.05, **P <* *0.01. The statistical analysis was performed using JMP 9.0, JMP 16, and GraphPad Prism 6.0 software. Summaries of statistical analyses are provided in Supplemental File S5.

### Accession numbers

The RNA-seq and microRNA-seq data have been submitted to NCBI https://www.ncbi.nlm.nih.gov/bioproject/PRJNA667277 under accession number PRJNA667277. The nucleotide sequences of the *CARG–ARG1* loci were sequenced or downloaded from NCBI. Additional data related to this paper may be requested from the authors.

## Supplemental data

The following materials are available in the online version of this article.

**Supplemental Figure S1.** SNP-index and Δ (SNP-index) plots for 10 chromosomes of bulked DNA from resistant and susceptible recombinant inbred sorghum lines.

**Supplemental Figure S2.** Location and sequences of MITEs in the *CARG–ARG1* locus.

**Supplemental Figure S3.** *ARG1* and *CARG* gene expression in resistant and susceptible genotypes.

**Supplemental Figure S4.** Amino acid sequence alignment of ARG1 protein from different resistant and susceptible genotypes.

**Supplemental Figure S5.** Disease responses of sorghum genotypes carrying different *ARG1* alleles.

**Supplemental Figure S6.** Disease responses of F2 plants after drop inoculation with *Cs* spores.

**Supplemental Figure S7.** Sequence alignments of *ARG1* alleles from different genotypes.

**Supplemental Figure S8.** The 3′-UTR of the *CARG NAT* gene produces small RNAs.

**Supplemental Figure S9.** Small RNAs derived from the 3′-UTR of the Natural Antisense RNA transcript gene in susceptible genotype show similarity to sorghum miR6225.

**Supplemental Figure S10.** DNA methylation analysis of *ARG1* exons in SC283 and TAM428.

**Supplemental Figure S11.** H3K4, H3K9, and H3K36 di- and tri-methylation at the sorghum *Actin* gene in anthracnose resistant and susceptible lines.

**Supplemental Figure S12.** ARG1 confers resistance to the fungal diseases target spot and rust.

**Supplemental Figure S13.** Phylogenetic analysis of ARG1 with 85 other CC containing NLR receptors.

**Supplemental Figure S14.** Phylogenetic trees of NLR genes from Sorghum.

**Supplemental Data Set S1.** Sorghum natural variants used in the study.

**Supplemental Data Set S2.** Responses of sorghum cultivars to different isolates of *Cs*.

**Supplemental Data Set S3.** Phenotyping of RILs and parents for *Cs* resistance.

**Supplemental Data Set S4.** Summary of Illumina Hiseq 2500 sequencing for SC283 x TAM428 RILs and eight sorghum cultivars.

**Supplemental Data Set S5.** Genotyping of RILs using 12 InDel markers. The resistant allele (SC283) is marked 1; the susceptible allele (TAM428) is marked 2. Rec. is recombination.

**Supplemental Data Set S6.** Sequence variants within the 5'-UTR, exon, and 3'-UTR of predicted 29 genes in the 10–11 Mb on chromosome 7 between SC283 and TAM428.

**Supplemental Data Set S7.** List of domain hits by NCBI conserved domains, SMART, and InterPro analyses.

**Supplemental Data Set S8.** List of primers used in this study.

**Supplemental File S1.** Genomic structures and sequences of *CARG–ARG1* genes in TAM428 and SC283.

**Supplemental File S2.** Text file of protein sequences used for the phylogenetic analysis shown in Supplemental Figure S14.

**Supplemental File S3.** Text file of protein sequences used for the phylogenetic analysis shown in Figure 9.

**Supplemental File S4.** Alignment of *ARG1* sequences from different genotypes.

**Supplemental File S5.** Summary of statistical analyses.

## Funding

This study was made possible through funding by the Feed the Future Innovation Lab for Collaborative Research on Sorghum and Millet through grants from American People provided to the United States Agency for International Development (USAID) under cooperative agreement No. AID-OAA-A-13-00047. The contents are the sole responsibility of the authors and do not necessarily reflect the views of USAID or the US Government. We also acknowledge grant from the National Science Foundation (NSF, IOS-1916893) to T.M. and Hatch funding to D.L.

*Conflict of interest statement*. The authors declare that they have no competing interests.

## Supplementary Material

koab305_Supplementary_Data

## Notes

S.L. conducted marker analysis, SNP and InDel analysis, mapping and characterization of the *CARG–ARG1* loci, construction of all *CARG–ARG1* plasmids, generation of *CARG–ARG1* Arabidopsis transgenic plants, reporter gene assays, the genetic screen and disease assays on independent sorghum lines, F2 population, and RILs, ChIP, DNA methylation, RACE, alternative splicing, and gene expression analyses, and phylogenetic analysis. F.F. conducted the genetic screen, QTL-seq, bioinformatic analysis, SNPs and InDels analysis, gene expression analyses, disease assays on the RILs, phylogenetic analysis, and small RNA identification. C-J.L. conducted the reporter gene assay. A.A., D.B.M., conducted the additional disease assays, made the genetic crosses, and analyzed the genetic segregation data. T.M., S.L., F.F., G.E., D.L. designed the research, analyzed the data, and wrote the paper.

The author responsible for distribution of materials integral to the findings presented in this article in accordance with the policy described in the Instructions for Authors (https://academic.oup.com/plcell) is: Tesfaye Mengiste (mengiste@purdue.edu).
