# Supplementary material for: Broad-spectrum fungal resistance in sorghum is conferred through the complex regulation of an immune receptor gene embedded in a natural antisense transcript
Source: Plant Cell. 2022 Jan 9;34(5):1641–65. doi: 10.1093/plcell/koab305 (PMC9048912; doi:10.1093/plcell/koab305)
Supplement: koab305_Supplementary_Data [file koab305_supplementary_data.zip › tpc.21.00809_SupplementalFigures.pdf]

Supplemental Data. Lee, Fu et al (2022). Broad spectrum fungal resistance in sorghum is conferred through the complex regulation of an immune receptor gene embedded in a natural antisense transcript. Plant Cell.

A

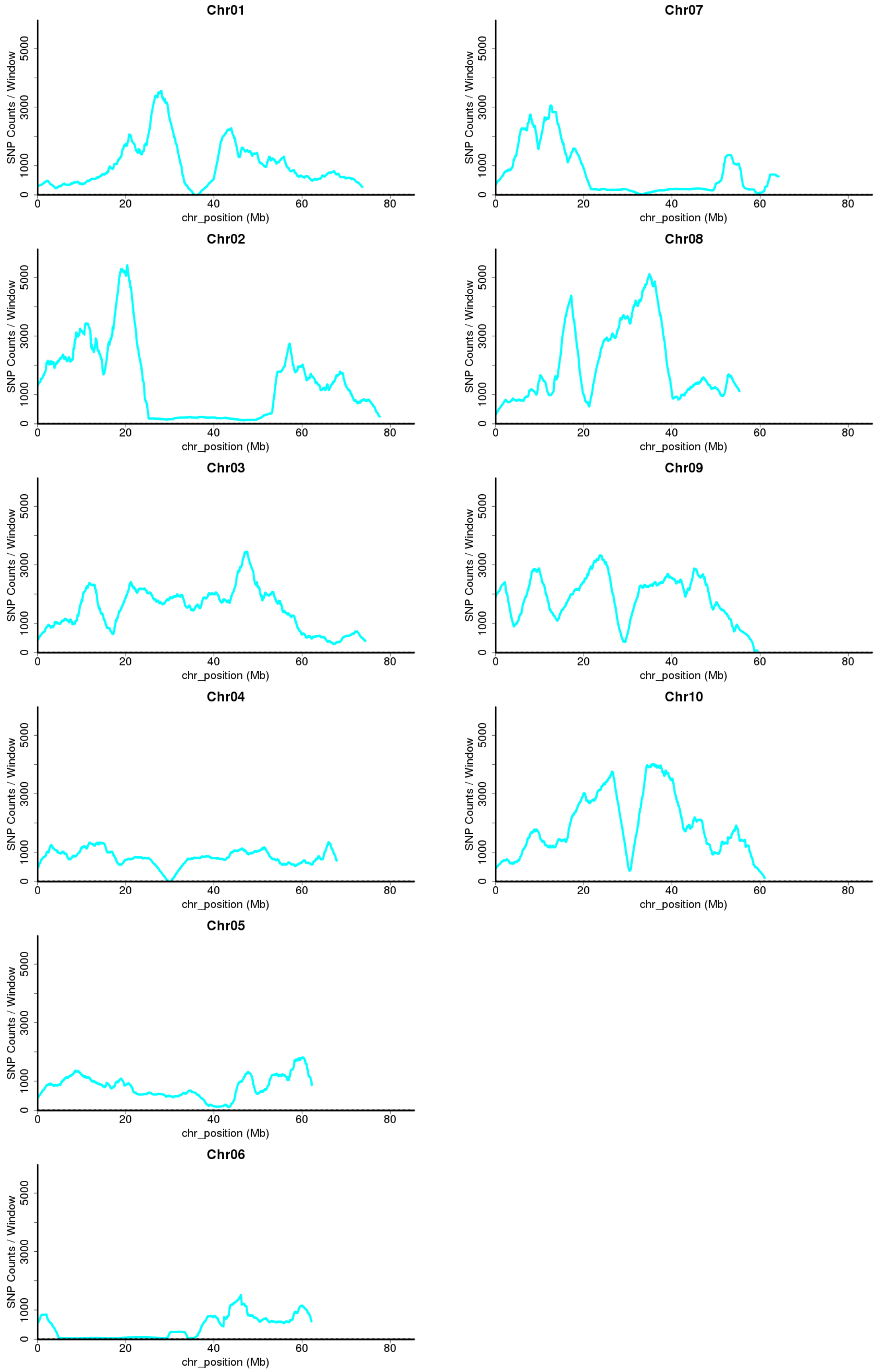

B

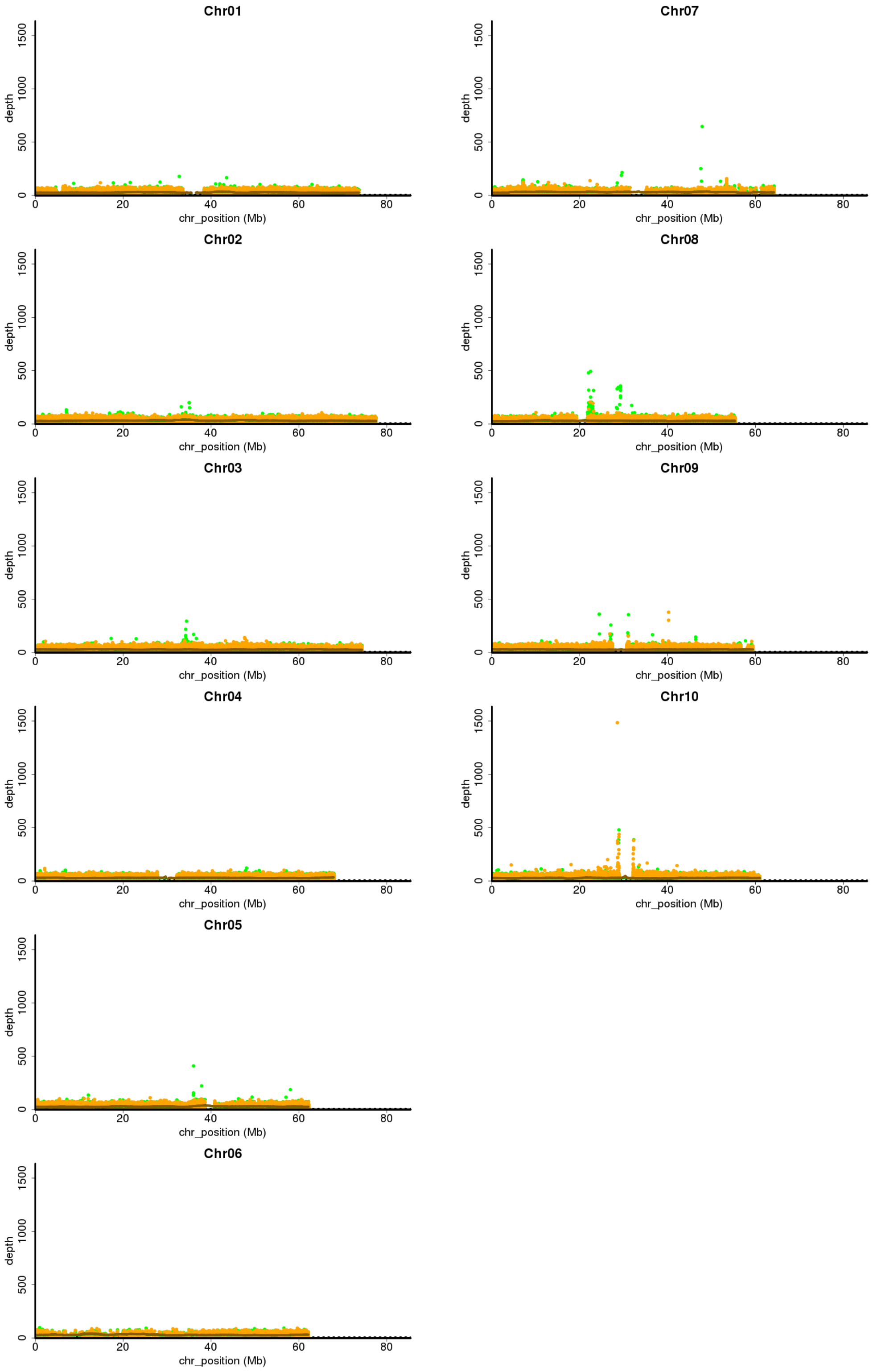

Chr01
Chr02
Chr03
Chr04
Chr05
Chr06
Chr07
Chr08
Chr09
Chr10
depth
0
500
1000
1500
chr_position (Mb)
0
20
40
60
80

C

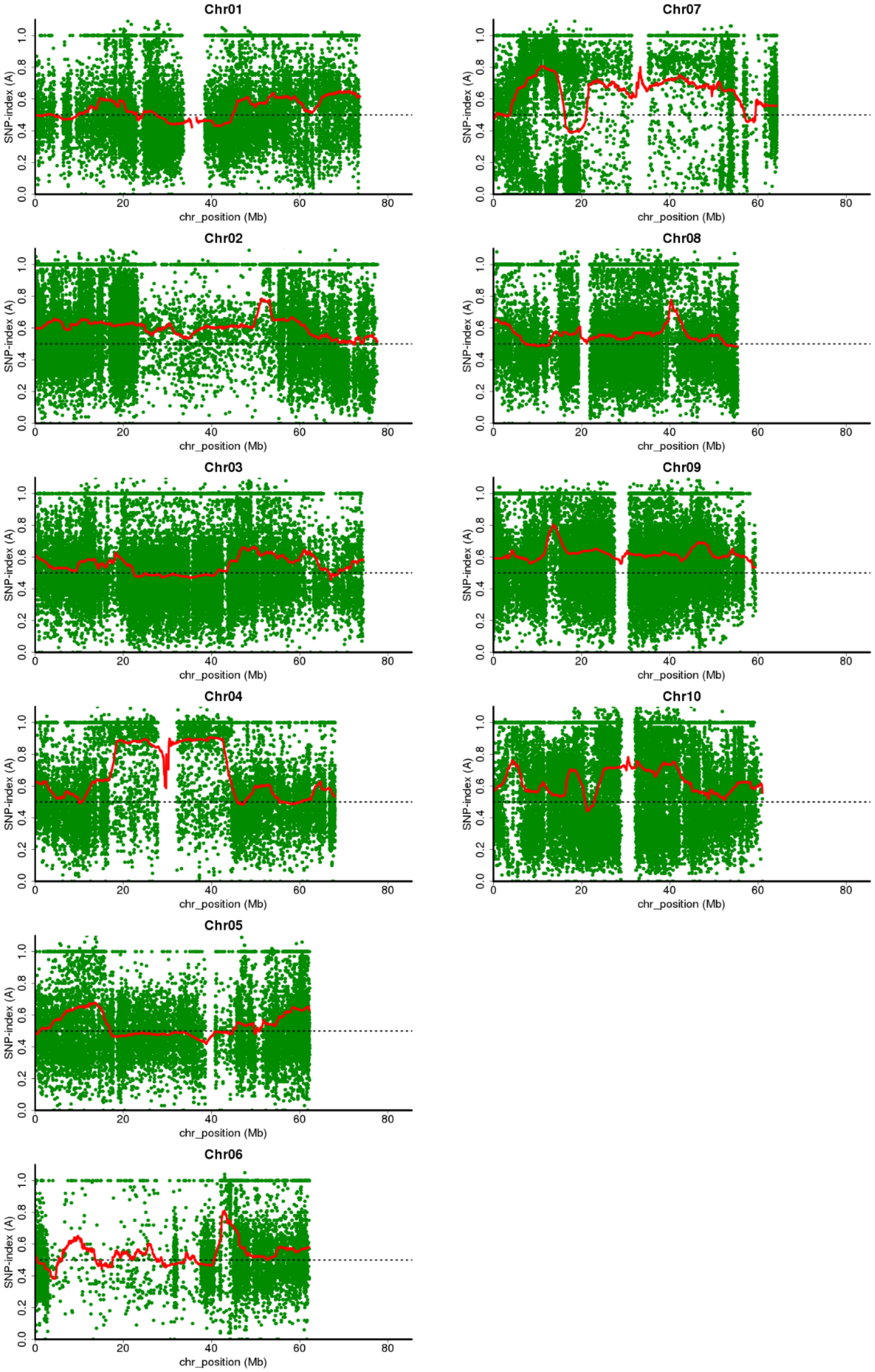
Chr01
Chr02
Chr03
Chr04
Chr05
Chr06
Chr07
Chr08
Chr09
Chr10
SNP-index (A)
chr_position (Mb)

D

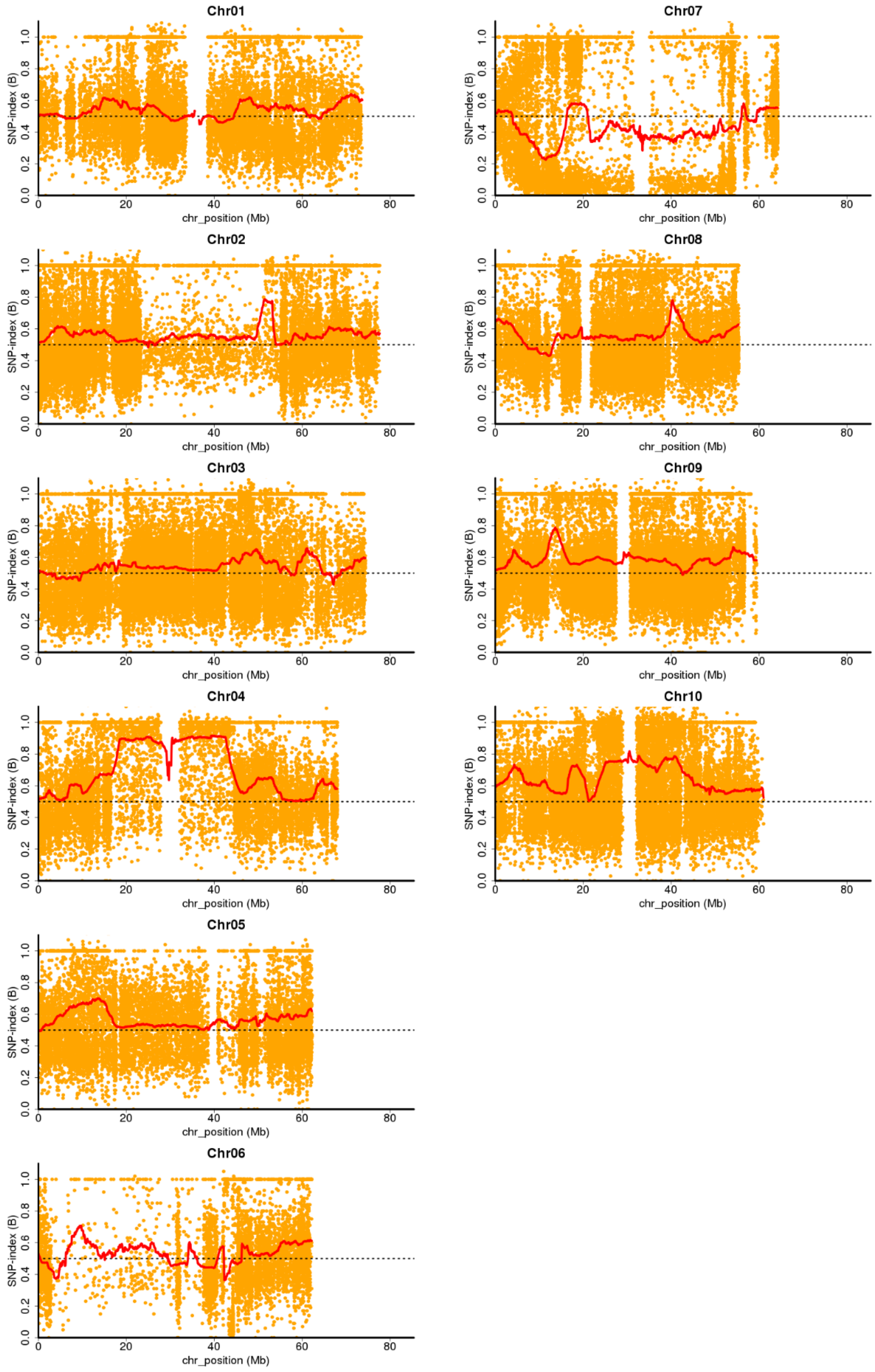
Chr01
Chr02
Chr03
Chr04
Chr05
Chr06
Chr07
Chr08
Chr09
Chr10
SNP-index (B)
chr_position (Mb)
0.0
0.2
0.4
0.6
0.8
1.0
0
20
40
60
80

E

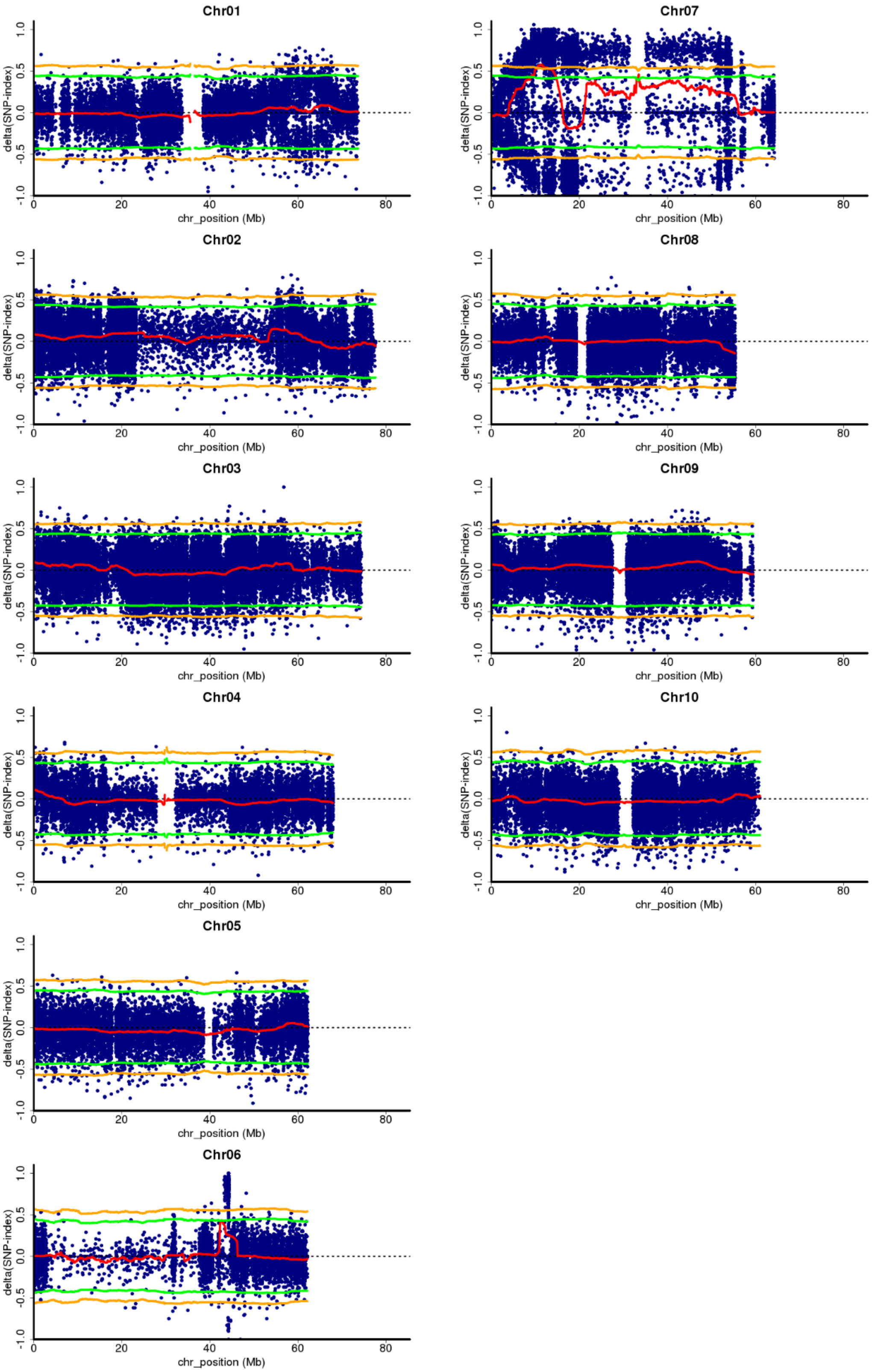
Chr01
Chr02
Chr03
Chr04
Chr05
Chr06
Chr07
Chr08
Chr09
Chr10
delta(SNP-index)
chr_position (Mb)
1.0
0.5
0.0
-0.5
-1.0
0
20
40
60
80

Supplemental Figure S1. SNP-index and ∆ (SNP-index) plots for 10 chromosomes of bulked DNA from resistant and susceptible recombinant inbred sorghum lines.

(A) The SNP density for bulked DNA of the RILs. **(B)** The SNP depth for bulked DNA of the RILs. **(C)** The SNP-index of resistant bulk. **(D)** The SNP-index of susceptible bulk. **(E)** The ∆(SNP-index) plot obtained by subtraction of S-bulk SNP-index from R-bulk SNP-index for RILs. Statistical confidence intervals under the null hypothesis of no QTL are shown (orange: $P < 0.1$; green: $P < 0.05$). Single nucleotide polymorphism (SNP)-index plots of R-bulk and S-bulk, and ∆ (SNP-index) plot of all sorghum chromosomes. The ∆ (SNP-index) plot was obtained by subtraction of S-bulk SNP-index from R-bulk SNP-index for RILs. The DNAs from resistant or susceptible plants were bulked to make separate resistant (R) and susceptible (S) DNA bulks. S-bulk, DNA from the susceptible plants, R-bulk, DNA from the resistant plants. SNP index and ∆ (SNP-index) was determined as described.

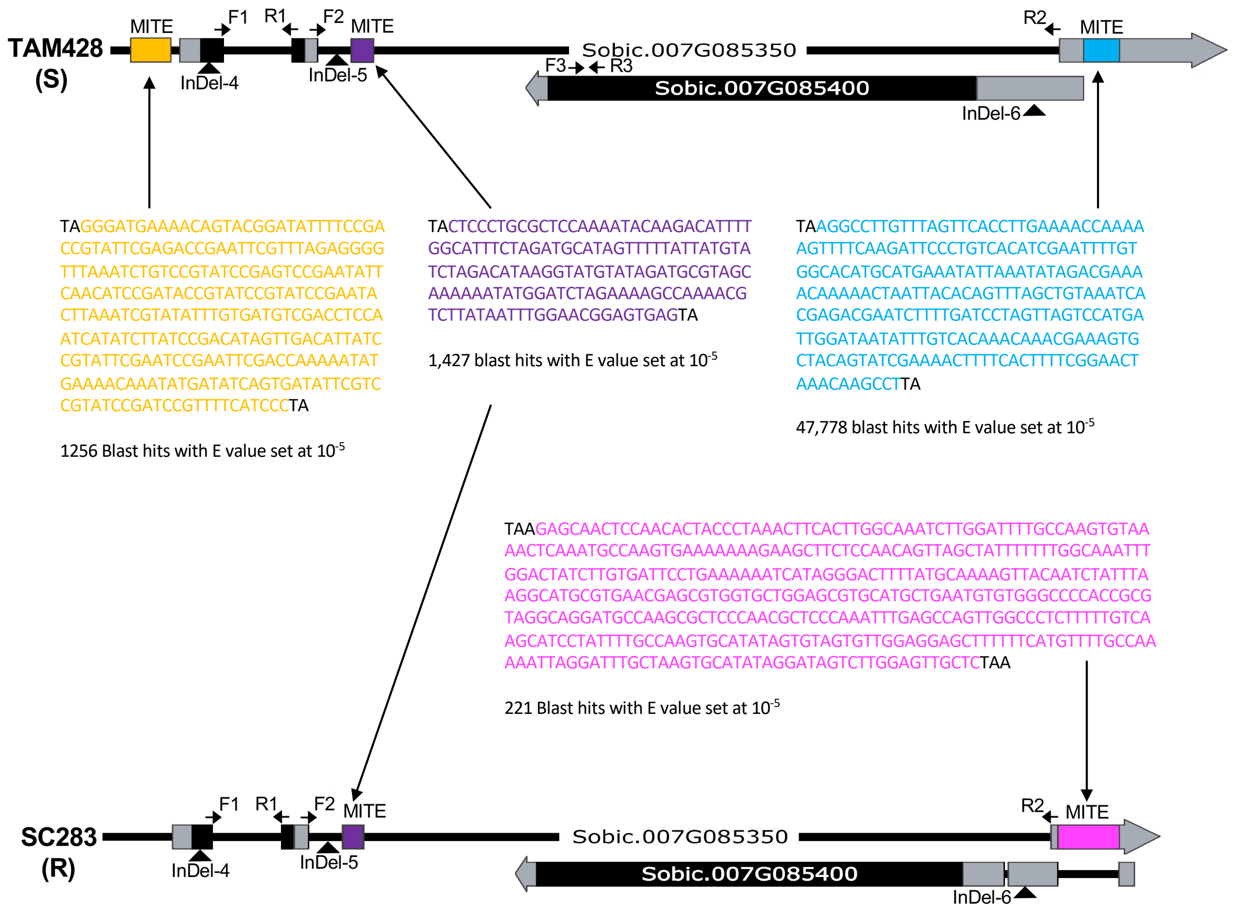

Supplemental Figure S2. Location and sequences of Miniature Inverted-repeat Transposable Elements in the *CARG-ARG1* locus.

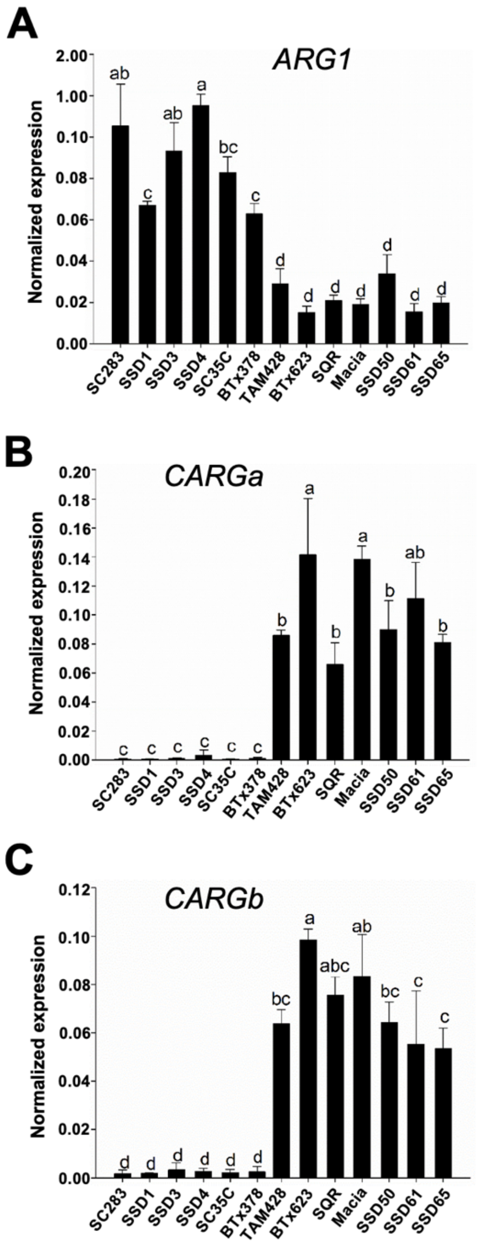

Supplemental Figure S3. *ARG1* and *CARG* gene expression in resistant and susceptible genotypes.

(A-C) Expression of *ARG1* **(A)** and *CARG* **(B and C)** genes in sorghum genotypes with different *CARG* alleles. qRT-PCR data was normalized to the constitutively expressed sorghum *Actin* gene (Sobic.001G112600) as a constitutive control. qRT-PCR was performed using *CARG* and *ARG1* gene specific primers. The qRT-PCR results are obtained with primers flanking the large intron (F2-R2, **B**) and the first exon of *CARG* (F1-R1, **C**). The F2-R2 primers were designed to flank the intron of the *CARG* to verify the identity of the transcript. Error bars indicate the standard deviation from three technical replicates of three independent biological repeats (n = 9). Error bars show ± SD (n = 9). Letters indicate significant difference based on the Least Significant Difference (LSD) ($P < 0.05$). Similar results were obtained in two independent experiments.

Supplemental Data. Lee, Fu et al (2022). Broad spectrum fungal resistance in sorghum is conferred through the complex regulation of an immune receptor gene embedded in a natural antisense transcript. Plant Cell.

```
TAM428      1 MGSVLFSLSSKCLDKLAVLLEEEIMMTLSVRKEIRKLHDYLKYFDSIHEDADARAMEHREMTGIWWGDVKDVMYDVDDIIDLLRAHSQKQRCCDLRMLSRFAQLQFDHMIARKIKGVNER
IS9830      1 ........................................................................................................................
ZZZ         1 ........................................................................................................................
KP33-2      1 ........................................................................................................................
Tetron      1 ........................................................................................................................
PQ434       1 ........................................................................................................................
SRN39       1 ........................................................................................................................
Ai4         1 ......................................EN................................................................................
SQR         1 ......................................EN................................................................................
SC283       1 .......................V........D...........N..R............T...........................................................
SC35C       1 .......................V........D...........N..R............T...........................................................
BTx378      1 .......................V........D...........N..R............T...........................................................
KS115       1 ........................................................................................................................
PI586439    1 .............N.........V........D.....EN....N..R.....W......T..................................H........................
PI585749    1 .............N.........V........D.....EN....N..R.....W......T..................................H........................

TAM428    121 LVEIQKNRDMFLPPGLYPQAQAPQTNGVDSRHLAASVDEIHVVGAEIKEATDSMVEMIVGYGHQSRISVYGIVGMGGIGKTTLAQKIYNDRRIRERFHQVLIWLSISQSIAENDLLKEAI
IS9830    121 ........................................................................................................................
ZZZ       121 ........................................................................................................................
KP33-2    121 .....................................................................C......................................G...........
Tetron    121 .....................................................................C......................................G...........
PQ434     121 ........................................................................................................................
SRN39     121 ........................................................................................................................
Ai4       121 ........................................................................................................................
SQR       121 ........................................................................................................................
SC283     121 ..........................R.............................................................................................
SC35C     121 ..........................R.............................................................................................
BTx378    121 ..........................R.............................................................................................
KS115     121 ........................................................................................................................
PI586439  121 ..........................R.............................................L..........................A......PH............
PI585749  121 ..........................R.............................................L..........................A......PH............

TAM428    241 EKAGGQCNQHKSKDQLVQILLHSISGKSVFLVLDNVTNPDVWIDLLRSPMERCLDAHVLVTTRSGHVLSQMNAVHVKEMHRLKDADGLELLMKRSFRTKDEVNVFSDIGAKIVKKCDGLP
IS9830    241 ........................................................................................................................
ZZZ       241 ........................................................................................................................
KP33-2    241 ......................................................................V.................................................
Tetron    241 ......................................................................V.................................................
PQ434     241 .................................................................R......................................................
SRN39     241 ........................................................................................................................
Ai4       241 ...........................CLSCAG*RYQP*C.DRSSPL.DG.VFGCSCTCYHKERSRIVTDECGACQGNAQTKGC*WPRTAYEEIFQNQRRSKCIQ*YWSKNC*EM*GPSS
SQR       241 ...........................CLSCAG*RYQP*C.DRSSPL.DG.VFGCSCTCYHKERSRIVTDECGACQGNAQTKGC*WPRTAYEEIFQNQRRSKCIQ*YWSKNC*EM*GPSS
SC283     241 ..................................................................................................E.....................
SC35C     241 ..................................................................................................E.....................
BTx378    241 ..................................................................................................E.....................
KS115     241 ........................................................................................................................
PI586439  241 .......................V.......................................IDG................................E.................E...
PI585749  241 .......................V.......................................IDG................................E.................E...
```

```
TAM428    361 LAIKVIGGVLSSRSSKEEWERILERRWSIDGLPEELEGALYLSYSDLHPQLKQCFLCCALLPQNFDIHRDVTYWWIAEGFVKEEGSGPIHNIAEDYYHELIKRNLLQARPEYVDKGVSTM
IS9830    361 ........................................................................................................................
ZZZ       361 ........................................................................................................................
KP33-2    361 ........................................................................................................................
Tetron    361 ........................................................................................................................
PQ434     361 ........................................................................................................................
SRN39     361 ........................................................................................................................
Ai4       355 CHQGHWRRPIIQVEQRRMG.NTG.KMVY*WASRRTRRCFVLKLQ*LTPTTQTVLP.L.PVASEFRYSP.CHILVDC*RFCEGRGQRTDT*HCRRLLP*ADQEESATGKARVCRQGSIDNA
SQR       355 CHQGHWRRPIIQVEQRRMG.NTG.KMVY*WASRRTRRCFVLKLQ*LTPTTQTVLP.L.PVASEFRYSP.CHILVDC*RFCEGRGQRTDT*HCRRLLP*ADQEESATGKARVCRQGSIDNA
SC283     361 ........................................................W......................L....T......V........................I...
SC35C     361 ........................................................W......................L....T......V........................I...
BTx378    361 ........................................................W......................L....T......V........................I...
KS115     361 ....................................................................................................................I...
PI586439  361 .................................K..........C...........W...................S..L....T......V........................I...
PI585749  361 .................................K..........C...........W...................S..L....T......V........................I...

TAM428    481 HDLLRQLGQFLKRNEAIFMNEKRERCL*SIRRLGVGSAVDEIPSIEEKKRLRCLIVLHHDTCRSVKRDIFRKLVHLRILVLRGAGLESIPASVGYLALLRLLDLSYNEIKELPGSIGNLT
IS9830    481 ...........................*............................................................................................
ZZZ       481 ...........................*............................................................................................
KP33-2    481 ...........................*............................................................................................
Tetron    481 ...........................*............................................................................................
PQ434     481 ...........T...........D...*............................................................................................
SRN39     481 ...........................*............................................................................................
Ai4       470 *PVEATWPISD.K*SHLHER.A*SLP.KYSPTRCRERC*RNTFYRR.EAPTVPHCLASRHMQIGEEGHLQKVGASSHLSSTWSRP*.HTCIGGLLG.AEAAGSQLQ*DQGASRVHRKPYQ
SQR       470 *PVEATWPISD.K*SHLHER.A*SLP.KYSPTRCRERC*RNTFYRR.EAPTVPHCLASRHMQIGEEGHLQKVGASSHLSSTWSRP*.HTCIGGLLG.AEAAGSQLQ*DQGASRVHRKPYQ
SC283     481 ...........T....V......D..PS.............................D..............................................................
SC35C     481 ...........T....V......D..PS.............................D..............................................................
BTx378    481 ...........T....V......D..PS.............................D..............................................................
KS115     481 ...........T....V......D..PS.............................D..............................................................
PI586439  481 ...........T....V......D..PS.............................D.....L...................................W....................
PI585749  481 ...........T....V......D..PS.............................D.....L...................................W....................

TAM428    600 SLGCLSVFGCTKLAALPTSLMRLTTISFLKIGNTGLAQVPKGIENFKQMDNLRSVFQNGTDGFRLDELRALSMIRRLWVIRLETAIPPTEPILCDKGYLKELGLRCTMGKEANCRTHYPD
IS9830    600 ........................................................................................................................
ZZZ       600 ........................................................................................................................
KP33-2    600 .....................................................................................................................H..
Tetron    600 .....................................................................................................................H..
PQ434     600 ........................................................................................................................
SRN39     600 ........................................................................................................................
Ai4       584 PWLPF..WLHKVGSFADK.DEADHNKLPQNRKHRTG.GSERY*.FQADG*P*I.FPKQY*WFQIR*TEGT.YDTTP.GYPAGDSDTAN*AHTVRQGLPERARPALHHG*GSQLSNSLSGQ
SQR       584 PWLPF..WLHKVGSFADK.DEADHNKLPQNRKHRTG.GSERY*.FQADG*P*I.FPKQY*WFQIR*TEGT.YDTTP.GYPAGDSDTAN*AHTVRQGLPERARPALHHG*GSQLSNSLSGQ
SC283     601 .................Q...........Q................R.I....................................T.....V............................
SC35C     601 .................Q...........Q................R.I....................................T.....V............................
BTx378    601 .................Q...........Q................R.I....................................T.....V............................
KS115     601 .................Q...........Q................R.I....................................T.....V............................
PI586439  601 .................Q...........Q................R.I.........S...................F.W....T....SV............................
PI585749  601 .................Q...........Q................R.I.........S...................F.W....T....SV............................
```

```
TAM428    720 SKVKRIEEIYESFCPPPSLSYVFIDGFPGCMFPTWLSSEPQNKLPNLAHMHFNDCISCPKLPPAGQLPFLQVLHVKGADAVVNIGAELLGNSIPSGTHTTAFPKLELLEILDMYNWQNWS
IS9830    720 ........................................................................................................................
ZZZ       720 ........................................................................................................................
KP33-2    720 ...R.............G...........................S..........................................................................
Tetron    720 ...R.............G...........................S..........................................................................
PQ434     720 ........................................................................................................................
SRN39     720 ........................................................................................................................
Ai4       697 QGEED*GDLR.FLPTAKPKLRLH*WVPWLHVSNLAIFRTAE*TAKPGSYALQRLH.LPEAS.SRPATVSAGSSRQRS*RSSEHRC*APRKQHPIRN.YHCFSKARAA*DPRHVQLAELVT
SQR       697 QGEED*GDLR.FLPTAKPKLRLH*WVPWLHVSNLAIFRTAE*TAKPGSYALQRLH.LPEAS.SRPATVSAGSSRQRS*RSSEHRC*APRKQHPIRN.YHCFSKARAA*DPRHVQLAELVT
SC283     721 .............................R.......L..............Y......................................G............................
SC35C     721 .............................R.......L..............Y......................................G............................
BTx378    721 .............................R.......L..............Y......................................G............................
KS115     721 ........V....................R.......L..............Y......................................G............................
PI586439  721 .............................R.......L..............Y......................................G............................
PI585749  721 .............................R.......L..............Y......................................G............................

TAM428    840 LSMDTLFEKTQQQSLMPCLTRLRLINCPKLRALPDHLHRVVNLQRIQIEGADSLQEIVNHPGVVWLKVKNNKSLRNISNLPKLRLLLAQDCQELQQAENLSSLKALYVVDCPMEQILWKC
IS9830    840 ........................................................................................................................
ZZZ       840 ........................................................................................................................
KP33-2    840 ........................................................................................................................
Tetron    840 ........................................................................................................................
PQ434     840 .............................................................R..........................................................
SRN39     840 ........................................................................................................................
Ai4       811 KHGYLVR.N.TAIPYAMPY.AAADKLPQVESSP*SSSQSCQSTKDPNRRS*QPAGDCQPSR.C.AQG*EQQVLEEY.QPS*AVP.ACTRLPRIAAGR.P*LTQGLVRCRLPYGADT.EVF
SQR       811 KHGYLVR.N.TAIPYAMPY.AAADKLPQVESSP*SSSQSCQSTKDPNRRS*QPAGDCQPSR.C.AQG*EQQVLEEY.QPS*AVP.ACTRLPRIAAGR.P*LTQGLVRCRLPYGADT.EVF
SC283     841 ........N....F..........................................V...............................................................
SC35C     841 ........N....F..........................................V...............................................................
BTx378    841 ........N....F..........................................V...............................................................
KS115     841 ........N....F..........................................V..........A....................................................
PI586439  841 .......DN....LF...............................H.........V.D........................C.......H............................
PI585749  841 .......DN....LF...............................H.........V.D........................C.......H............................

TAM428    960 FPIEQQSTIVRVVTTGAHGQDIYPLESVFH*
IS9830    960 ..............................*
ZZZ       960 ..............................*
KP33-2    960 ..............................*
Tetron    960 ..............................*
PQ434     960 ..............................*
SRN39     960 ..............................*
Ai4       926 PHRTTEHDCPCCHHW.PWSGYLSS*I.ISLX
SQR       926 PHRTTEHDCPCCHHW.PWSGYLSS*I.ISLX
SC283     961 ..............................*
SC35C     961 ..............................*
BTx378    961 ..............................*
KS115     961 ..............................*
PI586439  961 ................T.............*
PI585749  961 ................T.............*
```

Supplemental Figure S4. Amino acid sequence alignment of ARG1 protein from different resistant and susceptible genotypes. Dots represent amino acids identical to the susceptible TAM428. The sequence changes that alter the ORF and introduce stop codon in susceptible genotypes are shaded in yellow. BoxShade (https://embnet.vital-it.ch/software/BOX_form.html) was used to perform the multiple protein sequence alignment and visualization.

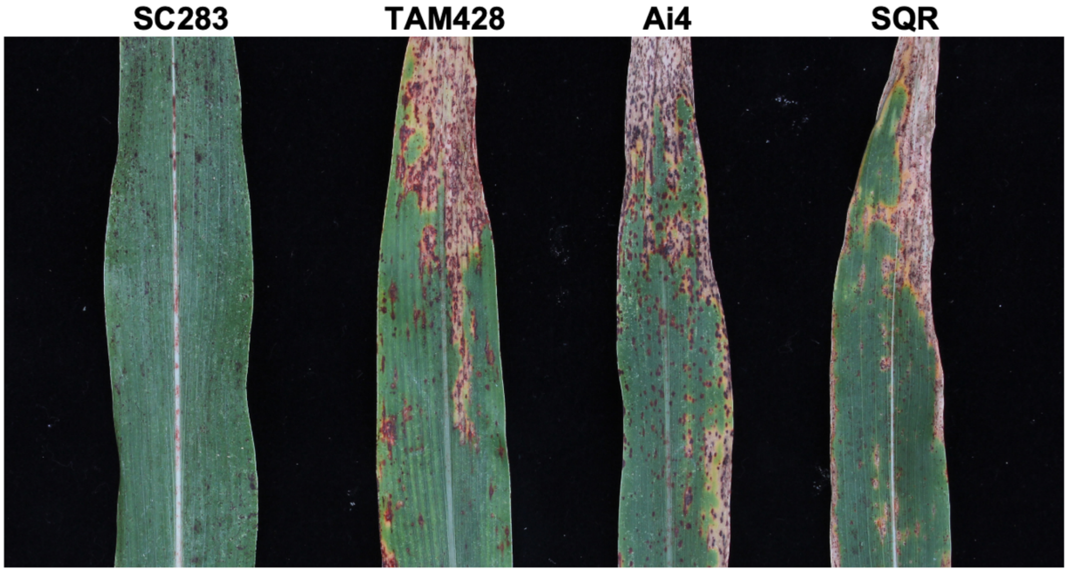

Supplemental Figure S5. Disease responses of sorghum genotypes carrying different *ARG1* alleles.

Five-week-old plants were spray inoculated with spores of *Colletotrichum sublineola* strain Csgl2 (10^6 spores/mL). Leaves were photographed 10 days post inoculation.

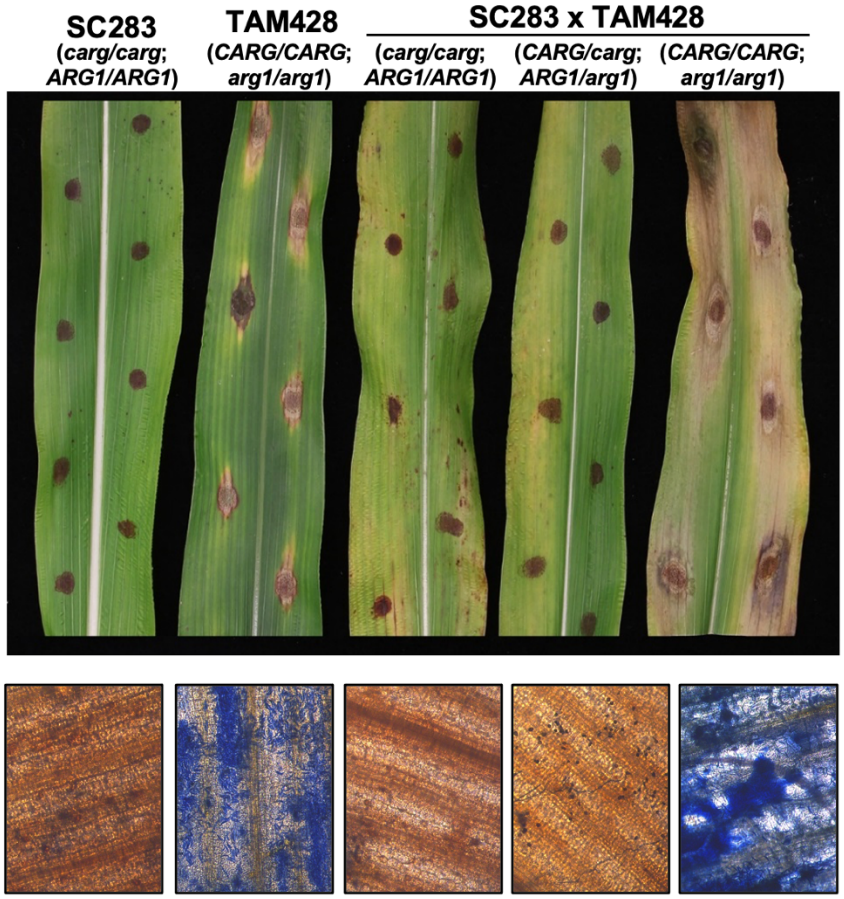

Supplemental Figure S6. Disease responses of F2 plants after drop inoculation with *Colletotrichum sublineola* spores.

Disease response on detached leaves of SC283, TAM428 and F2 plants after drop inoculation (Top row). Leaves from 4-week-old plants were inoculated with *Colletotrichum sublineola* strain Csgl2 (20 μL of 10^6 spores/mL). Leaves were photographed 12 days post inoculation. Bottom row shows trypan blue staining of infected tissues to reveal fungal growth.

```
SC283     ATGGGCTCAGTGTTGTTTAGTCTATCATCCAAATGCCTTGACAAGCTTGCTGTACTGCTGGAGGAGGA
TAM428-U  ATGGGCTCAGTGTTGTTTAGTCTATCATCCAAATGCCTTGACAAGCTTGCTGTACTGCTGGAGGAGGA
TAM428-D  ATGGGCTCAGTGTTGTTTAGTCTATCATCCAAATGCCTTGACAAGCTTGCTGTACTGCTGGAGGAGGA
          ********************************************************************

SC283     GGTTATGATGACGCTTTCTGTGCGTAAAGATATCAGGAAGCTCCATGACTACCTCAAGTACTTCAATT
TAM428-U  GATTATGATGACGCTATCTGTGCGTAAAGAGATCAGGAAGCTCCATGACTACCTCAAGTACTTTGATT
TAM428-D  GATTATGATGACGCTATCTGTGCGTAAAGAGATCAGGAAGCTCCATGACTACCTCAAGTACTTTGATT
          *.************* ************** ********************************..***

SC283     CCATTCGTGAGGATGCTGATGCTCGGGCAATGGAACACAGAGAGACGACAGGAATATGGTGGGGTGAC
TAM428-U  CCATTCATGAGGATGCTGATGCTCGGGCAATGGAACACAGAGAGATGACAGGAATATGGTGGGGTGAC
TAM428-D  CCATTCATGAGGATGCTGATGCTCGGGCAATGGAACACAGAGAGATGACAGGAATATGGTGGGGTGAC
          ******.**************************************.**********************

SC283     GTGAAAGATGTCATGTATGATGTTGATGACATCATAGATCTTTTGAGGGCTCACTCTCAGAAGCAACG
TAM428-U  GTGAAAGATGTCATGTATGATGTTGATGACATCATAGATCTTTTGAGGGCTCACTCTCAGAAGCAACG
TAM428-D  GTGAAAGATGTCATGTATGATGTTGATGACATCATAGATCTTTTGAGGGCTCACTCTCAGAAGCAACG
          ********************************************************************

SC283     ATGTTGTGATTTACGCATGCTCTCTAGGTTTGCACAACTTCAATTTGACCACATGATTGCCAGAAAAA
TAM428-U  ATGTTGTGATTTACGCATGCTCTCTAGGTTTGCACAACTTCAATTTGACCACATGATTGCCAGAAAAA
TAM428-D  ATGTTGTGATTTACGCATGCTCTCTAGGTTTGCACAACTTCAATTTGACCACATGATTGCCAGAAAAA
          ********************************************************************

SC283     TCAAGGGTGTAAACGAAAGGCTTGTAGAAATCCAAAAGAACAGAGATATGTTCCTTCCTCCGGGGTTG
TAM428-U  TCAAGGGTGTAAACGAAAGGCTTGTAGAAATCCAAAAGAACAGAGATATGTTCCTTCCTCCGGGGTTG
TAM428-D  TCAAGGGTGTAAACGAAAGGCTTGTAGAAATCCAAAAGAACAGAGATATGTTCCTTCCTCCGGGGTTG
          ********************************************************************

SC283     TATCCTCAGGCTCAGGCCCCTCAAACAAACAGAGTTGATAGCAGGCATCTGGCTGCTTCGGTTGATGA
TAM428-U  TATCCTCAGGCTCAGGCCCCTCAAACAAACGGAGTTGATAGCAGGCATCTGGCTGCTTCGGTTGATGA
TAM428-D  TATCCTCAGGCTCAGGCCCCTCAAACAAACGGAGTTGATAGCAGGCATCTGGCTGCTTCGGTTGATGA
          ******************************.*************************************

SC283     AATCCATGTTGTTGGAGCAGAAATCAAGGAAGCTACTGATAGTATGGTAGAAATGATCGTCGGCTATG
TAM428-U  AATCCATGTTGTTGGAGCAGAAATCAAGGAAGCTACTGATAGTATGGTAGAAATGATTGTCGGTTATG
TAM428-D  AATCCATGTTGTTGGAGCAGAAATCAAGGAAGCTACTGATAGTATGGTAGAAATGATTGTCGGTTATG
          *********************************************************.*****.****

SC283     GCCATCAGAGCAGGATATCAGTTTATGGGATCGTTGGGATGGGAGGAATTGGCAAGACAACTTTAGCT
TAM428-U  GCCATCAGAGCAGGATATCAGTTTATGGGATCGTTGGGATGGGAGGAATTGGTAAGACAACTTTAGCT
TAM428-D  GCCATCAGAGCAGGATATCAGTTTATGGGATCGTTGGGATGGGAGGAATTGGTAAGACAACTTTAGCT
          ****************************************************.***************

SC283     CAGAAGATATACAATGATCGTAGGATAAGAGAGAGATTTCACCAGGTCCTCATCTGGCTGTCCATTTC
TAM428-U  CAGAAGATATACAATGATCGTAGGATAAGAGAGAGATTTCACCAGGTCCTCATCTGGCTGTCCATTTC
TAM428-D  CAGAAGATATACAATGATCGTAGGATAAGAGAGAGATTTCACCAGGTCCTCATCTGGCTGTCCATTTC
          ********************************************************************

SC283     ACAGAGCATTGCCGAGAATGATCTACTCAAGGAGGCCATCGAAAAGGCAGGAGGGCAGTGCAATCAGC
TAM428-U  ACAGAGCATTGCCGAGAATGATCTACTCAAGGAGGCCATCGAAAAGGCAGGAGGGCAGTGCAATCAGC
TAM428-D  ACAGAGCATTGCCGAGAATGATCTACTCAAGGAGGCCATCGAAAAGGCAGGAGGGCAGTGCAATCAGC
          ********************************************************************

SC283     ACAAGAGTAAGGATCAGCTCGTGCAAATTCTGCTACATTCCATCAGTGGGAAGAGTGTCTTTCTTGTG
TAM428-U  ACAAGAGCAAGGATCAGCTCGTGCAAATTCTGCTACATTCCATCAGTGGGAAGAGTGTCTTTCTTGTG
TAM428-D  ACAAGAGCAAGGATCAGCTCGTGCAAATTCTGCTACATTCCATCAGTGGGAAGAGTGTCTTTCTTGTG
          *******.************************************************************
```

```
SC283     CTGGATAACGTTACCAACCCTGATGTGTGGATCGATCTTCTCCGCTCTCCGATGGAGAGGTGTTTGGA
TAM428-U  CTGGATAACGTTACCAACCCTGATGTGTGGATCGATCTTCTCCGCTCTCCGATGGAGAGGTGTTTGGA
TAM428-D  CTGGATAACGTTACCAACCCTGATGTGTGGATCGATCTTCTCCGCTCTCCGATGGAGAGGTGTTTGGA
          ********************************************************************

SC283     TGCTCATGTACTTGTTACCACAAGGAGCGGTCACGTATTGTCACAGATGAATGCGGTTCATGTCAAGG
TAM428-U  TGCTCATGTACTTGTTACCACAAGGAGCGGTCACGTATTGTCACAGATGAATGCGGTGCATGTCAAGG
TAM428-D  TGCTCATGTACTTGTTACCACAAGGAGCGGTCACGTATTGTCACAGATGAATGCGGTGCATGTCAAGG
          ********************************************************* **********

SC283     AAATGCATAGACTAAAGGATGCTGATGGCCTAGAACTGCTTATGAAGAGATCTTTCAGAACTGAAGAT
TAM428-U  AAATGCACAGACTAAAGGATGCTGATGGCCTAGAACTGCTTATGAAGAGATCTTTCAGAACCAAAGAC
TAM428-D  AAATGCACAGACTAAAGGATGCTGATGGCCTAGAACTGCTTATGAAGAGATCTTTCAGAACCAAAGAC
          *******.*****************************************************..****.

SC283     GAAGTAAATGTATTCAGTGATATTGGAGCAAAAATTGTTAAGAAATGTGATGGCCTTCCGCTTGCCAT
TAM428-U  GAAGTAAATGTATTCAGTGATATTGGAGCAAAAATTGTTAAGAAATGTGATGGCCTTCCTCTTGCCAT
TAM428-D  GAAGTAAATGTATTCAGTGATATTGGAGCAAAAATTGTTAAGAAATGTGATGGCCTTCCTCTTGCCAT
          ********************************************************************

SC283     CAAGGTCATTGGGGGCGTCCTATCATCCAGGTCGAGCAAAGAAGAATGGGAGAGAATACTGGAGAGAA
TAM428-U  CAAGGTCATTGGAGGCGTCCTATCATCCAGGTCGAGCAAAGAAGAATGGGAGAGAATACTGGAGAGAA
TAM428-D  CAAGGTCATTGGAGGCGTCCTATCATCCAGGTCGAGCAAAGAAGAATGGGAGAGAATACTGGAGAGAA
          ************.*******************************************************

SC283     GATGGTCTATTGATGGGCTTCCAGAAGAACTAGAAGGTGCTTTGTACTTAAGCTACAGTGACTTACAC
TAM428-U  GATGGTCTATTGATGGGCTTCCAGAAGAACTAGAAGGTGCTTTGTACTTAAGCTACAGTGACTTACAC
TAM428-D  GATGGTCTATTGATGGGCTTCCAGAAGAACTAGAAGGTGCTTTGTACTTAAGCTACAGTGACTTACAC
          ********************************************************************

SC283     CCACAACTGAAACAGTGCTTCCTCTGGTGTGCCTTGTTGCCCCAGAATTTCGATATTCACCGAGATGT
TAM428-U  CCACAACTCAAACAGTGCTTCCTCTGCTGTGCCCTGTTGCCTCAGAATTTCGATATTCACCGAGATGT
TAM428-D  CCACAACTCAAACAGTGCTTCCTCTGCTGTGCCCTGTTGCCTCAGAATTTCGATATTCACCGAGATGT
          ******** ***************** ******.*******.**************************

SC283     CACATACTGGTGGATTGCTGAAGGTCTTGTGAAGGAAGAGACCAGTGGACCAATACATAACGTCGCCG
TAM428-U  CACATACTGGTGGATTGCTGAAGGTTTTGTGAAGGAAGAGGGCAGCGGACCGATACATAACATTGCCG
TAM428-D  CACATACTGGTGGATTGCTGAAGGTTTTGTGAAGGAAGAGGGCAGCGGACCGATACATAACATTGCCG
          *************************.**************. ***.*****.*********.*.****

SC283     AAGATTACTACCATGAGCTGATCAAGAGGAATCTGCTACAGGCAAGGCCAGAGTATGTCGACAAGGGA
TAM428-U  AAGATTACTACCATGAGCTGATCAAGAGGAATCTGCTACAGGCAAGGCCAGAGTATGTCGACAAGGGA
TAM428-D  AAGATTACTACCATGAGCTGATCAAGAGGAATCTGCTACAGGCAAGGCCAGAGTATGTCGACAAGGGA
          ********************************************************************

SC283     ATATCGACAATGCATGACCTGTTAAGGCAACTTGGCCAATTTCTGACAAGGAATGAAGCCGTCTTCAT
TAM428-U  GTATCGACAATGCATGACCTGTTGAGGCAACTTGGCCAATTTCTGAAAAGAAATGAAGCCATCTTCAT
TAM428-D  GTATCGACAATGCATGACCTGTTGAGGCAACTTGGCCAATTTCTGAAAAGAAATGAAGCCATCTTCA-
          .**********************.********************** ***.****************

SC283     GAACGAGAAGCGTGATCGTTGCCCTTCTAGTATTCGCCGATTAGGTGTCGGGAGCGCTGTTGACGAAA
TAM428-U  GAACGAGAAGCGTGAACGTTGCCTTTGAAGTATTCGCCGACTAGGTGTCGGGAGCGCTGTTGACGAAA
TAM428-D  --------------------------------------------------------------------

SC283     TACCTTCTATAGAAGAGAAGAAGCGCCTACGGTGCCTCATTGTCTTGGATCACGACACATGCAGATCG
TAM428-U  TACCTTCTATAGAAGAGAAGAAGCGCCTACGGTGCCTCATTGTCTTGCATCACGACACATGCAGATCG
TAM428-D  --------------------------------------------------------------------
```

```
SC283    GTGAAGAGGGACATCTTCAGAAAGCTGGTGCATCTTCGCATCTTAGTTCTACGTGGAGCAGGCCTCGA
TAM428-U GTGAAGAGGGACATCTTCAGAAAGTTGGTGCATCTTCGCATCTTAGTTCTACGTGGAGCAGGCCTTGA
TAM428-D --------------------------------------------------------------------

SC283    GAGCATACCTGCATCGGTGGGTTACTTGGCGCTGCTGAGGCTGCTGGATCTCAGCTACAACGAGATCA
TAM428-U GAGCATACCTGCATCGGTGGGTTACTTGGCGCTGCTGAGGCTGCTGGATCTCAGCTACAATGAGATCA
TAM428-D --------------------------------------------------------------------

SC283    AGGAGCTTCCGGGGTCCATCGGAAACCTTACCAGCCTTGGTTGCCTTTCAGTGTTTGGTTGCACAAAG
TAM428-U AGGAGCTTCCAGGGTCCATCGGAAACCTTACCAGCCTTGGTTGCCTTTCAGTGTTTGGTTGCACAAAG
TAM428-D --------------------------------------------------------------------

SC283    TTGGCAGCTTTGCCACAAAGTCTGATGAGGCTGACCACAATAAGCTTCCTCCAGATAGGAAACACAGG
TAM428-U TTGGCAGCTTTGCCAACAAGTCTGATGAGGCTGACCACAATAAGCTTCCTCAAAATAGGAAACACAGG
TAM428-D --------------------------------------------------------------------

SC283    ACTGGCGCAGGTTCCGAAAGGTATTGAGAATTTCAGGCAGATAGATAACCTTAGATCAGTTTTCCAAA
TAM428-U ACTGGCGCAGGTTCCGAAAGGTATTGAGAATTTCAAGCAGATGGATAACCTTAGATCAGTTTTCCAAA
TAM428-D --------------------------------------------------------------------

SC283    ACGGTACTGATGGTTTCAGATTAGATGAACTGAGGGCACTCTCCATGATACGACGCCTCTGGGTTATC
TAM428-U ACGGTACTGATGGTTTCAGATTAGATGAACTGAGGGCACTCTCTATGATACGACGCCTCTGGGTTATC
TAM428-D --------------------------------------------------------------------

SC283    CGGCTGGAGACAGCGACACCGCCAACTGAGCCCGTATTGTGCGACAAGGGTTACCTGAAAGAGCTAGG
TAM428-U CGGCTGGAGACAGCGATACCGCCAACTGAGCCCATACTGTGCGACAAGGGTTACCTGAAAGAGCTAGG
TAM428-D --------------------------------------------------------------------

SC283    CCTGCGCTGTACCATGGGCAAGGAAGCCAATTGTCGAACTCACTACCCGGACAGTAAGGTGAAGAGGA
TAM428-U CCTGCGCTGCACCATGGGTAAGGAAGCCAATTGTCGAACTCACTATCCGGACAGCAAGGTGAAGAGGA
TAM428-D --------------------------------------------------------------------

SC283    TTGAGGAGATCTACGAGAGTTTTTGCCCACCGCCAAGCCTAAGCTACGTCTTTATTGATGGGTTCCCT
TAM428-U TTGAGGAGATCTACGAGAGTTTTTGCCCACCGCCAAGCCTAAGCTACGTCTTCATTGATGGGTTCCCT
TAM428-D --------------------------------------------------------------------

SC283    GGTCGCATGTTTCCAACTTGGCTATCTTTAGAACCGCAGAATAAACTTCCAAACCTGGCTCATATGCA
TAM428-U GGTTGCATGTTTCCAACTTGGCTATCTTCAGAACCGCAGAATAAACTGCCAAACCTGGCTCATATGCA
TAM428-D --------------------------------------------------------------------

SC283    CTTCTATGACTGCATATCCTGCCCGAAGCTTCCCCCAGCAGGCCAGCTACCGTTTCTGCAGGTTCTTC
TAM428-U CTTCAACGACTGCATATCCTGCCCGAAGCTTCCCCCAGCAGGCCAGCTACCGTTTCTGCAGGTTCTTC
TAM428-D --------------------------------------------------------------------

SC283    ACGTCAAAGGAGCTGATGCAGTGGTGAACATAGGTGCTGAGCTCCTCGGAAACGGCATCCCATCTGGA
TAM428-U ACGTCAAAGGAGCTGACGCAGTAGTGAACATAGGTGCTGAGCTCCTCGGAAACAGCATCCCATCCGGA
TAM428-D --------------------------------------------------------------------

SC283    ACACATACCACTGCTTTTCCAAAGCTCGAGCTGCTTGAGATCCTCGACATGTACAACTGGCAGAATTG
TAM428-U ACACATACCACTGCTTTTCCAAAGCTCGAGCTGCTTGAGATCCTCGACATGTACAACTGGCAGAATTG
TAM428-D --------------------------------------------------------------------

SC283    GTCACTAAGCATGGATACCTTGTTCGAGAACACACAACAGCAATTCCTTATGCCATGCCTTACACGCC
TAM428-U GTCACTAAGCATGGATACCTTGTTCGAGAAAACACAACAGCAATCCCTTATGCCATGCCTTACACGGC
TAM428-D --------------------------------------------------------------------
```

```
SC283     TGCGGCTGATAAATTGCCCCAAGTTGAGAGCTCTCCCTGATCATCTTCACAGAGTTGTCAATCTACAA
TAM428-U  TGCGGCTGATAAATTGCCCCAAGTTGAGAGCTCTCCCTGATCATCTTCACAGAGTTGTCAATCTACAA
TAM428-D  -----------------------------------------------------CAGAGTTGTCAATCTACAA
                                                                 *******************

SC283     AGGATCCAAATAGAAGGAGCTGACAGCCTGCAGGAGGTTGTCAACCATCCCGGGGTTGTGTGGCTCAA
TAM428-U  AGGATCCAAATAGAAGGAGCTGACAGCCTGCAGGAGATTGTCAACCATCCCGGGGTTGTGTGGCTCAA
TAM428-D  AGGATCCAAATAGAAGGAGCTGACAGCCTGCAGGAGATTGTCAACCATCCCGGGGTTGTGTGGCTCAA
          ************************************.*******************************

SC283     GGTTAAGAACAACAAGTCCTTGAGGAATATATCCAACCTTCCTAAGCTGCGCCTCTTGCTTGCACAAG
TAM428-U  GGTTAAGAACAACAAGTCCTTGAGGAATATATCCAACCTTCCTAAGCTGCGCCTCTTGCTTGCACAAG
TAM428-D  GGTTAAGAACAACAAGTCCTTGAGGAATATATCCAACCTTCCTAAGCTGCGCCTCTTGCTTGCACAAG
          ********************************************************************

SC283     ATTGCCAAGAATTGCAGCAGGCAGAGAACCTTAGCTCACTCAAGGCCTTGTACGTTGTCGATTGCCCT
TAM428-U  ATTGCCAAGAATTGCAGCAGGCAGAGAACCTTAGCTCACTCAAGGCCTTGTACGTTGTCGATTGCCCT
TAM428-D  ATTGCCAAGAATTGCAGCAGGCAGAGAACCTTAGCTCACTCAAGGCCTTGTACGTTGTCGATTGCCCT
          ********************************************************************

SC283     ATGGAGCAGATACTCTGGAAGTGTTTCCCCATAGAACAACAGAGCACGATTGTCCGTGTTGTCACCAC
TAM428-U  ATGGAGCAGATACTCTGGAAGTGTTTCCCCATAGAACAACAGAGCACGATTGTCCGTGTTGTCACCAC
TAM428-D  ATGGAGCAGATACTCTGGAAGTGTTTCCCCATAGAACAACAGAGCACGATTGTCCGTGTTGTCACCAC
          ********************************************************************

SC283     TGGGGCCCATGGTCAGGATATTTATCCTCTTGAATCAGTATTTCATTAA
TAM428-U  TGGGGCCCATGGTCAGGATATTTATCCTCTTGAATCAGTATTTCATTAA
TAM428-D  TGGGGCCCATGGTCAGGATATTTATCCTCTTGAATCAGTATTTCATTAA
          *************************************************
```

Supplemental Figure S7. Sequence alignments of *ARG1* alleles from different genotypes. The sequences of full-length and spliced *ARG1* from SC283 and TAM428 are aligned by using MAFFT (Multiple Alignment using Fast Fourier Transform, http://www.ebi.ac.uk/Tools/msa/mafft/). TAM428-U, full length *ARG1* in TAM428; TAM428-D, splice variant of *ARG1* in TAM428.

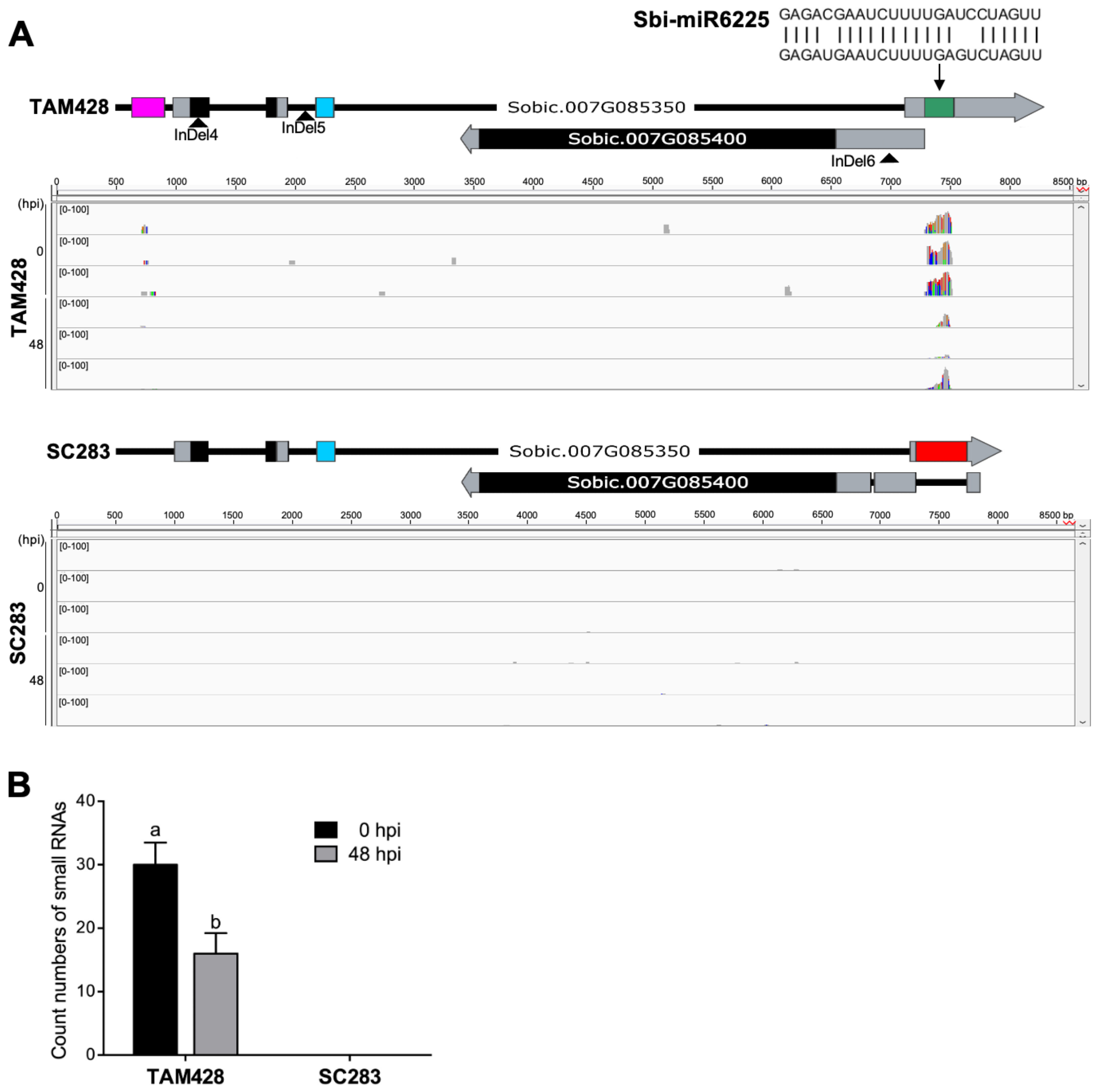

Supplemental Figure S8. The 3'-UTR of the *CARG* Natural Antisense Transcript gene produces small RNAs.

(A) Small RNA profiling identifies small RNAs derived from the *CARG* 3'-UTR in TAM428. The alignment shows the newly identified small RNA sequence and a previously described Sbi-miR6225 small RNA. **(B)** Small RNA count numbers based on small RNA-seq data. Error bars indicate the standard deviation of three libraries. Error bars ± SD (*n*=3). Letters indicate significant difference based on the Least Significant Difference (LSD, $P<0.05$).

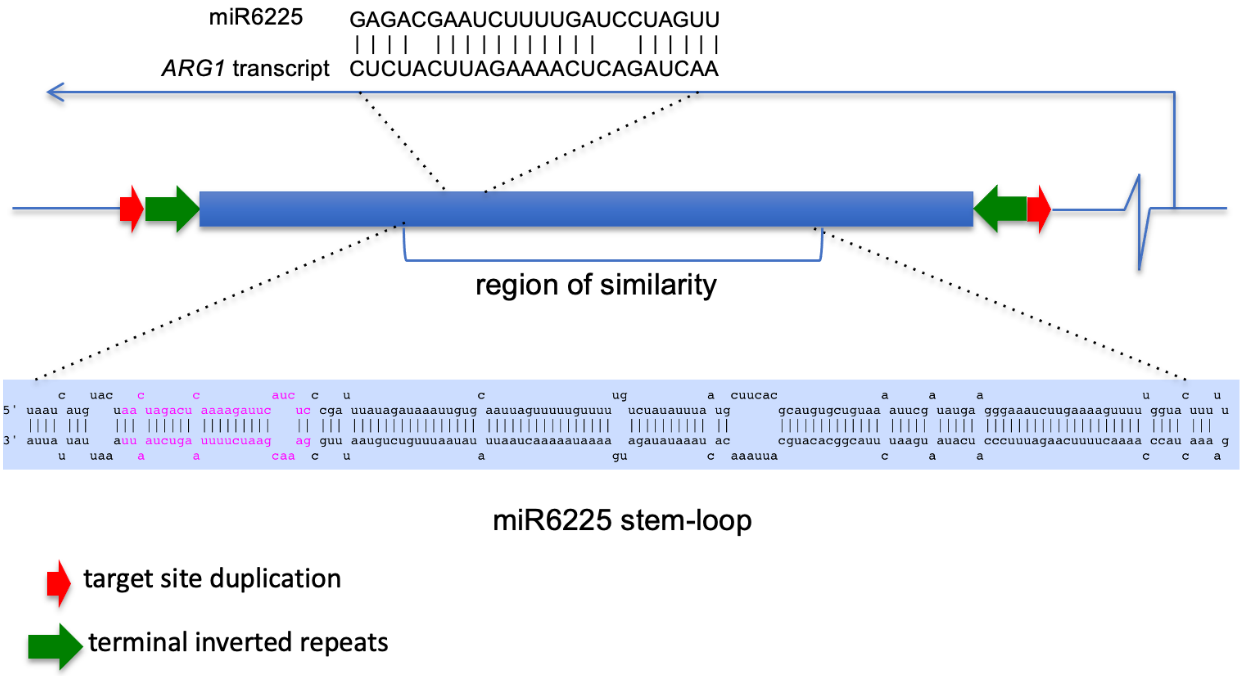

Supplemental Figure S9. Small RNAs derived from the 3'-UTR of the Natural Antisense RNA transcript gene in susceptible genotype show similarity to sorghum miR6225.

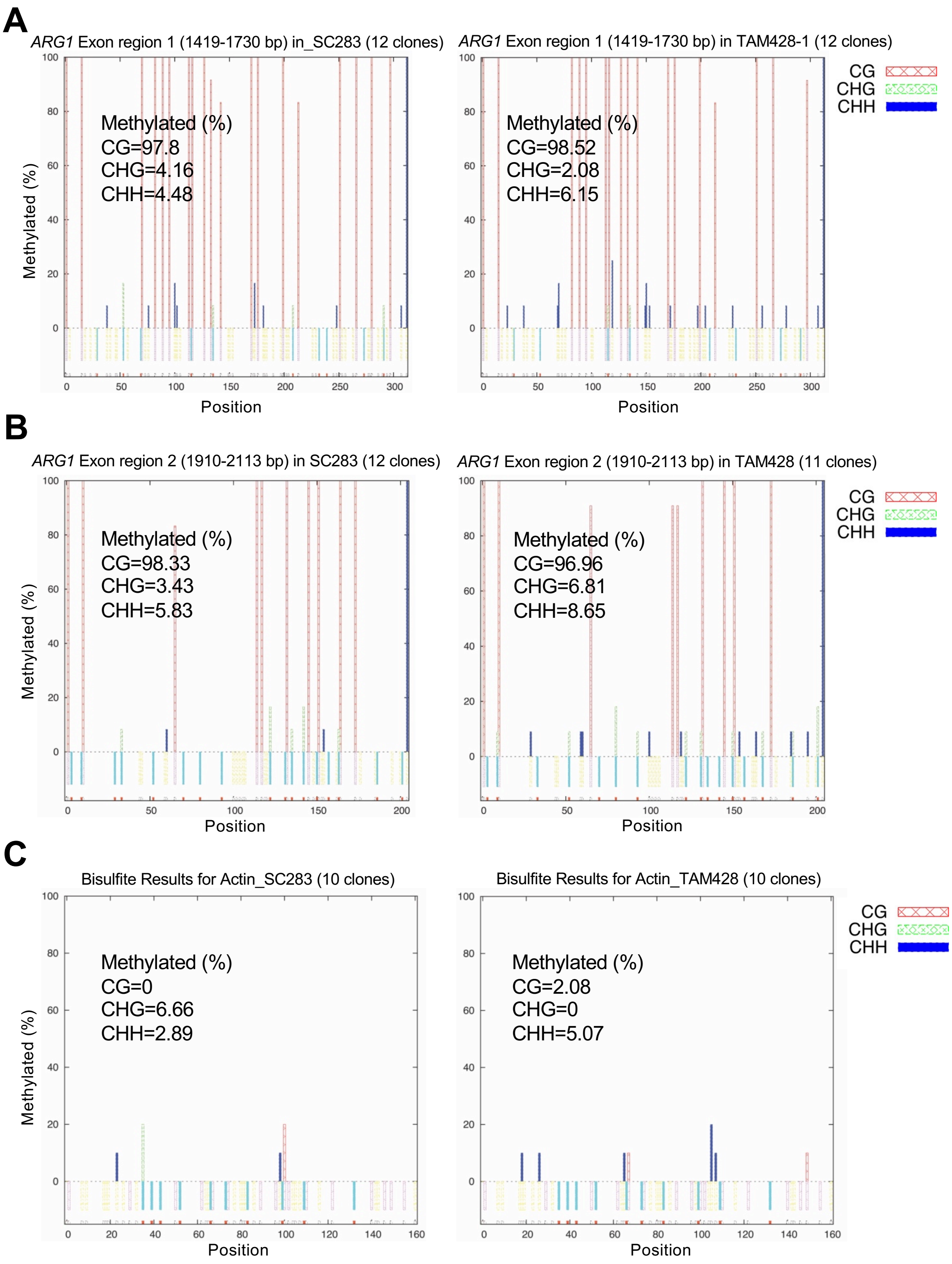

Supplemental Figure S10. DNA methylation analysis of *ARG1* exons in SC283 and TAM428. (A-C) Representative graphs for comparison of DNA methylation in two *ARG1* exon regions **(A** and **B)** and *Actin* exon **(C)** between SC283 and TAM428 using Kismath. Red, green and blue colors indicate CG, CHG and CHH methylation types, respectively, as determined by direct sequencing of individual clones. *Actin* gene was used as a positive control for bisulfite conversion.

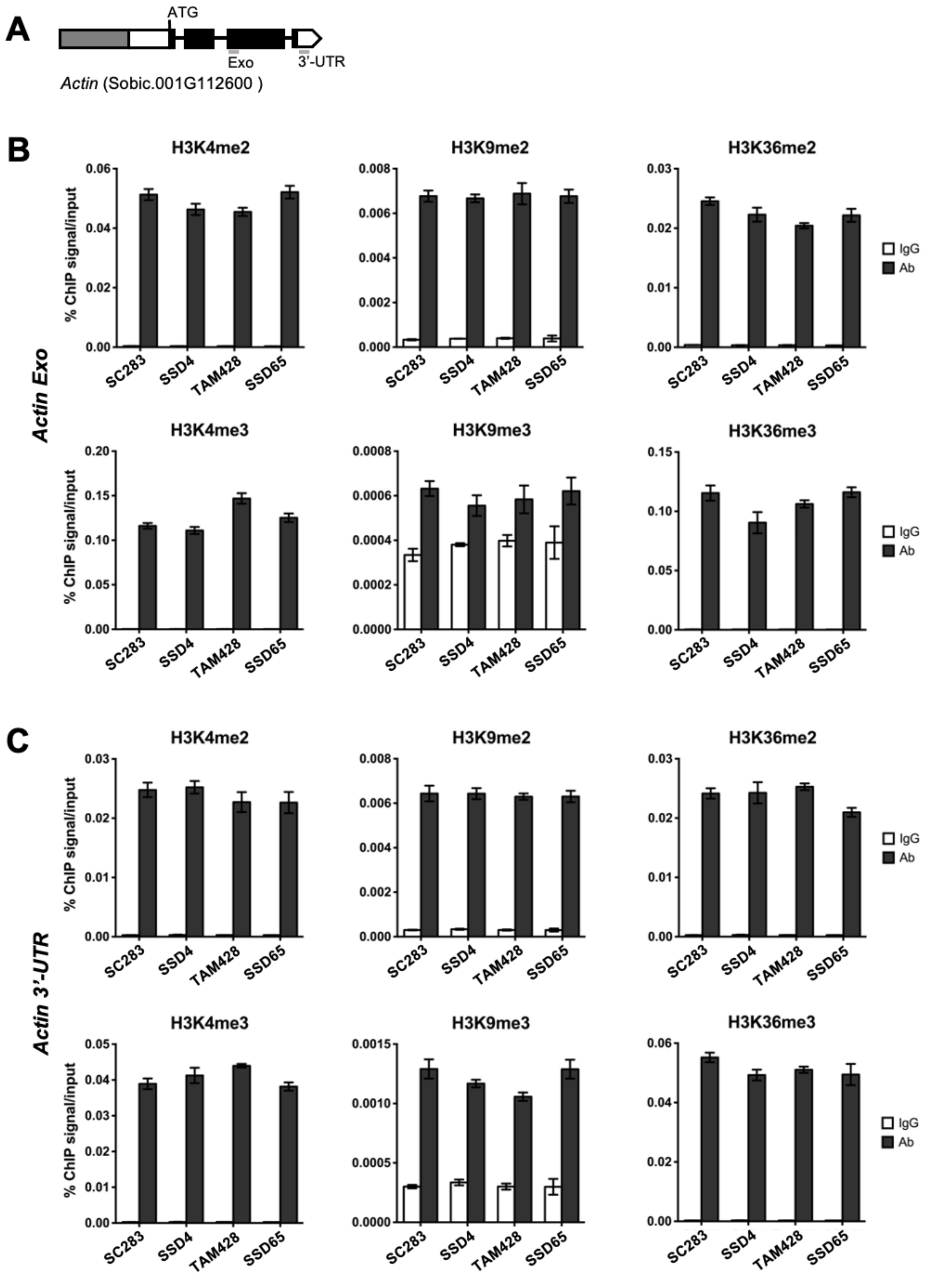
A
ATG
Exo
3'-UTR
Actin (Sobic.001G112600)
B
Actin Exo
H3K4me2
H3K9me2
H3K36me2
H3K4me3
H3K9me3
H3K36me3
% ChIP signal/input
SC283
SSD4
TAM428
SSD65
IgG
Ab
C
Actin 3'-UTR

Supplemental Figure S11. H3K4, H3K9 and H3K36 di- and tri-methylations at sorghum *Actin* gene in anthracnose resistant and susceptible lines.

(A) Schematic showing the genomic region of sorghum *Actin* gene (Sobic.001G112600). The location of primers at the coding regions (Exo) and 3'-UTR (3'-UTR) that were used to analyze the level of H3 methylations by ChIP assays are shown. The gray, white and black boxes indicate promoter, UTRs and exons, respectively. **(B)** Relative enrichment of H3K4me2/3, H3K9me2/3 and H3K36me2/3 at the exon of *Actin* gene. **(C)** Relative enrichment of H3K4me2/3, H3K9me2/3 and H3K36me2/3 at the 3'-UTR of *Actin* gene. ChIP was performed on chromatin extracts using antibodies that recognize different histone methylations as indicated, and IgG serves as a background level. Precipitated DNA was quantified by qPCR, and the DNA enrichment is shown as a percentage of IP/input. Error bars indicate the standard deviation. Similar results were obtained in two independent biological experiments. Ab, Antibody. SSD4 and SSD65 are resistant and susceptible recombinant inbred lines, respectively.

A

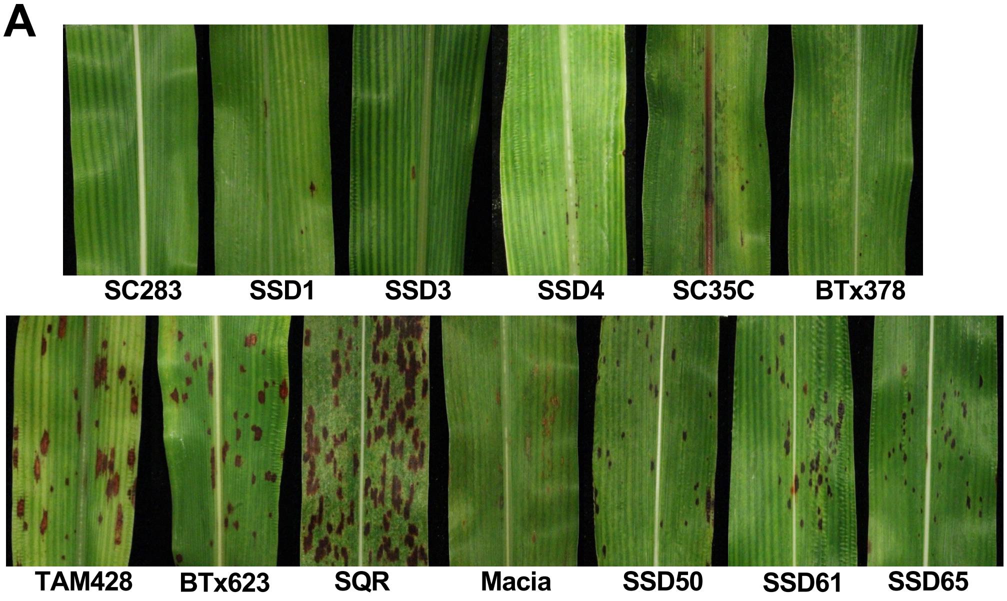

B

Genotype	Rust score	Phenotype
SC283	F^b	Resistant
SSD1	1	Resistant
SSD3	1	Resistant
SSD4	F^b	Resistant
SC35C	1	Resistant
BTx378	1	Resistant
TAM428	3	Susceptible
BTx623	2	Susceptible
SQR	2	Susceptible
Macia	2	Susceptible
SSD50	2	Susceptible
SSD61	2	Susceptible
SSD65	2	Susceptible

Supplemental Figure S12. ARG1 confers resistance to the fungal diseases target spot and rust.

(A) Target spot disease symptoms caused by *Bipolaris sorghicola* on different sorghum genotypes.

(B) Rust disease score on different sorghum genotypes. Symptoms were scored as described by White et al., 2014[48] where the infections types were as follows. 0 = complete immunity; no visible symptom; F^b = Resistant, producing hypersensitive flecks; 1 = Resistant, hypersensitive flecking in combination with small (<0.15 mm diameter) uredia with limited sporulation, surrounded by chlorosis or necrosis; 2 = Susceptible, Medium sized uredia (0.15−0.2 mm diameter) with or without chlorosis; 3 = Susceptible, Large sized uredia (>0.2 mm diameter) with or without chlorosis.

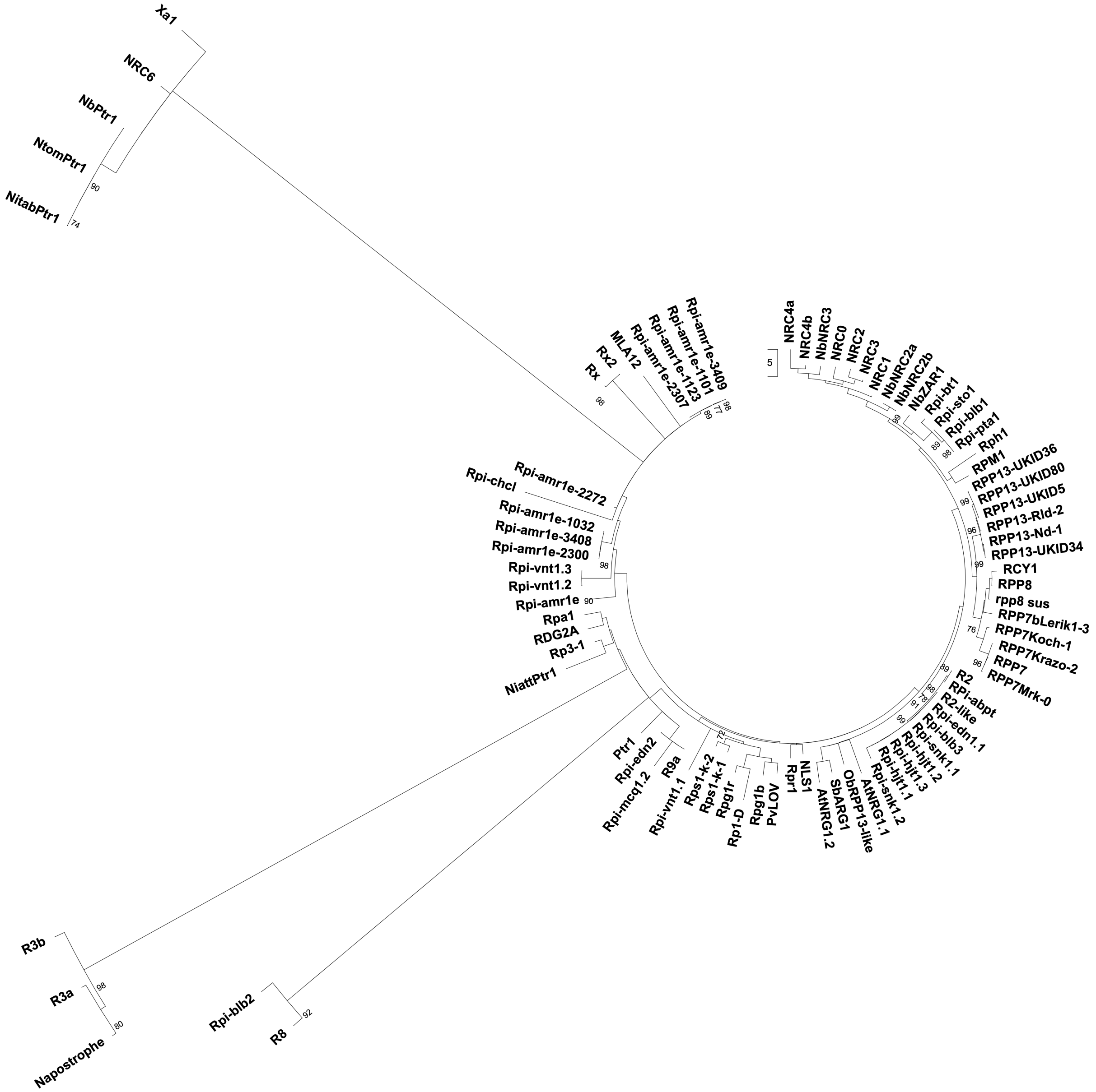

Supplemental Figure S13. Phylogenetic analysis of ARG1 with 85 other coiled-coil (CC) containing nucleotide-binding leucine-rich repeat (NLR) receptors.

The phylogenetic tree was constructed from whole amino acid sequences of the ARG1 and other CC-NRLs with MEGA X using the Maximum Likelihood method and JTT matrix-based model analysis. The sequences of 85 CC-NRLs were obtained from RefPlantNLR (http://doi.org/10.5281/zenodo.3936022) and NCBI. The tree is drawn to scale, with branch lengths measured in the number of substitutions per site. Bootstrap values are given at the node as a percentage of 1000 replicates.

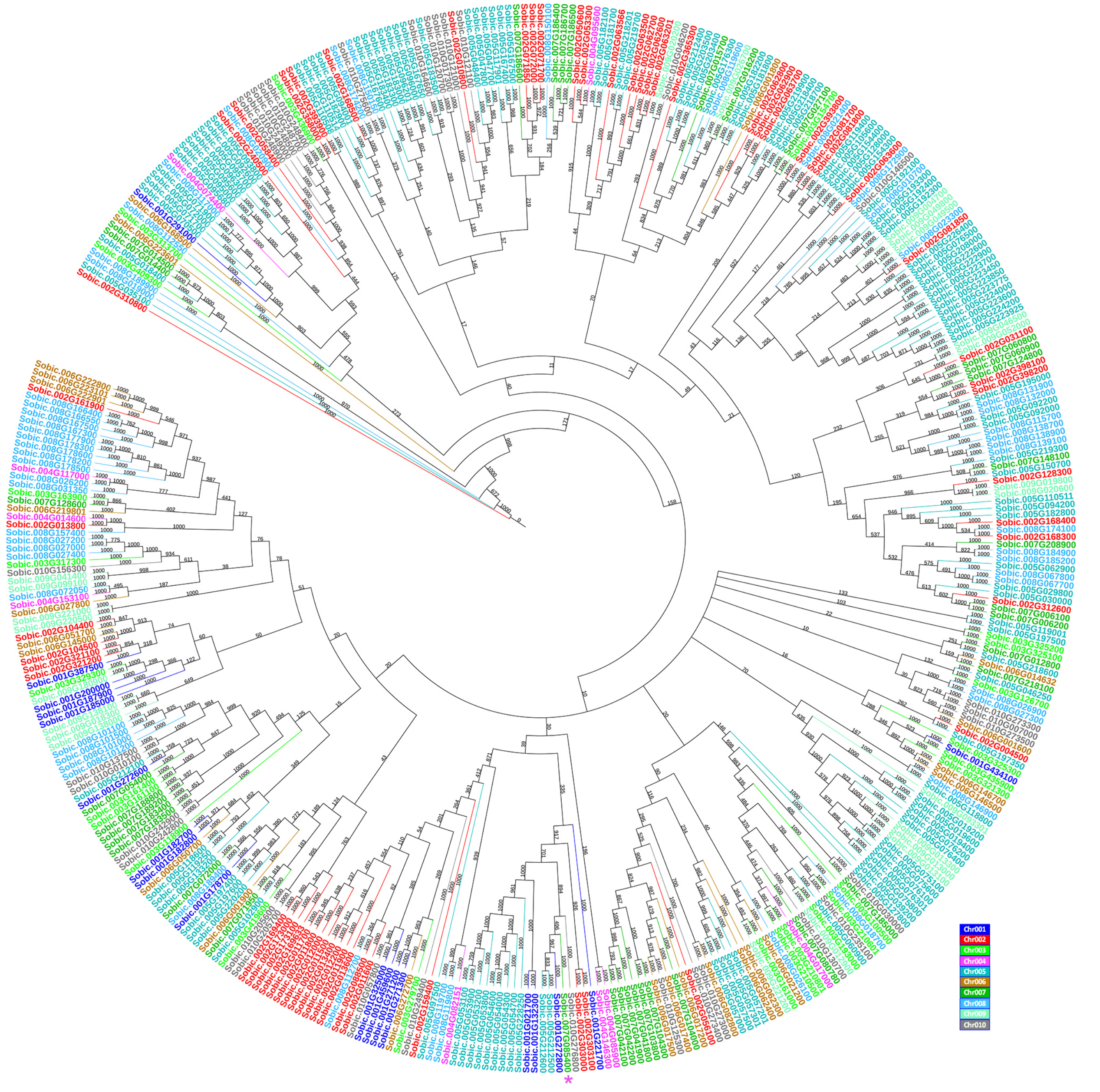

Supplemental Figure S14. Phylogenetic trees of nucleotide-binding leucine-rich repeat genes from Sorghum.

Color coded by chromosomes as indicated. The tree was constructed by the Neighbor-Joining method using bootstrap value based on 1,000 cycles of resampling with PhyIL program. Magenta asterisk indicates the ARG1 gene (Sobic.007G085400).
