# Supplementary material for: Broad-spectrum fungal resistance in sorghum is conferred through the complex regulation of an immune receptor gene embedded in a natural antisense transcript
Source: Plant Cell. 2022 Jan 9;34(5):1641–65. doi: 10.1093/plcell/koab305 (PMC9048912; doi:10.1093/plcell/koab305)
Supplement: koab305_Supplementary_Data [file koab305_supplementary_data.zip › tpc.21.00809_SupplementalFile1.pdf]

Sequence: CARG-ARG1-TAM428.dna (Linear / 8543 bp)

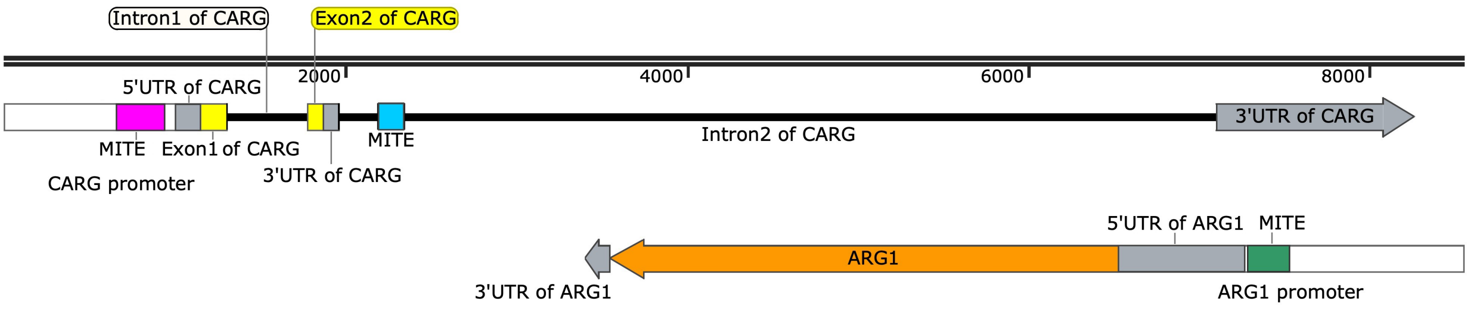

CARG-ARG1-TAM428
8543 bp

5′ ACAAGCAGGCAAACTCCCTTCTCTCCCCTTTTCCCCCCTTATCCGTCTCCAGCGCGCGGGAAAAGCCGCGCCAGAAAGTC 80
3′ TGTTCGTCCGTTTGAGGGAAGAGAGGGGAAAAGGGGGGAATAGGCAGAGGTCGCGCGCCCTTTTCGGCGCGGTCTTTCAG

CARG promoter

CGCGCGTGCGCGCTCTGCCCGCCGAGCTATTGTTTCGCGAGCCCTGGCCACCTAGCTCGCGTCCGCGCCGCCTAGGGCAC 160
GCGCGCACGCGCGAGACGGGCGGCTCGATAACAAAGCGCTCGGGACCGGTGGATCGAGCGCAGGCGCGGCGGATCCCGTG

CARG promoter

CTGGGCGCCGTCGAATCGCCGCCGAATCGGAGAAAGTAGCCGCCTAGGCGCTTCGCCCGCCTAGAGGACCGACTACCCCC 240
GACCCGCGGCAGCTTAGCGGCGGCTTAGCCTCTTTCATCGGCGGATCCGCGAAGCGGGCGGATCTCCTGGCTGATGGGGG

CARG promoter

CAAATCGTGTCGCCGGGCCGTAGACTAGCGACTAGGCGCGACTAGGCTCGTCGTAGGCGTCGATTTTCAAAACTATGAGG 320
GTTTAGCACAGCGGCCCGGCATCTGATCGCTGATCCGCGCTGATCCGAGCAGCATCCGCAGCTAAAAGTTTTGATACTCC

CARG promoter

TTGACCAAATTTATATGATAAAATAATAATTATTATGATATCAACTAAGTATCATTAGATTCTTTCTTAATTATATTTTC 400
AACTGGTTTAAATATACTATTTTATTATTAATAATACTATAGTTGATTCATAGTAATCTAAGAAAGAATTAATATAAAAG

CARG promoter

ATAATATACTCACTTTATGTTACAAATCTTAGTATTTTTCTCTATAAATTTGGTCAAACATGAAAATGCTTTGAAGGTTT 480
TATTATATGAGTGAAATACAATGTTTAGAATCATAAAAAGAGATATTTAAACCAGTTTGTACTTTTACGAAACTTCCAAA

CARG promoter

TGCGTTTTTCTTTATATTTATTTTTGCTAAATCTTTGAAAAATCATAGTAAATTACATAAAAACATAAAATATAAAAAAT 560
ACGCAAAAAGAAATATAAATAAAAACGATTTAGAAACTTTTTAGTATCATTTAATGTATTTTTGTATTTTATATTTTTTA

CARG promoter

ATAATTTTGTTAGACTTCACCATCAAAACCACTGTTAGAGATTAGAGTTGAGGGAGGAAAAAACAAATTCAGAAATGAAA 640
TATTAAAACAATCTGAAGTGGTAGTTTTGGTGACAATCTCTAATCTCAACTCCCTCCTTTTTTGTTTAAGTCTTTACTTT

CARG promoter

GACTTCTTCCTTTATTATTTATAGGGATGAAAACAGTACGGATATTTTCCGACCGTATTCGAGACCGAATTCGTTTAGAG 720
CTGAAGAAGGAAATAATAAATATCCCTACTTTTGTCATGCCTATAAAAGGCTGGCATAAGCTCTGGCTTAAGCAAATCTC

CARG promoter

MITE

GGGTTCAAATCTGTCCGTATCCGAGTCCGAATATTCAATATTCGATACCGTATCCGTATCTGAATACTTAAATCGTATAT 800
CCCAAGTTTAGACAGGCATAGGCTCAGGCTTATAAGTTATAAGCTATGGCATAGGCATAGACTTATGAATTTAGCATATA

CARG promoter

MITE

```
TTGTGATGTCGACCTCCAATCATATCTTATCCGACATAGTTGACATTATCTATATTCGAAATCGAATCCGACCAAAATAT
                                                                                 880
AACACTACAGCTGGAGGTTAGTATAGAATAGGCTGTATCAACTGTAATAGATATAAGCTTTAGCTTAGGCTGGTTTTATA
```

CARG promoter

MITE

```
GAAAACAAATATGATATTAGTGATATCCATCCGTATCCGATCCGTTTTCATCCCTAATTATTTATAATAAGGCCTAGCAG
                                                                                 960
CTTTTGTTTATACTATAATCACTATAGGTAGGCATAGGCTAGGCAAAAGTAGGGATTAATAAATATTATTCCGGATCGTC
```

CARG promoter

MITE

```
ATGAATGAAAGCCCAACAAAGCCTTGCCTCATGTGGGCCGTGACGGCTGTGTACACTCTGCTCGGCGTTCTCGCCGGACG
                                                                                1040
TACTTACTTTCGGGTTGTTTCGGAACGGAGTACACCCGGCACTGCCGACACATGTGAGACGAGCCGCAAGAGCGGCCTGC
```

CARG promoter

5'UTR of CARG

```
CTAGTCGCGGCTCGTGGAGATCAGCGCGTCGCGCCGTCGGCAAAGTTGCCAGGGCACCCGACCAGTGGAGGCGCAGAGCG
                                                                                1120
GATCAGCGCCGAGCACCTCTAGTCGCGCAGCGCGGCAGCCGTTTCAACGGTCCCGTGGGCTGGTCACCTCCGCGTCTCGC
```

CARG promoter

5'UTR of CARG

```
GGCACCAGCCAATCTAGTCTGCGCAGCTGCGCGATGGCGCCACGCGGGACCTGGCCTGCGCGGCTGCGCGTCTATGCTGC
                                                                                1200
CCGTGGTCGGTTAGATCAGACGCGTCGACGCGCTACCGCGGTGCGCCCTGGACCGGACGCGCCGACGCGCAGATACGACG
```

Exon1 of CARG

CARG promoter

5'UTR of CARG

```
CCCCCTGACAGCAAACTTTGTGGATCTACCGGACTTCAGCCCGACGAGGCGACCTGGCGACACCCCGCCGCCGCTGTCCT
                                                                                1280
GGGGGACTGTCGTTTGAAACACCTAGATGGCCTGAAGTCGGGCTGCTCCGCTGGACCGCTGTGGGGCGGCGGCGACAGGA
```

Exon1 of CARG

```
TGTCCGCCACATCTGAGCATCTCTGgtgagtttctactgagtagagagttgggagtattagagacatatgctgaaattgg
                                                                                1360
ACAGGCGGTGTAGACTCGTAGAGACcactcaaagatgactcatctctcaaccctcataatctctgtatacgactttaacc
```

Exon1 of CARG

Intron1 of CARG

```
tgattgtaatttgtgaattgctgctgaatttgtgagtagaatatatagtacagtactgtactgagttgaaagagtattcc
                                                                                1440
actaacattaaacacttaacgacgacttaaacactcatcttatatatcatgtcatgacatgactcaactttctcataagg
```

Intron1 of CARG

```
ctgcaatgtgtctagctaagaataaaagagagaaatagaatgggtgaccagctattgaattatatttagcgggtgtgat
                                                                                1520
gacgttacacagatcgattcttatttctctctttatcttacccactggtcgataacttaatataaaatcgcccacacta
```

Intron1 of CARG

```
atttttctctgttgtgaggatgacatggtttacactttatggccatgaagaagcctaataaagaaaaagctctaaacga
                                                                                1600
taaaaagagacaacactcctactgtaccaaatgtgaaaataccggtacttcttcggattatttctttttcgagatttgct
```

Intron1 of CARG

gtattgtaatcttctatttatgtaccttatcttatgtttgaacaatttttattgtcatttatgcaagttttgaatttaga 1680
cataacattagaagataaatacatggaatagaatacaaacttgttaaaaataacagtaaatacgttcaaaacttaaatct
Intron1 of CARG

gctctttttagacactttatgttttcttttactgaactatttaaataatttttgtattgaagtatactgtattagataat 1760
cgagaaaaatctgtgaaatacaaaagaaaatgacttgataaatttattaaaaacataacttcatatgacataatctatta
Intron1 of CARG

agatttttttttttgttagTCATACCGTGGACTCGAATCCTGGGTCTGCTATTGTGGACAGCCGCACCACTATACTCTGC 1840
tctaaaaaaaaaaacaatcAGTATGGCACCTGAGCTTAGGACCCAGACGATAACACCTGTCGGCGTGGTGATATGAGACG
Intron1 of CARG
Exon2 of CARG

TACGGTGGTGGATCTACACCGGCGTAGGCTCTGCACTGCTGGCACCCCGCACCCCACAGTCCCACACACATGGCAGCCTC 1920
ATGCCACCACCTAGATGTGGCCGCATCCGAGACGTGACGACCGTGGGGCGTGGGGTGTCAGGGTGTGTGTACCGTCGGAG
Exon2 of CARG
3'UTR of CARG

AAAGGCCAAGGCGGAGGCTCTGAACCAGTACGGTGCAGgtacaacaatctctgaccaaactctcaacgagccataggccc 2000
TTTCCGGTTCCGCCTCCGAGACTTGGTCATGCCACGTCcatgttgttagagactggtttgagagttgctcggtatccggg
3'UTR of CARG
Intron2 of CARG

atagtcaccaaaacttccttgtgccactcgaatagataatcgatccgaggaattttgattgtgcacatgctattgcctac 2080
tatcagtggttttgaaggaacacggtgagcttatctattagctaggctccttaaaactaacacgtgtacgataacggatg
Intron2 of CARG

tacagtattttgtatcagggcattttacaacaagtatgaaatataggctaaattaggctctgcaattagtttctcccttt 2160
atgtcataaaacatagtcccgtaaaatgttgttcatactttatatccgatttaatccgagacgttaatcaaagagggaaa
Intron2 of CARG

ttttctcttgcataagtagctaggctctttaattactccctgcgctccaaaatacaagacattttggcatttctagatgc 2240
aaaagagaacgtattcatcgatccgagaaattaatgagggacgcgaggttttatgttctgtaaaaccgtaaagatctacg
Intron2 of CARG
MITE

atagttttattatgtatctagacataaggtatgtatagatgcgtagcaaaaaatatggatctagaaaagccaaaacgtc 2320
tatcaaaataatacatagatctgtattccatacatatctacgcatcgttttttatacctagatcttttcggttttgcag
Intron2 of CARG
MITE

ttataatttggaacggagtgagtagttagcaatgactgtactctttaatttgttcatctcaattttcacaaatttttgtg 2400
aatattaaaccttgcctcactcatcaatcgttactgacatgagaaattaaacaagtagagttaaaagtgtttaaaaacac
Intron2 of CARG
MITE

tgcttatatttggtcatcaaaatcttatatataaatacaaagttcagctggtggtaatgatgtcgttggggaagaaatag 2480
acgaatataaaccagtagttttagaatatatatttatgtttcaagtcgaccaccattactacagcaaccccttctttatc
Intron2 of CARG

attacccaatacccatacctaaaaatcacccaccccaatttgtccatccttgaatacccatacccaaaaaccaggcaccc 2560
taatgggttatgggtatggatttttagtgggtggggttaaacaggtaggaacttatgggtatgggtttttggtccgtggg
Intron2 of CARG

aaatttatccatcgctgaaactagtgtataatgcccaacaggcatagattacccattgggtaagcaggtgatgaagtaga 2640
tttaaataggtagcgactttgatcacatattacgggttgtccgtatctaatgggtaacccattcgtccactacttcatct
Intron2 of CARG

agagacaaattggcaaaccaactcctgcataccagcctaacatcacctgttgctgcaaccacccgccccaccatggccct 2720
tctctgtttaaccgtttggttgaggacgtatggtcggattgtagtggacaacgacgttggtgggcggggtggtaccggga
Intron2 of CARG

gtcagacacctcgctatgcgccgatgtgtatctttgttgtaacatgtttgcctagtttgtttttttttagaatttaaatt 2800
cagtctgtggagcgatacgcggctacacatagaaacaacattgtacaaacggatcaaacaaaaaaaaatcttaaatttaa
Intron2 of CARG

aggctattcaccctctctctagccatccataaatctttacacgtggtgtaggagaccatttatgttattataaaaaatta 2880
tccgataagtgggagagagatcggtaggtatttagaaatgtgcaccacatcctctggtaaatacaataatattttttaat
Intron2 of CARG

cagtgcagcctataactgtaatcgttattatctttttttcagaatatatgacaaaggggcttcaaaaaagataccacaag 2960
gtcacgtcggatattgacattagcaataatagaaaaaaagtcttatatactgtttccccgaagttttttctatggtgttc
Intron2 of CARG

cacaagaatttcaacataacatgcagataaaaaatgtatccaacaaaacataagtaatggtttcaactaaatccatatgg 3040
gtgttcttaaagttgtattgtacgtctatttttacataggttgttttgtattcattaccaaagttgatttaggtatacc
Intron2 of CARG

aggacatgtaggtgccttgagaaaaacaatggttatggatggtaaaaacattaaagtaattcataagttcatcacaactg 3120
tcctgtacatccacggaactctttttgttaccaatacctaccatttttgtaatttcattaagtattcaagtagtgttgac
Intron2 of CARG

gatagacattatagagtatttatatgggtatggcgttagaaaatgaagaaatagagggagtacatgtgcaaagatgagta 3200
ctatctgtaatatctcataaatatacccataccgcaatcttttacttctttatctccctcatgtacacgtttctactcat
Intron2 of CARG

gttttgattaattgtaggtactaaacacgaacttctaatacttacacttataagatcctgcccgtgcatctcttaaaatt 3280
caaaactaattaacatccatgatttgtgcttgaagattatgaatgtgaatattctaggacgggcacgtagagaattttaa
Intron2 of CARG

actggccagtgtgtagccacatgatgatgaactattttgtagaaacggacgtgagcacaagttccaaactaaaaaacaga 3360
tgaccggtcacacatcggtgtactactacttgataaaacatctttgcctgcactcgtgttcaaggtttgatttttttgtct
Intron2 of CARG

cacaagaatacggtaataggcctctaagagactatgtgctgaccagattaatgaaatatactgattgaagatgataggta 3440
gtgttcttatgccattatccggagattctctgatacacgactggtctaattactttatatgactaacttctactatccat

Intron2 of CARG

3'UTR of ARG1

tcctgaccaatttgcacagacaggtgttttcacaagttatgaatagaaaaaaaggaaagagacacaagaatactgtaata 3520
aggactggttaaacgtgtctgtccacaaaagtgttcaatacttatctttttttcctttctctgtgttcttatgacattat

Intron2 of CARG

3'UTR of ARG1

tttctctaagacatcttgtgctcactatagattaatgaaatactgattcaagaggataaatatcctgaccatgggcccca 3600
aaagagattctgtagaacacgagtgatatctaattactttatgactaagttctcctatttataggactggtacccggggt

990 985 980
* His Phe Val Ser Glu Leu Pro Tyr Ile Asp Gln Gly His Ala Gly

3'UTR of ARG1 ARG1

Intron2 of CARG

gtggtgacaacacggacaatcgtgctctgttgttctatggggaaacacttccagagtatctgctccatagggcaatcgac 3680
caccactgttgtgcctgttagcacgagacaacaagatacccctttgtgaaggtctcatagacgaggtatcccgttagctg

975 970 965 960 955 950
Thr Thr Val Val Arg Val Ile Thr Ser Gln Gln Glu Ile Pro Phe Cys Lys Trp Leu Ile Gln Glu Met Pro Cys Asp Val

ARG1

Intron2 of CARG

aacgtacaaggccttgagtgagctaaggttctctgcctgctgcaattcttggcaatcttgtgcaagcaagaggcgcagct 3760
ttgcatgttccggaactcactcgattccaagagacggacgacgttaagaaccgttagaacacgttcgttctccgcgtcga

945 940 935 930 925
Val Tyr Leu Ala Lys Leu Ser Ser Leu Asn Glu Ala Gln Gln Leu Glu Gln Cys Asp Gln Ala Leu Leu Leu Arg Leu Lys

ARG1

Intron2 of CARG

taggaaggttggatatattcctcaaggacttgttgttcttaaccttgagccacacaaccccgggatggttgacaatctcc 3840
atccttccaacctatataaggagttcctgaacaacaagaattggaactcggtgtgttggggccctaccaactgttagagg

920 915 910 905 900
Pro Leu Asn Ser Ile Asn Arg Leu Ser Lys Asn Asn Lys Val Lys Leu Trp Val Val Gly Pro His Asn Val Ile Glu

ARG1

Intron2 of CARG

tgcaggctgtcagctccttctatttggatcctttgtagattgacaactctgtgaagatgatcagggagagctctcaactt 3920
acgtccgacagtcgaggaagataaacctaggaaacatctaactgttgagacacttctactagtccctctcgagagttgaa

895 890 885 880 875 870
Gln Leu Ser Asp Ala Gly Glu Ile Gln Ile Arg Gln Leu Asn Val Val Arg His Leu His Asp Pro Leu Ala Arg Leu Lys

ARG1

Intron2 of CARG

ggggcaatttatcagccgcagccgtgtaaggcatggcataagggattgctgttgtgttttctcgaacaaggtatccatgc 4000
ccccgttaaatagtcggcgtcggcacattccgtaccgtattccctaacgacaacacaaaagagcttgttccataggtacg

865 860 855 850 845
Pro Cys Asn Ile Leu Arg Leu Arg Thr Leu Cys Pro Met Leu Ser Gln Gln Gln Thr Lys Glu Phe Leu Thr Asp Met Ser
ARG1

Intron2 of CARG

ttagtgaccaattctgccagttgtacatgtcgaggatctcaagcagctcgagctttggaaaagcagtggtatgtgttccg 4080
aatcactggttaagacggtcaacatgtacagctcctagagttcgtcgagctcgaaaccttttcgtcaccatacacaaggc

840 835 830 825 820
Leu Ser Trp Asn Gln Trp Asn Tyr Met Asp Leu Ile Glu Leu Leu Glu Leu Lys Pro Phe Ala Thr Thr His Thr Gly
ARG1

Intron2 of CARG

gatgggatgctgtttccgaggagctcagcacctatgttcactactgcgtcagctcctttgacgtgaagaacctgcagaaa 4160
ctaccctacgacaaaggctcctcgagtcgtggatacaagtgatgacgcagtcgaggaaactgcacttcttggacgtcttt

815 810 805 800 795 790
Ser Pro Ile Ser Asn Gly Leu Leu Glu Ala Gly Ile Asn Val Val Ala Asp Ala Gly Lys Val His Leu Val Gln Leu Phe
ARG1

Intron2 of CARG

cggtagctggcctgctgggggaagcttcgggcaggatatgcagtcgttgaagtgcatatgagccaggtttggcagtttat 4240
gccatcgaccggacgacccccttcgaagcccgtcctatacgtcagcaacttcacgtatactcggtccaaaccgtcaaata

785 780 775 770 765
Pro Leu Gln Gly Ala Pro Pro Leu Lys Pro Cys Ser Ile Cys Asp Asn Phe His Met His Ala Leu Asn Pro Leu Lys Asn
ARG1

Intron2 of CARG

tctgcggttctgaagatagccaagttggaaacatgcaaccagggaacccatcaatgaagacgtagcttaggcttggcggt 4320
agacgccaagacttctatcggttcaacctttgtacgttggtcccttgggtagttacttctgcatcgaatccgaaccgcca

760 755 750 745 740
Gln Pro Glu Ser Ser Leu Trp Thr Pro Phe Met Cys Gly Pro Phe Gly Asp Ile Phe Val Tyr Ser Leu Ser Pro Pro
ARG1

Intron2 of CARG

gggcaaaaactctcgtagatctcctcaatcctcttcaccttgctgtccggatagtgagttcgacaattggcttccttacc 4400
cccgtttttgagagcatctagaggagttaggagaagtggaacgacaggcctatcactcaagctgttaaccgaaggaatgg

735 730 725 720 715 710
Pro Cys Phe Ser Glu Tyr Ile Glu Glu Ile Arg Lys Val Lys Ser Asp Pro Tyr His Thr Arg Cys Asn Ala Glu Lys Gly
ARG1

Intron2 of CARG

catggtgcagcgcaggcctagctctttcaggtaacccttgtcgcacagtatgggctcagttggcggtatcgctgtctcca 4480
gtaccacgtcgcgtccggatcgagaaagtccattgggaacagcgtgtcatacccgagtcaaccgccatagcgacagaggt

705 700 695 690 685
Met Thr Cys Arg Leu Gly Leu Glu Lys Leu Tyr Gly Lys Asp Cys Leu Ile Pro Glu Thr Pro Pro Ile Ala Thr Glu Leu
ARG1

Intron2 of CARG

gccggataacccagaggcgtcgtatcatagagagtgccctcagttcatctaatctgaaaccatcagtaccgttttggaaa 4560
cggcctattgggtctccgcagcatagtatctctcacgggagtcaagtagattagactttggtagtcatggcaaaaccttt

680 675 670 665 660
Arg Ile Val Trp Leu Arg Arg Ile Met Ser Leu Ala Arg Leu Glu Asp Leu Arg Phe Gly Asp Thr Gly Asn Gln Phe
ARG1

Intron2 of CARG

actgatctaaggttatccatctgcttgaaattctcaatacctttcggaacctgcgccagtcctgtgtttcctatttgag 4640
tgactagattccaataggtagacgaactttaagagttatggaaagccttggacgcggtcaggacacaaaggataaaactc

655 650 645 640 635 630
Val Ser Arg Leu Asn Asp Met Gln Lys Phe Asn Glu Ile Gly Lys Pro Val Gln Ala Leu Gly Thr Asn Gly Ile Lys Leu
ARG1

Intron2 of CARG

gaagcttattgtggtcagcctcatcagacttgttggcaaagctgccaactttgtgcaaccaaacactgaaaggcaaccaa 4720
cttcgaataacaccagtcggagtagtctgaacaaccgtttcgacggttgaaacacgttggtttgtgactttccgttggtt

625 620 615 610 605
Phe Ser Ile Thr Thr Leu Arg Met Leu Ser Thr Pro Leu Ala Ala Leu Lys Thr Cys Gly Phe Val Ser Leu Cys Gly Leu
ARG1

Intron2 of CARG

ggctggtaaggtttccgatggaccctggaagctccttgatctcattgtagctgagatccagcagcctcagcagcgccaag 4800
ccgaccattccaaaggctacctgggaccttcgaggaactagagtaacatcgactctaggtcgtcggagtcgtcgcggttc

600 595 590 585 580
Ser Thr Leu Asn Gly Ile Ser Gly Pro Leu Glu Lys Ile Glu Asn Tyr Ser Leu Asp Leu Leu Arg Leu Leu Ala Leu
ARG1

Intron2 of CARG

taacccaccgatgcaggtatgctctcaaggcctgctccacgtagaactaagatgcgaagatgcaccaactttctgaagat 4880
attgggtggctacgtccatacgagagttccggacgaggtgcatcttgattctacgcttctacgtggttgaaagacttcta

575 570 565 560 555 550
Tyr Gly Val Ser Ala Pro Ile Ser Glu Leu Gly Ala Gly Arg Leu Val Leu Ile Arg Leu His Val Leu Lys Arg Phe Ile
ARG1

Intron2 of CARG

gtccctcttcaccgatctgcatgtgtcgtgatgcaagacaatgaggcaccgtaggcgcttcttctcttctatagaaggta 4960
cagggagaagtggctagacgtacacagcactacgttctgttactccgtggcatccgcgaagaagagaagatatcttccat

545 540 535 530 525
Asp Arg Lys Val Ser Arg Cys Thr Asp His His Leu Val Ile Leu Cys Arg Leu Arg Lys Lys Glu Glu Ile Ser Pro Ile
ARG1

Intron2 of CARG

tttcgtcaacagcgctcccgacacctagtcggcgaatacttcaaaggcaacgttcacgcttctcgttcatgaagatggct 5040
aaagcagttgtcgcgagggctgtggatcagccgcttatgaagtttccgttgcaagtgcgaagagcaagtacttctaccga

520 515 510 505 500
Glu Asp Val Ala Ser Gly Val Gly Leu Arg Arg Ile Ser * Leu Cys Arg Glu Arg Lys Glu Asn Met Phe Ile Ala
ARG1

Intron2 of CARG

```
tcatttcttttcagaaattggccaagttgcctcaacaggtcatgcattgtcgatactcccttgtcgacatactctggcct   5120
agtaaagaaaagtctttaaccggttcaacggagttgtccagtacgtaacagctatgagggaacagctgtatgagaccgga
495 490 485 480 475 470
Glu Asn Arg Lys Leu Phe Gln Gly Leu Gln Arg Leu Leu Asp His Met Thr Ser Val Gly Lys Asp Val Tyr Glu Pro Arg
< ARG1
Intron2 of CARG
```

```
tgcctgtagcagattcctcttgatcagctcatggtagtaatcttcggcaatgttatgtatcggtccgctgccctcttcct   5200
acggacatcgtctaaggagaactagtcgagtaccatcattagaagccgttacaatacatagccaggcgacgggagaagga
465 460 455 450 445
Ala Gln Leu Leu Asn Arg Lys Ile Leu Glu His Tyr Tyr Asp Glu Ala Ile Asn His Ile Pro Gly Ser Gly Glu Glu Lys
< ARG1
Intron2 of CARG
```

```
tcacaaaaccttcagcaatccaccagtatgtgacatctcggtgaatatcgaaattctgaggcaacagggcacagcagagg   5280
agtgttttggaagtcgttaggtggtcatacactgtagagccacttatagctttaagactccgttgtcccgtgtcgtctcc
440 435 430 425 420
Val Phe Gly Glu Ala Ile Trp Trp Tyr Thr Val Asp Arg His Ile Asp Phe Asn Gln Pro Leu Leu Ala Cys Cys Leu
< ARG1
Intron2 of CARG
```

```
aagcactgtttgagttgtgggtgtaagtcactgtagcttaagtacaaagcaccttctagttcttctggaagcccatcaat   5360
ttcgtgacaaactcaacacccacattcagtgacatcgaattcatgtttcgtggaagatcaagaagaccttcgggtagtta
415 410 405 400 395 390
Phe Cys Gln Lys Leu Gln Pro His Leu Asp Ser Tyr Ser Leu Tyr Leu Ala Gly Glu Leu Glu Glu Pro Leu Gly Asp Ile
< ARG1
Intron2 of CARG
```

```
agaccatcttctctccagtattctctcccattcttctttgctcgacctggatgataggacgcctccaatgaccttgatgg   5440
tctggtagaagagaggtcataagagagggtaagaagaaacgagctggacctactatcctgcggaggttactggaactacc
385 380 375 370 365
Ser Trp Arg Arg Glu Leu Ile Arg Glu Trp Glu Glu Lys Ser Ser Arg Ser Ser Leu Val Gly Gly Ile Val Lys Ile Ala
< ARG1
Intron2 of CARG
```

```
caagaggaaggccatcacatttcttaacaatttttgctccaatatcactgaatacatttacttcgtctttggttctgaaa   5520
gttctccttccggtagtgtaaagaattgttaaaaacgaggttatagtgacttatgtaaatgaagcagaaaccaagacttt
360 355 350 345 340
Leu Pro Leu Gly Asp Cys Lys Lys Val Ile Lys Ala Gly Ile Asp Ser Phe Val Asn Val Glu Asp Lys Thr Arg Phe
< ARG1
Intron2 of CARG
```

```
gatctcttcataagcagttctaggccatcagcatcctttagtctgtgcatttccttgacatgcaccgcattcatctgtga   5600
ctagagaagtattcgtcaagatccggtagtcgtaggaaatcagacacgtaaaggaactgtacgtggcgtaagtagacact
335 330 325 320 315 310
Ser Arg Lys Met Leu Leu Glu Leu Gly Asp Ala Asp Lys Leu Arg His Met Glu Lys Val His Val Ala Asn Met Gln Ser
< ARG1
Intron2 of CARG
```

caatacgtgaccgctccttgtggtaacaagtacatgagcatccaaacacctctccatcggagagcggagaagatcgatcc 5680
gttatgcactggcgaggaacaccattgttcatgtactcgtaggtttgtggagaggtagcctctcgcctcttctagctagg

305 300 295 290 285
Leu Val His Gly Ser Arg Thr Thr Val Leu Val His Ala Asp Leu Cys Arg Glu Met Pro Ser Arg Leu Leu Asp Ile Trp
ARG1

Intron2 of CARG

acacatcagggttggtaacgttatccagcacaagaaagacactcttcccactgatggaatgtagcagaatttgcacgagc 5760
tgtgtagtcccaaccattgcaataggtcgtgttctttctgtgagaagggtgactaccttacatcgtcttaaacgtgctcg

280 275 270 265 260
Val Asp Pro Asn Thr Val Asn Asp Leu Val Leu Phe Val Ser Lys Gly Ser Ile Ser His Leu Leu Ile Gln Val Leu
ARG1

Intron2 of CARG

tgatccttgctcttgtgctgattgcactgccctcctgccttttcgatggcctccttgagtagatcattctcggcaatgct 5840
actaggaacgagaacacgactaacgtgacgggaggacggaaaagctaccggaggaactcatctagtaagagccgttacga

255 250 245 240 235 230
Gln Asp Lys Ser Lys His Gln Asn Cys Gln Gly Gly Ala Lys Glu Ile Ala Glu Lys Leu Leu Asp Asn Glu Ala Ile Ser
ARG1

Intron2 of CARG

ctgtgaaatggacagccagatgaggacctggtgaaatctctctcttatcctacgatcattgtatatcttctgagctaaag 5920
gacactttacctgtcggtctactcctggaccactttagagagagaataggatgctagtaacatatagaagactcgatttc

225 220 215 210 205
Gln Ser Ile Ser Leu Trp Ile Leu Val Gln His Phe Arg Glu Arg Ile Arg Arg Asp Asn Tyr Ile Lys Gln Ala Leu Thr
ARG1

Intron2 of CARG

ttgtcttaccaattcctcccatcccaacgatcccataaactgatatcctgctctgatggccataaccgacaatcatttct 6000
aacagaatggttaaggagggtagggttgctagggtatttgactataggacgagactaccggtattggctgttagtaaaga

200 195 190 185 180
Thr Lys Gly Ile Gly Gly Met Gly Val Ile Gly Tyr Val Ser Ile Arg Ser Gln His Gly Tyr Gly Val Ile Met Glu
ARG1

Intron2 of CARG

accatactatcagtagcttccttgatttctgctccaacaacatggatttcatcaaccgaagcagccagatgcctgctatc 6080
tggtatgatagtcatcgaaggaactaaagacgaggttgttgtacctaaagtagttggcttcgtcggtctacggacgatag

175 170 165 160 155 150
Val Met Ser Asp Thr Ala Glu Lys Ile Glu Ala Gly Val Val His Ile Glu Asp Val Ser Ala Ala Leu His Arg Ser Asp
ARG1

Intron2 of CARG

aactccgtttgtttgaggggcctgagcctgaggatacaacccggaggaaggaacatatctctgttcttttggatttcta 6160
ttgaggcaaacaaactccccggactcggactcctatgttgggcctccttccttgtatagagacaagaaaacctaaagat

145 140 135 130 125
Val Gly Asn Thr Gln Pro Ala Gln Ala Gln Pro Tyr Leu Gly Pro Pro Leu Phe Met Asp Arg Asn Lys Gln Ile Glu Val
ARG1

Intron2 of CARG

c a a g c c t t t c g t t t a c a c c c t t g a t t t t t c t g g c a a t c a t g t g g t c a a a t t g a a g t t g t g c a a a c c t a g a g a g c a t g c g t 6240
g t t c g g a a a g c a a a t g t g g g a a c t a a a a a g a c c g t t a g t a c a c c a g t t t a a c t t c a a c a c g t t t g g a t c t c t c g t a c g c a

120 115 110 105 100
Leu Arg Glu Asn Val Gly Lys Ile Lys Arg Ala Ile Met His Asp Phe Gln Leu Gln Ala Phe Arg Ser Leu Met Arg
ARG1
Intron2 of CARG

a a a t c a c a a c a t c g t t g c t t c t g a g a g t g a g c c c t c a a a a g a t c t a t g a t g t c a t c a a c a t c a t a c a t g a c a t c t t t c a c 6320
t t t a g t g t t g t a g c a a c g a a g a c t c t c a c t c g g g a g t t t t c t a g a t a c t a c a g t a g t t g t a g t a t g t a c t g t a g a a a g t g

95 90 85 80 75 70
Leu Asp Cys Cys Arg Gln Lys Gln Ser His Ala Arg Leu Leu Asp Ile Ile Asp Asp Val Asp Tyr Met Val Asp Lys Val
ARG1
Intron2 of CARG

g t c a c c c c a c c a t a t t c c t g t c a t c t c t c t g t g t t c c a t t g c c c g a g c a t c a g c a t c c t c a t g a a t g g a a t c a a a g t a c t 6400
c a g t g g g g t g g t a t a a g g a c a g t a g a g a g a c a c a a g g t a a c g g g c t c g t a g t c g t a g g a g t a c t t a c c t t a g t t t c a t g a

65 60 55 50 45
Asp Gly Trp Trp Ile Gly Thr Met Glu Arg His Glu Met Ala Arg Ala Asp Ala Asp Glu His Ile Ser Asp Phe Tyr Lys
ARG1
Intron2 of CARG

t g a g g t a g t c a t g g a g c t t c c t g a t c t c t t t a c g c a c a g a t a g c g t c a t c a t a a t c t c c t c c t c c a g c a g t a c a g c a a g c 6480
a c t c c a t c a g t a c c t c g a a g g a c t a g a g a a a t g c g t g t c t a t c g c a g t a g t a t t a g a g g a g g a g g t c g t c a t g t c g t t c g

40 35 30 25 20
Leu Tyr Asp His Leu Lys Arg Ile Glu Lys Arg Val Ser Leu Thr Met Met Ile Glu Glu Glu Leu Leu Val Ala Leu
ARG1
Intron2 of CARG

t t g t c a a g g c a t t t g g a t g a t a g a c t a a a c a a c a c t g a g c c c a t g g c g c g g c a c c t c t a c t t g t c t t g g c t t c t c c g c c g 6560
a a c a g t t c c g t a a a c c t a c t a t c t g a t t t g t t g t g a c t c g g g t a c c g c g c c g t g g a g a t g a a c a g a a c c g a a g a g g c g g c

15 10 5 1
Lys Asp Leu Cys Lys Ser Ser Leu Ser Phe Leu Val Ser Gly Met
ARG1
ARG1 promoter
Intron2 of CARG
5'UTR of ARG1

c a g g t a c t a c a c a g c a a a g a a a g g g a t t a g a a a c t g c a g c c a c t a c t a c a a g c c t g g a g a t g t t a g a a t t g a a t c t t c c t 6640
g t c c a t g a t g t g t c g t t t c t t t c c c t a a t c t t t g a c g t c g g t g a t g a t g t t c g g a c c t c t a c a a t c t t a a c t t a g a a g g a

Intron2 of CARG
ARG1 promoter
5'UTR of ARG1

a c c t g a a g c a c t c c g a t t g a t g t g g t g a c t g g t g a g g a a c c g a a g t g c g g a a g c a a g g a g g c g t c a g a t c a g a t c t g t t g 6720
t g g a c t t c g t g a g g c t a a c t a c a c c a c t g a c c a c t c c t t g g c t t c a c g c c t t c g t t c c t c c g c a g t c t a g t c t a g a c a a c

Intron2 of CARG
ARG1 promoter
5'UTR of ARG1

g a a g c a t c c t t g g a a a c t c t c t c t c t c c c a c c c t c a g c a g a a t g g c t c t c t t t t g a a c g c a a c t a c t t t t c c t t g t t t a t 6800
c t t c g t a g g a a c c t t t g a g a g a g a g a g g g t g g g a g t c g t c t t a c c g a g a g a a a a c t t g c g t t g a t g a a a a g g a a c a a a t a

Intron2 of CARG

ARG1 promoter

5'UTR of ARG1

g g g t c c a c c t a a a t a t g c a a a c t a a g c g g g c g c a a g g a a a a c c g g c a a a g a g t a a a t t g t t c a t g a t t a a a c c c g t g a c t 6880
c c c a g g t g g a t t t a t a c g t t t g a t t c g c c c g c g t t c c t t t t g g c c g t t t c t c a t t t a a c a a g t a c t a a t t t g g g c a c t g a

Intron2 of CARG

ARG1 promoter

5'UTR of ARG1

g t a g a g a a g c a g c a c t g c g t a g c a a c c a g c a a c t a a a a g a g a c a t c t t g t a a g g c t c t a t c t a t t a g c t t a c a a a c t g c t 6960
c a t c t c t t c g t c g t g a c g c a t c g t t g g t c g t t g a t t t t c t c t g t a g a a c a t t c c g a g a t a g a t a a t c g a a t g t t t g a c g a

Intron2 of CARG

ARG1 promoter

5'UTR of ARG1

t a t g a c a a a c a t a a g c t g a a a t t a g a a g c t a a a c a a t a c g t t t t t c t a c a g c a t t t t c t a g c c a c t c t t c t c c c a c a a c t 7040
a t a c t g t t t g t a t t c g a c t t t a a t c t t c g a t t t g t t a t g c a a a a a g a t g t c g t a a a a g a t c g g t g a g a a g a g g g t g t t g a

Intron2 of CARG

ARG1 promoter

5'UTR of ARG1

c t c a t t t a t a t t a a a a t t g g a a t t a a c c a g c t a a t t t t t a c t g t g t t t t c c t t t t t a t t t t t g c a g G T T C T C A G G A A A G C 7120
g a g t a a a t a t a a t t t t a a c c t t a a t t g g t c g a t t a a a a a t g a c a c a a a a g g a a a a a t a a a a a c g t c C A A G A G T C C T T T C G

3'UTR of CARG

Intron2 of CARG

ARG1 promoter

5'UTR of ARG1

G T T T G T C C C T A C T A C T G C T G T A T C A A G C A A G G C T A A G G A C G T G A A A T G C T T T T A C A T T T G G A T A G T G A C T C T T G A A C A G C 7200
C A A A C A G G G A T G A T G A C G A C A T A G T T C G T T C C G A T T C C T G C A C T T T A C G A A A A T G T A A A C C T A T C A C T G A G A A C T T G T C G

ARG1 promoter

3'UTR of CARG

5'UTR of ARG1

A A A T T A A A G C G A C C T G C T A C T T T C A T T T C G G T T C A G C C T T T T T A T T T T C T C T C C C T C G T G C T G T C A G C C A G T C T C T A T T T 7280
T T T A A T T T C G C T G G A C G A T G A A A G T A A A G C C A A G T C G G A A A A A T A A A A G A G A G G G A G C A C G A C A G T C G G T C A G A G A T A A A

ARG1 promoter

3'UTR of CARG

5'UTR of ARG1

CTAAGGCCTTGTTTAGTTCACCTTGAAAACCAAAAAGTTTTCAAGATTCCCTGTCACATCGAATTTTGTGGCACATGCAT 7360
GATTCCGGAACAAATCAAGTGGAACTTTTGGTTTTTCAAAAGTTCTAAGGGACAGTGTAGCTTAAAACACCGTGTACGTA

ARG1 promoter

3'UTR of CARG

MITE

GAAATATTAAATATAGACGAAAACAAAAACTAATTACACAGTTTAGCTGTAAATCACGAGACGAATCTTTTGATCCTAGT 7440
CTTTATAATTTATATCTGCTTTTGTTTTTGATTAATGTGTCAAATCGACATTTAGTGCTCTGCTTAGAAAACTAGGATCA

ARG1 promoter

3'UTR of CARG

MITE

TAGTCCATGATTGGATAATATTTGTCACAAACAAACGAAAGTGCTACAGTATCGAAAACTTTTCACTTTTCGGAACTAAA 7520
ATCAGGTACTAACCTATTATAAACAGTGTTTGTTTGCTTTCACGATGTCATAGCTTTTGAAAAGTGAAAAGCCTTGATTT

ARG1 promoter

3'UTR of CARG

MITE

CAAGCCTTACGCAGTGCAGTCAAAAAATATTGCAATCACGCAGTGCAGTTTTCCATGACTAAGCGACAGAATAGTATGCA 7600
GTTCGGAATGCGTCACGTCAGTTTTTTATAACGTTAGTGCGTCACGTCAAAAGGTACTGATTCGCTGTCTTATCATACGT

ARG1 promoter

3'UTR of CARG

MITE

GTTTTTAATGTCGTGCACCACGGCGAAAGGGACTTTCGTCTGTGTTGATTCCGTCGAAGATAATAGATGGAGGCAACGGT 7680
CAAAAATTACAGCACGTGGTGCCGCTTTCCCTGAAAGCAGACACAACTAAGGCAGCTTCTATTATCTACCTCCGTTGCCA

ARG1 promoter

3'UTR of CARG

GCTTAAAAATCTTTATTGATTTATTTAATGATAGTCACAATTCGTTATACTATTATTTTGGATATTTATATATATGAAGT 7760
CGAATTTTTAGAAATAACTAAATAAATTACTATCAGTGTTAAGCAATATGATAATAAAACCTATAAATATATATACTTCA

ARG1 promoter

3'UTR of CARG

TTAAACGCTAAACGGCTTGATCCCATAGTTTTAATCAAGTGAGTTAAAAGTATACATTTTAATTTTGGAAGATGATAGTG 7840
AATTTGCGATTTGCCGAACTAGGGTATCAAAATTAGTTCACTCAATTTTCATATGTAAAATTAAAACCTTCTACTATCAC

ARG1 promoter

3'UTR of CARG

ATAATCATATAAAGATTGATACATTATGCATAGATAGAAAATCCTTCTATGAGTTACGTAATTTAAATCTTTTCTGCCGT 7920
TATTAGTATATTTCTAACTATGTAATACGTATCTATCTTTTAGGAAGATACTCAATGCATTAAATTTAGAAAAGACGGCA

ARG1 promoter

3'UTR of CARG

ATACTTAAATGCTATATTTATGCTACTTTTTTCTGATCTTACCATTCATGTAATAAGTGAACAATGCAAATGGATGCTCG 8000
TATGAATTTACGATATAAATACGATGAAAAAAGACTAGAATGGTAAGTACATTATTCACTTGTTACGTTTACCTACGAGC

ARG1 promoter

3'UTR of CARG

TATTTTCTAGATTTATTCTAGGATTTAACGACACTATGCTTTCAGTGTTAGACGACGTGTTCGTCGACAACGAGATGCCT 8080
ATAAAAGATCTAAATAAGATCCTAAATTGCTGTGATACGAAAGTCACAATCTGCTGCACAAGCAGCTGTTGCTCTACGGA

ARG1 promoter

3'UTR of CARG

ATGGTGACTTTGTCAATTTTGAGATTTGTCGGTCCAATTCGGTTCTTCAGAGGTTCTCATAAGGGAGGGTGTGTGTGCGT 8160
TACCACTGAAACAGTTAAAACTCTAAACAGCCAGGTTAAGCCAAGAAGTCTCCAAGAGTATTCCCTCCCACACACACGCA

ARG1 promoter

3'UTR of CARG

GCGTTTATAGGGATGAGTGTGCGCTTGTGTATGTGAGCGTCTATGTCTGTAACTGGGCCTAAAAAAACTTTGTCTTGTTC 8240
CGCAAATATCCCTACTCACACGCGAACACATACACTCGCAGATACAGACATTGACCCGGATTTTTTTGAAACAGAACAAG

ARG1 promoter

3'UTR of CARG

AATGTGCCTAAGGTTAGAGAAGAATACAAAAAGAGTTATGGTTTGTTGGTGTTTTACCACCTGTGGGCCTGTGCACTTGA 8320
TTACACGGATTCCAATCTCTTCTTATGTTTTTCTCAATACCAAACAACCACAAAATGGTGGACACCCGGACACGTGAACT

ARG1 promoter

3'UTR of CARG

TCTCTACCTTGTCTTTACAAACAGGACACATTTGAGCAAAATACCGAGTTTGTTAATTCCTTAAAATTCTACAAGTGAAA 8400
AGAGATGGAACAGAAATGTTTGTCCTGTGTAAACTCGTTTTATGGCTCAAACAATTAAGGAATTTTAAGATGTTCACTTT

ARG1 promoter

CTTCTGAGCTTGTGAACATGCACTCTAAACAAGATGCCACCAGCTTCCACCATCAATTGTCTACTTTAACCTACTGGCCA 8480
GAAGACTCGAACACTTGTACGTGAGATTTGTTCTACGGTGGTCGAAGGTGGTAGTTAACAGATGAAATTGGATGACCGGT

ARG1 promoter

AGAAAAGTATAAGGATATACAAAAGAATAAGGGTGACATCATAAATCACCATCACAAGAGGTG 3′
8543
TCTTTTCATATTCCTATATGTTTTCTTATTCCCACTGTAGTATTTAGTGGTAGTGTTCTCCAC 5′

ARG1 promoter

	Feature	Location	Size (bp)			Type
✓	CARG promoter	1 .. 1153	1153			promoter
✓	MITE	662 .. 936	275			MITE
✓	5'UTR of CARG	1006 .. 1153	148			5'UTR
✓	Exon1 of CARG	1154 .. 1305	152			exon
✓	Intron1 of CARG	1306 .. 1779	474			intron
✓	Exon2 of CARG	1780 .. 1867	88			exon
✓	3'UTR of CARG	1868 .. 1958	91			3'UTR
✓	Intron2 of CARG	1959 .. 7106	5148			intron
✓	MITE	2194 .. 2344	151			MITE
✓	3'UTR of ARG1	3401 .. 3551	151		←	3'UTR
✓	ARG1	3552 .. 6524	2973		←	CDS

/translation = MGSVLFSLSSKCLDKLAVLLEEEIMMTLSVRKEIRKLHDYLKYFDSIHEDADARAMEHREMTGIWWGDVKDVMYDVDDIIDLLRAHSQKQRCCDLRMLSRFAQLQFDHMIARKIKGVN
VEIQKNRDMFLPPGLYPQAQAPQTNGVDSRHLAASVDEIHVVGAEIKEATDSMVEMIVGYGHQSRISVYGIVGMGGIGKTTLAQKIYNDRRIRERFHQVLIWLSISQSIAENDLLKEAIE
GQCNQHKSKDQLVQILLHSISGKSVFLVLDNVTNPDVWIDLLRSPMERCLDAHVLVTTRSGHVLSQMNAVHVKEMHRLKDADGLELLMKRSFRTKDEVNVFSDIGAKIVKKCDGLPLA
GGVLSSRSSKEEWERILERRWSIDGLPEELEGALYLSYSDLHPQLKQCFLCCALLPQNFDIHRDVTYWWIAEGFVKEEGSGPIHNIAEDYYHELIKRNLLQARPEYVDKGVSTMHDLLRQ
FLKRNEAIFMNEKRERCL*SIRRLGVGSAVDEIPSIEEKKRLRCLIVLHHDTCRSVKRDIFRKLVHLRILVLRGAGLESIPASVGYLALLRLLDLSYNEIKELPGSIGNLTSLGCLSVFGCTKL
TSLMRLTTISFLKIGNTGLAQVPKGIENFKQMDNLRSVFQNGTDGFRLDELRALSMIRRLWVIRLETAIPPTEPILCDKGYLKELGLRCTMGKEANCRTHYPDSKVKRIEEIYESFCPPPSL
IDGFPGCMFPTWLSSEPQNKLPNLAHMHFNDCISCPKLPPAGQLPFLQVLHVKGADAVVNIGAELLGNSIPSGTHTTAFPKLELLEILDMYNWQNWSLSMDTLFEKTQQQSLMPCLTRL
CPKLRALPDHLHRVVNLQRIQIEGADSLQEIVNHPGVVWLKVKNNKSLRNISNLPKLRLLLAQDCQELQQAENLSSLKALYVVDCPMEQILWKCFPIEQQSTIVRVVTTGAHGQDIYPLE
H*

991 codons

	Feature	Location	Size (bp)			Type
✓	ARG1 promoter	6525 .. 8543	2019			promoter
✓	5'UTR of ARG1	6525 .. 7264	740			5'UTR
✓	3'UTR of CARG	7107 .. 8269	1163		→	3'UTR
✓	MITE	7282 .. 7529	248			MITE

Sequence: CARG-ARG1-SC283.dna (Linear / 8666 bp)

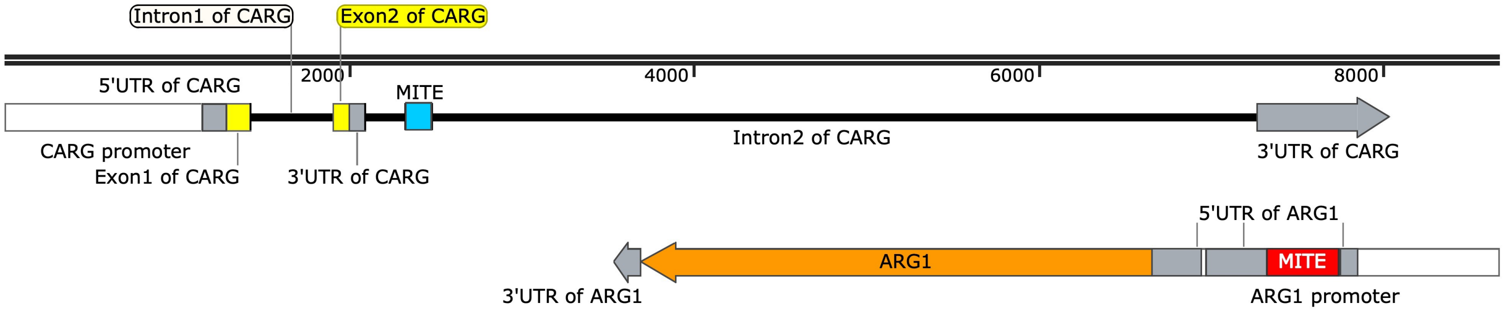

CARG-ARG1-SC283
8666 bp

5′ CCGAACGAGCCCTCAGGCGAAATATGCCCCGCGCCAAATGCTCACCCGAGAACAAAATTTGATAGTGTATACATAAAGAT 80
3′ GGCTTGCTCGGGAGTCCGCTTTATACGGGGCGCGGTTTACGAGTGGGCTCTTGTTTTAAACTATCACATATGTATTTCTA

CARG promoter

CGTTCCAATTTCTCAAAAGCAATTGGCTGAATCTTGTAAAGAACTGTTCAAATTCATCACTAAAGTAATTATTCCAATTC 160
GCAAGGTTAAAGAGTTTTCGTTAACCGACTTAGAACATTTCTTGACAAGTTTAAGTAGTGATTTCATTAATAAGGTTAAG

CARG promoter

ACCATAAGATTTTTTTCCTCCAAATATATCAACCGAAAATGTTGCAGTCACATTTGATGTCCAATCCACTTCTAGACAAG 240
TGGTATTCTAAAAAAAGGAGGTTTATATAGTTGGCTTTTACAACGTCAGTGTAAACTACAGGTTAGGTGAAGATCTGTTC

CARG promoter

AAACATGACGGCACTGCAGGCTGCAGCATAACATAGTGGAAGCTTTATTGGGCATAAGATCCAGATTGCTCAGGAGGAAA 320
TTTGTACTGCCGTGACGTCCGACGTCGTATTGTATCACCTTCGAAATAACCCGTATTCTAGGTCTAACGAGTCCTCCTTT

CARG promoter

CACATTCCCGTACAACTGTTCATCATAGTCAAGTACTCCCTCCATACTCATATTAGTTGTCATTCTGGGTGATACTTCTG 400
GTGTAAGGGCATGTTGACAAGTAGTATCAGTTCATGAGGGAGGTATGAGTATAATCAACAGTAAGACCCACTATGAAGAC

CARG promoter

TTTTCGCAAAGGATGTCGCTAGGCCACTGCATGCGGTCGGTGGACGTTTTTGCCCCTGTGGCTATAATTAATGCTTACCG 480
AAAAGCGTTTCCTACAGCGATCCGGTGACGTACGCCAGCCACCTGCAAAAACGGGGACACCGATATTAATTACGAATGGC

CARG promoter

GCGCATGCTAGGCCCGAAGCCCGGTGGCTCGTATGCAAGCACAAATCGTGGCCACTTTCACTCGCAAACAGTCGACTTCA 560
CGCGTACGATCCGGGCTTCGGGCCACCGAGCATACGTTCGTGTTTAGCACCGGTGAAAGTGAGCGTTTGTCAGCTGAAGT

CARG promoter

CTGCTAGGAGCCTTAGCGTTGCTGCAGTAGCCATCTAAACCATGATTGTTTCACATAAAGAATACACCCGCAACCATATT 640
GACGATCCTCGGAATCGCAACGACGTCATCGGTAGATTTGGTACTAACAAAGTGTATTTCTTATGTGGGCGTTGGTATAA

CARG promoter

TAATAGGGGTATACATGAAAAAATACCCTAATTAATGACTTCTTGGTAAGCACAATTATGTCCTAAATGTCAACTAATCA 720
ATTATCCCCATATGTACTTTTTTATGGGATTAATTACTGAAGAACCATTCGTGTTAATACAGGATTTACAGTTGATTAGT

CARG promoter

GGGTATGGAGGAAGTACAAGAAAGGTGGTGGATTATTCTGTGACTGATGTTTTGACTCTTCAAGATTCTTGAAATGCCTT 800
CCCATACCTCCTTCATGTTCTTTCCACCACCTAATAAGACACTGACTACAAAACTGAGAAGTTCTAAGAACTTTACGGAA

CARG promoter

ATAATTTAGGATGGAGAGACTACCATACATCGGGATTGTTACAATTTGTGGGTTTTCCCTTACAAAAATTGAAGTCAAAT 880
TATTAAATCCTACCTCTCTGATGGTATGTAGCCCTAACAATGTTAAACACCCAAAAGGGAATGTTTTTAACTTCAGTTTA

CARG promoter

```
TTAAATTTGAGAAGTTTCAACAATTAGCAAAAGAAAAAAAAATCTTGTGTCGGCTAGCAACTTCCAACTTCCAGGCCCAA
                                                                                 960
AATTTAAACTCTTCAAAGTTGTTAATCGTTTTCTTTTTTTTTAGAACACAGCCGATCGTTGAAGGTTGAAGGTCCGGGTT
```

CARG promoter

```
TGGCCGGGCAGCCAGTCCAGCCCAGAGGTAAACCCAACGCTGGTAGTCCATACAGACCCAGAACAACCTTGAAATTTGGC
                                                                                 1040
ACCGGCCCGTCGGTCAGGTCGGGTCTCCATTTGGGTTGCGACCATCAGGTATGTCTGGGTCTTGTTGGAACTTTAAACCG
```

CARG promoter

```
CCATTAAACTACTTGAACTACTTTATCCTTTAAGGCCTAGCAGATGAATGAAAGCCCAACAAAGCCTTGCCTCATGTGGG
                                                                                 1120
GGTAATTTGATGAACTTGATGAAATAGGAAATTCCGGATCGTCTACTTACTTTCGGGTTGTTTCGGAACGGAGTACACCC
```

CARG promoter

```
CCGTGACGGCTGTTGTGTAGAGCGCCTCTGCTCGGCGTTCTCGTCGAGGCTCGTGGAGATCAGCGCGTCGCGCCGTCGGC
                                                                                 1200
GGCACTGCCGACAACACATCTCGCGGAGACGAGCCGCAAGAGCAGCTCCGAGCACCTCTAGTCGCGCAGCGCGGCAGCCG
```

CARG promoter

5'UTR of CARG

```
AAAGCTGCCAGGGCACCCGAGCGCGAGCAGTGGAGGCGCAGAGGCAGAGCAGGCACCAGCCAACCTAGCCTGCGCAGCTG
                                                                                 1280
TTTCGACGGTCCCGTGGGCTCGCGCTCGTCACCTCCGCGTCTCCGTCTCGTCCGTGGTCGGTTGGATCGGACGCGTCGAC
```

CARG promoter

5'UTR of CARG

```
CGCGATGGCGCCACGCGGGACCTGGCCTGCGCGGCTGCGCGTCTATGCTGCCCCCTGACAGCAAACTTTGTGGATCTACC
                                                                                 1360
GCGCTACCGCGGTGCGCCCTGGACCGGACGCGCCGACGCGCAGATACGACGGGGGACTGTCGTTTGAAACACCTAGATGG
```

Exon1 of CARG

CARG promoter

5'UTR of CARG

```
GGACTTCAGCCCGACGAGGCGACACCTCGCCGCCGCTGTCCTTGTCCGCCACATCTGAGCATCTCTGgtgagtttctact
                                                                                 1440
CCTGAAGTCGGGCTGCTCCGCTGTGGAGCGGCGGCGACAGGAACAGGCGGTGTAGACTCGTAGAGACcactcaaagatga
```

Exon1 of CARG — Intron1 of CARG

```
gagtagagagtttggagtgttagagacatatgcttaaattggtgattgtagtttgtgaattgctgctgaatttgtgagta
                                                                                 1520
ctcatctctcaaacctcacaatctctgtatacgaatttaaccactaacatcaaacacttaacgacgacttaaacactcat
```

Intron1 of CARG

```
gaatatatagtacagtactgtgtactgagttgaaagagtattattccctgcaatgtgtctagctaagaataaaagagaga
                                                                                 1600
cttatatatcatgtcatgacacatgactcaactttctcataataagggacgttacacagatcgattcttattttctctct
```

Intron1 of CARG

```
aatagaatgggtgaccagctattgaattatatttagcgggtgtgatatttttctctgttgtgacttgtgaggatgacat
                                                                                 1680
ttatcttacccactggtcgataacttaatataaaaatcgcccacactataaaaagagacaacactgaacactcctactgta
```

Intron1 of CARG

```
ggtttacacttttatggccatgaagaagcctaaagaaaaagctctaaacgagtattgtaatcttctatttatgtacctta
                                                                                 1760
ccaaatgtgaaaataccggtacttcttcggatttctttttcgagatttgctcataacattagaagataaatacatggaat
```

Intron1 of CARG

tcttatatctgaacaattttattgtcatttatgcaagttttgaatttagagctcttttagacactttatatttcttt 1840
agaatatagacttgttaaaataacagtaaatacgttcaaaacttaaatctcgagaaaatctgtgaaatataaaagaaa
Intron1 of CARG

tactgaactatttatataattttgtattgaagtatactgtattagataatagattttttttttgttagTCATACCGTGG 1920
atgacttgataaatatattaaaacataacttcatatgacataatctattatctaaaaaaaaaacaatcAGTATGGCACC
Intron1 of CARG
Exon2 of CARG

ACTCGAATCCTGGGTCTGCTATTGTGGACAGCTGCACCGCTATACTCTGCTAGGGTGGTGGATCTACACCGGCGTAGGCT 2000
TGAGCTTAGGACCCAGACGATAACACCTGTCGACGTGGCGATATGAGACGATCCCACCACCTAGATGTGGCCGCATCCGA
Exon2 of CARG
3'UTR of CARG

CTGCCCTGCTGGCACCCCGCACCCCACACACATGGCAGCCTCAAAGGCCAAGGCGGAGGCTCTGAACCAGGTATACAACA 2080
GACGGGACGACCGTGGGGCGTGGGGTGTGTGTACCGTCGGAGTTTCCGGTTCCGCCTCCGAGACTTGGTCCATATGTTGT
3'UTR of CARG

GTACGGTACAGGTACAACAATCTCTGACCAAACTCTCGACGAGCCATAGGCCCGTAGTCACCAAAAGTGCCTTGCGCCAC 2160
CATGCCATGTCCATGTTGTTAGAGACTGGTTTGAGAGCTGCTCGGTATCCGGGCATCAGTGGTTTTCACGGAACGCGGTG
3'UTR of CARG
Intron2 of CARG

TCGAGTAGATAATTGATCTGAGGAATTTTGATTGTGCAGATGCTATTGCCTATATGGTATTTTGTATCAGGGCATTTTAC 2240
AGCTCATCTATTAACTAGACTCCTTAAAACTAACACGTCTACGATAACGGATATACCATAAAACATAGTCCCGTAAAATG
Intron2 of CARG

AACAAGTATGAAATATAGGCTAAATTAGGCTCTGCAATTAGTTTCTCCCTTTTTTTTTTCTCTTGCATAAGTAGCTAAGC 2320
TTGTTCATACTTTATATCCGATTTAATCCGAGACGTTAATCAAAGAGGGAAAAAAAAAAGAGAACGTATTCATCGATTCG
Intron2 of CARG

TCTTTAATTACTCCCTGCGCTCCAAACTACAAGACATTTTGGCATTTCTAGATGCATAGTTTTTATTATGTATCTAGACA 2400
AGAAATTAATGAGGGACGCGAGGTTTGATGTTCTGTAAAACCGTAAAGATCTACGTATCAAAAATAATACATAGATCTGT
Intron2 of CARG
MITE

TAACGTATGTATAGATGCGTAGCAAAAAATACGGATCTAGAAAAGCCAAAACGTCTTATAATTTGGAACGGAGTGAGTAG 2480
ATTGCATACATATCTACGCATCGTTTTTTATGCCTAGATCTTTTCGGTTTTGCAGAATATTAAACCTTGCCTCACTCATC
Intron2 of CARG
MITE

TTAGCAATGACTGTACTCTTTAATTTGTTCATCTCAATTTTCACAAATTTTTGTGTGCTTATATTTGGTCATCAAAATCT 2560
AATCGTTACTGACATGAGAAATTAAACAAGTAGAGTTAAAAGTGTTTAAAAACACACGAATATAAACCAGTAGTTTTAGA
Intron2 of CARG

TATATATAAATACAAAGTTCAGCTGGTGGTAATGATGTCGTTGGGGAAGAAATAGATTACCCAATACCCATACCTAAAAA 2640
ATATATATTTATGTTTCAAGTCGACCACCATTACTACAGCAACCCCTTCTTTATCTAATGGGTTATGGGTATGGATTTTT
Intron2 of CARG

TCACCCACCCCAATTTGTCCATCCTTGAATACCCATACCCAAAAACCAGGCACCCAAATTTATCCATCGCTGAAACTAGT 2720
AGTGGGTGGGGTTAAACAGGTAGGAACTTATGGGTATGGGTTTTTGGTCCGTGGGTTTAAATAGGTAGCGACTTTGATCA

Intron2 of CARG

GTATAATGCCCAACAGGCAGAGATTACCCATTGGGTAAGCAGGTGATGAAGTAGAAGAGACAAATTGGCAAACCAACTCG 2800
CATATTACGGGTTGTCCGTCTCTAATGGGTAACCCATTCGTCCACTACTTCATCTTCTCTGTTTAACCGTTTGGTTGAGC

Intron2 of CARG

TGCATACCAGCCTAACATCACCTGTTGCTTCAACCGCCCGCCCCACCATGGCCCTGTCAGACACCTCGCTATGCATCGAT 2880
ACGTATGGTCGGATTGTAGTGGACAACGAAGTTGGCGGGCGGGGTGGTACCGGGACAGTCTGTGGAGCGATACGTAGCTA

Intron2 of CARG

GTGTATCTTTGTTGTAACATGTTTGCCTAGTTTGTTTTTTTTTTAAATTTAAATTAGGCTATTCACCCTCTCTCTAGCCA 2960
CACATAGAAACAACATTGTACAAACGGATCAAACAAAAAAAAAATTTAAATTTAATCCGATAAGTGGGAGAGAGATCGGT

Intron2 of CARG

TCCATAAATCTTTACACGTGGTGTAGGAGACCATTTATGTTATCATAAAAAATTACAGTGCAGCCTATAACTGTAATCTT 3040
AGGTATTTAGAAATGTGCACCACATCCTCTGGTAAATACAATAGTATTTTTTAATGTCACGTCGGATATTGACATTAGAA

Intron2 of CARG

TATTATCTTTTTTTCAGAATATATGATAAAGGGGCTTCAAAAAAGACACCACAAGCACAAGAATTTCAACATAACATGCA 3120
ATAATAGAAAAAAAGTCTTATATACTATTTCCCCGAAGTTTTTTCTGTGGTGTTCGTGTTCTTAAAGTTGTATTGTACGT

Intron2 of CARG

GACAAAAAATGTATCCAACAAAACATAAGTTATGGTTTCAACTAAATCCATATGGAGGACATGTAGTTGCCTTGAGAAAA 3200
CTGTTTTTTACATAGGTTGTTTTGTATTCAATACCAAAGTTGATTTAGGTATACCTCCTGTACATCAACGGAACTCTTTT

Intron2 of CARG

ACATTGGTTATGGATGGTAAAAACATTAAAGTAATTCATAAGTTCATCACAACTAGATAGACATTATAGAGTATTTATAT 3280
TGTAACCAATACCTACCATTTTTGTAATTTCATTAAGTATTCAAGTAGTGTTGATCTATCTGTAATATCTCATAAATATA

Intron2 of CARG

GGGTATGGCGTTAGAAAATGAAGAAATAGAGGGTGTACATGTGCAAAGATGAGTAGTTTTGATTAATTGTAGGTACTAAA 3360
CCCATACCGCAATCTTTTACTTCTTTATCTCCCACATGTACACGTTTCTACTCATCAAAACTAATTAACATCCATGATTT

Intron2 of CARG

CACGAACTTCTAATACTTACACTTATAAGATTCTGCCCGTGCATCTCTTAAAATTACTGGCCAGTGTGTAGCCACATGAT 3440
GTGCTTGAAGATTATGAATGTGAATATTCTAAGACGGGCACGTAGAGAATTTTAATGACCGGTCACACATCGGTGTACTA

Intron2 of CARG

GATGAACTATTTTGTAGAAACGGACGTGGGCACAAGTTCCAAACTAAAAAACAGACACAAGAATACGATAATAGGCCTCT 3520
CTACTTGATAAAACATCTTTGCCTGCACCCGTGTTCAAGGTTTGATTTTTTGTCTGTGTTCTTATGCTATTATCCGGAGA

Intron2 of CARG

AAGAGACTATGTGCTGACCAGATTAATGAAATATACTGATTGAAGATGATAGGTATCCTGACCAATTTGCACAAACAGGT 3600
TTCTCTGATACACGACTGGTCTAATTACTTTATATGACTAACTTCTACTATCCATAGGACTGGTTAAACGTGTTTGTCCA
Intron2 of CARG
3'UTR of ARG1

GTTTTCACAAGTTATGAATAGAAAAAAAGGAACGAGACACAAGAATACTGTAACATTTCTCTAAGACATCTTGTGCTCAC 3680
CAAAAGTGTTCAATACTTATCTTTTTTTCCTTGCTCTGTGTTCTTATGACATTGTAAAGAGATTCTGTAGAACACGAGTG
Intron2 of CARG
3'UTR of ARG1

TATAGATTAATGAAATACTGATTCAAGAGGATAAATATCCTGACCATGGGCCCCAGTGGTGACAACACGGACAATCGTGC 3760
ATATCTAATTACTTTATGACTAAGTTCTCCTATTTATAGGACTGGTACCCGGGGTCACCACTGTTGTGCCTGTTAGCACG
990 985 980 975 970
* His Phe Val Ser Glu Leu Pro Tyr Ile Asp Gln Gly His Ala Gly Thr Thr Val Val Arg Val Ile Thr Ser
ARG1
3'UTR of ARG1
Intron2 of CARG

TCTGTTGTTCTATGGGGAAACACTTCCAGAGTATCTGCTCCATAGGGCAATCGACAACGTACAAGGCCTTGAGTGAGCTA 3840
AGACAACAAGATACCCCTTTGTGAAGGTCTCATAGACGAGGTATCCCGTTAGCTGTTGCATGTTCCGGAACTCACTCGAT
965 960 955 950 945
Gln Gln Glu Ile Pro Phe Cys Lys Trp Leu Ile Gln Glu Met Pro Cys Asp Val Val Tyr Leu Ala Lys Leu Ser Ser
ARG1
Intron2 of CARG

AGGTTCTCTGCCTGCTGCAATTCTTGGCAATCTTGTGCAAGCAAGAGGCGCAGCTTAGGAAGGTTGGATATATTCCTCAA 3920
TCCAAGAGACGGACGACGTTAAGAACCGTTAGAACACGTTCGTTCTCCGCGTCGAATCCTTCCAACCTATATAAGGAGTT
940 935 930 925 920 915
Leu Asn Glu Ala Gln Gln Leu Glu Gln Cys Asp Gln Ala Leu Leu Leu Arg Leu Lys Pro Leu Asn Ser Ile Asn Arg Leu
ARG1
Intron2 of CARG

GGACTTGTTGTTCTTAACCTTGAGCCACACAACCCCGGGATGGTTGACAACCTCCTGCAGGCTGTCAGCTCCTTCTATTT 4000
CCTGAACAACAAGAATTGGAACTCGGTGTGTTGGGGCCCTACCAACTGTTGGAGGACGTCCGACAGTCGAGGAAGATAAA
910 905 900 895 890
Ser Lys Asn Asn Lys Val Lys Leu Trp Val Val Gly Pro His Asn Val Val Glu Gln Leu Ser Asp Ala Gly Glu Ile Gln
ARG1
Intron2 of CARG

GGATCCTTTGTAGATTGACAACTCTGTGAAGATGATCAGGGAGAGCTCTCAACTTGGGGCAATTTATCAGCCGCAGGCGT 4080
CCTAGGAAACATCTAACTGTTGAGACACTTCTACTAGTCCCTCTCGAGAGTTGAACCCCGTTAAATAGTCGGCGTCCGCA
885 880 875 870 865
Ile Arg Gln Leu Asn Val Val Arg His Leu His Asp Pro Leu Ala Arg Leu Lys Pro Cys Asn Ile Leu Arg Leu Arg
ARG1
Intron2 of CARG

GTAAGGCATGGCATAAGGAATTGCTGTTGTGTGTTCTCGAACAAGGTATCCATGCTTAGTGACCAATTCTGCCAGTTGTA 4160
CATTCCGTACCGTATTCCTTAACGACAACACACAAGAGCTTGTTCCATAGGTACGAATCACTGGTTAAGACGGTCAACAT
860 855 850 845 840 835
Thr Leu Cys Pro Met Leu Phe Gln Gln Gln Thr Asn Glu Phe Leu Thr Asp Met Ser Leu Ser Trp Asn Gln Trp Asn Tyr
ARG1
Intron2 of CARG

CATGTCGAGGATCTCAAGCAGCTCGAGCTTTGGAAAAGCAGTGGTATGTGTTCCAGATGGGATGCCGTTTCCGAGGAGCT 4240
GTACAGCTCCTAGAGTTCGTCGAGCTCGAAACCTTTTCGTCACCATACACAAGGTCTACCCTACGGCAAAGGCTCCTCGA
830 825 820 815 810
Met Asp Leu Ile Glu Leu Leu Glu Leu Lys Pro Phe Ala Thr Thr His Thr Gly Ser Pro Ile Gly Asn Gly Leu Leu Glu
ARG1
Intron2 of CARG

CAGCACCTATGTTCACCACTGCATCAGCTCCTTTGACGTGAAGAACCTGCAGAAACGGTAGCTGGCCTGCTGGGGGAAGC 4320
GTCGTGGATACAAGTGGTGACGTAGTCGAGGAAACTGCACTTCTTGGACGTCTTTGCCATCGACCGGACGACCCCCTTCG
805 800 795 790 785
Ala Gly Ile Asn Val Val Ala Asp Ala Gly Lys Val His Leu Val Gln Leu Phe Pro Leu Gln Gly Ala Pro Pro Leu
ARG1
Intron2 of CARG

TTCGGGCAGGATATGCAGTCATAGAAGTGCATATGAGCCAGGTTTGGAAGTTTATTCTGCGGTTCTAAAGATAGCCAAGT 4400
AAGCCCGTCCTATACGTCAGTATCTTCACGTATACTCGGTCCAAACCTTCAAATAAGACGCCAAGATTTCTATCGGTTCA
780 775 770 765 760 755
Lys Pro Cys Ser Ile Cys Asp Tyr Phe His Met His Ala Leu Asn Pro Leu Lys Asn Gln Pro Glu Leu Ser Leu Trp Thr
ARG1
Intron2 of CARG

TGGAAACATGCGACCAGGGAACCCATCAATAAAGACGTAGCTTAGGCTTGGCGGTGGGCAAAAACTCTCGTAGATCTCCT 4480
ACCTTTGTACGCTGGTCCCTTGGGTAGTTATTTCTGCATCGAATCCGAACCGCCACCCGTTTTTGAGAGCATCTAGAGGA
750 745 740 735 730
Pro Phe Met Arg Gly Pro Phe Gly Asp Ile Phe Val Tyr Ser Leu Ser Pro Pro Pro Cys Phe Ser Glu Tyr Ile Glu Glu
ARG1
Intron2 of CARG

CAATCCTCTTCACCTTACTGTCCGGGTAGTGAGTTCGACAATTGGCTTCCTTGCCCATGGTACAGCGCAGGCCTAGCTCT 4560
GTTAGGAGAAGTGGAATGACAGGCCCATCACTCAAGCTGTTAACCGAAGGAACGGGTACCATGTCGCGTCCGGATCGAGA
725 720 715 710 705
Ile Arg Lys Val Lys Ser Asp Pro Tyr His Thr Arg Cys Asn Ala Glu Lys Gly Met Thr Cys Arg Leu Gly Leu Glu
ARG1
Intron2 of CARG

TTCAGGTAACCCTTGTCGCACAATACGGGCTCAGTTGGCGGTGTCGCTGTCTCCAGCCGGATAACCCAGAGGCGTCGTAT 4640
AAGTCCATTGGGAACAGCGTGTTATGCCCGAGTCAACCGCCACAGCGACAGAGGTCGGCCTATTGGGTCTCCGCAGCATA
700 695 690 685 680 675
Lys Leu Tyr Gly Lys Asp Cys Leu Val Pro Glu Thr Pro Pro Thr Ala Thr Glu Leu Arg Ile Val Trp Leu Arg Arg Ile
ARG1
Intron2 of CARG

CATGGAGAGTGCCCTCAGTTCATCTAATCTGAAACCATCAGTACCGTTTTGGAAAACTGATCTAAGGTTATCTATCTGCC 4720
GTACCTCTCACGGGAGTCAAGTAGATTAGACTTTGGTAGTCATGGCAAAACCTTTTGACTAGATTCCAATAGATAGACGG
670 665 660 655 650
Met Ser Leu Ala Arg Leu Glu Asp Leu Arg Phe Gly Asp Thr Gly Asn Gln Phe Val Ser Arg Leu Asn Asp Ile Gln Arg
ARG1
Intron2 of CARG

TGAAATTCTCAATACCTTTCGGAACCTGCGCCAGTCCTGTGTTTCCTATCTGGAGGAAGCTTATTGTGGTCAGCCTCATC 4800
ACTTTAAGAGTTATGGAAAGCCTTGGACGCGGTCAGGACACAAAGGATAGACCTCCTTCGAATAACACCAGTCGGAGTAG
645 640 635 630 625
Phe Asn Glu Ile Gly Lys Pro Val Gln Ala Leu Gly Thr Asn Gly Ile Gln Leu Phe Ser Ile Thr Thr Leu Arg Met
ARG1
Intron2 of CARG

AGACTTTGTGGCAAAGCTGCCAACTTTGTGCAACCAAACACTGAAAGGCAACCAAGGCTGGTAAGGTTTCCGATGGACCC 4880
TCTGAAACACCGTTTCGACGGTTGAAACACGTTGGTTTGTGACTTTCCGTTGGTTCCGACCATTCCAAAGGCTACCTGGG
620 615 610 605 600 595
Leu Ser Gln Pro Leu Ala Ala Leu Lys Thr Cys Gly Phe Val Ser Leu Cys Gly Leu Ser Thr Leu Asn Gly Ile Ser Gly
ARG1
Intron2 of CARG

CGGAAGCTCCTTGATCTCGTTGTAGCTGAGATCCAGCAGCCTCAGCAGCGCCAAGTAACCCACCGATGCAGGTATGCTCT 4960
GCCTTCGAGGAACTAGAGCAACATCGACTCTAGGTCGTCGGAGTCGTCGCGGTTCATTGGGTGGCTACGTCCATACGAGA
590 585 580 575 570
Pro Leu Glu Lys Ile Glu Asn Tyr Ser Leu Asp Leu Leu Arg Leu Leu Ala Leu Tyr Gly Val Ser Ala Pro Ile Ser Glu
ARG1
Intron2 of CARG

CGAGGCCTGCTCCACGTAGAACTAAGATGCGAAGATGCACCAGCTTTCTGAAGATGTCCCTCTTCACCGATCTGCATGTG 5040
GCTCCGGACGAGGTGCATCTTGATTCTACGCTTCTACGTGGTCGAAAGACTTCTACAGGGAGAAGTGGCTAGACGTACAC
565 560 555 550 545
Leu Gly Ala Gly Arg Leu Val Leu Ile Arg Leu His Val Leu Lys Arg Phe Ile Asp Arg Lys Val Ser Arg Cys Thr
ARG1
Intron2 of CARG

TCGTGATCCAAGACAATGAGGCACCGTAGGCGCTTCTTCTCTTCTATAGAAGGTATTTCGTCAACAGCGCTCCCGACACC 5120
AGCACTAGGTTCTGTTACTCCGTGGCATCCGCGAAGAAGAGAAGATATCTTCCATAAAGCAGTTGTCGCGAGGGCTGTGG
540 535 530 525 520 515
Asp His Asp Leu Val Ile Leu Cys Arg Leu Arg Lys Lys Glu Glu Ile Ser Pro Ile Glu Asp Val Ala Ser Gly Val Gly
ARG1
Intron2 of CARG

TAATCGGCGAATACTAGAAGGGCAACGATCACGCTTCTCGTTCATGAAGACGGCTTCATTCCTTGTCAGAAATTGGCCAA 5200
ATTAGCCGCTTATGATCTTCCCGTTGCTAGTGCGAAGAGCAAGTACTTCTGCCGAAGTAAGGAACAGTCTTTAACCGGTT
510 505 500 495 490
Leu Arg Arg Ile Ser Ser Pro Cys Arg Asp Arg Lys Glu Asn Met Phe Val Ala Glu Asn Arg Thr Leu Phe Gln Gly Leu
ARG1
Intron2 of CARG

```
GTTGCCTTAACAGGTCATGCATTGTCGATATTCCCTTGTCGACATACTCTGGCCTTGCCTGTAGCAGATTCCTCTTGATC   5280
CAACGGAATTGTCCAGTACGTAACAGCTATAAGGGAACAGCTGTATGAGACCGGAACGGACATCGTCTAAGGAGAACTAG
    485                 480                 475                 470                 465
Gln Arg Leu Leu Asp His Met Thr Ser Ile Gly Lys Asp Val Tyr Glu Pro Arg Ala Gln Leu Leu Asn Arg Lys Ile
< ARG1
Intron2 of CARG

AGCTCATGGTAGTAATCTTCGGCGACGTTATGTATTGGTCCACTGGTCTCTTCCTTCACAAGACCTTCAGCAATCCACCA   5360
TCGAGTACCATCATTAGAAGCCGCTGCAATACATAACCAGGTGACCAGAGAAGGAAGTGTTCTGGAAGTCGTTAGGTGGT
460                 455                 450                 445                 440                 435
Leu Glu His Tyr Tyr Asp Glu Ala Val Asn His Ile Pro Gly Ser Thr Glu Glu Lys Val Leu Gly Glu Ala Ile Trp Trp
< ARG1
Intron2 of CARG

GTATGTGACATCTCGGTGAATATCGAAATTCTGGGGCAACAAGGCACACCAGAGGAAGCACTGTTTCAGTTGTGGGTGTA   5440
CATACACTGTAGAGCCACTTATAGCTTTAAGACCCCGTTGTTCCGTGTGGTCTCCTTCGTGACAAAGTCAACACCCACAT
            430                 425                 420                 415                 410
Tyr Thr Val Asp Arg His Ile Asp Phe Asn Gln Pro Leu Leu Ala Cys Trp Leu Phe Cys Gln Lys Leu Gln Pro His Leu
< ARG1
Intron2 of CARG

AGTCACTGTAGCTTAAGTACAAAGCACCTTCTAGTTCTTCTGGAAGCCCATCAATAGACCATCTTCTCTCCAGTATTCTC   5520
TCAGTGACATCGAATTCATGTTTCGTGGAAGATCAAGAAGACCTTCGGGTAGTTATCTGGTAGAAGAGAGGTCATAAGAG
    405                 400                 395                 390                 385
Asp Ser Tyr Ser Leu Tyr Leu Ala Gly Glu Leu Glu Glu Pro Leu Gly Asp Ile Ser Trp Arg Arg Glu Leu Ile Arg
< ARG1
Intron2 of CARG

TCCCATTCTTCTTTGCTCGACCTGGATGATAGGACGCCCCCAATGACCTTGATGGCAAGCGGAAGGCCATCACATTTCTT   5600
AGGGTAAGAAGAAACGAGCTGGACCTACTATCCTGCGGGGGTTACTGGAACTACCGTTCGCCTTCCGGTAGTGTAAAGAA
380                 375                 370                 365                 360                 355
Glu Trp Glu Glu Lys Ser Ser Arg Ser Ser Leu Val Gly Gly Ile Val Lys Ile Ala Leu Pro Leu Gly Asp Cys Lys Lys
< ARG1
Intron2 of CARG

AACAATTTTTGCTCCAATATCACTGAATACATTTACTTCATCTTCAGTTCTGAAAGATCTCTTCATAAGCAGTTCTAGGC   5680
TTGTTAAAAACGAGGTTATAGTGACTTATGTAAATGAAGTAGAAGTCAAGACTTTCTAGAGAAGTATTCGTCAAGATCCG
            350                 345                 340                 335                 330
Val Ile Lys Ala Gly Ile Asp Ser Phe Val Asn Val Glu Asp Glu Thr Arg Phe Ser Arg Lys Met Leu Leu Glu Leu Gly
< ARG1
Intron2 of CARG

CATCAGCATCCTTTAGTCTATGCATTTCCTTGACATGAACCGCATTCATCTGTGACAATACGTGACCGCTCCTTGTGGTA   5760
GTAGTCGTAGGAAATCAGATACGTAAAGGAACTGTACTTGGCGTAAGTAGACACTGTTATGCACTGGCGAGGAACACCAT
    325                 320                 315                 310                 305
Asp Ala Asp Lys Leu Arg His Met Glu Lys Val His Val Ala Asn Met Gln Ser Leu Val His Gly Ser Arg Thr Thr
< ARG1
Intron2 of CARG
```

ACAAGTACATGAGCATCCAAACACCTCTCCATCGGAGAGCGGAGAAGATCGATCCACACATCAGGGTTGGTAACGTTATC 5840
TGTTCATGTACTCGTAGGTTTGTGGAGAGGTAGCCTCTCGCCTCTTCTAGCTAGGTGTGTAGTCCCAACCATTGCAATAG

300 295 290 285 280 275
Val Leu Val His Ala Asp Leu Cys Arg Glu Met Pro Ser Arg Leu Leu Asp Ile Trp Val Asp Pro Asn Thr Val Asn Asp
ARG1

Intron2 of CARG

CAGCACAAGAAAGACACTCTTCCCACTGATGGAATGTAGCAGAATTTGCACGAGCTGATCCTTACTCTTGTGCTGATTGC 5920
GTCGTGTTCTTTCTGTGAGAAGGGTGACTACCTTACATCGTCTTAAACGTGCTCGACTAGGAATGAGAACACGACTAACG

270 265 260 255 250
Leu Val Leu Phe Val Ser Lys Gly Ser Ile Ser His Leu Leu Ile Gln Val Leu Gln Asp Lys Ser Lys His Gln Asn Cys
ARG1

Intron2 of CARG

ACTGCCCTCCTGCCTTTTCGATGGCCTCCTTGAGTAGATCATTCTCGGCAATGCTCTGTGAAATGGACAGCCAGATGAGG 6000
TGACGGGAGGACGGAAAAGCTACCGGAGGAACTCATCTAGTAAGAGCCGTTACGAGACACTTTACCTGTCGGTCTACTCC

245 240 235 230 225
Gln Gly Gly Ala Lys Glu Ile Ala Glu Lys Leu Leu Asp Asn Glu Ala Ile Ser Gln Ser Ile Ser Leu Trp Ile Leu
ARG1

Intron2 of CARG

ACCTGGTGAAATCTCTCTCTTATCCTACGATCATTGTATATCTTCTGAGCTAAAGTTGTCTTGCCAATTCCTCCCATCCC 6080
TGGACCACTTTAGAGAGAGAATAGGATGCTAGTAACATATAGAAGACTCGATTTCAACAGAACGGTTAAGGAGGGTAGGG

220 215 210 205 200 195
Val Gln His Phe Arg Glu Arg Ile Arg Arg Asp Asn Tyr Ile Lys Gln Ala Leu Thr Thr Lys Gly Ile Gly Gly Met Gly
ARG1

Intron2 of CARG

AACGATCCCATAAACTGATATCCTGCTCTGATGGCCATAGCCGACGATCATTTCTACCATACTATCAGTAGCTTCCTTGA 6160
TTGCTAGGGTATTTGACTATAGGACGAGACTACCGGTATCGGCTGCTAGTAAAGATGGTATGATAGTCATCGAAGGAACT

190 185 180 175 170
Val Ile Gly Tyr Val Ser Ile Arg Ser Gln His Gly Tyr Gly Val Ile Met Glu Val Met Ser Asp Thr Ala Glu Lys Ile
ARG1

Intron2 of CARG

TTTCTGCTCCAACAACATGGATTTCATCAACCGAAGCAGCCAGATGCCTGCTATCAACTCTGTTTGTTTGAGGGGCCTGA 6240
AAAGACGAGGTTGTTGTACCTAAAGTAGTTGGCTTCGTCGGTCTACGGACGATAGTTGAGACAAACAAACTCCCCGGACT

165 160 155 150 145
Glu Ala Gly Val Val His Ile Glu Asp Val Ser Ala Ala Leu His Arg Ser Asp Val Arg Asn Thr Gln Pro Ala Gln
ARG1

Intron2 of CARG

GCCTGAGGATACAACCCCGGAGGAAGGAACATATCTCTGTTCTTTTGGATTTCTACAAGCCTTTCGTTTACACCCTTGAT 6320
CGGACTCCTATGTTGGGGCCTCCTTCCTTGTATAGAGACAAGAAAACCTAAAGATGTTCGGAAAGCAAATGTGGGAACTA

140 135 130 125 120 115
Ala Gln Pro Tyr Leu Gly Pro Pro Leu Phe Met Asp Arg Asn Lys Gln Ile Glu Val Leu Arg Glu Asn Val Gly Lys Ile
ARG1

Intron2 of CARG

TTTTCTGGCAATCATGTGGTCAAATTGAAGTTGTGCAAACCTAGAGAGCATGCGTAAATCACAACATCGTTGCTTCTGAG 6400
AAAAGACCGTTAGTACACCAGTTTAACTTCAACACGTTTGGATCTCTCGTACGCATTTAGTGTTGTAGCAACGAAGACTC

110 105 100 95 90
Lys Arg Ala Ile Met His Asp Phe Gln Leu Gln Ala Phe Arg Ser Leu Met Arg Leu Asp Cys Cys Arg Gln Lys Gln Ser
ARG1
Intron2 of CARG

AGTGAGCCCTCAAAAGATCTATGATGTCATCAACATCATACATGACATCTTTCACGTCACCCCACCATATTCCTGTCGTC 6480
TCACTCGGGAGTTTTCTAGATACTACAGTAGTTGTAGTATGTACTGTAGAAAGTGCAGTGGGGTGGTATAAGGACAGCAG

85 80 75 70 65
His Ala Arg Leu Leu Asp Ile Ile Asp Asp Val Asp Tyr Met Val Asp Lys Val Asp Gly Trp Trp Ile Gly Thr Thr
ARG1
Intron2 of CARG

TCTCTGTGTTCCATTGCCCGAGCATCAGCATCCTCACGAATGGAATTGAAGTACTTGAGGTAGTCATGGAGCTTCCTGAT 6560
AGAGACACAAGGTAACGGGCTCGTAGTCGTAGGAGTGCTTACCTTAACTTCATGAACTCCATCAGTACCTCGAAGGACTA

60 55 50 45 40 35
Glu Arg His Glu Met Ala Arg Ala Asp Ala Asp Glu Arg Ile Ser Asn Phe Tyr Lys Leu Tyr Asp His Leu Lys Arg Ile
ARG1
Intron2 of CARG

ATCTTTACGCACAGAAAGCGTCATCATAACCTCCTCCTCCAGCAGTACAGCAAGCTTGTCAAGGCATTTGGATGATAGAC 6640
TAGAAATGCGTGTCTTTCGCAGTAGTATTGGAGGAGGAGGTCGTCATGTCGTTCGAACAGTTCCGTAAACCTACTATCTG

30 25 20 15 10
Asp Lys Arg Val Ser Leu Thr Met Met Val Glu Glu Glu Leu Leu Val Ala Leu Lys Asp Leu Cys Lys Ser Ser Leu Ser
ARG1
Intron2 of CARG

TAAACAACACTGAGCCCATGGCGCGGCACCTCTACTTGTCTTGGCTTCTCCGCCGCAGGTACTACACAGCAAAGAAAGGG 6720
ATTTGTTGTGACTCGGGTACCGCGCCGTGGAGATGAACAGAACCGAAGAGGCGGCGTCCATGATGTGTCGTTTCTTTCCC

5 1
Phe Leu Val Ser Gly Met
ARG1
ARG1 promoter
Intron2 of CARG
5'UTR

ATTAGAAACTGCAGCGCACTACTACAAGCCTGGAGATGTTAGAATTGAATCTTCCTACCTGAAGCACTCCGATTGATCTG 6800
TAATCTTTGACGTCGCGTGATGATGTTCGGACCTCTACAATCTTAACTTAGAAGGATGGACTTCGTGAGGCTAACTAGAC

Intron2 of CARG
ARG1 promoter
5'UTR

GTGACTGGTGAGGAACCGAAGTGCGGAAGCAAGGAGGCGTCAGATCAGATCTGTAGGAAGCATCCTTGGAAACTCTCTCT 6880
CACTGACCACTCCTTGGCTTCACGCCTTCGTTCCTCCGCAGTCTAGTCTAGACATCCTTCGTAGGAACCTTTGAGAGAGA

Intron2 of CARG
ARG1 promoter
5'UTR

CCCAACCTTCAGCAGAATGGCTCTCTTTTGGACGCAACTACTTTTCCTTGTTTATGGGTCCACCTAAATGGTAGTAGTTG 6960
GGGTTGGAAGTCGTCTTACCGAGAGAAAACCTGCGTTGATGAAAAGGAACAAATACCCAGGTGGATTTACCATCATCAAC

Intron2 of CARG

ARG1 promoter

5'UTR

ACGATGACTAGCCTAACCGAATATGCCAACTAAGCGGGCGCAAGGAACACCGGCAAAAATGAGTAAATTGTCTATGATTA 7040
TGCTACTGATCGGATTGGCTTATACGGTTGATTCGCCCGCGTTCCTTGTGGCCGTTTTTACTCATTTAACAGATACTAAT

Intron2 of CARG

ARG1 promoter

5'UTR

AACCCGTGACTGTAGAGAAGCAGCACTGCGTAGCAACCAGCAACTAAAAGAGACATCTTGTAAGGGTCTATTTATTAGCT 7120
TTGGGCACTGACATCTCTTCGTCGTGACGCATCGTTGGTCGTTGATTTTCTCTGTAGAACATTCCCAGATAAATAATCGA

Intron2 of CARG

ARG1 promoter

5'UTR

TACAAACTGCTTATGACAAACATAAGCTGAAATTAGAAGCCAAACAATACGTTTTCTACAGCGTTTTCTAGCCACTCTTC 7200
ATGTTTGACGAATACTGTTTGTATTCGACTTTAATCTTCGGTTTGTTATGCAAAAGATGTCGCAAAAGATCGGTGAGAAG

Intron2 of CARG

ARG1 promoter

5'UTR

TCCCACAACTCTCATTTATATTAAAATTGGATTTGACCAGCTAATTTTTACACTGTTTTTCTTTTATTTTTTGCAGGTTC 7280
AGGGTGTTGAGAGTAAATATAATTTTAACCTAAACTGGTCGATTAAAAATGTGACAAAAAGAAAATAAAAAACGTCCAAG

Intron2 of CARG

3'UTR of CARG

ARG1 promoter

5'UTR

TCAGGACAGCGTTTGTCCCTACGTGCTCTATCAAGCAATGCTAAGAGCAACTCCAACACTACCCTAAACTTCACTTGGCA 7360
AGTCCTGTCGCAAACAGGGATGCACGAGATAGTTCGTTACGATTCTCGTTGAGGTTGTGATGGGATTTGAAGTGAACCGT

ARG1 promoter

3'UTR of CARG

5'UTR

MITE

AATCTTGGATTTTGCCAAGTGTAAAACTCAAATGCCAAGTGAAAAAAAGAAGCTTCTCCAACAGTTAGCTATTTTTTTGG 7440
TTAGAACCTAAAACGGTTCACATTTTGAGTTTACGGTTCACTTTTTTTCTTCGAAGAGGTTGTCAATCGATAAAAAAACC

ARG1 promoter

3'UTR of CARG

MITE

CAAATTTGGACTATCTTGTGATTCCTGAAAAAATCATAGGGACTTTTATGCAAAAGTTACAATCTATTTAAGGCATGCGT 7520
GTTTAAACCTGATAGAACACTAAGGACTTTTTTAGTATCCCTGAAAATACGTTTTCAATGTTAGATAAATTCCGTACGCA

ARG1 promoter

3'UTR of CARG

MITE

GAACGAGCGTGGTGCTGGAGCGTGCATGCTGAATGTGTGGGCCCCACCGCGTAGGCAGGATGCCAAGCGCTCCCAACGCT 7600
CTTGCTCGCACCACGACCTCGCACGTACGACTTACACACCCGGGGTGGCGCATCCGTCCTACGGTTCGCGAGGGTTGCGA

ARG1 promoter

3'UTR of CARG

MITE

CCCAAATTTGAGCCAGTTGGCCCTCTTTTTGTCAAGCATCCTATTTTGCCAAGTGCATATAGTGTAGTGTTGGAGGAGCT 7680
GGGTTTAAACTCGGTCAACCGGGAGAAAAACAGTTCGTAGGATAAAACGGTTCACGTATATCACATCACAACCTCCTCGA

ARG1 promoter

3'UTR of CARG

MITE

TTTTTCATGTTTTGCCAAAAATTAGGATTTGCTAAGTGCATATAGGATAGTCTTGGAGTTGCTCTAAGAACGTGAAATGC 7760
AAAAAGTACAAAACGGTTTTTAATCCTAAACGATTCACGTATATCCTATCAGAACCTCAACGAGATTCTTGCACTTTACG

ARG1 promoter

3'UTR of CARG

MITE

5'UTR

TTTTACATTTGGATAGTGACTCTTGAACAACAAATTAAAGCGACCTGCTACTTTCGTTTCGGTTCAGCCTATTTATTTTC 7840
AAAATGTAAACCTATCACTGAGAACTTGTTGTTTAATTTCGCTGGACGATGAAAGCAAAGCCAAGTCGGATAAATAAAAG

ARG1 promoter

3'UTR of CARG

5'UTR

TCTCCCTCGTGCTGTCAGCCAGTCTCTAGTCCATCTACTCTTAGCTTTATTCGCCCACCTGCCAAGTGCGAAAAATAACG 7920
AGAGGGAGCACGACAGTCGGTCAGAGATCAGGTAGATGAGAATCGAAATAAGCGGGTGGACGGTTCACGCTTTTTATTGC

ARG1 promoter

3'UTR of CARG

5'UTR

TAGTAGTACATTGTATATTCTCCCATTTGCCGATATTAAAAGTCAAAAAATATTGCAATCACGCGGTGCAGTTTTCCATG 8000
ATCATCATGTAACATATAAGAGGGTAAACGGCTATAATTTTCAGTTTTTTATAACGTTAGTGCGCCACGTCAAAAGGTAC

ARG1 promoter

3'UTR of CARG

ACTAAGCGACTGAATAGTATGCAGTTTTTAATGTCGGTTGCTGTTGCTGACTGTGCATGTGGCCCACGGTGAAAGGGACT
8080
TGATTCGCTGACTTATCATACGTCAAAAATTACAGCCAACGACAACGACTGACACGTACACCGGGTGCCACTTTCCCTGA

ARG1 promoter

3'UTR of CARG

TTCGTCTGTGTGGATTCGGTCGAAGATGATAGATGGAAGGCAACAGGGCTTAAAATCTTTATTGATTTATTTAGTGATAG
8160
AAGCAGACACACCTAAGCCAGCTTCTACTATCTACCTTCCGTTGTCCCGAATTTTAGAAATAACTAAATAAATCACTATC

ARG1 promoter

TCAAAATTCGCTATACTATTATTTTGGATGTTTATAGGACGTTTAGCCGCTAAATGGCATTTGGACCCTAAACAGTCCAA
8240
AGTTTTAAGCGATATGATAATAAAACCTACAAATATCCTGCAAATCGGCGATTTACCGTAAACCTGGGATTTGTCAGGTT

ARG1 promoter

TAAACATTTTAACTGGTAAAAATAGTACAAAAATGATTGAGACGGCCTAAACAAAACTGTAAAATATTCATTATAAATAG
8320
ATTTGTAAAATTGACCATTTTTATCATGTTTTTACTAACTCTGCCGGATTTGTTTTGACATTTTATAAGTAATATTTATC

ARG1 promoter

GTTAAATATCTGTTTAAATTAAGCATTAATTAGTCAATAGGAGGATTTCAGCATCCACGTCGGCACCCATAGGGTGTTTA
8400
CAATTTATAGACAAATTTAATTCGTAATTAATCAGTTATCCTCCTAAAGTCGTAGGTGCAGCCGTGGGTATCCCACAAAT

ARG1 promoter

CGATTTTTTCCTACTCCAAAATTTAGTTTGTACTCGCTACAAATAATTATTTTTTACATGCATAAATAAAAAAATATTGC
8480
GCTAAAAAAGGATGAGGTTTTAAATCAAACATGAGCGATGTTTATTAATAAAAAATGTACGTATTTATTTTTTTATAACG

ARG1 promoter

AATCACGCAGTGCAGTTTTCCATGACTAAGCGACAGAATAGTATGCAGTTTTTAATGTCTGTTGCTGTTACTTGTCAAAA
8560
TTAGTGCGTCACGTCAAAAGGTACTGATTCGCTGTCTTATCATACGTCAAAAATTACAGACAACGACAATGAACAGTTTT

ARG1 promoter

AAAAAAAGTCTGTTGCTGTTGCTGACTGTGCATGTGGCTCACGGCGAAAGTGCGAAAGGGACTTTGTTTGTGTGGATTCG
8640
TTTTTTTCAGACAACGACAACGACTGACACGTACACCGAGTGCCGCTTTCACGCTTTCCCTGAAACAAACACACCTAAGC

ARG1 promoter

GTCGAAGATAATAGATGGAGGCAACG 3′
8666
CAGCTTCTATTATCTACCTCCGTTGC 5′

ARG1 promoter

	Feature	Location	Size (bp)			Type
✓	CARG promoter	1 .. 1284	1284			promoter
✓	5'UTR of CARG	1146 .. 1284	139			5'UTR
✓	Exon1 of CARG	1285 .. 1427	143			exon
✓	Intron1 of CARG	1428 .. 1909	482			intron
✓	Exon2 of CARG	1910 .. 1997	88			exon
✓	3'UTR of CARG	1998 .. 2091	94			3'UTR
✓	Intron2 of CARG	2092 .. 7273	5182			intron
✓	MITE	2329 .. 2479	151			MITE
✓	3'UTR of ARG1	3536 .. 3686	151		←	3'UTR
✓	ARG1	3687 .. 6659	2973		←	CDS

/translation = MGSVLFSLSSKCLDKLAVLLEEEVMMTLSVRKDIRKLHDYLKYFNSIREDADARAMEHRETTGIWWGDVKDVMYDVDDIIDLLRAHSQKQRCCDLRMLSRFAQLQFDHMIARKIKGVN
VEIQKNRDMFLPPGLYPQAQAPQTNRVDSRHLAASVDEIHVVGAEIKEATDSMVEMIVGYGHQSRISVYGIVGMGGIGKTTLAQKIYNDRRIRERFHQVLIWLSISQSIAENDLLKEAIE
GQCNQHKSKDQLVQILLHSISGKSVFLVLDNVTNPDVWIDLLRSPMERCLDAHVLVTTRSGHVLSQMNAVHVKEMHRLKDADGLELLMKRSFRTEDEVNVFSDIGAKIVKKCDGLPLA
GGVLSSRSSKEEWERILERRWSIDGLPEELEGALYLSYSDLHPQLKQCFLWCALLPQNFDIHRDVTYWWIAEGLVKEETSGPIHNVAEDYYHELIKRNLLQARPEYVDKGISTMHDLLR
FLTRNEAVFMNEKRDRCPSSIRRLGVGSAVDEIPSIEEKKRLRCLIVLDHDTCRSVKRDIFRKLVHLRILVLRGAGLESIPASVGYLALLRLLDLSYNEIKELPGSIGNLTSLGCLSVFGCTK
PQSLMRLTTISFLQIGNTGLAQVPKGIENFRQIDNLRSVFQNGTDGFRLDELRALSMIRRLWVIRLETATPPTEPVLCDKGYLKELGLRCTMGKEANCRTHYPDSKVKRIEEIYESFCPPPS
VFIDGFPGRMFPTWLSLEPQNKLPNLAHMHFYDCISCPKLPPAGQLPFLQVLHVKGADAVVNIGAELLGNGIPSGTHTTAFPKLELLEILDMYNWQNWSLSMDTLFENTQQQFLMPCLT
NCPKLRALPDHLHRVVNLQRIQIEGADSLQEVVNHPGVVWLKVKNNKSLRNISNLPKLRLLLAQDCQELQQAENLSSLKALYVVDCPMEQILWKCFPIEQQSTIVRVVTTGAHGQDIYP
FH*

990 amino acids = 112.7 kDa

	Feature	Location	Size (bp)			Type
✓	ARG1 promoter	6660 .. 8666	2007			promoter
✓	5'UTR	6660 .. 6946	287		←	5'UTR
✓	5'UTR	6980 .. 7320	341		←	5'UTR
✓	3'UTR of CARG	7274 .. 8048	775		→	3'UTR
✓	MITE	7325 .. 7744	420			MITE
✓	5'UTR	7744 .. 7855	112		←	5'UTR

Supplemental Figure S2. Genomic structures and sequences of *CARG-ARG1* genes in TAM428 and SC283.
