# Supplementary material for: Broad-spectrum fungal resistance in sorghum is conferred through the complex regulation of an immune receptor gene embedded in a natural antisense transcript
Source: Plant Cell. 2022 Jan 9;34(5):1641–65. doi: 10.1093/plcell/koab305 (PMC9048912; doi:10.1093/plcell/koab305)
Supplement: koab305_Supplementary_Data [file koab305_supplementary_data.zip › tpc.21.00809_SupplementalFile4.pdf]

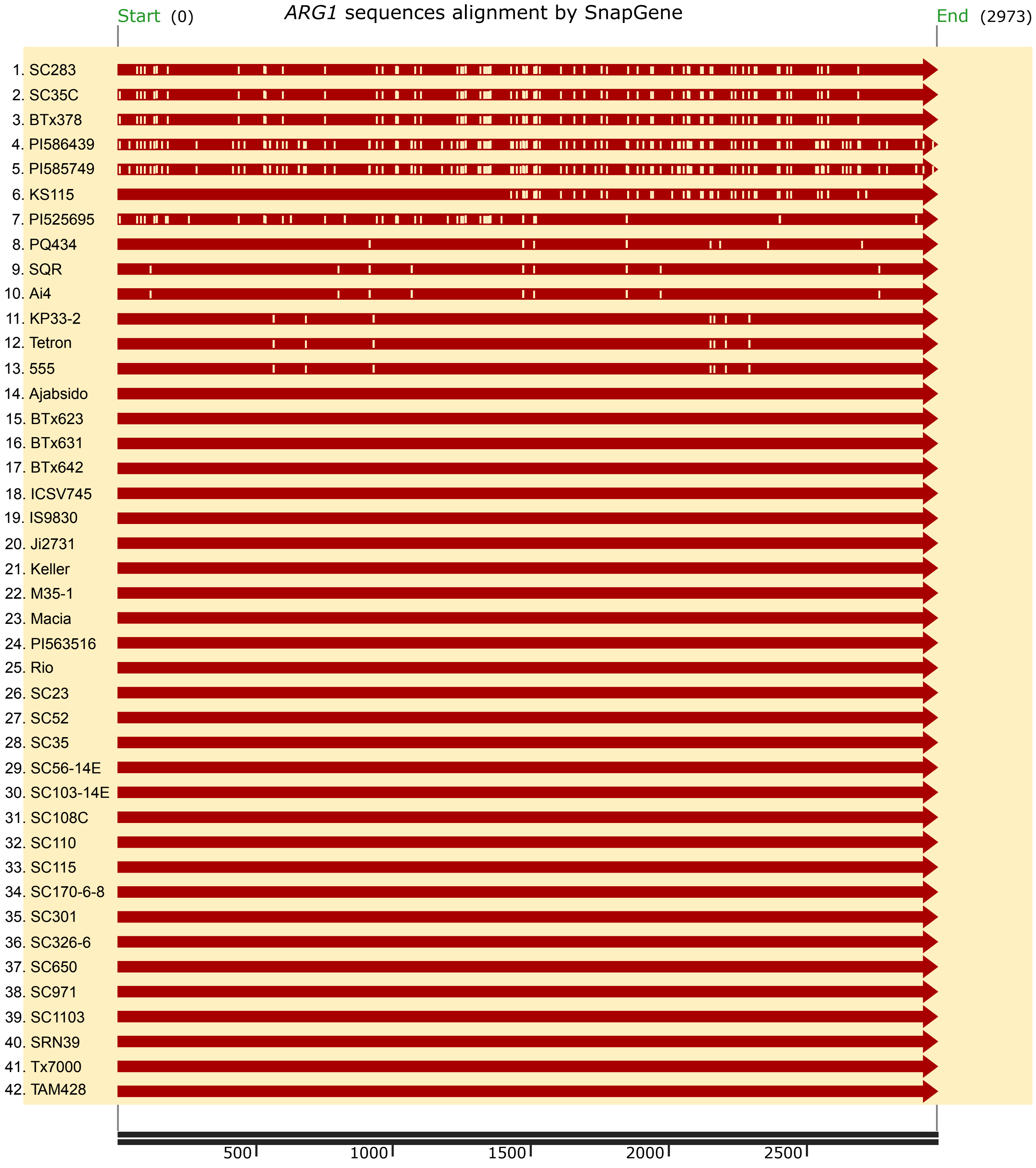

Start (0)
ARG1 sequences alignment by SnapGene
End (2973)
1. SC283
2. SC35C
3. BTx378
4. PI586439
5. PI585749
6. KS115
7. PI525695
8. PQ434
9. SQR
10. Ai4
11. KP33-2
12. Tetron
13. 555
14. Ajabsido
15. BTx623
16. BTx631
17. BTx642
18. ICSV745
19. IS9830
20. Ji2731
21. Keller
22. M35-1
23. Macia
24. PI563516
25. Rio
26. SC23
27. SC52
28. SC35
29. SC56-14E
30. SC103-14E
31. SC108C
32. SC110
33. SC115
34. SC170-6-8
35. SC301
36. SC326-6
37. SC650
38. SC971
39. SC1103
40. SRN39
41. Tx7000
42. TAM428
500
1000
1500
2000
2500

	10 20 30 40 50 60
	ATGGGCTCAGTGTTGTTTAGTCTATCATCCAAATGCCTTGACAAGCTTGCTGTACTGCTGGAGGA
1	ATGGGCTCAGTGTTGTTTAGTCTATCATCCAAATGCCTTGACAAGCTTGCTGTACTGCTGGAGGA
2	ATGGGCTCGGTGTTGTTTAGTCTATCATCCAAATGCCTTGACAAGCTTGCTGTACTGCTGGAGGA
3	ATGGGCTCGGTGTTGTTTAGTCTATCATCCAAATGCCTTGACAAGCTTGCTGTACTGCTGGAGGA
4	ATGGGCTCGGTGTTGTTTAGTCTATCATCCAAATGCCTTAACAAGCTTGCTGTACTGCTGGAGGA
5	ATGGGCTCGGTGTTGTTTAGTCTATCATCCAAATGCCTTAACAAGCTTGCTGTACTGCTGGAGGA
6	ATGGGCTCAGTGTTGTTTAGTCTATCATCCAAATGCCTTGACAAGCTTGCTGTACTGCTGGAGGA
7	ATGGGCTCGGTGTTGTTTAGTCTATCATCCAAATGCCTTGACAAGCTTGCTGTACTGCTGGAGGA
8	ATGGGCTCAGTGTTGTTTAGTCTATCATCCAAATGCCTTGACAAGCTTGCTGTACTGCTGGAGGA
9	ATGGGCTCAGTGTTGTTTAGTCTATCATCCAAATGCCTTGACAAGCTTGCTGTACTGCTGGAGGA
10	ATGGGCTCAGTGTTGTTTAGTCTATCATCCAAATGCCTTGACAAGCTTGCTGTACTGCTGGAGGA
11	ATGGGCTCAGTGTTGTTTAGTCTATCATCCAAATGCCTTGACAAGCTTGCTGTACTGCTGGAGGA
12	ATGGGCTCAGTGTTGTTTAGTCTATCATCCAAATGCCTTGACAAGCTTGCTGTACTGCTGGAGGA
13	ATGGGCTCAGTGTTGTTTAGTCTATCATCCAAATGCCTTGACAAGCTTGCTGTACTGCTGGAGGA
14	ATGGGCTCAGTGTTGTTTAGTCTATCATCCAAATGCCTTGACAAGCTTGCTGTACTGCTGGAGGA
15	ATGGGCTCAGTGTTGTTTAGTCTATCATCCAAATGCCTTGACAAGCTTGCTGTACTGCTGGAGGA
16	ATGGGCTCAGTGTTGTTTAGTCTATCATCCAAATGCCTTGACAAGCTTGCTGTACTGCTGGAGGA
17	ATGGGCTCAGTGTTGTTTAGTCTATCATCCAAATGCCTTGACAAGCTTGCTGTACTGCTGGAGGA
18	ATGGGCTCAGTGTTGTTTAGTCTATCATCCAAATGCCTTGACAAGCTTGCTGTACTGCTGGAGGA
19	ATGGGCTCAGTGTTGTTTAGTCTATCATCCAAATGCCTTGACAAGCTTGCTGTACTGCTGGAGGA
20	ATGGGCTCAGTGTTGTTTAGTCTATCATCCAAATGCCTTGACAAGCTTGCTGTACTGCTGGAGGA
21	ATGGGCTCAGTGTTGTTTAGTCTATCATCCAAATGCCTTGACAAGCTTGCTGTACTGCTGGAGGA
22	ATGGGCTCAGTGTTGTTTAGTCTATCATCCAAATGCCTTGACAAGCTTGCTGTACTGCTGGAGGA
23	ATGGGCTCAGTGTTGTTTAGTCTATCATCCAAATGCCTTGACAAGCTTGCTGTACTGCTGGAGGA
24	ATGGGCTCAGTGTTGTTTAGTCTATCATCCAAATGCCTTGACAAGCTTGCTGTACTGCTGGAGGA
25	ATGGGCTCAGTGTTGTTTAGTCTATCATCCAAATGCCTTGACAAGCTTGCTGTACTGCTGGAGGA
26	ATGGGCTCAGTGTTGTTTAGTCTATCATCCAAATGCCTTGACAAGCTTGCTGTACTGCTGGAGGA
27	ATGGGCTCAGTGTTGTTTAGTCTATCATCCAAATGCCTTGACAAGCTTGCTGTACTGCTGGAGGA
28	ATGGGCTCAGTGTTGTTTAGTCTATCATCCAAATGCCTTGACAAGCTTGCTGTACTGCTGGAGGA
29	ATGGGCTCAGTGTTGTTTAGTCTATCATCCAAATGCCTTGACAAGCTTGCTGTACTGCTGGAGGA
30	ATGGGCTCAGTGTTGTTTAGTCTATCATCCAAATGCCTTGACAAGCTTGCTGTACTGCTGGAGGA
31	ATGGGCTCAGTGTTGTTTAGTCTATCATCCAAATGCCTTGACAAGCTTGCTGTACTGCTGGAGGA
32	ATGGGCTCAGTGTTGTTTAGTCTATCATCCAAATGCCTTGACAAGCTTGCTGTACTGCTGGAGGA
33	ATGGGCTCAGTGTTGTTTAGTCTATCATCCAAATGCCTTGACAAGCTTGCTGTACTGCTGGAGGA
34	ATGGGCTCAGTGTTGTTTAGTCTATCATCCAAATGCCTTGACAAGCTTGCTGTACTGCTGGAGGA
35	ATGGGCTCAGTGTTGTTTAGTCTATCATCCAAATGCCTTGACAAGCTTGCTGTACTGCTGGAGGA
36	ATGGGCTCAGTGTTGTTTAGTCTATCATCCAAATGCCTTGACAAGCTTGCTGTACTGCTGGAGGA
37	ATGGGCTCAGTGTTGTTTAGTCTATCATCCAAATGCCTTGACAAGCTTGCTGTACTGCTGGAGGA
38	ATGGGCTCAGTGTTGTTTAGTCTATCATCCAAATGCCTTGACAAGCTTGCTGTACTGCTGGAGGA
39	ATGGGCTCAGTGTTGTTTAGTCTATCATCCAAATGCCTTGACAAGCTTGCTGTACTGCTGGAGGA
40	ATGGGCTCAGTGTTGTTTAGTCTATCATCCAAATGCCTTGACAAGCTTGCTGTACTGCTGGAGGA
41	ATGGGCTCAGTGTTGTTTAGTCTATCATCCAAATGCCTTGACAAGCTTGCTGTACTGCTGGAGGA
42	ATGGGCTCAGTGTTGTTTAGTCTATCATCCAAATGCCTTGACAAGCTTGCTGTACTGCTGGAGGA

70 80 90 100 110 120 130

	GGAGATTATGATGACGCTATCTGTGCGTAAAGAGATCAGGAAGCTCCATGACTACCTCAAGTACT
1	GGAGGTTATGATGACGCTTTCTGTGCGTAAAGATATCAGGAAGCTCCATGACTACCTCAAGTACT
2	GGAGGTTATGATGACGCTTTCTGTGCGTAAAGATATCAGGAAGCTCCATGACTACCTCAAGTACT
3	GGAGGTTATGATGACGCTTTCTGTGCGTAAAGATATCAGGAAGCTCCATGACTACCTCAAGTACT
4	GGAGGTTATGATGACGCTTTCTGTGCGTAAAGATATCAGGAAGCTCCATGAAAACCTCAAGTACT
5	GGAGGTTATGATGACGCTTTCTGTGCGTAAAGATATCAGGAAGCTCCATGAAAACCTCAAGTACT
6	GGAGATTATGATGACGCTATCTGTGCGTAAAGAGATCAGGAAGCTCCATGACTACCTCAAGTACT
7	GGAGGTTATGATGACGCTTTCTGTGCGTAAAGATATCAGGAAGCTCCATGACTACCTCAAGTACT
8	GGAGATTATGATGACGCTATCTGTGCGTAAAGAGATCAGGAAGCTCCATGACTACCTCAAGTACT
9	GGAGATTATGATGACGCTATCTGTGCGTAAAGAGATCAGGAAGCTCCATGAAAACCTCAAGTACT
10	GGAGATTATGATGACGCTATCTGTGCGTAAAGAGATCAGGAAGCTCCATGAAAACCTCAAGTACT
11	GGAGATTATGATGACGCTATCTGTGCGTAAAGAGATCAGGAAGCTCCATGACTACCTCAAGTACT
12	GGAGATTATGATGACGCTATCTGTGCGTAAAGAGATCAGGAAGCTCCATGACTACCTCAAGTACT
13	GGAGATTATGATGACGCTATCTGTGCGTAAAGAGATCAGGAAGCTCCATGACTACCTCAAGTACT
14	GGAGATTATGATGACGCTATCTGTGCGTAAAGAGATCAGGAAGCTCCATGACTACCTCAAGTACT
15	GGAGATTATGATGACGCTATCTGTGCGTAAAGAGATCAGGAAGCTCCATGACTACCTCAAGTACT
16	GGAGATTATGATGACGCTATCTGTGCGTAAAGAGATCAGGAAGCTCCATGACTACCTCAAGTACT
17	GGAGATTATGATGACGCTATCTGTGCGTAAAGAGATCAGGAAGCTCCATGACTACCTCAAGTACT
18	GGAGATTATGATGACGCTATCTGTGCGTAAAGAGATCAGGAAGCTCCATGACTACCTCAAGTACT
19	GGAGATTATGATGACGCTATCTGTGCGTAAAGAGATCAGGAAGCTCCATGACTACCTCAAGTACT
20	GGAGATTATGATGACGCTATCTGTGCGTAAAGAGATCAGGAAGCTCCATGACTACCTCAAGTACT
21	GGAGATTATGATGACGCTATCTGTGCGTAAAGAGATCAGGAAGCTCCATGACTACCTCAAGTACT
22	GGAGATTATGATGACGCTATCTGTGCGTAAAGAGATCAGGAAGCTCCATGACTACCTCAAGTACT
23	GGAGATTATGATGACGCTATCTGTGCGTAAAGAGATCAGGAAGCTCCATGACTACCTCAAGTACT
24	GGAGATTATGATGACGCTATCTGTGCGTAAAGAGATCAGGAAGCTCCATGACTACCTCAAGTACT
25	GGAGATTATGATGACGCTATCTGTGCGTAAAGAGATCAGGAAGCTCCATGACTACCTCAAGTACT
26	GGAGATTATGATGACGCTATCTGTGCGTAAAGAGATCAGGAAGCTCCATGACTACCTCAAGTACT
27	GGAGATTATGATGACGCTATCTGTGCGTAAAGAGATCAGGAAGCTCCATGACTACCTCAAGTACT
28	GGAGATTATGATGACGCTATCTGTGCGTAAAGAGATCAGGAAGCTCCATGACTACCTCAAGTACT
29	GGAGATTATGATGACGCTATCTGTGCGTAAAGAGATCAGGAAGCTCCATGACTACCTCAAGTACT
30	GGAGATTATGATGACGCTATCTGTGCGTAAAGAGATCAGGAAGCTCCATGACTACCTCAAGTACT
31	GGAGATTATGATGACGCTATCTGTGCGTAAAGAGATCAGGAAGCTCCATGACTACCTCAAGTACT
32	GGAGATTATGATGACGCTATCTGTGCGTAAAGAGATCAGGAAGCTCCATGACTACCTCAAGTACT
33	GGAGATTATGATGACGCTATCTGTGCGTAAAGAGATCAGGAAGCTCCATGACTACCTCAAGTACT
34	GGAGATTATGATGACGCTATCTGTGCGTAAAGAGATCAGGAAGCTCCATGACTACCTCAAGTACT
35	GGAGATTATGATGACGCTATCTGTGCGTAAAGAGATCAGGAAGCTCCATGACTACCTCAAGTACT
36	GGAGATTATGATGACGCTATCTGTGCGTAAAGAGATCAGGAAGCTCCATGACTACCTCAAGTACT
37	GGAGATTATGATGACGCTATCTGTGCGTAAAGAGATCAGGAAGCTCCATGACTACCTCAAGTACT
38	GGAGATTATGATGACGCTATCTGTGCGTAAAGAGATCAGGAAGCTCCATGACTACCTCAAGTACT
39	GGAGATTATGATGACGCTATCTGTGCGTAAAGAGATCAGGAAGCTCCATGACTACCTCAAGTACT
40	GGAGATTATGATGACGCTATCTGTGCGTAAAGAGATCAGGAAGCTCCATGACTACCTCAAGTACT
41	GGAGATTATGATGACGCTATCTGTGCGTAAAGAGATCAGGAAGCTCCATGACTACCTCAAGTACT
42	GGAGATTATGATGACGCTATCTGTGCGTAAAGAGATCAGGAAGCTCCATGACTACCTCAAGTACT

140 150 160 170 180 190

	TTGATTCCATTCATGAGGATGCTGATGCTCGGGCAATGGAACACAGAGAGATGACAGGAATATGG
1 ➨	TCAATTCCATTCGTGAGGATGCTGATGCTCGGGCAATGGAACACAGAGAGACGACAGGAATATGG
2 ➨	TCAATTCCATTCGTGAGGATGCTGATGCTCGGGCAATGGAACACAGAGAGACGACAGGAATATGG
3 ➨	TCAATTCCATTCGTGAGGATGCTGATGCTCGGGCAATGGAACACAGAGAGACGACAGGAATATGG
4 ➨	TCAATTCCATTCGTGAGGATGCTGATGCTTGGGCAATGGAACACAGAGAGACGACAGGAATATGG
5 ➨	TCAATTCCATTCGTGAGGATGCTGATGCTTGGGCAATGGAACACAGAGAGACGACAGGAATATGG
6 ➨	TTGATTCCATTCATGAGGATGCTGATGCTCGGGCAATGGAACACAGAGAGATGACAGGAATATGG
7 ➨	TCAATTCCATTCGTGAGGATGCTGATGCTCGGGCAATGGAACACGGAGAGACGACAGGAATATGG
8 ➨	TTGATTCCATTCATGAGGATGCTGATGCTCGGGCAATGGAACACAGAGAGATGACAGGAATATGG
9 ➨	TTGATTCCATTCATGAGGATGCTGATGCTCGGGCAATGGAACACAGAGAGATGACAGGAATATGG
10 ➨	TTGATTCCATTCATGAGGATGCTGATGCTCGGGCAATGGAACACAGAGAGATGACAGGAATATGG
11 ➨	TTGATTCCATTCATGAGGATGCTGATGCTCGGGCAATGGAACACAGAGAGATGACAGGAATATGG
12 ➨	TTGATTCCATTCATGAGGATGCTGATGCTCGGGCAATGGAACACAGAGAGATGACAGGAATATGG
13 ➨	TTGATTCCATTCATGAGGATGCTGATGCTCGGGCAATGGAACACAGAGAGATGACAGGAATATGG
14 ➨	TTGATTCCATTCATGAGGATGCTGATGCTCGGGCAATGGAACACAGAGAGATGACAGGAATATGG
15 ➨	TTGATTCCATTCATGAGGATGCTGATGCTCGGGCAATGGAACACAGAGAGATGACAGGAATATGG
16 ➨	TTGATTCCATTCATGAGGATGCTGATGCTCGGGCAATGGAACACAGAGAGATGACAGGAATATGG
17 ➨	TTGATTCCATTCATGAGGATGCTGATGCTCGGGCAATGGAACACAGAGAGATGACAGGAATATGG
18 ➨	TTGATTCCATTCATGAGGATGCTGATGCTCGGGCAATGGAACACAGAGAGATGACAGGAATATGG
19 ➨	TTGATTCCATTCATGAGGATGCTGATGCTCGGGCAATGGAACACAGAGAGATGACAGGAATATGG
20 ➨	TTGATTCCATTCATGAGGATGCTGATGCTCGGGCAATGGAACACAGAGAGATGACAGGAATATGG
21 ➨	TTGATTCCATTCATGAGGATGCTGATGCTCGGGCAATGGAACACAGAGAGATGACAGGAATATGG
22 ➨	TTGATTCCATTCATGAGGATGCTGATGCTCGGGCAATGGAACACAGAGAGATGACAGGAATATGG
23 ➨	TTGATTCCATTCATGAGGATGCTGATGCTCGGGCAATGGAACACAGAGAGATGACAGGAATATGG
24 ➨	TTGATTCCATTCATGAGGATGCTGATGCTCGGGCAATGGAACACAGAGAGATGACAGGAATATGG
25 ➨	TTGATTCCATTCATGAGGATGCTGATGCTCGGGCAATGGAACACAGAGAGATGACAGGAATATGG
26 ➨	TTGATTCCATTCATGAGGATGCTGATGCTCGGGCAATGGAACACAGAGAGATGACAGGAATATGG
27 ➨	TTGATTCCATTCATGAGGATGCTGATGCTCGGGCAATGGAACACAGAGAGATGACAGGAATATGG
28 ➨	TTGATTCCATTCATGAGGATGCTGATGCTCGGGCAATGGAACACAGAGAGATGACAGGAATATGG
29 ➨	TTGATTCCATTCATGAGGATGCTGATGCTCGGGCAATGGAACACAGAGAGATGACAGGAATATGG
30 ➨	TTGATTCCATTCATGAGGATGCTGATGCTCGGGCAATGGAACACAGAGAGATGACAGGAATATGG
31 ➨	TTGATTCCATTCATGAGGATGCTGATGCTCGGGCAATGGAACACAGAGAGATGACAGGAATATGG
32 ➨	TTGATTCCATTCATGAGGATGCTGATGCTCGGGCAATGGAACACAGAGAGATGACAGGAATATGG
33 ➨	TTGATTCCATTCATGAGGATGCTGATGCTCGGGCAATGGAACACAGAGAGATGACAGGAATATGG
34 ➨	TTGATTCCATTCATGAGGATGCTGATGCTCGGGCAATGGAACACAGAGAGATGACAGGAATATGG
35 ➨	TTGATTCCATTCATGAGGATGCTGATGCTCGGGCAATGGAACACAGAGAGATGACAGGAATATGG
36 ➨	TTGATTCCATTCATGAGGATGCTGATGCTCGGGCAATGGAACACAGAGAGATGACAGGAATATGG
37 ➨	TTGATTCCATTCATGAGGATGCTGATGCTCGGGCAATGGAACACAGAGAGATGACAGGAATATGG
38 ➨	TTGATTCCATTCATGAGGATGCTGATGCTCGGGCAATGGAACACAGAGAGATGACAGGAATATGG
39 ➨	TTGATTCCATTCATGAGGATGCTGATGCTCGGGCAATGGAACACAGAGAGATGACAGGAATATGG
40 ➨	TTGATTCCATTCATGAGGATGCTGATGCTCGGGCAATGGAACACAGAGAGATGACAGGAATATGG
41 ➨	TTGATTCCATTCATGAGGATGCTGATGCTCGGGCAATGGAACACAGAGAGATGACAGGAATATGG
42 ➨	TTGATTCCATTCATGAGGATGCTGATGCTCGGGCAATGGAACACAGAGAGATGACAGGAATATGG

	200 210 220 230 240 250 260
	TGGGGTGACGTGAAAGATGTCATGTATGATGTTGATGACATCATAGATCTTTTGAGGGCTCACTC
1 ➡	TGGGGTGACGTGAAAGATGTCATGTATGATGTTGATGACATCATAGATCTTTTGAGGGCTCACTC
2 ➡	TGGGGTGACGTGAAAGATGTCATGTATGATGTTGATGACATCATAGATCTTTTGAGGGCTCACTC
3 ➡	TGGGGTGACGTGAAAGATGTCATGTATGATGTTGATGACATCATAGATCTTTTGAGGGCTCACTC
4 ➡	TGGGGTGACGTGAAAGATGTCATGTATGATGTTGATGACATCATAGATCTTTTGAGGGCTCACTC
5 ➡	TGGGGTGACGTGAAAGATGTCATGTATGATGTTGATGACATCATAGATCTTTTGAGGGCTCACTC
6 ➡	TGGGGTGACGTGAAAGATGTCATGTATGATGTTGATGACATCATAGATCTTTTGAGGGCTCACTC
7 ➡	TGGGGTGACGTGAAAGATGTCATGTATGATGTTGATGACATCATAGATCTTTTGAGGGCTCACCC
8 ➡	TGGGGTGACGTGAAAGATGTCATGTATGATGTTGATGACATCATAGATCTTTTGAGGGCTCACTC
9 ➡	TGGGGTGACGTGAAAGATGTCATGTATGATGTTGATGACATCATAGATCTTTTGAGGGCTCACTC
10 ➡	TGGGGTGACGTGAAAGATGTCATGTATGATGTTGATGACATCATAGATCTTTTGAGGGCTCACTC
11 ➡	TGGGGTGACGTGAAAGATGTCATGTATGATGTTGATGACATCATAGATCTTTTGAGGGCTCACTC
12 ➡	TGGGGTGACGTGAAAGATGTCATGTATGATGTTGATGACATCATAGATCTTTTGAGGGCTCACTC
13 ➡	TGGGGTGACGTGAAAGATGTCATGTATGATGTTGATGACATCATAGATCTTTTGAGGGCTCACTC
14 ➡	TGGGGTGACGTGAAAGATGTCATGTATGATGTTGATGACATCATAGATCTTTTGAGGGCTCACTC
15 ➡	TGGGGTGACGTGAAAGATGTCATGTATGATGTTGATGACATCATAGATCTTTTGAGGGCTCACTC
16 ➡	TGGGGTGACGTGAAAGATGTCATGTATGATGTTGATGACATCATAGATCTTTTGAGGGCTCACTC
17 ➡	TGGGGTGACGTGAAAGATGTCATGTATGATGTTGATGACATCATAGATCTTTTGAGGGCTCACTC
18 ➡	TGGGGTGACGTGAAAGATGTCATGTATGATGTTGATGACATCATAGATCTTTTGAGGGCTCACTC
19 ➡	TGGGGTGACGTGAAAGATGTCATGTATGATGTTGATGACATCATAGATCTTTTGAGGGCTCACTC
20 ➡	TGGGGTGACGTGAAAGATGTCATGTATGATGTTGATGACATCATAGATCTTTTGAGGGCTCACTC
21 ➡	TGGGGTGACGTGAAAGATGTCATGTATGATGTTGATGACATCATAGATCTTTTGAGGGCTCACTC
22 ➡	TGGGGTGACGTGAAAGATGTCATGTATGATGTTGATGACATCATAGATCTTTTGAGGGCTCACTC
23 ➡	TGGGGTGACGTGAAAGATGTCATGTATGATGTTGATGACATCATAGATCTTTTGAGGGCTCACTC
24 ➡	TGGGGTGACGTGAAAGATGTCATGTATGATGTTGATGACATCATAGATCTTTTGAGGGCTCACTC
25 ➡	TGGGGTGACGTGAAAGATGTCATGTATGATGTTGATGACATCATAGATCTTTTGAGGGCTCACTC
26 ➡	TGGGGTGACGTGAAAGATGTCATGTATGATGTTGATGACATCATAGATCTTTTGAGGGCTCACTC
27 ➡	TGGGGTGACGTGAAAGATGTCATGTATGATGTTGATGACATCATAGATCTTTTGAGGGCTCACTC
28 ➡	TGGGGTGACGTGAAAGATGTCATGTATGATGTTGATGACATCATAGATCTTTTGAGGGCTCACTC
29 ➡	TGGGGTGACGTGAAAGATGTCATGTATGATGTTGATGACATCATAGATCTTTTGAGGGCTCACTC
30 ➡	TGGGGTGACGTGAAAGATGTCATGTATGATGTTGATGACATCATAGATCTTTTGAGGGCTCACTC
31 ➡	TGGGGTGACGTGAAAGATGTCATGTATGATGTTGATGACATCATAGATCTTTTGAGGGCTCACTC
32 ➡	TGGGGTGACGTGAAAGATGTCATGTATGATGTTGATGACATCATAGATCTTTTGAGGGCTCACTC
33 ➡	TGGGGTGACGTGAAAGATGTCATGTATGATGTTGATGACATCATAGATCTTTTGAGGGCTCACTC
34 ➡	TGGGGTGACGTGAAAGATGTCATGTATGATGTTGATGACATCATAGATCTTTTGAGGGCTCACTC
35 ➡	TGGGGTGACGTGAAAGATGTCATGTATGATGTTGATGACATCATAGATCTTTTGAGGGCTCACTC
36 ➡	TGGGGTGACGTGAAAGATGTCATGTATGATGTTGATGACATCATAGATCTTTTGAGGGCTCACTC
37 ➡	TGGGGTGACGTGAAAGATGTCATGTATGATGTTGATGACATCATAGATCTTTTGAGGGCTCACTC
38 ➡	TGGGGTGACGTGAAAGATGTCATGTATGATGTTGATGACATCATAGATCTTTTGAGGGCTCACTC
39 ➡	TGGGGTGACGTGAAAGATGTCATGTATGATGTTGATGACATCATAGATCTTTTGAGGGCTCACTC
40 ➡	TGGGGTGACGTGAAAGATGTCATGTATGATGTTGATGACATCATAGATCTTTTGAGGGCTCACTC
41 ➡	TGGGGTGACGTGAAAGATGTCATGTATGATGTTGATGACATCATAGATCTTTTGAGGGCTCACTC
42 ➡	TGGGGTGACGTGAAAGATGTCATGTATGATGTTGATGACATCATAGATCTTTTGAGGGCTCACTC

270 280 290 300 310 320

	TCAGAAGCAACGATGTTGTGATTTACGCATGCTCTCTAGGTTTGCACAACTTCAATTTGACCACA
1	TCAGAAGCAACGATGTTGTGATTTACGCATGCTCTCTAGGTTTGCACAACTTCAATTTGACCACA
2	TCAGAAGCAACGATGTTGTGATTTACGCATGCTCTCTAGGTTTGCACAACTTCAATTTGACCACA
3	TCAGAAGCAACGATGTTGTGATTTACGCATGCTCTCTAGGTTTGCACAACTTCAATTTGACCACA
4	TCAGAAGCAACGATGTTGTGATTTACACATGCTCTCTAGGTTTGCACAACTTCAATTTGACCACA
5	TCAGAAGCAACGATGTTGTGATTTACACATGCTCTCTAGGTTTGCACAACTTCAATTTGACCACA
6	TCAGAAGCAACGATGTTGTGATTTACGCATGCTCTCTAGGTTTGCACAACTTCAATTTGACCACA
7	TCAGAAGCAACGATGTTGTGATTTACGCATGCTCTCTAGGTTTGCACAACTTCAATTTGACCACA
8	TCAGAAGCAACGATGTTGTGATTTACGCATGCTCTCTAGGTTTGCACAACTTCAATTTGACCACA
9	TCAGAAGCAACGATGTTGTGATTTACGCATGCTCTCTAGGTTTGCACAACTTCAATTTGACCACA
10	TCAGAAGCAACGATGTTGTGATTTACGCATGCTCTCTAGGTTTGCACAACTTCAATTTGACCACA
11	TCAGAAGCAACGATGTTGTGATTTACGCATGCTCTCTAGGTTTGCACAACTTCAATTTGACCACA
12	TCAGAAGCAACGATGTTGTGATTTACGCATGCTCTCTAGGTTTGCACAACTTCAATTTGACCACA
13	TCAGAAGCAACGATGTTGTGATTTACGCATGCTCTCTAGGTTTGCACAACTTCAATTTGACCACA
14	TCAGAAGCAACGATGTTGTGATTTACGCATGCTCTCTAGGTTTGCACAACTTCAATTTGACCACA
15	TCAGAAGCAACGATGTTGTGATTTACGCATGCTCTCTAGGTTTGCACAACTTCAATTTGACCACA
16	TCAGAAGCAACGATGTTGTGATTTACGCATGCTCTCTAGGTTTGCACAACTTCAATTTGACCACA
17	TCAGAAGCAACGATGTTGTGATTTACGCATGCTCTCTAGGTTTGCACAACTTCAATTTGACCACA
18	TCAGAAGCAACGATGTTGTGATTTACGCATGCTCTCTAGGTTTGCACAACTTCAATTTGACCACA
19	TCAGAAGCAACGATGTTGTGATTTACGCATGCTCTCTAGGTTTGCACAACTTCAATTTGACCACA
20	TCAGAAGCAACGATGTTGTGATTTACGCATGCTCTCTAGGTTTGCACAACTTCAATTTGACCACA
21	TCAGAAGCAACGATGTTGTGATTTACGCATGCTCTCTAGGTTTGCACAACTTCAATTTGACCACA
22	TCAGAAGCAACGATGTTGTGATTTACGCATGCTCTCTAGGTTTGCACAACTTCAATTTGACCACA
23	TCAGAAGCAACGATGTTGTGATTTACGCATGCTCTCTAGGTTTGCACAACTTCAATTTGACCACA
24	TCAGAAGCAACGATGTTGTGATTTACGCATGCTCTCTAGGTTTGCACAACTTCAATTTGACCACA
25	TCAGAAGCAACGATGTTGTGATTTACGCATGCTCTCTAGGTTTGCACAACTTCAATTTGACCACA
26	TCAGAAGCAACGATGTTGTGATTTACGCATGCTCTCTAGGTTTGCACAACTTCAATTTGACCACA
27	TCAGAAGCAACGATGTTGTGATTTACGCATGCTCTCTAGGTTTGCACAACTTCAATTTGACCACA
28	TCAGAAGCAACGATGTTGTGATTTACGCATGCTCTCTAGGTTTGCACAACTTCAATTTGACCACA
29	TCAGAAGCAACGATGTTGTGATTTACGCATGCTCTCTAGGTTTGCACAACTTCAATTTGACCACA
30	TCAGAAGCAACGATGTTGTGATTTACGCATGCTCTCTAGGTTTGCACAACTTCAATTTGACCACA
31	TCAGAAGCAACGATGTTGTGATTTACGCATGCTCTCTAGGTTTGCACAACTTCAATTTGACCACA
32	TCAGAAGCAACGATGTTGTGATTTACGCATGCTCTCTAGGTTTGCACAACTTCAATTTGACCACA
33	TCAGAAGCAACGATGTTGTGATTTACGCATGCTCTCTAGGTTTGCACAACTTCAATTTGACCACA
34	TCAGAAGCAACGATGTTGTGATTTACGCATGCTCTCTAGGTTTGCACAACTTCAATTTGACCACA
35	TCAGAAGCAACGATGTTGTGATTTACGCATGCTCTCTAGGTTTGCACAACTTCAATTTGACCACA
36	TCAGAAGCAACGATGTTGTGATTTACGCATGCTCTCTAGGTTTGCACAACTTCAATTTGACCACA
37	TCAGAAGCAACGATGTTGTGATTTACGCATGCTCTCTAGGTTTGCACAACTTCAATTTGACCACA
38	TCAGAAGCAACGATGTTGTGATTTACGCATGCTCTCTAGGTTTGCACAACTTCAATTTGACCACA
39	TCAGAAGCAACGATGTTGTGATTTACGCATGCTCTCTAGGTTTGCACAACTTCAATTTGACCACA
40	TCAGAAGCAACGATGTTGTGATTTACGCATGCTCTCTAGGTTTGCACAACTTCAATTTGACCACA
41	TCAGAAGCAACGATGTTGTGATTTACGCATGCTCTCTAGGTTTGCACAACTTCAATTTGACCACA
42	TCAGAAGCAACGATGTTGTGATTTACGCATGCTCTCTAGGTTTGCACAACTTCAATTTGACCACA

	330 340 350 360 370 380 390
	TGATTGCCAGAAAAATCAAGGGTGTAAACGAAAGGCTTGTAGAAATCCAAAAGAACAGAGATATG
1 ➡	TGATTGCCAGAAAAATCAAGGGTGTAAACGAAAGGCTTGTAGAAATCCAAAAGAACAGAGATATG
2 ➡	TGATTGCCAGAAAAATCAAGGGTGTAAACGAAAGGCTTGTAGAAATCCAAAAGAACAGAGATATG
3 ➡	TGATTGCCAGAAAAATCAAGGGTGTAAACGAAAGGCTTGTAGAAATCCAAAAGAACAGAGATATG
4 ➡	TGATTGCCAGAAAAATCAAGGGTGTAAACGAAAGGCTTGTAGAAATCCAAAAGAACAGAGATATG
5 ➡	TGATTGCCAGAAAAATCAAGGGTGTAAACGAAAGGCTTGTAGAAATCCAAAAGAACAGAGATATG
6 ➡	TGATTGCCAGAAAAATCAAGGGTGTAAACGAAAGGCTTGTAGAAATCCAAAAGAACAGAGATATG
7 ➡	TGATTGCCAGAAAAATCAAGGGTGTAAACGAAAGGCTTGTAGAAATCCAAAAGAACAGAGATATG
8 ➡	TGATTGCCAGAAAAATCAAGGGTGTAAACGAAAGGCTTGTAGAAATCCAAAAGAACAGAGATATG
9 ➡	TGATTGCCAGAAAAATCAAGGGTGTAAACGAAAGGCTTGTAGAAATCCAAAAGAACAGAGATATG
10 ➡	TGATTGCCAGAAAAATCAAGGGTGTAAACGAAAGGCTTGTAGAAATCCAAAAGAACAGAGATATG
11 ➡	TGATTGCCAGAAAAATCAAGGGTGTAAACGAAAGGCTTGTAGAAATCCAAAAGAACAGAGATATG
12 ➡	TGATTGCCAGAAAAATCAAGGGTGTAAACGAAAGGCTTGTAGAAATCCAAAAGAACAGAGATATG
13 ➡	TGATTGCCAGAAAAATCAAGGGTGTAAACGAAAGGCTTGTAGAAATCCAAAAGAACAGAGATATG
14 ➡	TGATTGCCAGAAAAATCAAGGGTGTAAACGAAAGGCTTGTAGAAATCCAAAAGAACAGAGATATG
15 ➡	TGATTGCCAGAAAAATCAAGGGTGTAAACGAAAGGCTTGTAGAAATCCAAAAGAACAGAGATATG
16 ➡	TGATTGCCAGAAAAATCAAGGGTGTAAACGAAAGGCTTGTAGAAATCCAAAAGAACAGAGATATG
17 ➡	TGATTGCCAGAAAAATCAAGGGTGTAAACGAAAGGCTTGTAGAAATCCAAAAGAACAGAGATATG
18 ➡	TGATTGCCAGAAAAATCAAGGGTGTAAACGAAAGGCTTGTAGAAATCCAAAAGAACAGAGATATG
19 ➡	TGATTGCCAGAAAAATCAAGGGTGTAAACGAAAGGCTTGTAGAAATCCAAAAGAACAGAGATATG
20 ➡	TGATTGCCAGAAAAATCAAGGGTGTAAACGAAAGGCTTGTAGAAATCCAAAAGAACAGAGATATG
21 ➡	TGATTGCCAGAAAAATCAAGGGTGTAAACGAAAGGCTTGTAGAAATCCAAAAGAACAGAGATATG
22 ➡	TGATTGCCAGAAAAATCAAGGGTGTAAACGAAAGGCTTGTAGAAATCCAAAAGAACAGAGATATG
23 ➡	TGATTGCCAGAAAAATCAAGGGTGTAAACGAAAGGCTTGTAGAAATCCAAAAGAACAGAGATATG
24 ➡	TGATTGCCAGAAAAATCAAGGGTGTAAACGAAAGGCTTGTAGAAATCCAAAAGAACAGAGATATG
25 ➡	TGATTGCCAGAAAAATCAAGGGTGTAAACGAAAGGCTTGTAGAAATCCAAAAGAACAGAGATATG
26 ➡	TGATTGCCAGAAAAATCAAGGGTGTAAACGAAAGGCTTGTAGAAATCCAAAAGAACAGAGATATG
27 ➡	TGATTGCCAGAAAAATCAAGGGTGTAAACGAAAGGCTTGTAGAAATCCAAAAGAACAGAGATATG
28 ➡	TGATTGCCAGAAAAATCAAGGGTGTAAACGAAAGGCTTGTAGAAATCCAAAAGAACAGAGATATG
29 ➡	TGATTGCCAGAAAAATCAAGGGTGTAAACGAAAGGCTTGTAGAAATCCAAAAGAACAGAGATATG
30 ➡	TGATTGCCAGAAAAATCAAGGGTGTAAACGAAAGGCTTGTAGAAATCCAAAAGAACAGAGATATG
31 ➡	TGATTGCCAGAAAAATCAAGGGTGTAAACGAAAGGCTTGTAGAAATCCAAAAGAACAGAGATATG
32 ➡	TGATTGCCAGAAAAATCAAGGGTGTAAACGAAAGGCTTGTAGAAATCCAAAAGAACAGAGATATG
33 ➡	TGATTGCCAGAAAAATCAAGGGTGTAAACGAAAGGCTTGTAGAAATCCAAAAGAACAGAGATATG
34 ➡	TGATTGCCAGAAAAATCAAGGGTGTAAACGAAAGGCTTGTAGAAATCCAAAAGAACAGAGATATG
35 ➡	TGATTGCCAGAAAAATCAAGGGTGTAAACGAAAGGCTTGTAGAAATCCAAAAGAACAGAGATATG
36 ➡	TGATTGCCAGAAAAATCAAGGGTGTAAACGAAAGGCTTGTAGAAATCCAAAAGAACAGAGATATG
37 ➡	TGATTGCCAGAAAAATCAAGGGTGTAAACGAAAGGCTTGTAGAAATCCAAAAGAACAGAGATATG
38 ➡	TGATTGCCAGAAAAATCAAGGGTGTAAACGAAAGGCTTGTAGAAATCCAAAAGAACAGAGATATG
39 ➡	TGATTGCCAGAAAAATCAAGGGTGTAAACGAAAGGCTTGTAGAAATCCAAAAGAACAGAGATATG
40 ➡	TGATTGCCAGAAAAATCAAGGGTGTAAACGAAAGGCTTGTAGAAATCCAAAAGAACAGAGATATG
41 ➡	TGATTGCCAGAAAAATCAAGGGTGTAAACGAAAGGCTTGTAGAAATCCAAAAGAACAGAGATATG
42 ➡	TGATTGCCAGAAAAATCAAGGGTGTAAACGAAAGGCTTGTAGAAATCCAAAAGAACAGAGATATG

```
     400        410        420        430        440        450
     TTCCTTCCTCCGGGGTTGTATCCTCAGGCTCAGGCCCCTCAAACAAACGGAGTTGATAGCAGGCA
 1 ➨ TTCCTTCCTCCGGGGTTGTATCCTCAGGCTCAGGCCCCTCAAACAAACAGAGTTGATAGCAGGCA
 2 ➨ TTCCTTCCTCCGGGGTTGTATCCTCAGGCTCAGGCCCCTCAAACAAACAGAGTTGATAGCAGGCA
 3 ➨ TTCCTTCCTCCGGGGTTGTATCCTCAGGCTCAGGCCCCTCAAACAAACAGAGTTGATAGCAGGCA
 4 ➨ TTCCTTCCTCCGGGGTTGTATCCTCAGGCACAGGCCCCTCAAACAAACAGAGTTGATAGCAGGCA
 5 ➨ TTCCTTCCTCCGGGGTTGTATCCTCAGGCACAGGCCCCTCAAACAAACAGAGTTGATAGCAGGCA
 6 ➨ TTCCTTCCTCCGGGGTTGTATCCTCAGGCTCAGGCCCCTCAAACAAACGGAGTTGATAGCAGGCA
 7 ➨ TTCCTTCCTCCGGGGTTGTATCCTCAGGCTCAGGCCCCTCAAACAAACAGAGTTGATAGCAGGCA
 8 ➨ TTCCTTCCTCCGGGGTTGTATCCTCAGGCTCAGGCCCCTCAAACAAACGGAGTTGATAGCAGGCA
 9 ➨ TTCCTTCCTCCGGGGTTGTATCCTCAGGCTCAGGCCCCTCAAACAAACGGAGTTGATAGCAGGCA
10 ➨ TTCCTTCCTCCGGGGTTGTATCCTCAGGCTCAGGCCCCTCAAACAAACGGAGTTGATAGCAGGCA
11 ➨ TTCCTTCCTCCGGGGTTGTATCCTCAGGCTCAGGCCCCTCAAACAAACGGAGTTGATAGCAGGCA
12 ➨ TTCCTTCCTCCGGGGTTGTATCCTCAGGCTCAGGCCCCTCAAACAAACGGAGTTGATAGCAGGCA
13 ➨ TTCCTTCCTCCGGGGTTGTATCCTCAGGCTCAGGCCCCTCAAACAAACGGAGTTGATAGCAGGCA
14 ➨ TTCCTTCCTCCGGGGTTGTATCCTCAGGCTCAGGCCCCTCAAACAAACGGAGTTGATAGCAGGCA
15 ➨ TTCCTTCCTCCGGGGTTGTATCCTCAGGCTCAGGCCCCTCAAACAAACGGAGTTGATAGCAGGCA
16 ➨ TTCCTTCCTCCGGGGTTGTATCCTCAGGCTCAGGCCCCTCAAACAAACGGAGTTGATAGCAGGCA
17 ➨ TTCCTTCCTCCGGGGTTGTATCCTCAGGCTCAGGCCCCTCAAACAAACGGAGTTGATAGCAGGCA
18 ➨ TTCCTTCCTCCGGGGTTGTATCCTCAGGCTCAGGCCCCTCAAACAAACGGAGTTGATAGCAGGCA
19 ➨ TTCCTTCCTCCGGGGTTGTATCCTCAGGCTCAGGCCCCTCAAACAAACGGAGTTGATAGCAGGCA
20 ➨ TTCCTTCCTCCGGGGTTGTATCCTCAGGCTCAGGCCCCTCAAACAAACGGAGTTGATAGCAGGCA
21 ➨ TTCCTTCCTCCGGGGTTGTATCCTCAGGCTCAGGCCCCTCAAACAAACGGAGTTGATAGCAGGCA
22 ➨ TTCCTTCCTCCGGGGTTGTATCCTCAGGCTCAGGCCCCTCAAACAAACGGAGTTGATAGCAGGCA
23 ➨ TTCCTTCCTCCGGGGTTGTATCCTCAGGCTCAGGCCCCTCAAACAAACGGAGTTGATAGCAGGCA
24 ➨ TTCCTTCCTCCGGGGTTGTATCCTCAGGCTCAGGCCCCTCAAACAAACGGAGTTGATAGCAGGCA
25 ➨ TTCCTTCCTCCGGGGTTGTATCCTCAGGCTCAGGCCCCTCAAACAAACGGAGTTGATAGCAGGCA
26 ➨ TTCCTTCCTCCGGGGTTGTATCCTCAGGCTCAGGCCCCTCAAACAAACGGAGTTGATAGCAGGCA
27 ➨ TTCCTTCCTCCGGGGTTGTATCCTCAGGCTCAGGCCCCTCAAACAAACGGAGTTGATAGCAGGCA
28 ➨ TTCCTTCCTCCGGGGTTGTATCCTCAGGCTCAGGCCCCTCAAACAAACGGAGTTGATAGCAGGCA
29 ➨ TTCCTTCCTCCGGGGTTGTATCCTCAGGCTCAGGCCCCTCAAACAAACGGAGTTGATAGCAGGCA
30 ➨ TTCCTTCCTCCGGGGTTGTATCCTCAGGCTCAGGCCCCTCAAACAAACGGAGTTGATAGCAGGCA
31 ➨ TTCCTTCCTCCGGGGTTGTATCCTCAGGCTCAGGCCCCTCAAACAAACGGAGTTGATAGCAGGCA
32 ➨ TTCCTTCCTCCGGGGTTGTATCCTCAGGCTCAGGCCCCTCAAACAAACGGAGTTGATAGCAGGCA
33 ➨ TTCCTTCCTCCGGGGTTGTATCCTCAGGCTCAGGCCCCTCAAACAAACGGAGTTGATAGCAGGCA
34 ➨ TTCCTTCCTCCGGGGTTGTATCCTCAGGCTCAGGCCCCTCAAACAAACGGAGTTGATAGCAGGCA
35 ➨ TTCCTTCCTCCGGGGTTGTATCCTCAGGCTCAGGCCCCTCAAACAAACGGAGTTGATAGCAGGCA
36 ➨ TTCCTTCCTCCGGGGTTGTATCCTCAGGCTCAGGCCCCTCAAACAAACGGAGTTGATAGCAGGCA
37 ➨ TTCCTTCCTCCGGGGTTGTATCCTCAGGCTCAGGCCCCTCAAACAAACGGAGTTGATAGCAGGCA
38 ➨ TTCCTTCCTCCGGGGTTGTATCCTCAGGCTCAGGCCCCTCAAACAAACGGAGTTGATAGCAGGCA
39 ➨ TTCCTTCCTCCGGGGTTGTATCCTCAGGCTCAGGCCCCTCAAACAAACGGAGTTGATAGCAGGCA
40 ➨ TTCCTTCCTCCGGGGTTGTATCCTCAGGCTCAGGCCCCTCAAACAAACGGAGTTGATAGCAGGCA
41 ➨ TTCCTTCCTCCGGGGTTGTATCCTCAGGCTCAGGCCCCTCAAACAAACGGAGTTGATAGCAGGCA
42 ➨ TTCCTTCCTCCGGGGTTGTATCCTCAGGCTCAGGCCCCTCAAACAAACGGAGTTGATAGCAGGCA
```

	460 470 480 490 500 510 520
	TCTGGCTGCTTCGGTTGATGAAATCCATGTTGTTGGAGCAGAAATCAAGGAAGCTACTGATAGTA
1 ➨	TCTGGCTGCTTCGGTTGATGAAATCCATGTTGTTGGAGCAGAAATCAAGGAAGCTACTGATAGTA
2 ➨	TCTGGCTGCTTCGGTTGATGAAATCCATGTTGTTGGAGCAGAAATCAAGGAAGCTACTGATAGTA
3 ➨	TCTGGCTGCTTCGGTTGATGAAATCCATGTTGTTGGAGCAGAAATCAAGGAAGCTACTGATAGTA
4 ➨	TCTAGCTGCTTCGGTTGATGAAATCCATGTTGTTGGAGCAGAAATCAAGGAAGCTACTGATAGTA
5 ➨	TCTAGCTGCTTCGGTTGATGAAATCCATGTTGTTGGAGCAGAAATCAAGGAAGCTACTGATAGTA
6 ➨	TCTGGCTGCTTCGGTTGATGAAATCCATGTTGTTGGAGCAGAAATCAAGGAAGCTACTGATAGTA
7 ➨	TCTGGCTGCTTCGGTTGATGAAATCCATGTTGTTGGAGCAGAAATCAAGGAAGCTACTGATAGTA
8 ➨	TCTGGCTGCTTCGGTTGATGAAATCCATGTTGTTGGAGCAGAAATCAAGGAAGCTACTGATAGTA
9 ➨	TCTGGCTGCTTCGGTTGATGAAATCCATGTTGTTGGAGCAGAAATCAAGGAAGCTACTGATAGTA
10 ➨	TCTGGCTGCTTCGGTTGATGAAATCCATGTTGTTGGAGCAGAAATCAAGGAAGCTACTGATAGTA
11 ➨	TCTGGCTGCTTCGGTTGATGAAATCCATGTTGTTGGAGCAGAAATCAAGGAAGCTACTGATAGTA
12 ➨	TCTGGCTGCTTCGGTTGATGAAATCCATGTTGTTGGAGCAGAAATCAAGGAAGCTACTGATAGTA
13 ➨	TCTGGCTGCTTCGGTTGATGAAATCCATGTTGTTGGAGCAGAAATCAAGGAAGCTACTGATAGTA
14 ➨	TCTGGCTGCTTCGGTTGATGAAATCCATGTTGTTGGAGCAGAAATCAAGGAAGCTACTGATAGTA
15 ➨	TCTGGCTGCTTCGGTTGATGAAATCCATGTTGTTGGAGCAGAAATCAAGGAAGCTACTGATAGTA
16 ➨	TCTGGCTGCTTCGGTTGATGAAATCCATGTTGTTGGAGCAGAAATCAAGGAAGCTACTGATAGTA
17 ➨	TCTGGCTGCTTCGGTTGATGAAATCCATGTTGTTGGAGCAGAAATCAAGGAAGCTACTGATAGTA
18 ➨	TCTGGCTGCTTCGGTTGATGAAATCCATGTTGTTGGAGCAGAAATCAAGGAAGCTACTGATAGTA
19 ➨	TCTGGCTGCTTCGGTTGATGAAATCCATGTTGTTGGAGCAGAAATCAAGGAAGCTACTGATAGTA
20 ➨	TCTGGCTGCTTCGGTTGATGAAATCCATGTTGTTGGAGCAGAAATCAAGGAAGCTACTGATAGTA
21 ➨	TCTGGCTGCTTCGGTTGATGAAATCCATGTTGTTGGAGCAGAAATCAAGGAAGCTACTGATAGTA
22 ➨	TCTGGCTGCTTCGGTTGATGAAATCCATGTTGTTGGAGCAGAAATCAAGGAAGCTACTGATAGTA
23 ➨	TCTGGCTGCTTCGGTTGATGAAATCCATGTTGTTGGAGCAGAAATCAAGGAAGCTACTGATAGTA
24 ➨	TCTGGCTGCTTCGGTTGATGAAATCCATGTTGTTGGAGCAGAAATCAAGGAAGCTACTGATAGTA
25 ➨	TCTGGCTGCTTCGGTTGATGAAATCCATGTTGTTGGAGCAGAAATCAAGGAAGCTACTGATAGTA
26 ➨	TCTGGCTGCTTCGGTTGATGAAATCCATGTTGTTGGAGCAGAAATCAAGGAAGCTACTGATAGTA
27 ➨	TCTGGCTGCTTCGGTTGATGAAATCCATGTTGTTGGAGCAGAAATCAAGGAAGCTACTGATAGTA
28 ➨	TCTGGCTGCTTCGGTTGATGAAATCCATGTTGTTGGAGCAGAAATCAAGGAAGCTACTGATAGTA
29 ➨	TCTGGCTGCTTCGGTTGATGAAATCCATGTTGTTGGAGCAGAAATCAAGGAAGCTACTGATAGTA
30 ➨	TCTGGCTGCTTCGGTTGATGAAATCCATGTTGTTGGAGCAGAAATCAAGGAAGCTACTGATAGTA
31 ➨	TCTGGCTGCTTCGGTTGATGAAATCCATGTTGTTGGAGCAGAAATCAAGGAAGCTACTGATAGTA
32 ➨	TCTGGCTGCTTCGGTTGATGAAATCCATGTTGTTGGAGCAGAAATCAAGGAAGCTACTGATAGTA
33 ➨	TCTGGCTGCTTCGGTTGATGAAATCCATGTTGTTGGAGCAGAAATCAAGGAAGCTACTGATAGTA
34 ➨	TCTGGCTGCTTCGGTTGATGAAATCCATGTTGTTGGAGCAGAAATCAAGGAAGCTACTGATAGTA
35 ➨	TCTGGCTGCTTCGGTTGATGAAATCCATGTTGTTGGAGCAGAAATCAAGGAAGCTACTGATAGTA
36 ➨	TCTGGCTGCTTCGGTTGATGAAATCCATGTTGTTGGAGCAGAAATCAAGGAAGCTACTGATAGTA
37 ➨	TCTGGCTGCTTCGGTTGATGAAATCCATGTTGTTGGAGCAGAAATCAAGGAAGCTACTGATAGTA
38 ➨	TCTGGCTGCTTCGGTTGATGAAATCCATGTTGTTGGAGCAGAAATCAAGGAAGCTACTGATAGTA
39 ➨	TCTGGCTGCTTCGGTTGATGAAATCCATGTTGTTGGAGCAGAAATCAAGGAAGCTACTGATAGTA
40 ➨	TCTGGCTGCTTCGGTTGATGAAATCCATGTTGTTGGAGCAGAAATCAAGGAAGCTACTGATAGTA
41 ➨	TCTGGCTGCTTCGGTTGATGAAATCCATGTTGTTGGAGCAGAAATCAAGGAAGCTACTGATAGTA
42 ➨	TCTGGCTGCTTCGGTTGATGAAATCCATGTTGTTGGAGCAGAAATCAAGGAAGCTACTGATAGTA

	530 540 550 560 570 580
	TGGTAGAAATGATTGTCGGTTATGGCCATCAGAGCAGGATATCAGTTTATGGGATCGTTGGGATG
1 ➨	TGGTAGAAATGATCGTCGGCTATGGCCATCAGAGCAGGATATCAGTTTATGGGATCGTTGGGATG
2 ➨	TGGTAGAAATGATCGTCGGCTATGGCCATCAGAGCAGGATATCAGTTTATGGGATCGTTGGGATG
3 ➨	TGGTAGAAATGATCGTCGGCTATGGCCATCAGAGCAGGATATCAGTTTATGGGATCGTTGGGATG
4 ➨	TGGTAGAAATGATCGTCGGCTATGGCCATCAGAGTAGGATATCAGTTTATGGGATCCTTGGGATG
5 ➨	TGGTAGAAATGATCGTCGGCTATGGCCATCAGAGTAGGATATCAGTTTATGGGATCCTTGGGATG
6 ➨	TGGTAGAAATGATTGTCGGTTATGGCCATCAGAGCAGGATATCAGTTTATGGGATCGTTGGGATG
7 ➨	TGGTAGAAATGATCGTCGGCTATGGCCATCAGAGCAGGATATCAGTTTATGGGATCGTTGGGATG
8 ➨	TGGTAGAAATGATTGTCGGTTATGGCCATCAGAGCAGGATATCAGTTTATGGGATCGTTGGGATG
9 ➨	TGGTAGAAATGATTGTCGGTTATGGCCATCAGAGCAGGATATCAGTTTATGGGATCGTTGGGATG
10 ➨	TGGTAGAAATGATTGTCGGTTATGGCCATCAGAGCAGGATATCAGTTTATGGGATCGTTGGGATG
11 ➨	TGGTAGAAATGATTGTCGGTTATGGCCATCAGAGCAGGATATCAGTTTGTGGGATCGTTGGGATG
12 ➨	TGGTAGAAATGATTGTCGGTTATGGCCATCAGAGCAGGATATCAGTTTGTGGGATCGTTGGGATG
13 ➨	TGGTAGAAATGATTGTCGGTTATGGCCATCAGAGCAGGATATCAGTTTGTGGGATCGTTGGGATG
14 ➨	TGGTAGAAATGATTGTCGGTTATGGCCATCAGAGCAGGATATCAGTTTATGGGATCGTTGGGATG
15 ➨	TGGTAGAAATGATTGTCGGTTATGGCCATCAGAGCAGGATATCAGTTTATGGGATCGTTGGGATG
16 ➨	TGGTAGAAATGATTGTCGGTTATGGCCATCAGAGCAGGATATCAGTTTATGGGATCGTTGGGATG
17 ➨	TGGTAGAAATGATTGTCGGTTATGGCCATCAGAGCAGGATATCAGTTTATGGGATCGTTGGGATG
18 ➨	TGGTAGAAATGATTGTCGGTTATGGCCATCAGAGCAGGATATCAGTTTATGGGATCGTTGGGATG
19 ➨	TGGTAGAAATGATTGTCGGTTATGGCCATCAGAGCAGGATATCAGTTTATGGGATCGTTGGGATG
20 ➨	TGGTAGAAATGATTGTCGGTTATGGCCATCAGAGCAGGATATCAGTTTATGGGATCGTTGGGATG
21 ➨	TGGTAGAAATGATTGTCGGTTATGGCCATCAGAGCAGGATATCAGTTTATGGGATCGTTGGGATG
22 ➨	TGGTAGAAATGATTGTCGGTTATGGCCATCAGAGCAGGATATCAGTTTATGGGATCGTTGGGATG
23 ➨	TGGTAGAAATGATTGTCGGTTATGGCCATCAGAGCAGGATATCAGTTTATGGGATCGTTGGGATG
24 ➨	TGGTAGAAATGATTGTCGGTTATGGCCATCAGAGCAGGATATCAGTTTATGGGATCGTTGGGATG
25 ➨	TGGTAGAAATGATTGTCGGTTATGGCCATCAGAGCAGGATATCAGTTTATGGGATCGTTGGGATG
26 ➨	TGGTAGAAATGATTGTCGGTTATGGCCATCAGAGCAGGATATCAGTTTATGGGATCGTTGGGATG
27 ➨	TGGTAGAAATGATTGTCGGTTATGGCCATCAGAGCAGGATATCAGTTTATGGGATCGTTGGGATG
28 ➨	TGGTAGAAATGATTGTCGGTTATGGCCATCAGAGCAGGATATCAGTTTATGGGATCGTTGGGATG
29 ➨	TGGTAGAAATGATTGTCGGTTATGGCCATCAGAGCAGGATATCAGTTTATGGGATCGTTGGGATG
30 ➨	TGGTAGAAATGATTGTCGGTTATGGCCATCAGAGCAGGATATCAGTTTATGGGATCGTTGGGATG
31 ➨	TGGTAGAAATGATTGTCGGTTATGGCCATCAGAGCAGGATATCAGTTTATGGGATCGTTGGGATG
32 ➨	TGGTAGAAATGATTGTCGGTTATGGCCATCAGAGCAGGATATCAGTTTATGGGATCGTTGGGATG
33 ➨	TGGTAGAAATGATTGTCGGTTATGGCCATCAGAGCAGGATATCAGTTTATGGGATCGTTGGGATG
34 ➨	TGGTAGAAATGATTGTCGGTTATGGCCATCAGAGCAGGATATCAGTTTATGGGATCGTTGGGATG
35 ➨	TGGTAGAAATGATTGTCGGTTATGGCCATCAGAGCAGGATATCAGTTTATGGGATCGTTGGGATG
36 ➨	TGGTAGAAATGATTGTCGGTTATGGCCATCAGAGCAGGATATCAGTTTATGGGATCGTTGGGATG
37 ➨	TGGTAGAAATGATTGTCGGTTATGGCCATCAGAGCAGGATATCAGTTTATGGGATCGTTGGGATG
38 ➨	TGGTAGAAATGATTGTCGGTTATGGCCATCAGAGCAGGATATCAGTTTATGGGATCGTTGGGATG
39 ➨	TGGTAGAAATGATTGTCGGTTATGGCCATCAGAGCAGGATATCAGTTTATGGGATCGTTGGGATG
40 ➨	TGGTAGAAATGATTGTCGGTTATGGCCATCAGAGCAGGATATCAGTTTATGGGATCGTTGGGATG
41 ➨	TGGTAGAAATGATTGTCGGTTATGGCCATCAGAGCAGGATATCAGTTTATGGGATCGTTGGGATG
42 ➨	TGGTAGAAATGATTGTCGGTTATGGCCATCAGAGCAGGATATCAGTTTATGGGATCGTTGGGATG

590 600 610 620 630 640 650

	GGAGGAATTGGTAAGACAACTTTAGCTCAGAAGATATACAATGATCGTAGGATAAGAGAGAGATT
1	GGAGGAATTGGCAAGACAACTTTAGCTCAGAAGATATACAATGATCGTAGGATAAGAGAGAGATT
2	GGAGGAATTGGCAAGACAACTTTAGCTCAGAAGATATACAATGATCGTAGGATAAGAGAGAGATT
3	GGAGGAATTGGCAAGACAACTTTAGCTCAGAAGATATACAATGATCGTAGGATAAGAGAGAGATT
4	GGAGGAATTGGCAAGACAACTTTAGCTCAAAAGATATACAATGATCGTAGGATAAGAGAGAGATT
5	GGAGGAATTGGCAAGACAACTTTAGCTCAAAAGATATACAATGATCGTAGGATAAGAGAGAGATT
6	GGAGGAATTGGTAAGACAACTTTAGCTCAGAAGATATACAATGATCGTAGGATAAGAGAGAGATT
7	GGAGGAATTGGCAAGACAACTTTAGCTCAGAAGATATACAATGATTGTAGGATAAGAGAGAGATT
8	GGAGGAATTGGTAAGACAACTTTAGCTCAGAAGATATACAATGATCGTAGGATAAGAGAGAGATT
9	GGAGGAATTGGTAAGACAACTTTAGCTCAGAAGATATACAATGATCGTAGGATAAGAGAGAGATT
10	GGAGGAATTGGTAAGACAACTTTAGCTCAGAAGATATACAATGATCGTAGGATAAGAGAGAGATT
11	GGAGGAATTGGTAAGACAACTTTAGCTCAGAAGATATACAATGATCGTAGGATAAGAGAGAGATT
12	GGAGGAATTGGTAAGACAACTTTAGCTCAGAAGATATACAATGATCGTAGGATAAGAGAGAGATT
13	GGAGGAATTGGTAAGACAACTTTAGCTCAGAAGATATACAATGATCGTAGGATAAGAGAGAGATT
14	GGAGGAATTGGTAAGACAACTTTAGCTCAGAAGATATACAATGATCGTAGGATAAGAGAGAGATT
15	GGAGGAATTGGTAAGACAACTTTAGCTCAGAAGATATACAATGATCGTAGGATAAGAGAGAGATT
16	GGAGGAATTGGTAAGACAACTTTAGCTCAGAAGATATACAATGATCGTAGGATAAGAGAGAGATT
17	GGAGGAATTGGTAAGACAACTTTAGCTCAGAAGATATACAATGATCGTAGGATAAGAGAGAGATT
18	GGAGGAATTGGTAAGACAACTTTAGCTCAGAAGATATACAATGATCGTAGGATAAGAGAGAGATT
19	GGAGGAATTGGTAAGACAACTTTAGCTCAGAAGATATACAATGATCGTAGGATAAGAGAGAGATT
20	GGAGGAATTGGTAAGACAACTTTAGCTCAGAAGATATACAATGATCGTAGGATAAGAGAGAGATT
21	GGAGGAATTGGTAAGACAACTTTAGCTCAGAAGATATACAATGATCGTAGGATAAGAGAGAGATT
22	GGAGGAATTGGTAAGACAACTTTAGCTCAGAAGATATACAATGATCGTAGGATAAGAGAGAGATT
23	GGAGGAATTGGTAAGACAACTTTAGCTCAGAAGATATACAATGATCGTAGGATAAGAGAGAGATT
24	GGAGGAATTGGTAAGACAACTTTAGCTCAGAAGATATACAATGATCGTAGGATAAGAGAGAGATT
25	GGAGGAATTGGTAAGACAACTTTAGCTCAGAAGATATACAATGATCGTAGGATAAGAGAGAGATT
26	GGAGGAATTGGTAAGACAACTTTAGCTCAGAAGATATACAATGATCGTAGGATAAGAGAGAGATT
27	GGAGGAATTGGTAAGACAACTTTAGCTCAGAAGATATACAATGATCGTAGGATAAGAGAGAGATT
28	GGAGGAATTGGTAAGACAACTTTAGCTCAGAAGATATACAATGATCGTAGGATAAGAGAGAGATT
29	GGAGGAATTGGTAAGACAACTTTAGCTCAGAAGATATACAATGATCGTAGGATAAGAGAGAGATT
30	GGAGGAATTGGTAAGACAACTTTAGCTCAGAAGATATACAATGATCGTAGGATAAGAGAGAGATT
31	GGAGGAATTGGTAAGACAACTTTAGCTCAGAAGATATACAATGATCGTAGGATAAGAGAGAGATT
32	GGAGGAATTGGTAAGACAACTTTAGCTCAGAAGATATACAATGATCGTAGGATAAGAGAGAGATT
33	GGAGGAATTGGTAAGACAACTTTAGCTCAGAAGATATACAATGATCGTAGGATAAGAGAGAGATT
34	GGAGGAATTGGTAAGACAACTTTAGCTCAGAAGATATACAATGATCGTAGGATAAGAGAGAGATT
35	GGAGGAATTGGTAAGACAACTTTAGCTCAGAAGATATACAATGATCGTAGGATAAGAGAGAGATT
36	GGAGGAATTGGTAAGACAACTTTAGCTCAGAAGATATACAATGATCGTAGGATAAGAGAGAGATT
37	GGAGGAATTGGTAAGACAACTTTAGCTCAGAAGATATACAATGATCGTAGGATAAGAGAGAGATT
38	GGAGGAATTGGTAAGACAACTTTAGCTCAGAAGATATACAATGATCGTAGGATAAGAGAGAGATT
39	GGAGGAATTGGTAAGACAACTTTAGCTCAGAAGATATACAATGATCGTAGGATAAGAGAGAGATT
40	GGAGGAATTGGTAAGACAACTTTAGCTCAGAAGATATACAATGATCGTAGGATAAGAGAGAGATT
41	GGAGGAATTGGTAAGACAACTTTAGCTCAGAAGATATACAATGATCGTAGGATAAGAGAGAGATT
42	GGAGGAATTGGTAAGACAACTTTAGCTCAGAAGATATACAATGATCGTAGGATAAGAGAGAGATT

	660 670 680 690 700 710
	TCACCAGGTCCTCATCTGGCTGTCCATTTCACAGAGCATTGCCGAGAATGATCTACTCAAGGAGG
1	TCACCAGGTCCTCATCTGGCTGTCCATTTCACAGAGCATTGCCGAGAATGATCTACTCAAGGAGG
2	TCACCAGGTCCTCATCTGGCTGTCCATTTCACAGAGCATTGCCGAGAATGATCTACTCAAGGAGG
3	TCACCAGGTCCTCATCTGGCTGTCCATTTCACAGAGCATTGCCGAGAATGATCTACTCAAGGAGG
4	TCACCAGGCCCTCATCTGGCTGTCCATTCCACATAGCATTGCCGAGAATGATCTACTCAAGGAGG
5	TCACCAGGCCCTCATCTGGCTGTCCATTCCACATAGCATTGCCGAGAATGATCTACTCAAGGAGG
6	TCACCAGGTCCTCATCTGGCTGTCCATTTCACAGAGCATTGCCGAGAATGATCTACTCAAGGAGG
7	TCACCAGGTCCTCATCTGGCTGTCCATTTCACAGAGCATTGCCGAGAATGATCTACTCAAGGAGG
8	TCACCAGGTCCTCATCTGGCTGTCCATTTCACAGAGCATTGCCGAGAATGATCTACTCAAGGAGG
9	TCACCAGGTCCTCATCTGGCTGTCCATTTCACAGAGCATTGCCGAGAATGATCTACTCAAGGAGG
10	TCACCAGGTCCTCATCTGGCTGTCCATTTCACAGAGCATTGCCGAGAATGATCTACTCAAGGAGG
11	TCACCAGGTCCTCATCTGGCTGTCCATTTCACAGGGCATTGCCGAGAATGATCTACTCAAGGAGG
12	TCACCAGGTCCTCATCTGGCTGTCCATTTCACAGGGCATTGCCGAGAATGATCTACTCAAGGAGG
13	TCACCAGGTCCTCATCTGGCTGTCCATTTCACAGGGCATTGCCGAGAATGATCTACTCAAGGAGG
14	TCACCAGGTCCTCATCTGGCTGTCCATTTCACAGAGCATTGCCGAGAATGATCTACTCAAGGAGG
15	TCACCAGGTCCTCATCTGGCTGTCCATTTCACAGAGCATTGCCGAGAATGATCTACTCAAGGAGG
16	TCACCAGGTCCTCATCTGGCTGTCCATTTCACAGAGCATTGCCGAGAATGATCTACTCAAGGAGG
17	TCACCAGGTCCTCATCTGGCTGTCCATTTCACAGAGCATTGCCGAGAATGATCTACTCAAGGAGG
18	TCACCAGGTCCTCATCTGGCTGTCCATTTCACAGAGCATTGCCGAGAATGATCTACTCAAGGAGG
19	TCACCAGGTCCTCATCTGGCTGTCCATTTCACAGAGCATTGCCGAGAATGATCTACTCAAGGAGG
20	TCACCAGGTCCTCATCTGGCTGTCCATTTCACAGAGCATTGCCGAGAATGATCTACTCAAGGAGG
21	TCACCAGGTCCTCATCTGGCTGTCCATTTCACAGAGCATTGCCGAGAATGATCTACTCAAGGAGG
22	TCACCAGGTCCTCATCTGGCTGTCCATTTCACAGAGCATTGCCGAGAATGATCTACTCAAGGAGG
23	TCACCAGGTCCTCATCTGGCTGTCCATTTCACAGAGCATTGCCGAGAATGATCTACTCAAGGAGG
24	TCACCAGGTCCTCATCTGGCTGTCCATTTCACAGAGCATTGCCGAGAATGATCTACTCAAGGAGG
25	TCACCAGGTCCTCATCTGGCTGTCCATTTCACAGAGCATTGCCGAGAATGATCTACTCAAGGAGG
26	TCACCAGGTCCTCATCTGGCTGTCCATTTCACAGAGCATTGCCGAGAATGATCTACTCAAGGAGG
27	TCACCAGGTCCTCATCTGGCTGTCCATTTCACAGAGCATTGCCGAGAATGATCTACTCAAGGAGG
28	TCACCAGGTCCTCATCTGGCTGTCCATTTCACAGAGCATTGCCGAGAATGATCTACTCAAGGAGG
29	TCACCAGGTCCTCATCTGGCTGTCCATTTCACAGAGCATTGCCGAGAATGATCTACTCAAGGAGG
30	TCACCAGGTCCTCATCTGGCTGTCCATTTCACAGAGCATTGCCGAGAATGATCTACTCAAGGAGG
31	TCACCAGGTCCTCATCTGGCTGTCCATTTCACAGAGCATTGCCGAGAATGATCTACTCAAGGAGG
32	TCACCAGGTCCTCATCTGGCTGTCCATTTCACAGAGCATTGCCGAGAATGATCTACTCAAGGAGG
33	TCACCAGGTCCTCATCTGGCTGTCCATTTCACAGAGCATTGCCGAGAATGATCTACTCAAGGAGG
34	TCACCAGGTCCTCATCTGGCTGTCCATTTCACAGAGCATTGCCGAGAATGATCTACTCAAGGAGG
35	TCACCAGGTCCTCATCTGGCTGTCCATTTCACAGAGCATTGCCGAGAATGATCTACTCAAGGAGG
36	TCACCAGGTCCTCATCTGGCTGTCCATTTCACAGAGCATTGCCGAGAATGATCTACTCAAGGAGG
37	TCACCAGGTCCTCATCTGGCTGTCCATTTCACAGAGCATTGCCGAGAATGATCTACTCAAGGAGG
38	TCACCAGGTCCTCATCTGGCTGTCCATTTCACAGAGCATTGCCGAGAATGATCTACTCAAGGAGG
39	TCACCAGGTCCTCATCTGGCTGTCCATTTCACAGAGCATTGCCGAGAATGATCTACTCAAGGAGG
40	TCACCAGGTCCTCATCTGGCTGTCCATTTCACAGAGCATTGCCGAGAATGATCTACTCAAGGAGG
41	TCACCAGGTCCTCATCTGGCTGTCCATTTCACAGAGCATTGCCGAGAATGATCTACTCAAGGAGG
42	TCACCAGGTCCTCATCTGGCTGTCCATTTCACAGAGCATTGCCGAGAATGATCTACTCAAGGAGG

720 730 740 750 760 770 780

	Sequence
	CCATCGAAAAGGCAGGAGGGCAGTGCAATCAGCACAAGAGCAAGGATCAGCTCGTGCAAATTCTG
1 ➔	CCATCGAAAAGGCAGGAGGGCAGTGCAATCAGCACAAGAGTAAGGATCAGCTCGTGCAAATTCTG
2 ➔	CCATCGAAAAGGCAGGAGGGCAGTGCAATCAGCACAAGAGTAAGGATCAGCTCGTGCAAATTCTG
3 ➔	CCATCGAAAAGGCAGGAGGGCAGTGCAATCAGCACAAGAGTAAGGATCAGCTCGTGCAAATTCTG
4 ➔	CCATCGAAAAGGCAGGAGGGCAGTGCAATCAGCACAAGAGTAAGGATCAGCTCGTGCAAATTCTG
5 ➔	CCATCGAAAAGGCAGGAGGGCAGTGCAATCAGCACAAGAGTAAGGATCAGCTCGTGCAAATTCTG
6 ➔	CCATCGAAAAGGCAGGAGGGCAGTGCAATCAGCACAAGAGCAAGGATCAGCTCGTGCAAATTCTG
7 ➔	CCATCGAAAAGGCAGGAGGGCAGTGCAATCAGCACAAGAGTAAGGATCAGCTCGTGCAAATTCTG
8 ➔	CCATCGAAAAGGCAGGAGGGCAGTGCAATCAGCACAAGAGCAAGGATCAGCTCGTGCAAATTCTG
9 ➔	CCATCGAAAAGGCAGGAGGGCAGTGCAATCAGCACAAGAGCAAGGATCAGCTCGTGCAAATTCTG
10 ➔	CCATCGAAAAGGCAGGAGGGCAGTGCAATCAGCACAAGAGCAAGGATCAGCTCGTGCAAATTCTG
11 ➔	CCATCGAAAAGGCAGGAGGGCAGTGCAATCAGCACAAGAGCAAGGATCAGCTCGTGCAAATTCTG
12 ➔	CCATCGAAAAGGCAGGAGGGCAGTGCAATCAGCACAAGAGCAAGGATCAGCTCGTGCAAATTCTG
13 ➔	CCATCGAAAAGGCAGGAGGGCAGTGCAATCAGCACAAGAGCAAGGATCAGCTCGTGCAAATTCTG
14 ➔	CCATCGAAAAGGCAGGAGGGCAGTGCAATCAGCACAAGAGCAAGGATCAGCTCGTGCAAATTCTG
15 ➔	CCATCGAAAAGGCAGGAGGGCAGTGCAATCAGCACAAGAGCAAGGATCAGCTCGTGCAAATTCTG
16 ➔	CCATCGAAAAGGCAGGAGGGCAGTGCAATCAGCACAAGAGCAAGGATCAGCTCGTGCAAATTCTG
17 ➔	CCATCGAAAAGGCAGGAGGGCAGTGCAATCAGCACAAGAGCAAGGATCAGCTCGTGCAAATTCTG
18 ➔	CCATCGAAAAGGCAGGAGGGCAGTGCAATCAGCACAAGAGCAAGGATCAGCTCGTGCAAATTCTG
19 ➔	CCATCGAAAAGGCAGGAGGGCAGTGCAATCAGCACAAGAGCAAGGATCAGCTCGTGCAAATTCTG
20 ➔	CCATCGAAAAGGCAGGAGGGCAGTGCAATCAGCACAAGAGCAAGGATCAGCTCGTGCAAATTCTG
21 ➔	CCATCGAAAAGGCAGGAGGGCAGTGCAATCAGCACAAGAGCAAGGATCAGCTCGTGCAAATTCTG
22 ➔	CCATCGAAAAGGCAGGAGGGCAGTGCAATCAGCACAAGAGCAAGGATCAGCTCGTGCAAATTCTG
23 ➔	CCATCGAAAAGGCAGGAGGGCAGTGCAATCAGCACAAGAGCAAGGATCAGCTCGTGCAAATTCTG
24 ➔	CCATCGAAAAGGCAGGAGGGCAGTGCAATCAGCACAAGAGCAAGGATCAGCTCGTGCAAATTCTG
25 ➔	CCATCGAAAAGGCAGGAGGGCAGTGCAATCAGCACAAGAGCAAGGATCAGCTCGTGCAAATTCTG
26 ➔	CCATCGAAAAGGCAGGAGGGCAGTGCAATCAGCACAAGAGCAAGGATCAGCTCGTGCAAATTCTG
27 ➔	CCATCGAAAAGGCAGGAGGGCAGTGCAATCAGCACAAGAGCAAGGATCAGCTCGTGCAAATTCTG
28 ➔	CCATCGAAAAGGCAGGAGGGCAGTGCAATCAGCACAAGAGCAAGGATCAGCTCGTGCAAATTCTG
29 ➔	CCATCGAAAAGGCAGGAGGGCAGTGCAATCAGCACAAGAGCAAGGATCAGCTCGTGCAAATTCTG
30 ➔	CCATCGAAAAGGCAGGAGGGCAGTGCAATCAGCACAAGAGCAAGGATCAGCTCGTGCAAATTCTG
31 ➔	CCATCGAAAAGGCAGGAGGGCAGTGCAATCAGCACAAGAGCAAGGATCAGCTCGTGCAAATTCTG
32 ➔	CCATCGAAAAGGCAGGAGGGCAGTGCAATCAGCACAAGAGCAAGGATCAGCTCGTGCAAATTCTG
33 ➔	CCATCGAAAAGGCAGGAGGGCAGTGCAATCAGCACAAGAGCAAGGATCAGCTCGTGCAAATTCTG
34 ➔	CCATCGAAAAGGCAGGAGGGCAGTGCAATCAGCACAAGAGCAAGGATCAGCTCGTGCAAATTCTG
35 ➔	CCATCGAAAAGGCAGGAGGGCAGTGCAATCAGCACAAGAGCAAGGATCAGCTCGTGCAAATTCTG
36 ➔	CCATCGAAAAGGCAGGAGGGCAGTGCAATCAGCACAAGAGCAAGGATCAGCTCGTGCAAATTCTG
37 ➔	CCATCGAAAAGGCAGGAGGGCAGTGCAATCAGCACAAGAGCAAGGATCAGCTCGTGCAAATTCTG
38 ➔	CCATCGAAAAGGCAGGAGGGCAGTGCAATCAGCACAAGAGCAAGGATCAGCTCGTGCAAATTCTG
39 ➔	CCATCGAAAAGGCAGGAGGGCAGTGCAATCAGCACAAGAGCAAGGATCAGCTCGTGCAAATTCTG
40 ➔	CCATCGAAAAGGCAGGAGGGCAGTGCAATCAGCACAAGAGCAAGGATCAGCTCGTGCAAATTCTG
41 ➔	CCATCGAAAAGGCAGGAGGGCAGTGCAATCAGCACAAGAGCAAGGATCAGCTCGTGCAAATTCTG
42 ➔	CCATCGAAAAGGCAGGAGGGCAGTGCAATCAGCACAAGAGCAAGGATCAGCTCGTGCAAATTCTG

```
      790       800       810       820       830       840
   CTACATTCCATCAGTGGGAAGAGTGTCTTTCTTGTGCTGGATAACGTTACCAACCCTGATGTGTG
1  CTACATTCCATCAGTGGGAAGAGTGTCTTTCTTGTGCTGGATAACGTTACCAACCCTGATGTGTG
2  CTACATTCCATCAGTGGGAAGAGTGTCTTTCTTGTGCTGGATAACGTTACCAACCCTGATGTGTG
3  CTACATTCCATCAGTGGGAAGAGTGTCTTTCTTGTGCTGGATAACGTTACCAACCCTGATGTGTG
4  CTACATTCCGTCAGTGGGAAGAGTGTCTTTCTTGTGCTGGATAACGTTACCAACCCTGATGTGTG
5  CTACATTCCGTCAGTGGGAAGAGTGTCTTTCTTGTGCTGGATAACGTTACCAACCCTGATGTGTG
6  CTACATTCCATCAGTGGGAAGAGTGTCTTTCTTGTGCTGGATAACGTTACCAACCCTGATGTGTG
7  CTACATTCCATCAGTGGGAAGAGTGTCTTTCTTGTGCTGGATAACGCTACCAACCCTGATGTGTG
8  CTACATTCCATCAGTGGGAAGAGTGTCTTTCTTGTGCTGGATAACGTTACCAACCCTGATGTGTG
9  CTACATTCCATCAGTGGGAAG--TGTCTTTCTTGTGCTGGATAACGTTACCAACCCTGATGTGTG
10 CTACATTCCATCAGTGGGAAG--TGTCTTTCTTGTGCTGGATAACGTTACCAACCCTGATGTGTG
11 CTACATTCCATCAGTGGGAAGAGTGTCTTTCTTGTGCTGGATAACGTTACCAACCCTGATGTGTG
12 CTACATTCCATCAGTGGGAAGAGTGTCTTTCTTGTGCTGGATAACGTTACCAACCCTGATGTGTG
13 CTACATTCCATCAGTGGGAAGAGTGTCTTTCTTGTGCTGGATAACGTTACCAACCCTGATGTGTG
14 CTACATTCCATCAGTGGGAAGAGTGTCTTTCTTGTGCTGGATAACGTTACCAACCCTGATGTGTG
15 CTACATTCCATCAGTGGGAAGAGTGTCTTTCTTGTGCTGGATAACGTTACCAACCCTGATGTGTG
16 CTACATTCCATCAGTGGGAAGAGTGTCTTTCTTGTGCTGGATAACGTTACCAACCCTGATGTGTG
17 CTACATTCCATCAGTGGGAAGAGTGTCTTTCTTGTGCTGGATAACGTTACCAACCCTGATGTGTG
18 CTACATTCCATCAGTGGGAAGAGTGTCTTTCTTGTGCTGGATAACGTTACCAACCCTGATGTGTG
19 CTACATTCCATCAGTGGGAAGAGTGTCTTTCTTGTGCTGGATAACGTTACCAACCCTGATGTGTG
20 CTACATTCCATCAGTGGGAAGAGTGTCTTTCTTGTGCTGGATAACGTTACCAACCCTGATGTGTG
21 CTACATTCCATCAGTGGGAAGAGTGTCTTTCTTGTGCTGGATAACGTTACCAACCCTGATGTGTG
22 CTACATTCCATCAGTGGGAAGAGTGTCTTTCTTGTGCTGGATAACGTTACCAACCCTGATGTGTG
23 CTACATTCCATCAGTGGGAAGAGTGTCTTTCTTGTGCTGGATAACGTTACCAACCCTGATGTGTG
24 CTACATTCCATCAGTGGGAAGAGTGTCTTTCTTGTGCTGGATAACGTTACCAACCCTGATGTGTG
25 CTACATTCCATCAGTGGGAAGAGTGTCTTTCTTGTGCTGGATAACGTTACCAACCCTGATGTGTG
26 CTACATTCCATCAGTGGGAAGAGTGTCTTTCTTGTGCTGGATAACGTTACCAACCCTGATGTGTG
27 CTACATTCCATCAGTGGGAAGAGTGTCTTTCTTGTGCTGGATAACGTTACCAACCCTGATGTGTG
28 CTACATTCCATCAGTGGGAAGAGTGTCTTTCTTGTGCTGGATAACGTTACCAACCCTGATGTGTG
29 CTACATTCCATCAGTGGGAAGAGTGTCTTTCTTGTGCTGGATAACGTTACCAACCCTGATGTGTG
30 CTACATTCCATCAGTGGGAAGAGTGTCTTTCTTGTGCTGGATAACGTTACCAACCCTGATGTGTG
31 CTACATTCCATCAGTGGGAAGAGTGTCTTTCTTGTGCTGGATAACGTTACCAACCCTGATGTGTG
32 CTACATTCCATCAGTGGGAAGAGTGTCTTTCTTGTGCTGGATAACGTTACCAACCCTGATGTGTG
33 CTACATTCCATCAGTGGGAAGAGTGTCTTTCTTGTGCTGGATAACGTTACCAACCCTGATGTGTG
34 CTACATTCCATCAGTGGGAAGAGTGTCTTTCTTGTGCTGGATAACGTTACCAACCCTGATGTGTG
35 CTACATTCCATCAGTGGGAAGAGTGTCTTTCTTGTGCTGGATAACGTTACCAACCCTGATGTGTG
36 CTACATTCCATCAGTGGGAAGAGTGTCTTTCTTGTGCTGGATAACGTTACCAACCCTGATGTGTG
37 CTACATTCCATCAGTGGGAAGAGTGTCTTTCTTGTGCTGGATAACGTTACCAACCCTGATGTGTG
38 CTACATTCCATCAGTGGGAAGAGTGTCTTTCTTGTGCTGGATAACGTTACCAACCCTGATGTGTG
39 CTACATTCCATCAGTGGGAAGAGTGTCTTTCTTGTGCTGGATAACGTTACCAACCCTGATGTGTG
40 CTACATTCCATCAGTGGGAAGAGTGTCTTTCTTGTGCTGGATAACGTTACCAACCCTGATGTGTG
41 CTACATTCCATCAGTGGGAAGAGTGTCTTTCTTGTGCTGGATAACGTTACCAACCCTGATGTGTG
42 CTACATTCCATCAGTGGGAAGAGTGTCTTTCTTGTGCTGGATAACGTTACCAACCCTGATGTGTG
```

	850 · 860 · 870 · 880 · 890 · 900 · 910
	GATCGATCTTCTCCGCTCTCCGATGGAGAGGTGTTTGGATGCTCATGTACTTGTTACCACAAGGA
1 ➔	GATCGATCTTCTCCGCTCTCCGATGGAGAGGTGTTTGGATGCTCATGTACTTGTTACCACAAGGA
2 ➔	GATCGATCTTCTCCGCTCTCCGATGGAGAGGTGTTTGGATGCTCATGTACTTGTTACCACAAGGA
3 ➔	GATCGATCTTCTCCGCTCTCCGATGGAGAGGTGTTTGGATGCTCATGTACTTGTTACCACAAGGA
4 ➔	GATCGATCTTCTCCGCTCTCCGATGGAGAGGTGTTTGGATGCTCATGTACTTGTTACCACAAGGA
5 ➔	GATCGATCTTCTCCGCTCTCCGATGGAGAGGTGTTTGGATGCTCATGTACTTGTTACCACAAGGA
6 ➔	GATCGATCTTCTCCGCTCTCCGATGGAGAGGTGTTTGGATGCTCATGTACTTGTTACCACAAGGA
7 ➔	GATCGATCTTCTCCGCTCTCCGATGGAGAGGTGTTTGGATGCTCATGTACTTGTTACCACAAGGA
8 ➔	GATCGATCTTCTCCGCTCTCCGATGGAGAGGTGTTTGGATGCTCATGTACTTGTTACCACAAGGA
9 ➔	GATCGATCTTCTCCGCTCTCCGATGGAGAGGTGTTTGGATGCTCATGTACTTGTTACCACAAGGA
10 ➔	GATCGATCTTCTCCGCTCTCCGATGGAGAGGTGTTTGGATGCTCATGTACTTGTTACCACAAGGA
11 ➔	GATCGATCTTCTCCGCTCTCCGATGGAGAGGTGTTTGGATGCTCATGTACTTGTTACCACAAGGA
12 ➔	GATCGATCTTCTCCGCTCTCCGATGGAGAGGTGTTTGGATGCTCATGTACTTGTTACCACAAGGA
13 ➔	GATCGATCTTCTCCGCTCTCCGATGGAGAGGTGTTTGGATGCTCATGTACTTGTTACCACAAGGA
14 ➔	GATCGATCTTCTCCGCTCTCCGATGGAGAGGTGTTTGGATGCTCATGTACTTGTTACCACAAGGA
15 ➔	GATCGATCTTCTCCGCTCTCCGATGGAGAGGTGTTTGGATGCTCATGTACTTGTTACCACAAGGA
16 ➔	GATCGATCTTCTCCGCTCTCCGATGGAGAGGTGTTTGGATGCTCATGTACTTGTTACCACAAGGA
17 ➔	GATCGATCTTCTCCGCTCTCCGATGGAGAGGTGTTTGGATGCTCATGTACTTGTTACCACAAGGA
18 ➔	GATCGATCTTCTCCGCTCTCCGATGGAGAGGTGTTTGGATGCTCATGTACTTGTTACCACAAGGA
19 ➔	GATCGATCTTCTCCGCTCTCCGATGGAGAGGTGTTTGGATGCTCATGTACTTGTTACCACAAGGA
20 ➔	GATCGATCTTCTCCGCTCTCCGATGGAGAGGTGTTTGGATGCTCATGTACTTGTTACCACAAGGA
21 ➔	GATCGATCTTCTCCGCTCTCCGATGGAGAGGTGTTTGGATGCTCATGTACTTGTTACCACAAGGA
22 ➔	GATCGATCTTCTCCGCTCTCCGATGGAGAGGTGTTTGGATGCTCATGTACTTGTTACCACAAGGA
23 ➔	GATCGATCTTCTCCGCTCTCCGATGGAGAGGTGTTTGGATGCTCATGTACTTGTTACCACAAGGA
24 ➔	GATCGATCTTCTCCGCTCTCCGATGGAGAGGTGTTTGGATGCTCATGTACTTGTTACCACAAGGA
25 ➔	GATCGATCTTCTCCGCTCTCCGATGGAGAGGTGTTTGGATGCTCATGTACTTGTTACCACAAGGA
26 ➔	GATCGATCTTCTCCGCTCTCCGATGGAGAGGTGTTTGGATGCTCATGTACTTGTTACCACAAGGA
27 ➔	GATCGATCTTCTCCGCTCTCCGATGGAGAGGTGTTTGGATGCTCATGTACTTGTTACCACAAGGA
28 ➔	GATCGATCTTCTCCGCTCTCCGATGGAGAGGTGTTTGGATGCTCATGTACTTGTTACCACAAGGA
29 ➔	GATCGATCTTCTCCGCTCTCCGATGGAGAGGTGTTTGGATGCTCATGTACTTGTTACCACAAGGA
30 ➔	GATCGATCTTCTCCGCTCTCCGATGGAGAGGTGTTTGGATGCTCATGTACTTGTTACCACAAGGA
31 ➔	GATCGATCTTCTCCGCTCTCCGATGGAGAGGTGTTTGGATGCTCATGTACTTGTTACCACAAGGA
32 ➔	GATCGATCTTCTCCGCTCTCCGATGGAGAGGTGTTTGGATGCTCATGTACTTGTTACCACAAGGA
33 ➔	GATCGATCTTCTCCGCTCTCCGATGGAGAGGTGTTTGGATGCTCATGTACTTGTTACCACAAGGA
34 ➔	GATCGATCTTCTCCGCTCTCCGATGGAGAGGTGTTTGGATGCTCATGTACTTGTTACCACAAGGA
35 ➔	GATCGATCTTCTCCGCTCTCCGATGGAGAGGTGTTTGGATGCTCATGTACTTGTTACCACAAGGA
36 ➔	GATCGATCTTCTCCGCTCTCCGATGGAGAGGTGTTTGGATGCTCATGTACTTGTTACCACAAGGA
37 ➔	GATCGATCTTCTCCGCTCTCCGATGGAGAGGTGTTTGGATGCTCATGTACTTGTTACCACAAGGA
38 ➔	GATCGATCTTCTCCGCTCTCCGATGGAGAGGTGTTTGGATGCTCATGTACTTGTTACCACAAGGA
39 ➔	GATCGATCTTCTCCGCTCTCCGATGGAGAGGTGTTTGGATGCTCATGTACTTGTTACCACAAGGA
40 ➔	GATCGATCTTCTCCGCTCTCCGATGGAGAGGTGTTTGGATGCTCATGTACTTGTTACCACAAGGA
41 ➔	GATCGATCTTCTCCGCTCTCCGATGGAGAGGTGTTTGGATGCTCATGTACTTGTTACCACAAGGA
42 ➔	GATCGATCTTCTCCGCTCTCCGATGGAGAGGTGTTTGGATGCTCATGTACTTGTTACCACAAGGA

	920 930 940 950 960 970
	GCGGTCACGTATTGTCACAGATGAATGCGGTGCATGTCAAGGAAATGCACAGACTAAAGGATGCT
1	GCGGTCACGTATTGTCACAGATGAATGCGGTTCATGTCAAGGAAATGCATAGACTAAAGGATGCT
2	GCGGTCACGTATTGTCACAGATGAATGCGGTTCATGTCAAGGAAATGCATAGACTAAAGGATGCT
3	GCGGTCACGTATTGTCACAGATGAATGCGGTTCATGTCAAGGAAATGCATAGACTAAAGGATGCT
4	TCGATGGCGTATTGTCACAGATGAATGCGGTTCATGTCAAGGAAATGCATAGACTAAAGGATGCT
5	TCGATGGCGTATTGTCACAGATGAATGCGGTTCATGTCAAGGAAATGCATAGACTAAAGGATGCT
6	GCGGTCACGTATTGTCACAGATGAATGCGGTGCATGTCAAGGAAATGCACAGACTAAAGGATGCT
7	GCGGTCACGTATTGTCACAGATGAATGCGGTTCATGTCAAGGAAATGCATAGACTAAAGGATGCT
8	GCGGTCGCGTATTGTCACAGATGAATGCGGTGCATGTCAAGGAAATGCACAGACTAAAGGATGCT
9	GCGGTCGCGTATTGTCACAGATGAATGCGGTGCATGTCAAGGAAATGCACAGACTAAAGGATGCT
10	GCGGTCGCGTATTGTCACAGATGAATGCGGTGCATGTCAAGGAAATGCACAGACTAAAGGATGCT
11	GCGGTCACGTATTGTCACAGGTGAATGCGGTGCATGTCAAGGAAATGCACAGACTAAAGGATGCT
12	GCGGTCACGTATTGTCACAGGTGAATGCGGTGCATGTCAAGGAAATGCACAGACTAAAGGATGCT
13	GCGGTCACGTATTGTCACAGGTGAATGCGGTGCATGTCAAGGAAATGCACAGACTAAAGGATGCT
14	GCGGTCACGTATTGTCACAGATGAATGCGGTGCATGTCAAGGAAATGCACAGACTAAAGGATGCT
15	GCGGTCACGTATTGTCACAGATGAATGCGGTGCATGTCAAGGAAATGCACAGACTAAAGGATGCT
16	GCGGTCACGTATTGTCACAGATGAATGCGGTGCATGTCAAGGAAATGCACAGACTAAAGGATGCT
17	GCGGTCACGTATTGTCACAGATGAATGCGGTGCATGTCAAGGAAATGCACAGACTAAAGGATGCT
18	GCGGTCACGTATTGTCACAGATGAATGCGGTGCATGTCAAGGAAATGCACAGACTAAAGGATGCT
19	GCGGTCACGTATTGTCACAGATGAATGCGGTGCATGTCAAGGAAATGCACAGACTAAAGGATGCT
20	GCGGTCACGTATTGTCACAGATGAATGCGGTGCATGTCAAGGAAATGCACAGACTAAAGGATGCT
21	GCGGTCACGTATTGTCACAGATGAATGCGGTGCATGTCAAGGAAATGCACAGACTAAAGGATGCT
22	GCGGTCACGTATTGTCACAGATGAATGCGGTGCATGTCAAGGAAATGCACAGACTAAAGGATGCT
23	GCGGTCACGTATTGTCACAGATGAATGCGGTGCATGTCAAGGAAATGCACAGACTAAAGGATGCT
24	GCGGTCACGTATTGTCACAGATGAATGCGGTGCATGTCAAGGAAATGCACAGACTAAAGGATGCT
25	GCGGTCACGTATTGTCACAGATGAATGCGGTGCATGTCAAGGAAATGCACAGACTAAAGGATGCT
26	GCGGTCACGTATTGTCACAGATGAATGCGGTGCATGTCAAGGAAATGCACAGACTAAAGGATGCT
27	GCGGTCACGTATTGTCACAGATGAATGCGGTGCATGTCAAGGAAATGCACAGACTAAAGGATGCT
28	GCGGTCACGTATTGTCACAGATGAATGCGGTGCATGTCAAGGAAATGCACAGACTAAAGGATGCT
29	GCGGTCACGTATTGTCACAGATGAATGCGGTGCATGTCAAGGAAATGCACAGACTAAAGGATGCT
30	GCGGTCACGTATTGTCACAGATGAATGCGGTGCATGTCAAGGAAATGCACAGACTAAAGGATGCT
31	GCGGTCACGTATTGTCACAGATGAATGCGGTGCATGTCAAGGAAATGCACAGACTAAAGGATGCT
32	GCGGTCACGTATTGTCACAGATGAATGCGGTGCATGTCAAGGAAATGCACAGACTAAAGGATGCT
33	GCGGTCACGTATTGTCACAGATGAATGCGGTGCATGTCAAGGAAATGCACAGACTAAAGGATGCT
34	GCGGTCACGTATTGTCACAGATGAATGCGGTGCATGTCAAGGAAATGCACAGACTAAAGGATGCT
35	GCGGTCACGTATTGTCACAGATGAATGCGGTGCATGTCAAGGAAATGCACAGACTAAAGGATGCT
36	GCGGTCACGTATTGTCACAGATGAATGCGGTGCATGTCAAGGAAATGCACAGACTAAAGGATGCT
37	GCGGTCACGTATTGTCACAGATGAATGCGGTGCATGTCAAGGAAATGCACAGACTAAAGGATGCT
38	GCGGTCACGTATTGTCACAGATGAATGCGGTGCATGTCAAGGAAATGCACAGACTAAAGGATGCT
39	GCGGTCACGTATTGTCACAGATGAATGCGGTGCATGTCAAGGAAATGCACAGACTAAAGGATGCT
40	GCGGTCACGTATTGTCACAGATGAATGCGGTGCATGTCAAGGAAATGCACAGACTAAAGGATGCT
41	GCGGTCACGTATTGTCACAGATGAATGCGGTGCATGTCAAGGAAATGCACAGACTAAAGGATGCT
42	GCGGTCACGTATTGTCACAGATGAATGCGGTGCATGTCAAGGAAATGCACAGACTAAAGGATGCT

980 990 1000 1010 1020 1030 1040

	GATGGCCTAGAACTGCTTATGAAGAGATCTTTCAGAACCAAAGACGAAGTAAATGTATTCAGTGA
1 ➨	GATGGCCTAGAACTGCTTATGAAGAGATCTTTCAGAACTGAAGATGAAGTAAATGTATTCAGTGA
2 ➨	GATGGCCTAGAACTGCTTATGAAGAGATCTTTCAGAACTGAAGATGAAGTAAATGTATTCAGTGA
3 ➨	GATGGCCTAGAACTGCTTATGAAGAGATCTTTCAGAACTGAAGATGAAGTAAATGTATTCAGTGA
4 ➨	GATGGCCTAGAACTGCTTATGAAGAGATCTTTCAGAACTGAAGATGAAGTAAATGTATTCAGTGA
5 ➨	GATGGCCTAGAACTGCTTATGAAGAGATCTTTCAGAACTGAAGATGAAGTAAATGTATTCAGTGA
6 ➨	GATGGCCTAGAACTGCTTATGAAGAGATCTTTCAGAACCAAAGACGAAGTAAATGTATTCAGTGA
7 ➨	GATGGCCTAGAACTGCTTATGAAGAGATCTTTCAGAACTGAAGATGAAGTAAATGTATTCAGTGA
8 ➨	GATGGCCTAGAACTGCTTATGAAGAGATCTTTCAGAACCAAAGACGAAGTAAATGTATTCAGTGA
9 ➨	GATGGCCTAGAACTGCTTATGAAGAGATCTTTCAGAACCAAAGACGAAGTAAATGTATTCAGTGA
10 ➨	GATGGCCTAGAACTGCTTATGAAGAGATCTTTCAGAACCAAAGACGAAGTAAATGTATTCAGTGA
11 ➨	GATGGCCTAGAACTGCTTATGAAGAGATCTTTCAGAACCAAAGACGAAGTAAATGTATTCAGTGA
12 ➨	GATGGCCTAGAACTGCTTATGAAGAGATCTTTCAGAACCAAAGACGAAGTAAATGTATTCAGTGA
13 ➨	GATGGCCTAGAACTGCTTATGAAGAGATCTTTCAGAACCAAAGACGAAGTAAATGTATTCAGTGA
14 ➨	GATGGCCTAGAACTGCTTATGAAGAGATCTTTCAGAACCAAAGACGAAGTAAATGTATTCAGTGA
15 ➨	GATGGCCTAGAACTGCTTATGAAGAGATCTTTCAGAACCAAAGACGAAGTAAATGTATTCAGTGA
16 ➨	GATGGCCTAGAACTGCTTATGAAGAGATCTTTCAGAACCAAAGACGAAGTAAATGTATTCAGTGA
17 ➨	GATGGCCTAGAACTGCTTATGAAGAGATCTTTCAGAACCAAAGACGAAGTAAATGTATTCAGTGA
18 ➨	GATGGCCTAGAACTGCTTATGAAGAGATCTTTCAGAACCAAAGACGAAGTAAATGTATTCAGTGA
19 ➨	GATGGCCTAGAACTGCTTATGAAGAGATCTTTCAGAACCAAAGACGAAGTAAATGTATTCAGTGA
20 ➨	GATGGCCTAGAACTGCTTATGAAGAGATCTTTCAGAACCAAAGACGAAGTAAATGTATTCAGTGA
21 ➨	GATGGCCTAGAACTGCTTATGAAGAGATCTTTCAGAACCAAAGACGAAGTAAATGTATTCAGTGA
22 ➨	GATGGCCTAGAACTGCTTATGAAGAGATCTTTCAGAACCAAAGACGAAGTAAATGTATTCAGTGA
23 ➨	GATGGCCTAGAACTGCTTATGAAGAGATCTTTCAGAACCAAAGACGAAGTAAATGTATTCAGTGA
24 ➨	GATGGCCTAGAACTGCTTATGAAGAGATCTTTCAGAACCAAAGACGAAGTAAATGTATTCAGTGA
25 ➨	GATGGCCTAGAACTGCTTATGAAGAGATCTTTCAGAACCAAAGACGAAGTAAATGTATTCAGTGA
26 ➨	GATGGCCTAGAACTGCTTATGAAGAGATCTTTCAGAACCAAAGACGAAGTAAATGTATTCAGTGA
27 ➨	GATGGCCTAGAACTGCTTATGAAGAGATCTTTCAGAACCAAAGACGAAGTAAATGTATTCAGTGA
28 ➨	GATGGCCTAGAACTGCTTATGAAGAGATCTTTCAGAACCAAAGACGAAGTAAATGTATTCAGTGA
29 ➨	GATGGCCTAGAACTGCTTATGAAGAGATCTTTCAGAACCAAAGACGAAGTAAATGTATTCAGTGA
30 ➨	GATGGCCTAGAACTGCTTATGAAGAGATCTTTCAGAACCAAAGACGAAGTAAATGTATTCAGTGA
31 ➨	GATGGCCTAGAACTGCTTATGAAGAGATCTTTCAGAACCAAAGACGAAGTAAATGTATTCAGTGA
32 ➨	GATGGCCTAGAACTGCTTATGAAGAGATCTTTCAGAACCAAAGACGAAGTAAATGTATTCAGTGA
33 ➨	GATGGCCTAGAACTGCTTATGAAGAGATCTTTCAGAACCAAAGACGAAGTAAATGTATTCAGTGA
34 ➨	GATGGCCTAGAACTGCTTATGAAGAGATCTTTCAGAACCAAAGACGAAGTAAATGTATTCAGTGA
35 ➨	GATGGCCTAGAACTGCTTATGAAGAGATCTTTCAGAACCAAAGACGAAGTAAATGTATTCAGTGA
36 ➨	GATGGCCTAGAACTGCTTATGAAGAGATCTTTCAGAACCAAAGACGAAGTAAATGTATTCAGTGA
37 ➨	GATGGCCTAGAACTGCTTATGAAGAGATCTTTCAGAACCAAAGACGAAGTAAATGTATTCAGTGA
38 ➨	GATGGCCTAGAACTGCTTATGAAGAGATCTTTCAGAACCAAAGACGAAGTAAATGTATTCAGTGA
39 ➨	GATGGCCTAGAACTGCTTATGAAGAGATCTTTCAGAACCAAAGACGAAGTAAATGTATTCAGTGA
40 ➨	GATGGCCTAGAACTGCTTATGAAGAGATCTTTCAGAACCAAAGACGAAGTAAATGTATTCAGTGA
41 ➨	GATGGCCTAGAACTGCTTATGAAGAGATCTTTCAGAACCAAAGACGAAGTAAATGTATTCAGTGA
42 ➨	GATGGCCTAGAACTGCTTATGAAGAGATCTTTCAGAACCAAAGACGAAGTAAATGTATTCAGTGA

```
        1050      1060      1070      1080      1090      1100
     TATTGGAGCAAAAATTGTTAAGAAATGTGATGGCCTTCCTCTTGCCATCAAGGTCATTGGAGGCG
1    TATTGGAGCAAAAATTGTTAAGAAATGTGATGGCCTTCCGCTTGCCATCAAGGTCATTGGGGGCG
2    TATTGGAGCAAAAATTGTTAAGAAATGTGATGGCCTTCCGCTTGCCATCAAGGTCATTGGGGGCG
3    TATTGGAGCAAAAATTGTTAAGAAATGTGATGGCCTTCCGCTTGCCATCAAGGTCATTGGGGGCG
4    TATTGGAGCAAAAATTGTTAAGAAATGCGAGGGCCTTCCGCTTGCCATCAAGGTCATTGGGGGTG
5    TATTGGAGCAAAAATTGTTAAGAAATGCGAGGGCCTTCCGCTTGCCATCAAGGTCATTGGGGGTG
6    TATTGGAGCAAAAATTGTTAAGAAATGTGATGGCCTTCCTCTTGCCATCAAGGTCATTGGAGGCG
7    TATTGGAGCAAAAATTGTTAAGAAATGTGATGGCCTTCCGCTTGCCATCAAGGTCATTGGGGGCG
8    TATTGGAGCAAAAATTGTTAAGAAATGTGATGGCCTTCCTCTTGCCATCAAGGTCATTGGAGGCG
9    TATTGGAGCAAAAATTGTTAAGAAATGTGAGGGCCTTCCTCTTGCCATCAAGGTCATTGGAGGCG
10   TATTGGAGCAAAAATTGTTAAGAAATGTGAGGGCCTTCCTCTTGCCATCAAGGTCATTGGAGGCG
11   TATTGGAGCAAAAATTGTTAAGAAATGTGATGGCCTTCCTCTTGCCATCAAGGTCATTGGAGGCG
12   TATTGGAGCAAAAATTGTTAAGAAATGTGATGGCCTTCCTCTTGCCATCAAGGTCATTGGAGGCG
13   TATTGGAGCAAAAATTGTTAAGAAATGTGATGGCCTTCCTCTTGCCATCAAGGTCATTGGAGGCG
14   TATTGGAGCAAAAATTGTTAAGAAATGTGATGGCCTTCCTCTTGCCATCAAGGTCATTGGAGGCG
15   TATTGGAGCAAAAATTGTTAAGAAATGTGATGGCCTTCCTCTTGCCATCAAGGTCATTGGAGGCG
16   TATTGGAGCAAAAATTGTTAAGAAATGTGATGGCCTTCCTCTTGCCATCAAGGTCATTGGAGGCG
17   TATTGGAGCAAAAATTGTTAAGAAATGTGATGGCCTTCCTCTTGCCATCAAGGTCATTGGAGGCG
18   TATTGGAGCAAAAATTGTTAAGAAATGTGATGGCCTTCCTCTTGCCATCAAGGTCATTGGAGGCG
19   TATTGGAGCAAAAATTGTTAAGAAATGTGATGGCCTTCCTCTTGCCATCAAGGTCATTGGAGGCG
20   TATTGGAGCAAAAATTGTTAAGAAATGTGATGGCCTTCCTCTTGCCATCAAGGTCATTGGAGGCG
21   TATTGGAGCAAAAATTGTTAAGAAATGTGATGGCCTTCCTCTTGCCATCAAGGTCATTGGAGGCG
22   TATTGGAGCAAAAATTGTTAAGAAATGTGATGGCCTTCCTCTTGCCATCAAGGTCATTGGAGGCG
23   TATTGGAGCAAAAATTGTTAAGAAATGTGATGGCCTTCCTCTTGCCATCAAGGTCATTGGAGGCG
24   TATTGGAGCAAAAATTGTTAAGAAATGTGATGGCCTTCCTCTTGCCATCAAGGTCATTGGAGGCG
25   TATTGGAGCAAAAATTGTTAAGAAATGTGATGGCCTTCCTCTTGCCATCAAGGTCATTGGAGGCG
26   TATTGGAGCAAAAATTGTTAAGAAATGTGATGGCCTTCCTCTTGCCATCAAGGTCATTGGAGGCG
27   TATTGGAGCAAAAATTGTTAAGAAATGTGATGGCCTTCCTCTTGCCATCAAGGTCATTGGAGGCG
28   TATTGGAGCAAAAATTGTTAAGAAATGTGATGGCCTTCCTCTTGCCATCAAGGTCATTGGAGGCG
29   TATTGGAGCAAAAATTGTTAAGAAATGTGATGGCCTTCCTCTTGCCATCAAGGTCATTGGAGGCG
30   TATTGGAGCAAAAATTGTTAAGAAATGTGATGGCCTTCCTCTTGCCATCAAGGTCATTGGAGGCG
31   TATTGGAGCAAAAATTGTTAAGAAATGTGATGGCCTTCCTCTTGCCATCAAGGTCATTGGAGGCG
32   TATTGGAGCAAAAATTGTTAAGAAATGTGATGGCCTTCCTCTTGCCATCAAGGTCATTGGAGGCG
33   TATTGGAGCAAAAATTGTTAAGAAATGTGATGGCCTTCCTCTTGCCATCAAGGTCATTGGAGGCG
34   TATTGGAGCAAAAATTGTTAAGAAATGTGATGGCCTTCCTCTTGCCATCAAGGTCATTGGAGGCG
35   TATTGGAGCAAAAATTGTTAAGAAATGTGATGGCCTTCCTCTTGCCATCAAGGTCATTGGAGGCG
36   TATTGGAGCAAAAATTGTTAAGAAATGTGATGGCCTTCCTCTTGCCATCAAGGTCATTGGAGGCG
37   TATTGGAGCAAAAATTGTTAAGAAATGTGATGGCCTTCCTCTTGCCATCAAGGTCATTGGAGGCG
38   TATTGGAGCAAAAATTGTTAAGAAATGTGATGGCCTTCCTCTTGCCATCAAGGTCATTGGAGGCG
39   TATTGGAGCAAAAATTGTTAAGAAATGTGATGGCCTTCCTCTTGCCATCAAGGTCATTGGAGGCG
40   TATTGGAGCAAAAATTGTTAAGAAATGTGATGGCCTTCCTCTTGCCATCAAGGTCATTGGAGGCG
41   TATTGGAGCAAAAATTGTTAAGAAATGTGATGGCCTTCCTCTTGCCATCAAGGTCATTGGAGGCG
42   TATTGGAGCAAAAATTGTTAAGAAATGTGATGGCCTTCCTCTTGCCATCAAGGTCATTGGAGGCG
```

	1110 1120 1130 1140 1150 1160 1170
	TCCTATCATCCAGGTCGAGCAAAGAAGAATGGGAGAGAATACTGGAGAGAAGATGGTCTATTGAT
1	TCCTATCATCCAGGTCGAGCAAAGAAGAATGGGAGAGAATACTGGAGAGAAGATGGTCTATTGAT
2	TCCTATCATCCAGGTCGAGCAAAGAAGAATGGGAGAGAATACTGGAGAGAAGATGGTCTATTGAT
3	TCCTATCATCCAGGTCGAGCAAAGAAGAATGGGAGAGAATACTGGAGAGAAGATGGTCTATTGAT
4	TCCTATCATCCAGGTCGAGCAAAGAAGAATGGGAGAGAATACTGGAGAGAAGATGGTCTATTGAT
5	TCCTATCATCCAGGTCGAGCAAAGAAGAATGGGAGAGAATACTGGAGAGAAGATGGTCTATTGAT
6	TCCTATCATCCAGGTCGAGCAAAGAAGAATGGGAGAGAATACTGGAGAGAAGATGGTCTATTGAT
7	TCCTATCATCCAGGTCGAGCAAAGAAGAATGGGAGAGAATACTGGAGAGAAGATGGTCTATTGAT
8	TCCTATCATCCAGGTCGAGCAAAGAAGAATGGGAGAGAATACTGGAGAGAAGATGGTCTATTGAT
9	TCCTATCATCCAGGTCGAGCAAAGAAGAATGGGAGAGAATACTGGAGAGAAGATGGTCTATTGAT
10	TCCTATCATCCAGGTCGAGCAAAGAAGAATGGGAGAGAATACTGGAGAGAAGATGGTCTATTGAT
11	TCCTATCATCCAGGTCGAGCAAAGAAGAATGGGAGAGAATACTGGAGAGAAGATGGTCTATTGAT
12	TCCTATCATCCAGGTCGAGCAAAGAAGAATGGGAGAGAATACTGGAGAGAAGATGGTCTATTGAT
13	TCCTATCATCCAGGTCGAGCAAAGAAGAATGGGAGAGAATACTGGAGAGAAGATGGTCTATTGAT
14	TCCTATCATCCAGGTCGAGCAAAGAAGAATGGGAGAGAATACTGGAGAGAAGATGGTCTATTGAT
15	TCCTATCATCCAGGTCGAGCAAAGAAGAATGGGAGAGAATACTGGAGAGAAGATGGTCTATTGAT
16	TCCTATCATCCAGGTCGAGCAAAGAAGAATGGGAGAGAATACTGGAGAGAAGATGGTCTATTGAT
17	TCCTATCATCCAGGTCGAGCAAAGAAGAATGGGAGAGAATACTGGAGAGAAGATGGTCTATTGAT
18	TCCTATCATCCAGGTCGAGCAAAGAAGAATGGGAGAGAATACTGGAGAGAAGATGGTCTATTGAT
19	TCCTATCATCCAGGTCGAGCAAAGAAGAATGGGAGAGAATACTGGAGAGAAGATGGTCTATTGAT
20	TCCTATCATCCAGGTCGAGCAAAGAAGAATGGGAGAGAATACTGGAGAGAAGATGGTCTATTGAT
21	TCCTATCATCCAGGTCGAGCAAAGAAGAATGGGAGAGAATACTGGAGAGAAGATGGTCTATTGAT
22	TCCTATCATCCAGGTCGAGCAAAGAAGAATGGGAGAGAATACTGGAGAGAAGATGGTCTATTGAT
23	TCCTATCATCCAGGTCGAGCAAAGAAGAATGGGAGAGAATACTGGAGAGAAGATGGTCTATTGAT
24	TCCTATCATCCAGGTCGAGCAAAGAAGAATGGGAGAGAATACTGGAGAGAAGATGGTCTATTGAT
25	TCCTATCATCCAGGTCGAGCAAAGAAGAATGGGAGAGAATACTGGAGAGAAGATGGTCTATTGAT
26	TCCTATCATCCAGGTCGAGCAAAGAAGAATGGGAGAGAATACTGGAGAGAAGATGGTCTATTGAT
27	TCCTATCATCCAGGTCGAGCAAAGAAGAATGGGAGAGAATACTGGAGAGAAGATGGTCTATTGAT
28	TCCTATCATCCAGGTCGAGCAAAGAAGAATGGGAGAGAATACTGGAGAGAAGATGGTCTATTGAT
29	TCCTATCATCCAGGTCGAGCAAAGAAGAATGGGAGAGAATACTGGAGAGAAGATGGTCTATTGAT
30	TCCTATCATCCAGGTCGAGCAAAGAAGAATGGGAGAGAATACTGGAGAGAAGATGGTCTATTGAT
31	TCCTATCATCCAGGTCGAGCAAAGAAGAATGGGAGAGAATACTGGAGAGAAGATGGTCTATTGAT
32	TCCTATCATCCAGGTCGAGCAAAGAAGAATGGGAGAGAATACTGGAGAGAAGATGGTCTATTGAT
33	TCCTATCATCCAGGTCGAGCAAAGAAGAATGGGAGAGAATACTGGAGAGAAGATGGTCTATTGAT
34	TCCTATCATCCAGGTCGAGCAAAGAAGAATGGGAGAGAATACTGGAGAGAAGATGGTCTATTGAT
35	TCCTATCATCCAGGTCGAGCAAAGAAGAATGGGAGAGAATACTGGAGAGAAGATGGTCTATTGAT
36	TCCTATCATCCAGGTCGAGCAAAGAAGAATGGGAGAGAATACTGGAGAGAAGATGGTCTATTGAT
37	TCCTATCATCCAGGTCGAGCAAAGAAGAATGGGAGAGAATACTGGAGAGAAGATGGTCTATTGAT
38	TCCTATCATCCAGGTCGAGCAAAGAAGAATGGGAGAGAATACTGGAGAGAAGATGGTCTATTGAT
39	TCCTATCATCCAGGTCGAGCAAAGAAGAATGGGAGAGAATACTGGAGAGAAGATGGTCTATTGAT
40	TCCTATCATCCAGGTCGAGCAAAGAAGAATGGGAGAGAATACTGGAGAGAAGATGGTCTATTGAT
41	TCCTATCATCCAGGTCGAGCAAAGAAGAATGGGAGAGAATACTGGAGAGAAGATGGTCTATTGAT
42	TCCTATCATCCAGGTCGAGCAAAGAAGAATGGGAGAGAATACTGGAGAGAAGATGGTCTATTGAT

1180 1190 1200 1210 1220 1230

	GGGCTTCCAGAAGAACTAGAAGGTGCTTTGTACTTAAGCTACAGTGACTTACACCCACAACTCAA
1	GGGCTTCCAGAAGAACTAGAAGGTGCTTTGTACTTAAGCTACAGTGACTTACACCCACAACTGAA
2	GGGCTTCCAGAAGAACTAGAAGGTGCTTTGTACTTAAGCTACAGTGACTTACACCCACAACTGAA
3	GGGCTTCCAGAAGAACTAGAAGGTGCTTTGTACTTAAGCTACAGTGACTTACACCCACAACTGAA
4	GGGCTTCCAAAAGAACTAGAAGGTGCTTTGTACTTAAGCTACTGTGACTTACACCCACAACTGAA
5	GGGCTTCCAAAAGAACTAGAAGGTGCTTTGTACTTAAGCTACTGTGACTTACACCCACAACTGAA
6	GGGCTTCCAGAAGAACTAGAAGGTGCTTTGTACTTAAGCTACAGTGACTTACACCCACAACTCAA
7	GGGCTTCCAGAAGAACTAGAAGGTGCTCTGTACTTAAGCTACAGTGACTTACACCCACAACTGAA
8	GGGCTTCCAGAAGAACTAGAAGGTGCTTTGTACTTAAGCTACAGTGACTTACACCCACAACTCAA
9	GGGCTTCCAGAAGAACTAGAAGGTGCTTTGTACTTAAGCTACAGTGACTTACACCCACAACTCAA
10	GGGCTTCCAGAAGAACTAGAAGGTGCTTTGTACTTAAGCTACAGTGACTTACACCCACAACTCAA
11	GGGCTTCCAGAAGAACTAGAAGGTGCTTTGTACTTAAGCTACAGTGACTTACACCCACAACTCAA
12	GGGCTTCCAGAAGAACTAGAAGGTGCTTTGTACTTAAGCTACAGTGACTTACACCCACAACTCAA
13	GGGCTTCCAGAAGAACTAGAAGGTGCTTTGTACTTAAGCTACAGTGACTTACACCCACAACTCAA
14	GGGCTTCCAGAAGAACTAGAAGGTGCTTTGTACTTAAGCTACAGTGACTTACACCCACAACTCAA
15	GGGCTTCCAGAAGAACTAGAAGGTGCTTTGTACTTAAGCTACAGTGACTTACACCCACAACTCAA
16	GGGCTTCCAGAAGAACTAGAAGGTGCTTTGTACTTAAGCTACAGTGACTTACACCCACAACTCAA
17	GGGCTTCCAGAAGAACTAGAAGGTGCTTTGTACTTAAGCTACAGTGACTTACACCCACAACTCAA
18	GGGCTTCCAGAAGAACTAGAAGGTGCTTTGTACTTAAGCTACAGTGACTTACACCCACAACTCAA
19	GGGCTTCCAGAAGAACTAGAAGGTGCTTTGTACTTAAGCTACAGTGACTTACACCCACAACTCAA
20	GGGCTTCCAGAAGAACTAGAAGGTGCTTTGTACTTAAGCTACAGTGACTTACACCCACAACTCAA
21	GGGCTTCCAGAAGAACTAGAAGGTGCTTTGTACTTAAGCTACAGTGACTTACACCCACAACTCAA
22	GGGCTTCCAGAAGAACTAGAAGGTGCTTTGTACTTAAGCTACAGTGACTTACACCCACAACTCAA
23	GGGCTTCCAGAAGAACTAGAAGGTGCTTTGTACTTAAGCTACAGTGACTTACACCCACAACTCAA
24	GGGCTTCCAGAAGAACTAGAAGGTGCTTTGTACTTAAGCTACAGTGACTTACACCCACAACTCAA
25	GGGCTTCCAGAAGAACTAGAAGGTGCTTTGTACTTAAGCTACAGTGACTTACACCCACAACTCAA
26	GGGCTTCCAGAAGAACTAGAAGGTGCTTTGTACTTAAGCTACAGTGACTTACACCCACAACTCAA
27	GGGCTTCCAGAAGAACTAGAAGGTGCTTTGTACTTAAGCTACAGTGACTTACACCCACAACTCAA
28	GGGCTTCCAGAAGAACTAGAAGGTGCTTTGTACTTAAGCTACAGTGACTTACACCCACAACTCAA
29	GGGCTTCCAGAAGAACTAGAAGGTGCTTTGTACTTAAGCTACAGTGACTTACACCCACAACTCAA
30	GGGCTTCCAGAAGAACTAGAAGGTGCTTTGTACTTAAGCTACAGTGACTTACACCCACAACTCAA
31	GGGCTTCCAGAAGAACTAGAAGGTGCTTTGTACTTAAGCTACAGTGACTTACACCCACAACTCAA
32	GGGCTTCCAGAAGAACTAGAAGGTGCTTTGTACTTAAGCTACAGTGACTTACACCCACAACTCAA
33	GGGCTTCCAGAAGAACTAGAAGGTGCTTTGTACTTAAGCTACAGTGACTTACACCCACAACTCAA
34	GGGCTTCCAGAAGAACTAGAAGGTGCTTTGTACTTAAGCTACAGTGACTTACACCCACAACTCAA
35	GGGCTTCCAGAAGAACTAGAAGGTGCTTTGTACTTAAGCTACAGTGACTTACACCCACAACTCAA
36	GGGCTTCCAGAAGAACTAGAAGGTGCTTTGTACTTAAGCTACAGTGACTTACACCCACAACTCAA
37	GGGCTTCCAGAAGAACTAGAAGGTGCTTTGTACTTAAGCTACAGTGACTTACACCCACAACTCAA
38	GGGCTTCCAGAAGAACTAGAAGGTGCTTTGTACTTAAGCTACAGTGACTTACACCCACAACTCAA
39	GGGCTTCCAGAAGAACTAGAAGGTGCTTTGTACTTAAGCTACAGTGACTTACACCCACAACTCAA
40	GGGCTTCCAGAAGAACTAGAAGGTGCTTTGTACTTAAGCTACAGTGACTTACACCCACAACTCAA
41	GGGCTTCCAGAAGAACTAGAAGGTGCTTTGTACTTAAGCTACAGTGACTTACACCCACAACTCAA
42	GGGCTTCCAGAAGAACTAGAAGGTGCTTTGTACTTAAGCTACAGTGACTTACACCCACAACTCAA

	1240 1250 1260 1270 1280 1290 1300
	ACAGTGCTTCCTCTGCTGTGCCCTGTTGCCTCAGAATTTCGATATTCACCGAGATGTCACATACT
1 ➨	ACAGTGCTTCCTCTGGTGTGCCTTGTTGCCCCAGAATTTCGATATTCACCGAGATGTCACATACT
2 ➨	ACAGTGCTTCCTCTGGTGTGCCTTGTTGCCCCAGAATTTCGATATTCACCGAGATGTCACATACT
3 ➨	ACAGTGCTTCCTCTGGTGTGCCTTGTTGCCCCAGAATTTCGATATTCACCGAGATGTCACATACT
4 ➨	ACAGTGCTTCCTCTGGTGTGCCTTGTTGCCCCAGAATTTCGATATTCACCGAGATGTCACATACT
5 ➨	ACAGTGCTTCCTCTGGTGTGCCTTGTTGCCCCAGAATTTCGATATTCACCGAGATGTCACATACT
6 ➨	ACAGTGCTTCCTCTGCTGTGCCCTGTTGCCTCAGAATTTCGATATTCACCGAGATGTCACATACT
7 ➨	ACAGTGCTTCCTCTGGTGTGCCTTGTTGCCCCAGAATTTCGATATTCACCGAGATGTCACATACT
8 ➨	ACAGTGCTTCCTCTGCTGTGCCCTGTTGCCTCAGAATTTCGATATTCACCGAGATGTCACATACT
9 ➨	ACAGTGCTTCCTCTGCTGTGCCCTGTTGCCTCAGAATTTCGATATTCACCGAGATGTCACATACT
10 ➨	ACAGTGCTTCCTCTGCTGTGCCCTGTTGCCTCAGAATTTCGATATTCACCGAGATGTCACATACT
11 ➨	ACAGTGCTTCCTCTGCTGTGCCCTGTTGCCTCAGAATTTCGATATTCACCGAGATGTCACATACT
12 ➨	ACAGTGCTTCCTCTGCTGTGCCCTGTTGCCTCAGAATTTCGATATTCACCGAGATGTCACATACT
13 ➨	ACAGTGCTTCCTCTGCTGTGCCCTGTTGCCTCAGAATTTCGATATTCACCGAGATGTCACATACT
14 ➨	ACAGTGCTTCCTCTGCTGTGCCCTGTTGCCTCAGAATTTCGATATTCACCGAGATGTCACATACT
15 ➨	ACAGTGCTTCCTCTGCTGTGCCCTGTTGCCTCAGAATTTCGATATTCACCGAGATGTCACATACT
16 ➨	ACAGTGCTTCCTCTGCTGTGCCCTGTTGCCTCAGAATTTCGATATTCACCGAGATGTCACATACT
17 ➨	ACAGTGCTTCCTCTGCTGTGCCCTGTTGCCTCAGAATTTCGATATTCACCGAGATGTCACATACT
18 ➨	ACAGTGCTTCCTCTGCTGTGCCCTGTTGCCTCAGAATTTCGATATTCACCGAGATGTCACATACT
19 ➨	ACAGTGCTTCCTCTGCTGTGCCCTGTTGCCTCAGAATTTCGATATTCACCGAGATGTCACATACT
20 ➨	ACAGTGCTTCCTCTGCTGTGCCCTGTTGCCTCAGAATTTCGATATTCACCGAGATGTCACATACT
21 ➨	ACAGTGCTTCCTCTGCTGTGCCCTGTTGCCTCAGAATTTCGATATTCACCGAGATGTCACATACT
22 ➨	ACAGTGCTTCCTCTGCTGTGCCCTGTTGCCTCAGAATTTCGATATTCACCGAGATGTCACATACT
23 ➨	ACAGTGCTTCCTCTGCTGTGCCCTGTTGCCTCAGAATTTCGATATTCACCGAGATGTCACATACT
24 ➨	ACAGTGCTTCCTCTGCTGTGCCCTGTTGCCTCAGAATTTCGATATTCACCGAGATGTCACATACT
25 ➨	ACAGTGCTTCCTCTGCTGTGCCCTGTTGCCTCAGAATTTCGATATTCACCGAGATGTCACATACT
26 ➨	ACAGTGCTTCCTCTGCTGTGCCCTGTTGCCTCAGAATTTCGATATTCACCGAGATGTCACATACT
27 ➨	ACAGTGCTTCCTCTGCTGTGCCCTGTTGCCTCAGAATTTCGATATTCACCGAGATGTCACATACT
28 ➨	ACAGTGCTTCCTCTGCTGTGCCCTGTTGCCTCAGAATTTCGATATTCACCGAGATGTCACATACT
29 ➨	ACAGTGCTTCCTCTGCTGTGCCCTGTTGCCTCAGAATTTCGATATTCACCGAGATGTCACATACT
30 ➨	ACAGTGCTTCCTCTGCTGTGCCCTGTTGCCTCAGAATTTCGATATTCACCGAGATGTCACATACT
31 ➨	ACAGTGCTTCCTCTGCTGTGCCCTGTTGCCTCAGAATTTCGATATTCACCGAGATGTCACATACT
32 ➨	ACAGTGCTTCCTCTGCTGTGCCCTGTTGCCTCAGAATTTCGATATTCACCGAGATGTCACATACT
33 ➨	ACAGTGCTTCCTCTGCTGTGCCCTGTTGCCTCAGAATTTCGATATTCACCGAGATGTCACATACT
34 ➨	ACAGTGCTTCCTCTGCTGTGCCCTGTTGCCTCAGAATTTCGATATTCACCGAGATGTCACATACT
35 ➨	ACAGTGCTTCCTCTGCTGTGCCCTGTTGCCTCAGAATTTCGATATTCACCGAGATGTCACATACT
36 ➨	ACAGTGCTTCCTCTGCTGTGCCCTGTTGCCTCAGAATTTCGATATTCACCGAGATGTCACATACT
37 ➨	ACAGTGCTTCCTCTGCTGTGCCCTGTTGCCTCAGAATTTCGATATTCACCGAGATGTCACATACT
38 ➨	ACAGTGCTTCCTCTGCTGTGCCCTGTTGCCTCAGAATTTCGATATTCACCGAGATGTCACATACT
39 ➨	ACAGTGCTTCCTCTGCTGTGCCCTGTTGCCTCAGAATTTCGATATTCACCGAGATGTCACATACT
40 ➨	ACAGTGCTTCCTCTGCTGTGCCCTGTTGCCTCAGAATTTCGATATTCACCGAGATGTCACATACT
41 ➨	ACAGTGCTTCCTCTGCTGTGCCCTGTTGCCTCAGAATTTCGATATTCACCGAGATGTCACATACT
42 ➨	ACAGTGCTTCCTCTGCTGTGCCCTGTTGCCTCAGAATTTCGATATTCACCGAGATGTCACATACT

	1310 1320 1330 1340 1350 1360
	GGTGGATTGCTGAAGGTTTTGTGAAGGAAGAGGGCAGCGGACCGATACATAACATTGCCGAAGAT
1	GGTGGATTGCTGAAGGTCTTGTGAAGGAAGAGACCAGTGGACCAATACATAACGTCGCCGAAGAT
2	GGTGGATTGCTGAAGGTCTTGTGAAGGAAGAGACCAGTGGACCAATACATAACGTCGCCGAAGAT
3	GGTGGATTGCTGAAGGTCTTGTGAAGGAAGAGACCAGTGGACCAATACATAACGTCGCCGAAGAT
4	GGTGGATTTCTGAAGGTCTTGTGAAGGAAGAGACCAGTGGACCAATACATAACGTCGCCGAAGAT
5	GGTGGATTTCTGAAGGTCTTGTGAAGGAAGAGACCAGTGGACCAATACATAACGTCGCCGAAGAT
6	GGTGGATTGCTGAAGGTTTTGTGAAGGAAGAGGGCAGCGGACCGATACATAACATTGCCGAAGAT
7	GGTGGATTGCTGAAGGTCTTGTGAAGGAAGAGACCAGTGGACCAATACATAACGTCGCCGAAGAT
8	GGTGGATTGCTGAAGGTTTTGTGAAGGAAGAGGGCAGCGGACCGATACATAACATTGCCGAAGAT
9	GGTGGATTGCTGAAGGTTTTGTGAAGGAAGAGGGCAGCGGACCGATACATAACATTGCCGAAGAT
10	GGTGGATTGCTGAAGGTTTTGTGAAGGAAGAGGGCAGCGGACCGATACATAACATTGCCGAAGAT
11	GGTGGATTGCTGAAGGTTTTGTGAAGGAAGAGGGCAGCGGACCGATACATAACATTGCCGAAGAT
12	GGTGGATTGCTGAAGGTTTTGTGAAGGAAGAGGGCAGCGGACCGATACATAACATTGCCGAAGAT
13	GGTGGATTGCTGAAGGTTTTGTGAAGGAAGAGGGCAGCGGACCGATACATAACATTGCCGAAGAT
14	GGTGGATTGCTGAAGGTTTTGTGAAGGAAGAGGGCAGCGGACCGATACATAACATTGCCGAAGAT
15	GGTGGATTGCTGAAGGTTTTGTGAAGGAAGAGGGCAGCGGACCGATACATAACATTGCCGAAGAT
16	GGTGGATTGCTGAAGGTTTTGTGAAGGAAGAGGGCAGCGGACCGATACATAACATTGCCGAAGAT
17	GGTGGATTGCTGAAGGTTTTGTGAAGGAAGAGGGCAGCGGACCGATACATAACATTGCCGAAGAT
18	GGTGGATTGCTGAAGGTTTTGTGAAGGAAGAGGGCAGCGGACCGATACATAACATTGCCGAAGAT
19	GGTGGATTGCTGAAGGTTTTGTGAAGGAAGAGGGCAGCGGACCGATACATAACATTGCCGAAGAT
20	GGTGGATTGCTGAAGGTTTTGTGAAGGAAGAGGGCAGCGGACCGATACATAACATTGCCGAAGAT
21	GGTGGATTGCTGAAGGTTTTGTGAAGGAAGAGGGCAGCGGACCGATACATAACATTGCCGAAGAT
22	GGTGGATTGCTGAAGGTTTTGTGAAGGAAGAGGGCAGCGGACCGATACATAACATTGCCGAAGAT
23	GGTGGATTGCTGAAGGTTTTGTGAAGGAAGAGGGCAGCGGACCGATACATAACATTGCCGAAGAT
24	GGTGGATTGCTGAAGGTTTTGTGAAGGAAGAGGGCAGCGGACCGATACATAACATTGCCGAAGAT
25	GGTGGATTGCTGAAGGTTTTGTGAAGGAAGAGGGCAGCGGACCGATACATAACATTGCCGAAGAT
26	GGTGGATTGCTGAAGGTTTTGTGAAGGAAGAGGGCAGCGGACCGATACATAACATTGCCGAAGAT
27	GGTGGATTGCTGAAGGTTTTGTGAAGGAAGAGGGCAGCGGACCGATACATAACATTGCCGAAGAT
28	GGTGGATTGCTGAAGGTTTTGTGAAGGAAGAGGGCAGCGGACCGATACATAACATTGCCGAAGAT
29	GGTGGATTGCTGAAGGTTTTGTGAAGGAAGAGGGCAGCGGACCGATACATAACATTGCCGAAGAT
30	GGTGGATTGCTGAAGGTTTTGTGAAGGAAGAGGGCAGCGGACCGATACATAACATTGCCGAAGAT
31	GGTGGATTGCTGAAGGTTTTGTGAAGGAAGAGGGCAGCGGACCGATACATAACATTGCCGAAGAT
32	GGTGGATTGCTGAAGGTTTTGTGAAGGAAGAGGGCAGCGGACCGATACATAACATTGCCGAAGAT
33	GGTGGATTGCTGAAGGTTTTGTGAAGGAAGAGGGCAGCGGACCGATACATAACATTGCCGAAGAT
34	GGTGGATTGCTGAAGGTTTTGTGAAGGAAGAGGGCAGCGGACCGATACATAACATTGCCGAAGAT
35	GGTGGATTGCTGAAGGTTTTGTGAAGGAAGAGGGCAGCGGACCGATACATAACATTGCCGAAGAT
36	GGTGGATTGCTGAAGGTTTTGTGAAGGAAGAGGGCAGCGGACCGATACATAACATTGCCGAAGAT
37	GGTGGATTGCTGAAGGTTTTGTGAAGGAAGAGGGCAGCGGACCGATACATAACATTGCCGAAGAT
38	GGTGGATTGCTGAAGGTTTTGTGAAGGAAGAGGGCAGCGGACCGATACATAACATTGCCGAAGAT
39	GGTGGATTGCTGAAGGTTTTGTGAAGGAAGAGGGCAGCGGACCGATACATAACATTGCCGAAGAT
40	GGTGGATTGCTGAAGGTTTTGTGAAGGAAGAGGGCAGCGGACCGATACATAACATTGCCGAAGAT
41	GGTGGATTGCTGAAGGTTTTGTGAAGGAAGAGGGCAGCGGACCGATACATAACATTGCCGAAGAT
42	GGTGGATTGCTGAAGGTTTTGTGAAGGAAGAGGGCAGCGGACCGATACATAACATTGCCGAAGAT

1370 1380 1390 1400 1410 1420 1430

	TACTACCATGAGCTGATCAAGAGGAATCTGCTACAGGCAAGGCCAGAGTATGTCGACAAGGGAGT
1 ➨	TACTACCATGAGCTGATCAAGAGGAATCTGCTACAGGCAAGGCCAGAGTATGTCGACAAGGGAAT
2 ➨	TACTACCATGAGCTGATCAAGAGGAATCTGCTACAGGCAAGGCCAGAGTATGTCGACAAGGGAAT
3 ➨	TACTACCATGAGCTGATCAAGAGGAATCTGCTACAGGCAAGGCCAGAGTATGTCGACAAGGGAAT
4 ➨	TACTACCATGAGCTGATCAAGAGGAATCTGCTACAGGCAAGGCCAGAGTATGTCGACAAGGGAAT
5 ➨	TACTACCATGAGCTGATCAAGAGGAATCTGCTACAGGCAAGGCCAGAGTATGTCGACAAGGGAAT
6 ➨	TACTACCATGAGCTGATCAAGAGGAATCTGCTACAGGCAAGGCCAGAGTATGTCGACAAGGGAAT
7 ➨	TACTACCATGAGCTGATCAAGAGGAACCTGCTACAGGCAAGGCCAGAGTATGTCGACAAGGGAGT
8 ➨	TACTACCATGAGCTGATCAAGAGGAATCTGCTACAGGCAAGGCCAGAGTATGTCGACAAGGGAGT
9 ➨	TACTACCATGAGCTGATCAAGAGGAATCTGCTACAGGCAAGGCCAGAGTATGTCGACAAGGGAGT
10 ➨	TACTACCATGAGCTGATCAAGAGGAATCTGCTACAGGCAAGGCCAGAGTATGTCGACAAGGGAGT
11 ➨	TACTACCATGAGCTGATCAAGAGGAATCTGCTACAGGCAAGGCCAGAGTATGTCGACAAGGGAGT
12 ➨	TACTACCATGAGCTGATCAAGAGGAATCTGCTACAGGCAAGGCCAGAGTATGTCGACAAGGGAGT
13 ➨	TACTACCATGAGCTGATCAAGAGGAATCTGCTACAGGCAAGGCCAGAGTATGTCGACAAGGGAGT
14 ➨	TACTACCATGAGCTGATCAAGAGGAATCTGCTACAGGCAAGGCCAGAGTATGTCGACAAGGGAGT
15 ➨	TACTACCATGAGCTGATCAAGAGGAATCTGCTACAGGCAAGGCCAGAGTATGTCGACAAGGGAGT
16 ➨	TACTACCATGAGCTGATCAAGAGGAATCTGCTACAGGCAAGGCCAGAGTATGTCGACAAGGGAGT
17 ➨	TACTACCATGAGCTGATCAAGAGGAATCTGCTACAGGCAAGGCCAGAGTATGTCGACAAGGGAGT
18 ➨	TACTACCATGAGCTGATCAAGAGGAATCTGCTACAGGCAAGGCCAGAGTATGTCGACAAGGGAGT
19 ➨	TACTACCATGAGCTGATCAAGAGGAATCTGCTACAGGCAAGGCCAGAGTATGTCGACAAGGGAGT
20 ➨	TACTACCATGAGCTGATCAAGAGGAATCTGCTACAGGCAAGGCCAGAGTATGTCGACAAGGGAGT
21 ➨	TACTACCATGAGCTGATCAAGAGGAATCTGCTACAGGCAAGGCCAGAGTATGTCGACAAGGGAGT
22 ➨	TACTACCATGAGCTGATCAAGAGGAATCTGCTACAGGCAAGGCCAGAGTATGTCGACAAGGGAGT
23 ➨	TACTACCATGAGCTGATCAAGAGGAATCTGCTACAGGCAAGGCCAGAGTATGTCGACAAGGGAGT
24 ➨	TACTACCATGAGCTGATCAAGAGGAATCTGCTACAGGCAAGGCCAGAGTATGTCGACAAGGGAGT
25 ➨	TACTACCATGAGCTGATCAAGAGGAATCTGCTACAGGCAAGGCCAGAGTATGTCGACAAGGGAGT
26 ➨	TACTACCATGAGCTGATCAAGAGGAATCTGCTACAGGCAAGGCCAGAGTATGTCGACAAGGGAGT
27 ➨	TACTACCATGAGCTGATCAAGAGGAATCTGCTACAGGCAAGGCCAGAGTATGTCGACAAGGGAGT
28 ➨	TACTACCATGAGCTGATCAAGAGGAATCTGCTACAGGCAAGGCCAGAGTATGTCGACAAGGGAGT
29 ➨	TACTACCATGAGCTGATCAAGAGGAATCTGCTACAGGCAAGGCCAGAGTATGTCGACAAGGGAGT
30 ➨	TACTACCATGAGCTGATCAAGAGGAATCTGCTACAGGCAAGGCCAGAGTATGTCGACAAGGGAGT
31 ➨	TACTACCATGAGCTGATCAAGAGGAATCTGCTACAGGCAAGGCCAGAGTATGTCGACAAGGGAGT
32 ➨	TACTACCATGAGCTGATCAAGAGGAATCTGCTACAGGCAAGGCCAGAGTATGTCGACAAGGGAGT
33 ➨	TACTACCATGAGCTGATCAAGAGGAATCTGCTACAGGCAAGGCCAGAGTATGTCGACAAGGGAGT
34 ➨	TACTACCATGAGCTGATCAAGAGGAATCTGCTACAGGCAAGGCCAGAGTATGTCGACAAGGGAGT
35 ➨	TACTACCATGAGCTGATCAAGAGGAATCTGCTACAGGCAAGGCCAGAGTATGTCGACAAGGGAGT
36 ➨	TACTACCATGAGCTGATCAAGAGGAATCTGCTACAGGCAAGGCCAGAGTATGTCGACAAGGGAGT
37 ➨	TACTACCATGAGCTGATCAAGAGGAATCTGCTACAGGCAAGGCCAGAGTATGTCGACAAGGGAGT
38 ➨	TACTACCATGAGCTGATCAAGAGGAATCTGCTACAGGCAAGGCCAGAGTATGTCGACAAGGGAGT
39 ➨	TACTACCATGAGCTGATCAAGAGGAATCTGCTACAGGCAAGGCCAGAGTATGTCGACAAGGGAGT
40 ➨	TACTACCATGAGCTGATCAAGAGGAATCTGCTACAGGCAAGGCCAGAGTATGTCGACAAGGGAGT
41 ➨	TACTACCATGAGCTGATCAAGAGGAATCTGCTACAGGCAAGGCCAGAGTATGTCGACAAGGGAGT
42 ➨	TACTACCATGAGCTGATCAAGAGGAATCTGCTACAGGCAAGGCCAGAGTATGTCGACAAGGGAGT

	1440 1450 1460 1470 1480 1490
	ATCGACAATGCATGACCTGTTGAGGCAACTTGGCCAATTTCTGAAAAGAAATGAAGCCATCTTCA
1	ATCGACAATGCATGACCTGTTAAGGCAACTTGGCCAATTTCTGACAAGGAATGAAGCCGTCTTCA
2	ATCGACAATGCATGACCTGTTAAGGCAACTTGGCCAATTTCTGACAAGGAATGAAGCCGTCTTCA
3	ATCGACAATGCATGACCTGTTAAGGCAACTTGGCCAATTTCTGACAAGGAATGAAGCCGTCTTCA
4	ATCAACAATGCATGACCTGTTAAGGCAACTTGGCCAGTTTCTGACAAGGAATGAAGCCGTCTTCA
5	ATCAACAATGCATGACCTGTTAAGGCAACTTGGCCAGTTTCTGACAAGGAATGAAGCCGTCTTCA
6	ATCGACAATGCATGACCTGTTAAGGCAACTTGGCCAATTTCTGACAAGGAATGAAGCCGTCTTCA
7	ATCGACAATGCATGACCTGTTGAGGCAACTTGGCCAATTTCTGACAAGAAATGAAGCCATCTTCA
8	ATCGACAATGCATGACCTGTTGAGGCAACTTGGCCAATTTCTGACAAGAAATGAAGCCATCTTCA
9	ATCGACAATGCATGACCTGTTGAGGCAACTTGGCCAATTTCTGACAAGAAATGAAGCCATCTTCA
10	ATCGACAATGCATGACCTGTTGAGGCAACTTGGCCAATTTCTGACAAGAAATGAAGCCATCTTCA
11	ATCGACAATGCATGACCTGTTGAGGCAACTTGGCCAATTTCTGAAAAGAAATGAAGCCATCTTCA
12	ATCGACAATGCATGACCTGTTGAGGCAACTTGGCCAATTTCTGAAAAGAAATGAAGCCATCTTCA
13	ATCGACAATGCATGACCTGTTGAGGCAACTTGGCCAATTTCTGAAAAGAAATGAAGCCATCTTCA
14	ATCGACAATGCATGACCTGTTGAGGCAACTTGGCCAATTTCTGAAAAGAAATGAAGCCATCTTCA
15	ATCGACAATGCATGACCTGTTGAGGCAACTTGGCCAATTTCTGAAAAGAAATGAAGCCATCTTCA
16	ATCGACAATGCATGACCTGTTGAGGCAACTTGGCCAATTTCTGAAAAGAAATGAAGCCATCTTCA
17	ATCGACAATGCATGACCTGTTGAGGCAACTTGGCCAATTTCTGAAAAGAAATGAAGCCATCTTCA
18	ATCGACAATGCATGACCTGTTGAGGCAACTTGGCCAATTTCTGAAAAGAAATGAAGCCATCTTCA
19	ATCGACAATGCATGACCTGTTGAGGCAACTTGGCCAATTTCTGAAAAGAAATGAAGCCATCTTCA
20	ATCGACAATGCATGACCTGTTGAGGCAACTTGGCCAATTTCTGAAAAGAAATGAAGCCATCTTCA
21	ATCGACAATGCATGACCTGTTGAGGCAACTTGGCCAATTTCTGAAAAGAAATGAAGCCATCTTCA
22	ATCGACAATGCATGACCTGTTGAGGCAACTTGGCCAATTTCTGAAAAGAAATGAAGCCATCTTCA
23	ATCGACAATGCATGACCTGTTGAGGCAACTTGGCCAATTTCTGAAAAGAAATGAAGCCATCTTCA
24	ATCGACAATGCATGACCTGTTGAGGCAACTTGGCCAATTTCTGAAAAGAAATGAAGCCATCTTCA
25	ATCGACAATGCATGACCTGTTGAGGCAACTTGGCCAATTTCTGAAAAGAAATGAAGCCATCTTCA
26	ATCGACAATGCATGACCTGTTGAGGCAACTTGGCCAATTTCTGAAAAGAAATGAAGCCATCTTCA
27	ATCGACAATGCATGACCTGTTGAGGCAACTTGGCCAATTTCTGAAAAGAAATGAAGCCATCTTCA
28	ATCGACAATGCATGACCTGTTGAGGCAACTTGGCCAATTTCTGAAAAGAAATGAAGCCATCTTCA
29	ATCGACAATGCATGACCTGTTGAGGCAACTTGGCCAATTTCTGAAAAGAAATGAAGCCATCTTCA
30	ATCGACAATGCATGACCTGTTGAGGCAACTTGGCCAATTTCTGAAAAGAAATGAAGCCATCTTCA
31	ATCGACAATGCATGACCTGTTGAGGCAACTTGGCCAATTTCTGAAAAGAAATGAAGCCATCTTCA
32	ATCGACAATGCATGACCTGTTGAGGCAACTTGGCCAATTTCTGAAAAGAAATGAAGCCATCTTCA
33	ATCGACAATGCATGACCTGTTGAGGCAACTTGGCCAATTTCTGAAAAGAAATGAAGCCATCTTCA
34	ATCGACAATGCATGACCTGTTGAGGCAACTTGGCCAATTTCTGAAAAGAAATGAAGCCATCTTCA
35	ATCGACAATGCATGACCTGTTGAGGCAACTTGGCCAATTTCTGAAAAGAAATGAAGCCATCTTCA
36	ATCGACAATGCATGACCTGTTGAGGCAACTTGGCCAATTTCTGAAAAGAAATGAAGCCATCTTCA
37	ATCGACAATGCATGACCTGTTGAGGCAACTTGGCCAATTTCTGAAAAGAAATGAAGCCATCTTCA
38	ATCGACAATGCATGACCTGTTGAGGCAACTTGGCCAATTTCTGAAAAGAAATGAAGCCATCTTCA
39	ATCGACAATGCATGACCTGTTGAGGCAACTTGGCCAATTTCTGAAAAGAAATGAAGCCATCTTCA
40	ATCGACAATGCATGACCTGTTGAGGCAACTTGGCCAATTTCTGAAAAGAAATGAAGCCATCTTCA
41	ATCGACAATGCATGACCTGTTGAGGCAACTTGGCCAATTTCTGAAAAGAAATGAAGCCATCTTCA
42	ATCGACAATGCATGACCTGTTGAGGCAACTTGGCCAATTTCTGAAAAGAAATGAAGCCATCTTCA

	1500 1510 1520 1530 1540 1550 1560
	TGAACGAGAAGCGTGAACGTTGCCTTTGAAGTATTCGCCGACTAGGTGTCGGGAGCGCTGTTGAC
1 ➨	TGAACGAGAAGCGTGATCGTTGCCCTTCTAGTATTCGCCGATTAGGTGTCGGGAGCGCTGTTGAC
2 ➨	TGAACGAGAAGCGTGATCGTTGCCCTTCTAGTATTCGCCGATTAGGTGTCGGGAGCGCTGTTGAC
3 ➨	TGAACGAGAAGCGTGATCGTTGCCCTTCTAGTATTCGCCGATTAGGTGTCGGGAGCGCTGTTGAC
4 ➨	TGAACGAGAAGCGTGATCGTTGCCCTTCTAGTATTCGCCGATTAGGTGTCGGGAGCGCTGTTGAC
5 ➨	TGAACGAGAAGCGTGATCGTTGCCCTTCTAGTATTCGCCGATTAGGTGTCGGGAGCGCTGTTGAC
6 ➨	TGAACGAGAAGCGTGATCGTTGCCCTTCTAGTATTCGCCGATTAGGTGTCGGGAGCGCTGTTGAC
7 ➨	TGAACGAGAAGCGTGATCGTTGCCCTTGAAGTATTCGCCGACTAGGTGTCGGGAGCGCTGTTGAC
8 ➨	TGAACGAGAAGCGTGATCGTTGCCTTTGAAGTATTCGCCGACTAGGTGTCGGGAGCGCTGTTGAC
9 ➨	TGAACGAGAAGCGTGATCGTTGCCTTTGAAGTATTCGCCGACTAGGTGTCGGGAGCGCTGTTGAC
10 ➨	TGAACGAGAAGCGTGATCGTTGCCTTTGAAGTATTCGCCGACTAGGTGTCGGGAGCGCTGTTGAC
11 ➨	TGAACGAGAAGCGTGAACGTTGCCTTTGAAGTATTCGCCGACTAGGTGTCGGGAGCGCTGTTGAC
12 ➨	TGAACGAGAAGCGTGAACGTTGCCTTTGAAGTATTCGCCGACTAGGTGTCGGGAGCGCTGTTGAC
13 ➨	TGAACGAGAAGCGTGAACGTTGCCTTTGAAGTATTCGCCGACTAGGTGTCGGGAGCGCTGTTGAC
14 ➨	TGAACGAGAAGCGTGAACGTTGCCTTTGAAGTATTCGCCGACTAGGTGTCGGGAGCGCTGTTGAC
15 ➨	TGAACGAGAAGCGTGAACGTTGCCTTTGAAGTATTCGCCGACTAGGTGTCGGGAGCGCTGTTGAC
16 ➨	TGAACGAGAAGCGTGAACGTTGCCTTTGAAGTATTCGCCGACTAGGTGTCGGGAGCGCTGTTGAC
17 ➨	TGAACGAGAAGCGTGAACGTTGCCTTTGAAGTATTCGCCGACTAGGTGTCGGGAGCGCTGTTGAC
18 ➨	TGAACGAGAAGCGTGAACGTTGCCTTTGAAGTATTCGCCGACTAGGTGTCGGGAGCGCTGTTGAC
19 ➨	TGAACGAGAAGCGTGAACGTTGCCTTTGAAGTATTCGCCGACTAGGTGTCGGGAGCGCTGTTGAC
20 ➨	TGAACGAGAAGCGTGAACGTTGCCTTTGAAGTATTCGCCGACTAGGTGTCGGGAGCGCTGTTGAC
21 ➨	TGAACGAGAAGCGTGAACGTTGCCTTTGAAGTATTCGCCGACTAGGTGTCGGGAGCGCTGTTGAC
22 ➨	TGAACGAGAAGCGTGAACGTTGCCTTTGAAGTATTCGCCGACTAGGTGTCGGGAGCGCTGTTGAC
23 ➨	TGAACGAGAAGCGTGAACGTTGCCTTTGAAGTATTCGCCGACTAGGTGTCGGGAGCGCTGTTGAC
24 ➨	TGAACGAGAAGCGTGAACGTTGCCTTTGAAGTATTCGCCGACTAGGTGTCGGGAGCGCTGTTGAC
25 ➨	TGAACGAGAAGCGTGAACGTTGCCTTTGAAGTATTCGCCGACTAGGTGTCGGGAGCGCTGTTGAC
26 ➨	TGAACGAGAAGCGTGAACGTTGCCTTTGAAGTATTCGCCGACTAGGTGTCGGGAGCGCTGTTGAC
27 ➨	TGAACGAGAAGCGTGAACGTTGCCTTTGAAGTATTCGCCGACTAGGTGTCGGGAGCGCTGTTGAC
28 ➨	TGAACGAGAAGCGTGAACGTTGCCTTTGAAGTATTCGCCGACTAGGTGTCGGGAGCGCTGTTGAC
29 ➨	TGAACGAGAAGCGTGAACGTTGCCTTTGAAGTATTCGCCGACTAGGTGTCGGGAGCGCTGTTGAC
30 ➨	TGAACGAGAAGCGTGAACGTTGCCTTTGAAGTATTCGCCGACTAGGTGTCGGGAGCGCTGTTGAC
31 ➨	TGAACGAGAAGCGTGAACGTTGCCTTTGAAGTATTCGCCGACTAGGTGTCGGGAGCGCTGTTGAC
32 ➨	TGAACGAGAAGCGTGAACGTTGCCTTTGAAGTATTCGCCGACTAGGTGTCGGGAGCGCTGTTGAC
33 ➨	TGAACGAGAAGCGTGAACGTTGCCTTTGAAGTATTCGCCGACTAGGTGTCGGGAGCGCTGTTGAC
34 ➨	TGAACGAGAAGCGTGAACGTTGCCTTTGAAGTATTCGCCGACTAGGTGTCGGGAGCGCTGTTGAC
35 ➨	TGAACGAGAAGCGTGAACGTTGCCTTTGAAGTATTCGCCGACTAGGTGTCGGGAGCGCTGTTGAC
36 ➨	TGAACGAGAAGCGTGAACGTTGCCTTTGAAGTATTCGCCGACTAGGTGTCGGGAGCGCTGTTGAC
37 ➨	TGAACGAGAAGCGTGAACGTTGCCTTTGAAGTATTCGCCGACTAGGTGTCGGGAGCGCTGTTGAC
38 ➨	TGAACGAGAAGCGTGAACGTTGCCTTTGAAGTATTCGCCGACTAGGTGTCGGGAGCGCTGTTGAC
39 ➨	TGAACGAGAAGCGTGAACGTTGCCTTTGAAGTATTCGCCGACTAGGTGTCGGGAGCGCTGTTGAC
40 ➨	TGAACGAGAAGCGTGAACGTTGCCTTTGAAGTATTCGCCGACTAGGTGTCGGGAGCGCTGTTGAC
41 ➨	TGAACGAGAAGCGTGAACGTTGCCTTTGAAGTATTCGCCGACTAGGTGTCGGGAGCGCTGTTGAC
42 ➨	TGAACGAGAAGCGTGAACGTTGCCTTTGAAGTATTCGCCGACTAGGTGTCGGGAGCGCTGTTGAC

	1570 1580 1590 1600 1610 1620
	GAAATACCTTCTATAGAAGAGAAGAAGCGCCTACGGTGCCTCATTGTCTTGCATCACGACACATG
1 ➨	GAAATACCTTCTATAGAAGAGAAGAAGCGCCTACGGTGCCTCATTGTCTTGGATCACGACACATG
2 ➨	GAAATACCTTCTATAGAAGAGAAGAAGCGCCTACGGTGCCTCATTGTCTTGGATCACGACACATG
3 ➨	GAAATACCTTCTATAGAAGAGAAGAAGCGCCTACGGTGCCTCATTGTCTTGGATCACGACACATG
4 ➨	GAAATACCTTCTATAGAAGAGAAGAAGCGCCTACGGTGCCTCATTGTCTTGGATCACGACACATG
5 ➨	GAAATACCTTCTATAGAAGAGAAGAAGCGCCTACGGTGCCTCATTGTCTTGGATCACGACACATG
6 ➨	GAAATACCTTCTATAGAAGAGAAGAAGCGCCTACGGTGCCTCATTGTCTTGGATCACGACACATG
7 ➨	GAAATACCTTCTATAGAAGAGAAGAAGCGCCTACGGTGCCTCATTGTCTTGCATCACGACACATG
8 ➨	GAAATACCTTCTATAGAAGAGAAGAAGCGCCTACGGTGCCTCATTGTCTTGCATCACGACACATG
9 ➨	GAAATACCTTCTATAGAAGAGAAGAAGCGCCTACGGTGCCTCATTGTCTTGCATCACGACACATG
10 ➨	GAAATACCTTCTATAGAAGAGAAGAAGCGCCTACGGTGCCTCATTGTCTTGCATCACGACACATG
11 ➨	GAAATACCTTCTATAGAAGAGAAGAAGCGCCTACGGTGCCTCATTGTCTTGCATCACGACACATG
12 ➨	GAAATACCTTCTATAGAAGAGAAGAAGCGCCTACGGTGCCTCATTGTCTTGCATCACGACACATG
13 ➨	GAAATACCTTCTATAGAAGAGAAGAAGCGCCTACGGTGCCTCATTGTCTTGCATCACGACACATG
14 ➨	GAAATACCTTCTATAGAAGAGAAGAAGCGCCTACGGTGCCTCATTGTCTTGCATCACGACACATG
15 ➨	GAAATACCTTCTATAGAAGAGAAGAAGCGCCTACGGTGCCTCATTGTCTTGCATCACGACACATG
16 ➨	GAAATACCTTCTATAGAAGAGAAGAAGCGCCTACGGTGCCTCATTGTCTTGCATCACGACACATG
17 ➨	GAAATACCTTCTATAGAAGAGAAGAAGCGCCTACGGTGCCTCATTGTCTTGCATCACGACACATG
18 ➨	GAAATACCTTCTATAGAAGAGAAGAAGCGCCTACGGTGCCTCATTGTCTTGCATCACGACACATG
19 ➨	GAAATACCTTCTATAGAAGAGAAGAAGCGCCTACGGTGCCTCATTGTCTTGCATCACGACACATG
20 ➨	GAAATACCTTCTATAGAAGAGAAGAAGCGCCTACGGTGCCTCATTGTCTTGCATCACGACACATG
21 ➨	GAAATACCTTCTATAGAAGAGAAGAAGCGCCTACGGTGCCTCATTGTCTTGCATCACGACACATG
22 ➨	GAAATACCTTCTATAGAAGAGAAGAAGCGCCTACGGTGCCTCATTGTCTTGCATCACGACACATG
23 ➨	GAAATACCTTCTATAGAAGAGAAGAAGCGCCTACGGTGCCTCATTGTCTTGCATCACGACACATG
24 ➨	GAAATACCTTCTATAGAAGAGAAGAAGCGCCTACGGTGCCTCATTGTCTTGCATCACGACACATG
25 ➨	GAAATACCTTCTATAGAAGAGAAGAAGCGCCTACGGTGCCTCATTGTCTTGCATCACGACACATG
26 ➨	GAAATACCTTCTATAGAAGAGAAGAAGCGCCTACGGTGCCTCATTGTCTTGCATCACGACACATG
27 ➨	GAAATACCTTCTATAGAAGAGAAGAAGCGCCTACGGTGCCTCATTGTCTTGCATCACGACACATG
28 ➨	GAAATACCTTCTATAGAAGAGAAGAAGCGCCTACGGTGCCTCATTGTCTTGCATCACGACACATG
29 ➨	GAAATACCTTCTATAGAAGAGAAGAAGCGCCTACGGTGCCTCATTGTCTTGCATCACGACACATG
30 ➨	GAAATACCTTCTATAGAAGAGAAGAAGCGCCTACGGTGCCTCATTGTCTTGCATCACGACACATG
31 ➨	GAAATACCTTCTATAGAAGAGAAGAAGCGCCTACGGTGCCTCATTGTCTTGCATCACGACACATG
32 ➨	GAAATACCTTCTATAGAAGAGAAGAAGCGCCTACGGTGCCTCATTGTCTTGCATCACGACACATG
33 ➨	GAAATACCTTCTATAGAAGAGAAGAAGCGCCTACGGTGCCTCATTGTCTTGCATCACGACACATG
34 ➨	GAAATACCTTCTATAGAAGAGAAGAAGCGCCTACGGTGCCTCATTGTCTTGCATCACGACACATG
35 ➨	GAAATACCTTCTATAGAAGAGAAGAAGCGCCTACGGTGCCTCATTGTCTTGCATCACGACACATG
36 ➨	GAAATACCTTCTATAGAAGAGAAGAAGCGCCTACGGTGCCTCATTGTCTTGCATCACGACACATG
37 ➨	GAAATACCTTCTATAGAAGAGAAGAAGCGCCTACGGTGCCTCATTGTCTTGCATCACGACACATG
38 ➨	GAAATACCTTCTATAGAAGAGAAGAAGCGCCTACGGTGCCTCATTGTCTTGCATCACGACACATG
39 ➨	GAAATACCTTCTATAGAAGAGAAGAAGCGCCTACGGTGCCTCATTGTCTTGCATCACGACACATG
40 ➨	GAAATACCTTCTATAGAAGAGAAGAAGCGCCTACGGTGCCTCATTGTCTTGCATCACGACACATG
41 ➨	GAAATACCTTCTATAGAAGAGAAGAAGCGCCTACGGTGCCTCATTGTCTTGCATCACGACACATG
42 ➨	GAAATACCTTCTATAGAAGAGAAGAAGCGCCTACGGTGCCTCATTGTCTTGCATCACGACACATG

	1630 · 1640 · 1650 · 1660 · 1670 · 1680 · 1690
	CAGATCGGTGAAGAGGGACATCTTCAGAAAGTTGGTGCATCTTCGCATCTTAGTTCTACGTGGAG
1	CAGATCGGTGAAGAGGGACATCTTCAGAAAGCTGGTGCATCTTCGCATCTTAGTTCTACGTGGAG
2	CAGATCGGTGAAGAGGGACATCTTCAGAAAGCTGGTGCATCTTCGCATCTTAGTTCTACGTGGAG
3	CAGATCGGTGAAGAGGGACATCTTCAGAAAGCTGGTGCATCTTCGCATCTTAGTTCTACGTGGAG
4	CAGATTGGTGAAGAGGGACATCTTCAGAAAGCTGGTGCATCTTCGCATCTTAGTTCTACGTGGAG
5	CAGATTGGTGAAGAGGGACATCTTCAGAAAGCTGGTGCATCTTCGCATCTTAGTTCTACGTGGAG
6	CAGATCGGTGAAGAGGGACATCTTCAGAAAGCTGGTGCATCTTCGCATCTTAGTTCTACGTGGAG
7	CAGATCGGTGAAGAGGGACATCTTCAGAAAGTTGGTGCATCTTCGCATCTTAGTTCTACGTGGAG
8	CAGATCGGTGAAGAGGGACATCTTCAGAAAGTTGGTGCATCTTCGCATCTTAGTTCTACGTGGAG
9	CAGATCGGTGAAGAGGGACATCTTCAGAAAGTTGGTGCATCTTCGCATCTTAGTTCTACGTGGAG
10	CAGATCGGTGAAGAGGGACATCTTCAGAAAGTTGGTGCATCTTCGCATCTTAGTTCTACGTGGAG
11	CAGATCGGTGAAGAGGGACATCTTCAGAAAGTTGGTGCATCTTCGCATCTTAGTTCTACGTGGAG
12	CAGATCGGTGAAGAGGGACATCTTCAGAAAGTTGGTGCATCTTCGCATCTTAGTTCTACGTGGAG
13	CAGATCGGTGAAGAGGGACATCTTCAGAAAGTTGGTGCATCTTCGCATCTTAGTTCTACGTGGAG
14	CAGATCGGTGAAGAGGGACATCTTCAGAAAGTTGGTGCATCTTCGCATCTTAGTTCTACGTGGAG
15	CAGATCGGTGAAGAGGGACATCTTCAGAAAGTTGGTGCATCTTCGCATCTTAGTTCTACGTGGAG
16	CAGATCGGTGAAGAGGGACATCTTCAGAAAGTTGGTGCATCTTCGCATCTTAGTTCTACGTGGAG
17	CAGATCGGTGAAGAGGGACATCTTCAGAAAGTTGGTGCATCTTCGCATCTTAGTTCTACGTGGAG
18	CAGATCGGTGAAGAGGGACATCTTCAGAAAGTTGGTGCATCTTCGCATCTTAGTTCTACGTGGAG
19	CAGATCGGTGAAGAGGGACATCTTCAGAAAGTTGGTGCATCTTCGCATCTTAGTTCTACGTGGAG
20	CAGATCGGTGAAGAGGGACATCTTCAGAAAGTTGGTGCATCTTCGCATCTTAGTTCTACGTGGAG
21	CAGATCGGTGAAGAGGGACATCTTCAGAAAGTTGGTGCATCTTCGCATCTTAGTTCTACGTGGAG
22	CAGATCGGTGAAGAGGGACATCTTCAGAAAGTTGGTGCATCTTCGCATCTTAGTTCTACGTGGAG
23	CAGATCGGTGAAGAGGGACATCTTCAGAAAGTTGGTGCATCTTCGCATCTTAGTTCTACGTGGAG
24	CAGATCGGTGAAGAGGGACATCTTCAGAAAGTTGGTGCATCTTCGCATCTTAGTTCTACGTGGAG
25	CAGATCGGTGAAGAGGGACATCTTCAGAAAGTTGGTGCATCTTCGCATCTTAGTTCTACGTGGAG
26	CAGATCGGTGAAGAGGGACATCTTCAGAAAGTTGGTGCATCTTCGCATCTTAGTTCTACGTGGAG
27	CAGATCGGTGAAGAGGGACATCTTCAGAAAGTTGGTGCATCTTCGCATCTTAGTTCTACGTGGAG
28	CAGATCGGTGAAGAGGGACATCTTCAGAAAGTTGGTGCATCTTCGCATCTTAGTTCTACGTGGAG
29	CAGATCGGTGAAGAGGGACATCTTCAGAAAGTTGGTGCATCTTCGCATCTTAGTTCTACGTGGAG
30	CAGATCGGTGAAGAGGGACATCTTCAGAAAGTTGGTGCATCTTCGCATCTTAGTTCTACGTGGAG
31	CAGATCGGTGAAGAGGGACATCTTCAGAAAGTTGGTGCATCTTCGCATCTTAGTTCTACGTGGAG
32	CAGATCGGTGAAGAGGGACATCTTCAGAAAGTTGGTGCATCTTCGCATCTTAGTTCTACGTGGAG
33	CAGATCGGTGAAGAGGGACATCTTCAGAAAGTTGGTGCATCTTCGCATCTTAGTTCTACGTGGAG
34	CAGATCGGTGAAGAGGGACATCTTCAGAAAGTTGGTGCATCTTCGCATCTTAGTTCTACGTGGAG
35	CAGATCGGTGAAGAGGGACATCTTCAGAAAGTTGGTGCATCTTCGCATCTTAGTTCTACGTGGAG
36	CAGATCGGTGAAGAGGGACATCTTCAGAAAGTTGGTGCATCTTCGCATCTTAGTTCTACGTGGAG
37	CAGATCGGTGAAGAGGGACATCTTCAGAAAGTTGGTGCATCTTCGCATCTTAGTTCTACGTGGAG
38	CAGATCGGTGAAGAGGGACATCTTCAGAAAGTTGGTGCATCTTCGCATCTTAGTTCTACGTGGAG
39	CAGATCGGTGAAGAGGGACATCTTCAGAAAGTTGGTGCATCTTCGCATCTTAGTTCTACGTGGAG
40	CAGATCGGTGAAGAGGGACATCTTCAGAAAGTTGGTGCATCTTCGCATCTTAGTTCTACGTGGAG
41	CAGATCGGTGAAGAGGGACATCTTCAGAAAGTTGGTGCATCTTCGCATCTTAGTTCTACGTGGAG
42	CAGATCGGTGAAGAGGGACATCTTCAGAAAGTTGGTGCATCTTCGCATCTTAGTTCTACGTGGAG

	1700 1710 1720 1730 1740 1750
	CAGGCCTTGAGAGCATACCTGCATCGGTGGGTTACTTGGCGCTGCTGAGGCTGCTGGATCTCAGC
1 ➨	CAGGCCTCGAGAGCATACCTGCATCGGTGGGTTACTTGGCGCTGCTGAGGCTGCTGGATCTCAGC
2 ➨	CAGGCCTCGAGAGCATACCTGCATCGGTGGGTTACTTGGCGCTGCTGAGGCTGCTGGATCTCAGC
3 ➨	CAGGCCTCGAGAGCATACCTGCATCGGTGGGTTACTTGGCGCTGCTGAGGCTGCTGGATCTCAGC
4 ➨	CAGGCCTCGAGAGCATACCTGCATCGGTGGGTTACTTGGCGCTGCTGTGGCTGCTGGATCTCAGC
5 ➨	CAGGCCTCGAGAGCATACCTGCATCGGTGGGTTACTTGGCGCTGCTGTGGCTGCTGGATCTCAGC
6 ➨	CAGGCCTCGAGAGCATACCTGCATCGGTGGGTTACTTGGCGCTGCTGAGGCTGCTGGATCTCAGC
7 ➨	CAGGCCTTGAGAGCATACCTGCATCGGTGGGTTACTTGGCGCTGCTGAGGCTGCTGGATCTCAGC
8 ➨	CAGGCCTTGAGAGCATACCTGCATCGGTGGGTTACTTGGCGCTGCTGAGGCTGCTGGATCTCAGC
9 ➨	CAGGCCTTGAGAGCATACCTGCATCGGTGGGTTACTTGGCGCTGCTGAGGCTGCTGGATCTCAGC
10 ➨	CAGGCCTTGAGAGCATACCTGCATCGGTGGGTTACTTGGCGCTGCTGAGGCTGCTGGATCTCAGC
11 ➨	CAGGCCTTGAGAGCATACCTGCATCGGTGGGTTACTTGGCGCTGCTGAGGCTGCTGGATCTCAGC
12 ➨	CAGGCCTTGAGAGCATACCTGCATCGGTGGGTTACTTGGCGCTGCTGAGGCTGCTGGATCTCAGC
13 ➨	CAGGCCTTGAGAGCATACCTGCATCGGTGGGTTACTTGGCGCTGCTGAGGCTGCTGGATCTCAGC
14 ➨	CAGGCCTTGAGAGCATACCTGCATCGGTGGGTTACTTGGCGCTGCTGAGGCTGCTGGATCTCAGC
15 ➨	CAGGCCTTGAGAGCATACCTGCATCGGTGGGTTACTTGGCGCTGCTGAGGCTGCTGGATCTCAGC
16 ➨	CAGGCCTTGAGAGCATACCTGCATCGGTGGGTTACTTGGCGCTGCTGAGGCTGCTGGATCTCAGC
17 ➨	CAGGCCTTGAGAGCATACCTGCATCGGTGGGTTACTTGGCGCTGCTGAGGCTGCTGGATCTCAGC
18 ➨	CAGGCCTTGAGAGCATACCTGCATCGGTGGGTTACTTGGCGCTGCTGAGGCTGCTGGATCTCAGC
19 ➨	CAGGCCTTGAGAGCATACCTGCATCGGTGGGTTACTTGGCGCTGCTGAGGCTGCTGGATCTCAGC
20 ➨	CAGGCCTTGAGAGCATACCTGCATCGGTGGGTTACTTGGCGCTGCTGAGGCTGCTGGATCTCAGC
21 ➨	CAGGCCTTGAGAGCATACCTGCATCGGTGGGTTACTTGGCGCTGCTGAGGCTGCTGGATCTCAGC
22 ➨	CAGGCCTTGAGAGCATACCTGCATCGGTGGGTTACTTGGCGCTGCTGAGGCTGCTGGATCTCAGC
23 ➨	CAGGCCTTGAGAGCATACCTGCATCGGTGGGTTACTTGGCGCTGCTGAGGCTGCTGGATCTCAGC
24 ➨	CAGGCCTTGAGAGCATACCTGCATCGGTGGGTTACTTGGCGCTGCTGAGGCTGCTGGATCTCAGC
25 ➨	CAGGCCTTGAGAGCATACCTGCATCGGTGGGTTACTTGGCGCTGCTGAGGCTGCTGGATCTCAGC
26 ➨	CAGGCCTTGAGAGCATACCTGCATCGGTGGGTTACTTGGCGCTGCTGAGGCTGCTGGATCTCAGC
27 ➨	CAGGCCTTGAGAGCATACCTGCATCGGTGGGTTACTTGGCGCTGCTGAGGCTGCTGGATCTCAGC
28 ➨	CAGGCCTTGAGAGCATACCTGCATCGGTGGGTTACTTGGCGCTGCTGAGGCTGCTGGATCTCAGC
29 ➨	CAGGCCTTGAGAGCATACCTGCATCGGTGGGTTACTTGGCGCTGCTGAGGCTGCTGGATCTCAGC
30 ➨	CAGGCCTTGAGAGCATACCTGCATCGGTGGGTTACTTGGCGCTGCTGAGGCTGCTGGATCTCAGC
31 ➨	CAGGCCTTGAGAGCATACCTGCATCGGTGGGTTACTTGGCGCTGCTGAGGCTGCTGGATCTCAGC
32 ➨	CAGGCCTTGAGAGCATACCTGCATCGGTGGGTTACTTGGCGCTGCTGAGGCTGCTGGATCTCAGC
33 ➨	CAGGCCTTGAGAGCATACCTGCATCGGTGGGTTACTTGGCGCTGCTGAGGCTGCTGGATCTCAGC
34 ➨	CAGGCCTTGAGAGCATACCTGCATCGGTGGGTTACTTGGCGCTGCTGAGGCTGCTGGATCTCAGC
35 ➨	CAGGCCTTGAGAGCATACCTGCATCGGTGGGTTACTTGGCGCTGCTGAGGCTGCTGGATCTCAGC
36 ➨	CAGGCCTTGAGAGCATACCTGCATCGGTGGGTTACTTGGCGCTGCTGAGGCTGCTGGATCTCAGC
37 ➨	CAGGCCTTGAGAGCATACCTGCATCGGTGGGTTACTTGGCGCTGCTGAGGCTGCTGGATCTCAGC
38 ➨	CAGGCCTTGAGAGCATACCTGCATCGGTGGGTTACTTGGCGCTGCTGAGGCTGCTGGATCTCAGC
39 ➨	CAGGCCTTGAGAGCATACCTGCATCGGTGGGTTACTTGGCGCTGCTGAGGCTGCTGGATCTCAGC
40 ➨	CAGGCCTTGAGAGCATACCTGCATCGGTGGGTTACTTGGCGCTGCTGAGGCTGCTGGATCTCAGC
41 ➨	CAGGCCTTGAGAGCATACCTGCATCGGTGGGTTACTTGGCGCTGCTGAGGCTGCTGGATCTCAGC
42 ➨	CAGGCCTTGAGAGCATACCTGCATCGGTGGGTTACTTGGCGCTGCTGAGGCTGCTGGATCTCAGC

1760 1770 1780 1790 1800 1810 1820

	TACAATGAGATCAAGGAGCTTCCAGGGTCCATCGGAAACCTTACCAGCCTTGGTTGCCTTTCAGT
1	TACAACGAGATCAAGGAGCTTCCGGGGTCCATCGGAAACCTTACCAGCCTTGGTTGCCTTTCAGT
2	TACAACGAGATCAAGGAGCTTCCGGGGTCCATCGGAAACCTTACCAGCCTTGGTTGCCTTTCAGT
3	TACAACGAGATCAAGGAGCTTCCGGGGTCCATCGGAAACCTTACCAGCCTTGGTTGCCTTTCAGT
4	TACAACGAGATCAAGGAGCTTCCGGGGTCCATCGGAAACCTTACCAGCCTTGGTTGCCTTTCAGT
5	TACAACGAGATCAAGGAGCTTCCGGGGTCCATCGGAAACCTTACCAGCCTTGGTTGCCTTTCAGT
6	TACAACGAGATCAAGGAGCTTCCGGGGTCCATCGGAAACCTTACCAGCCTTGGTTGCCTTTCAGT
7	TACAATGAGATCAAGGAGCTTCCAGGGTCCATCGGAAACCTTACCAGCCTTGGTTGCCTTTCAGT
8	TACAATGAGATCAAGGAGCTTCCAGGGTCCATCGGAAACCTTACCAGCCTTGGTTGCCTTTCAGT
9	TACAATGAGATCAAGGAGCTTCCAGGGTCCATCGGAAACCTTACCAGCCTTGGTTGCCTTTCAGT
10	TACAATGAGATCAAGGAGCTTCCAGGGTCCATCGGAAACCTTACCAGCCTTGGTTGCCTTTCAGT
11	TACAATGAGATCAAGGAGCTTCCAGGGTCCATCGGAAACCTTACCAGCCTTGGTTGCCTTTCAGT
12	TACAATGAGATCAAGGAGCTTCCAGGGTCCATCGGAAACCTTACCAGCCTTGGTTGCCTTTCAGT
13	TACAATGAGATCAAGGAGCTTCCAGGGTCCATCGGAAACCTTACCAGCCTTGGTTGCCTTTCAGT
14	TACAATGAGATCAAGGAGCTTCCAGGGTCCATCGGAAACCTTACCAGCCTTGGTTGCCTTTCAGT
15	TACAATGAGATCAAGGAGCTTCCAGGGTCCATCGGAAACCTTACCAGCCTTGGTTGCCTTTCAGT
16	TACAATGAGATCAAGGAGCTTCCAGGGTCCATCGGAAACCTTACCAGCCTTGGTTGCCTTTCAGT
17	TACAATGAGATCAAGGAGCTTCCAGGGTCCATCGGAAACCTTACCAGCCTTGGTTGCCTTTCAGT
18	TACAATGAGATCAAGGAGCTTCCAGGGTCCATCGGAAACCTTACCAGCCTTGGTTGCCTTTCAGT
19	TACAATGAGATCAAGGAGCTTCCAGGGTCCATCGGAAACCTTACCAGCCTTGGTTGCCTTTCAGT
20	TACAATGAGATCAAGGAGCTTCCAGGGTCCATCGGAAACCTTACCAGCCTTGGTTGCCTTTCAGT
21	TACAATGAGATCAAGGAGCTTCCAGGGTCCATCGGAAACCTTACCAGCCTTGGTTGCCTTTCAGT
22	TACAATGAGATCAAGGAGCTTCCAGGGTCCATCGGAAACCTTACCAGCCTTGGTTGCCTTTCAGT
23	TACAATGAGATCAAGGAGCTTCCAGGGTCCATCGGAAACCTTACCAGCCTTGGTTGCCTTTCAGT
24	TACAATGAGATCAAGGAGCTTCCAGGGTCCATCGGAAACCTTACCAGCCTTGGTTGCCTTTCAGT
25	TACAATGAGATCAAGGAGCTTCCAGGGTCCATCGGAAACCTTACCAGCCTTGGTTGCCTTTCAGT
26	TACAATGAGATCAAGGAGCTTCCAGGGTCCATCGGAAACCTTACCAGCCTTGGTTGCCTTTCAGT
27	TACAATGAGATCAAGGAGCTTCCAGGGTCCATCGGAAACCTTACCAGCCTTGGTTGCCTTTCAGT
28	TACAATGAGATCAAGGAGCTTCCAGGGTCCATCGGAAACCTTACCAGCCTTGGTTGCCTTTCAGT
29	TACAATGAGATCAAGGAGCTTCCAGGGTCCATCGGAAACCTTACCAGCCTTGGTTGCCTTTCAGT
30	TACAATGAGATCAAGGAGCTTCCAGGGTCCATCGGAAACCTTACCAGCCTTGGTTGCCTTTCAGT
31	TACAATGAGATCAAGGAGCTTCCAGGGTCCATCGGAAACCTTACCAGCCTTGGTTGCCTTTCAGT
32	TACAATGAGATCAAGGAGCTTCCAGGGTCCATCGGAAACCTTACCAGCCTTGGTTGCCTTTCAGT
33	TACAATGAGATCAAGGAGCTTCCAGGGTCCATCGGAAACCTTACCAGCCTTGGTTGCCTTTCAGT
34	TACAATGAGATCAAGGAGCTTCCAGGGTCCATCGGAAACCTTACCAGCCTTGGTTGCCTTTCAGT
35	TACAATGAGATCAAGGAGCTTCCAGGGTCCATCGGAAACCTTACCAGCCTTGGTTGCCTTTCAGT
36	TACAATGAGATCAAGGAGCTTCCAGGGTCCATCGGAAACCTTACCAGCCTTGGTTGCCTTTCAGT
37	TACAATGAGATCAAGGAGCTTCCAGGGTCCATCGGAAACCTTACCAGCCTTGGTTGCCTTTCAGT
38	TACAATGAGATCAAGGAGCTTCCAGGGTCCATCGGAAACCTTACCAGCCTTGGTTGCCTTTCAGT
39	TACAATGAGATCAAGGAGCTTCCAGGGTCCATCGGAAACCTTACCAGCCTTGGTTGCCTTTCAGT
40	TACAATGAGATCAAGGAGCTTCCAGGGTCCATCGGAAACCTTACCAGCCTTGGTTGCCTTTCAGT
41	TACAATGAGATCAAGGAGCTTCCAGGGTCCATCGGAAACCTTACCAGCCTTGGTTGCCTTTCAGT
42	TACAATGAGATCAAGGAGCTTCCAGGGTCCATCGGAAACCTTACCAGCCTTGGTTGCCTTTCAGT

	1830 1840 1850 1860 1870 1880
	GTTTGGTTGCACAAAGTTGGCAGCTTTGCCAACAAGTCTGATGAGGCTGACCACAATAAGCTTCC
1 ➡	GTTTGGTTGCACAAAGTTGGCAGCTTTGCCACAAAGTCTGATGAGGCTGACCACAATAAGCTTCC
2 ➡	GTTTGGTTGCACAAAGTTGGCAGCTTTGCCACAAAGTCTGATGAGGCTGACCACAATAAGCTTCC
3 ➡	GTTTGGTTGCACAAAGTTGGCAGCTTTGCCACAAAGTCTGATGAGGCTGACCACAATAAGCTTCC
4 ➡	GTTTGGTTGCACAAAGTTGGCAGCTTTGCCGCAGAGTCTGATGAGGCTGACCACAATAAGCTTCC
5 ➡	GTTTGGTTGCACAAAGTTGGCAGCTTTGCCGCAGAGTCTGATGAGGCTGACCACAATAAGCTTCC
6 ➡	GTTTGGTTGCACAAAGTTGGCAGCTTTGCCACAAAGTCTGATGAGGCTGACCACAATAAGCTTCC
7 ➡	GTTTGGTTGCACAAAGTTGGCAGCTTTGCCGACAAGTCTGATGAGGCTGACCACAATAAGCTTCC
8 ➡	GTTTGGTTGCACAAAGTTGGCAGCTTTGCCGACAAGTCTGATGAGGCTGACCACAATAAGCTTCC
9 ➡	GTTTGGTTGCACAAAGTTGGCAGCTTTGCCGACAAGTCTGATGAGGCTGACCACAATAAGCTTCC
10 ➡	GTTTGGTTGCACAAAGTTGGCAGCTTTGCCGACAAGTCTGATGAGGCTGACCACAATAAGCTTCC
11 ➡	GTTTGGTTGCACAAAGTTGGCAGCTTTGCCAACAAGTCTGATGAGGCTGACCACAATAAGCTTCC
12 ➡	GTTTGGTTGCACAAAGTTGGCAGCTTTGCCAACAAGTCTGATGAGGCTGACCACAATAAGCTTCC
13 ➡	GTTTGGTTGCACAAAGTTGGCAGCTTTGCCAACAAGTCTGATGAGGCTGACCACAATAAGCTTCC
14 ➡	GTTTGGTTGCACAAAGTTGGCAGCTTTGCCAACAAGTCTGATGAGGCTGACCACAATAAGCTTCC
15 ➡	GTTTGGTTGCACAAAGTTGGCAGCTTTGCCAACAAGTCTGATGAGGCTGACCACAATAAGCTTCC
16 ➡	GTTTGGTTGCACAAAGTTGGCAGCTTTGCCAACAAGTCTGATGAGGCTGACCACAATAAGCTTCC
17 ➡	GTTTGGTTGCACAAAGTTGGCAGCTTTGCCAACAAGTCTGATGAGGCTGACCACAATAAGCTTCC
18 ➡	GTTTGGTTGCACAAAGTTGGCAGCTTTGCCAACAAGTCTGATGAGGCTGACCACAATAAGCTTCC
19 ➡	GTTTGGTTGCACAAAGTTGGCAGCTTTGCCAACAAGTCTGATGAGGCTGACCACAATAAGCTTCC
20 ➡	GTTTGGTTGCACAAAGTTGGCAGCTTTGCCAACAAGTCTGATGAGGCTGACCACAATAAGCTTCC
21 ➡	GTTTGGTTGCACAAAGTTGGCAGCTTTGCCAACAAGTCTGATGAGGCTGACCACAATAAGCTTCC
22 ➡	GTTTGGTTGCACAAAGTTGGCAGCTTTGCCAACAAGTCTGATGAGGCTGACCACAATAAGCTTCC
23 ➡	GTTTGGTTGCACAAAGTTGGCAGCTTTGCCAACAAGTCTGATGAGGCTGACCACAATAAGCTTCC
24 ➡	GTTTGGTTGCACAAAGTTGGCAGCTTTGCCAACAAGTCTGATGAGGCTGACCACAATAAGCTTCC
25 ➡	GTTTGGTTGCACAAAGTTGGCAGCTTTGCCAACAAGTCTGATGAGGCTGACCACAATAAGCTTCC
26 ➡	GTTTGGTTGCACAAAGTTGGCAGCTTTGCCAACAAGTCTGATGAGGCTGACCACAATAAGCTTCC
27 ➡	GTTTGGTTGCACAAAGTTGGCAGCTTTGCCAACAAGTCTGATGAGGCTGACCACAATAAGCTTCC
28 ➡	GTTTGGTTGCACAAAGTTGGCAGCTTTGCCAACAAGTCTGATGAGGCTGACCACAATAAGCTTCC
29 ➡	GTTTGGTTGCACAAAGTTGGCAGCTTTGCCAACAAGTCTGATGAGGCTGACCACAATAAGCTTCC
30 ➡	GTTTGGTTGCACAAAGTTGGCAGCTTTGCCAACAAGTCTGATGAGGCTGACCACAATAAGCTTCC
31 ➡	GTTTGGTTGCACAAAGTTGGCAGCTTTGCCAACAAGTCTGATGAGGCTGACCACAATAAGCTTCC
32 ➡	GTTTGGTTGCACAAAGTTGGCAGCTTTGCCAACAAGTCTGATGAGGCTGACCACAATAAGCTTCC
33 ➡	GTTTGGTTGCACAAAGTTGGCAGCTTTGCCAACAAGTCTGATGAGGCTGACCACAATAAGCTTCC
34 ➡	GTTTGGTTGCACAAAGTTGGCAGCTTTGCCAACAAGTCTGATGAGGCTGACCACAATAAGCTTCC
35 ➡	GTTTGGTTGCACAAAGTTGGCAGCTTTGCCAACAAGTCTGATGAGGCTGACCACAATAAGCTTCC
36 ➡	GTTTGGTTGCACAAAGTTGGCAGCTTTGCCAACAAGTCTGATGAGGCTGACCACAATAAGCTTCC
37 ➡	GTTTGGTTGCACAAAGTTGGCAGCTTTGCCAACAAGTCTGATGAGGCTGACCACAATAAGCTTCC
38 ➡	GTTTGGTTGCACAAAGTTGGCAGCTTTGCCAACAAGTCTGATGAGGCTGACCACAATAAGCTTCC
39 ➡	GTTTGGTTGCACAAAGTTGGCAGCTTTGCCAACAAGTCTGATGAGGCTGACCACAATAAGCTTCC
40 ➡	GTTTGGTTGCACAAAGTTGGCAGCTTTGCCAACAAGTCTGATGAGGCTGACCACAATAAGCTTCC
41 ➡	GTTTGGTTGCACAAAGTTGGCAGCTTTGCCAACAAGTCTGATGAGGCTGACCACAATAAGCTTCC
42 ➡	GTTTGGTTGCACAAAGTTGGCAGCTTTGCCAACAAGTCTGATGAGGCTGACCACAATAAGCTTCC

```
      1890      1900      1910      1920      1930      1940      1950
    TCAAAATAGGAAACACAGGACTGGCGCAGGTTCCGAAAGGTATTGAGAATTTCAAGCAGATGGAT
 1  TCCAGATAGGAAACACAGGACTGGCGCAGGTTCCGAAAGGTATTGAGAATTTCAGGCAGATAGAT
 2  TCCAGATAGGAAACACAGGACTGGCGCAGGTTCCGAAAGGTATTGAGAATTTCAGGCAGATAGAT
 3  TCCAGATAGGAAACACAGGACTGGCGCAGGTTCCGAAAGGTATTGAGAATTTCAGGCAGATAGAT
 4  TCCAGATAGGAAACACAGGACTCGCACAGGTTCCGAAAGGTATTGAGAATTTTAGGCAGATAGAT
 5  TCCAGATAGGAAACACAGGACTCGCACAGGTTCCGAAAGGTATTGAGAATTTTAGGCAGATAGAT
 6  TCCAGATAGGAAACACAGGACTGGCGCAGGTTCCGAAAGGTATTGAGAATTTCAGGCAGATAGAT
 7  TCAAAATAGGAAACACAGGACTGGCGCAGGTTCCGAAAGGTATTGAGAATTTCAAGCAGATGGAT
 8  TCAAAATAGGAAACACAGGACTGGCGCAGGTTCCGAAAGGTATTGAGAATTTCAAGCAGATGGAT
 9  TCAAAATAGGAAACACAGGACTGGCGCAGGTTCCGAAAGGTATTGAGAATTTCAAGCAGATGGAT
10  TCAAAATAGGAAACACAGGACTGGCGCAGGTTCCGAAAGGTATTGAGAATTTCAAGCAGATGGAT
11  TCAAAATAGGAAACACAGGACTGGCGCAGGTTCCGAAAGGTATTGAGAATTTCAAGCAGATGGAT
12  TCAAAATAGGAAACACAGGACTGGCGCAGGTTCCGAAAGGTATTGAGAATTTCAAGCAGATGGAT
13  TCAAAATAGGAAACACAGGACTGGCGCAGGTTCCGAAAGGTATTGAGAATTTCAAGCAGATGGAT
14  TCAAAATAGGAAACACAGGACTGGCGCAGGTTCCGAAAGGTATTGAGAATTTCAAGCAGATGGAT
15  TCAAAATAGGAAACACAGGACTGGCGCAGGTTCCGAAAGGTATTGAGAATTTCAAGCAGATGGAT
16  TCAAAATAGGAAACACAGGACTGGCGCAGGTTCCGAAAGGTATTGAGAATTTCAAGCAGATGGAT
17  TCAAAATAGGAAACACAGGACTGGCGCAGGTTCCGAAAGGTATTGAGAATTTCAAGCAGATGGAT
18  TCAAAATAGGAAACACAGGACTGGCGCAGGTTCCGAAAGGTATTGAGAATTTCAAGCAGATGGAT
19  TCAAAATAGGAAACACAGGACTGGCGCAGGTTCCGAAAGGTATTGAGAATTTCAAGCAGATGGAT
20  TCAAAATAGGAAACACAGGACTGGCGCAGGTTCCGAAAGGTATTGAGAATTTCAAGCAGATGGAT
21  TCAAAATAGGAAACACAGGACTGGCGCAGGTTCCGAAAGGTATTGAGAATTTCAAGCAGATGGAT
22  TCAAAATAGGAAACACAGGACTGGCGCAGGTTCCGAAAGGTATTGAGAATTTCAAGCAGATGGAT
23  TCAAAATAGGAAACACAGGACTGGCGCAGGTTCCGAAAGGTATTGAGAATTTCAAGCAGATGGAT
24  TCAAAATAGGAAACACAGGACTGGCGCAGGTTCCGAAAGGTATTGAGAATTTCAAGCAGATGGAT
25  TCAAAATAGGAAACACAGGACTGGCGCAGGTTCCGAAAGGTATTGAGAATTTCAAGCAGATGGAT
26  TCAAAATAGGAAACACAGGACTGGCGCAGGTTCCGAAAGGTATTGAGAATTTCAAGCAGATGGAT
27  TCAAAATAGGAAACACAGGACTGGCGCAGGTTCCGAAAGGTATTGAGAATTTCAAGCAGATGGAT
28  TCAAAATAGGAAACACAGGACTGGCGCAGGTTCCGAAAGGTATTGAGAATTTCAAGCAGATGGAT
29  TCAAAATAGGAAACACAGGACTGGCGCAGGTTCCGAAAGGTATTGAGAATTTCAAGCAGATGGAT
30  TCAAAATAGGAAACACAGGACTGGCGCAGGTTCCGAAAGGTATTGAGAATTTCAAGCAGATGGAT
31  TCAAAATAGGAAACACAGGACTGGCGCAGGTTCCGAAAGGTATTGAGAATTTCAAGCAGATGGAT
32  TCAAAATAGGAAACACAGGACTGGCGCAGGTTCCGAAAGGTATTGAGAATTTCAAGCAGATGGAT
33  TCAAAATAGGAAACACAGGACTGGCGCAGGTTCCGAAAGGTATTGAGAATTTCAAGCAGATGGAT
34  TCAAAATAGGAAACACAGGACTGGCGCAGGTTCCGAAAGGTATTGAGAATTTCAAGCAGATGGAT
35  TCAAAATAGGAAACACAGGACTGGCGCAGGTTCCGAAAGGTATTGAGAATTTCAAGCAGATGGAT
36  TCAAAATAGGAAACACAGGACTGGCGCAGGTTCCGAAAGGTATTGAGAATTTCAAGCAGATGGAT
37  TCAAAATAGGAAACACAGGACTGGCGCAGGTTCCGAAAGGTATTGAGAATTTCAAGCAGATGGAT
38  TCAAAATAGGAAACACAGGACTGGCGCAGGTTCCGAAAGGTATTGAGAATTTCAAGCAGATGGAT
39  TCAAAATAGGAAACACAGGACTGGCGCAGGTTCCGAAAGGTATTGAGAATTTCAAGCAGATGGAT
40  TCAAAATAGGAAACACAGGACTGGCGCAGGTTCCGAAAGGTATTGAGAATTTCAAGCAGATGGAT
41  TCAAAATAGGAAACACAGGACTGGCGCAGGTTCCGAAAGGTATTGAGAATTTCAAGCAGATGGAT
42  TCAAAATAGGAAACACAGGACTGGCGCAGGTTCCGAAAGGTATTGAGAATTTCAAGCAGATGGAT
```

	1960 1970 1980 1990 2000 2010
	AACCTTAGATCAGTTTTCCAAAACGGTACTGATGGTTTCAGATTAGATGAACTGAGGGCACTCTC
1	AACCTTAGATCAGTTTTCCAAAACGGTACTGATGGTTTCAGATTAGATGAACTGAGGGCACTCTC
2	AACCTTAGATCAGTTTTCCAAAACGGTACTGATGGTTTCAGATTAGATGAACTGAGGGCACTCTC
3	AACCTTAGATCAGTTTTCCAAAACGGTACTGATGGTTTCAGATTAGATGAACTGAGGGCACTCTC
4	AACCTTAGATCAGTTTTCCAAAACAGTACTGATGGTTTCAGATTAGATGAACTGAGGGCACTCTC
5	AACCTTAGATCAGTTTTCCAAAACAGTACTGATGGTTTCAGATTAGATGAACTGAGGGCACTCTC
6	AACCTTAGATCAGTTTTCCAAAACGGTACTGATGGTTTCAGATTAGATGAACTGAGGGCACTCTC
7	AACCTTAGATCAGTTTTCCAAAACGGTACTGATGGTTTCAGATTAGATGAACTGAGGGCACTCTC
8	AACCTTAGATCAGTTTTCCAAAACGGTACTGATGGTTTCAGATTAGATGAACTGAGGGCACTCTC
9	AACCTTAGATCAGTTTTCCAAAACAGTACTGATGGTTTCAGATTAGATGAACTGAGGGCACTCTC
10	AACCTTAGATCAGTTTTCCAAAACAGTACTGATGGTTTCAGATTAGATGAACTGAGGGCACTCTC
11	AACCTTAGATCAGTTTTCCAAAACGGTACTGATGGTTTCAGATTAGATGAACTGAGGGCACTCTC
12	AACCTTAGATCAGTTTTCCAAAACGGTACTGATGGTTTCAGATTAGATGAACTGAGGGCACTCTC
13	AACCTTAGATCAGTTTTCCAAAACGGTACTGATGGTTTCAGATTAGATGAACTGAGGGCACTCTC
14	AACCTTAGATCAGTTTTCCAAAACGGTACTGATGGTTTCAGATTAGATGAACTGAGGGCACTCTC
15	AACCTTAGATCAGTTTTCCAAAACGGTACTGATGGTTTCAGATTAGATGAACTGAGGGCACTCTC
16	AACCTTAGATCAGTTTTCCAAAACGGTACTGATGGTTTCAGATTAGATGAACTGAGGGCACTCTC
17	AACCTTAGATCAGTTTTCCAAAACGGTACTGATGGTTTCAGATTAGATGAACTGAGGGCACTCTC
18	AACCTTAGATCAGTTTTCCAAAACGGTACTGATGGTTTCAGATTAGATGAACTGAGGGCACTCTC
19	AACCTTAGATCAGTTTTCCAAAACGGTACTGATGGTTTCAGATTAGATGAACTGAGGGCACTCTC
20	AACCTTAGATCAGTTTTCCAAAACGGTACTGATGGTTTCAGATTAGATGAACTGAGGGCACTCTC
21	AACCTTAGATCAGTTTTCCAAAACGGTACTGATGGTTTCAGATTAGATGAACTGAGGGCACTCTC
22	AACCTTAGATCAGTTTTCCAAAACGGTACTGATGGTTTCAGATTAGATGAACTGAGGGCACTCTC
23	AACCTTAGATCAGTTTTCCAAAACGGTACTGATGGTTTCAGATTAGATGAACTGAGGGCACTCTC
24	AACCTTAGATCAGTTTTCCAAAACGGTACTGATGGTTTCAGATTAGATGAACTGAGGGCACTCTC
25	AACCTTAGATCAGTTTTCCAAAACGGTACTGATGGTTTCAGATTAGATGAACTGAGGGCACTCTC
26	AACCTTAGATCAGTTTTCCAAAACGGTACTGATGGTTTCAGATTAGATGAACTGAGGGCACTCTC
27	AACCTTAGATCAGTTTTCCAAAACGGTACTGATGGTTTCAGATTAGATGAACTGAGGGCACTCTC
28	AACCTTAGATCAGTTTTCCAAAACGGTACTGATGGTTTCAGATTAGATGAACTGAGGGCACTCTC
29	AACCTTAGATCAGTTTTCCAAAACGGTACTGATGGTTTCAGATTAGATGAACTGAGGGCACTCTC
30	AACCTTAGATCAGTTTTCCAAAACGGTACTGATGGTTTCAGATTAGATGAACTGAGGGCACTCTC
31	AACCTTAGATCAGTTTTCCAAAACGGTACTGATGGTTTCAGATTAGATGAACTGAGGGCACTCTC
32	AACCTTAGATCAGTTTTCCAAAACGGTACTGATGGTTTCAGATTAGATGAACTGAGGGCACTCTC
33	AACCTTAGATCAGTTTTCCAAAACGGTACTGATGGTTTCAGATTAGATGAACTGAGGGCACTCTC
34	AACCTTAGATCAGTTTTCCAAAACGGTACTGATGGTTTCAGATTAGATGAACTGAGGGCACTCTC
35	AACCTTAGATCAGTTTTCCAAAACGGTACTGATGGTTTCAGATTAGATGAACTGAGGGCACTCTC
36	AACCTTAGATCAGTTTTCCAAAACGGTACTGATGGTTTCAGATTAGATGAACTGAGGGCACTCTC
37	AACCTTAGATCAGTTTTCCAAAACGGTACTGATGGTTTCAGATTAGATGAACTGAGGGCACTCTC
38	AACCTTAGATCAGTTTTCCAAAACGGTACTGATGGTTTCAGATTAGATGAACTGAGGGCACTCTC
39	AACCTTAGATCAGTTTTCCAAAACGGTACTGATGGTTTCAGATTAGATGAACTGAGGGCACTCTC
40	AACCTTAGATCAGTTTTCCAAAACGGTACTGATGGTTTCAGATTAGATGAACTGAGGGCACTCTC
41	AACCTTAGATCAGTTTTCCAAAACGGTACTGATGGTTTCAGATTAGATGAACTGAGGGCACTCTC
42	AACCTTAGATCAGTTTTCCAAAACGGTACTGATGGTTTCAGATTAGATGAACTGAGGGCACTCTC

```
      2020      2030      2040      2050      2060      2070      2080
    TATGATACGACGCCTCTGGGTTATCCGGCTGGAGACAGCGATACCGCCAACTGAGCCCATACTGT
 1  CATGATACGACGCCTCTGGGTTATCCGGCTGGAGACAGCGACACCGCCAACTGAGCCCGTATTGT
 2  CATGATACGACGCCTCTGGGTTATCCGGCTGGAGACAGCGACACCGCCAACTGAGCCCGTATTGT
 3  CATGATACGACGCCTCTGGGTTATCCGGCTGGAGACAGCGACACCGCCAACTGAGCCCGTATTGT
 4  CATGATACGACGCCTCTGGTTTATCTGGCTGGAGACAGCGACACCGCCAACTGAGTCCGTATTGT
 5  CATGATACGACGCCTCTGGTTTATCTGGCTGGAGACAGCGACACCGCCAACTGAGTCCGTATTGT
 6  CATGATACGACGCCTCTGGGTTATCCGGCTGGAGACAGCGACACCGCCAACTGAGCCCGTATTGT
 7  TATGATACGACGCCTCTGGGTTATCCGGCTGGAGACAGCGATACCGCCAACTGAGCCCATACTGT
 8  TATGATACGACGCCTCTGGGTTATCCGGCTGGAGACAGCGATACCGCCAACTGAGCCCATACTGT
 9  TATGATACGACGCCTCTGGGTTATCCGGCTGGAGACAGCGATACCGCCAACTGAGCCCATACTGT
10  TATGATACGACGCCTCTGGGTTATCCGGCTGGAGACAGCGATACCGCCAACTGAGCCCATACTGT
11  TATGATACGACGCCTCTGGGTTATCCGGCTGGAGACAGCGATACCGCCAACTGAGCCCATACTGT
12  TATGATACGACGCCTCTGGGTTATCCGGCTGGAGACAGCGATACCGCCAACTGAGCCCATACTGT
13  TATGATACGACGCCTCTGGGTTATCCGGCTGGAGACAGCGATACCGCCAACTGAGCCCATACTGT
14  TATGATACGACGCCTCTGGGTTATCCGGCTGGAGACAGCGATACCGCCAACTGAGCCCATACTGT
15  TATGATACGACGCCTCTGGGTTATCCGGCTGGAGACAGCGATACCGCCAACTGAGCCCATACTGT
16  TATGATACGACGCCTCTGGGTTATCCGGCTGGAGACAGCGATACCGCCAACTGAGCCCATACTGT
17  TATGATACGACGCCTCTGGGTTATCCGGCTGGAGACAGCGATACCGCCAACTGAGCCCATACTGT
18  TATGATACGACGCCTCTGGGTTATCCGGCTGGAGACAGCGATACCGCCAACTGAGCCCATACTGT
19  TATGATACGACGCCTCTGGGTTATCCGGCTGGAGACAGCGATACCGCCAACTGAGCCCATACTGT
20  TATGATACGACGCCTCTGGGTTATCCGGCTGGAGACAGCGATACCGCCAACTGAGCCCATACTGT
21  TATGATACGACGCCTCTGGGTTATCCGGCTGGAGACAGCGATACCGCCAACTGAGCCCATACTGT
22  TATGATACGACGCCTCTGGGTTATCCGGCTGGAGACAGCGATACCGCCAACTGAGCCCATACTGT
23  TATGATACGACGCCTCTGGGTTATCCGGCTGGAGACAGCGATACCGCCAACTGAGCCCATACTGT
24  TATGATACGACGCCTCTGGGTTATCCGGCTGGAGACAGCGATACCGCCAACTGAGCCCATACTGT
25  TATGATACGACGCCTCTGGGTTATCCGGCTGGAGACAGCGATACCGCCAACTGAGCCCATACTGT
26  TATGATACGACGCCTCTGGGTTATCCGGCTGGAGACAGCGATACCGCCAACTGAGCCCATACTGT
27  TATGATACGACGCCTCTGGGTTATCCGGCTGGAGACAGCGATACCGCCAACTGAGCCCATACTGT
28  TATGATACGACGCCTCTGGGTTATCCGGCTGGAGACAGCGATACCGCCAACTGAGCCCATACTGT
29  TATGATACGACGCCTCTGGGTTATCCGGCTGGAGACAGCGATACCGCCAACTGAGCCCATACTGT
30  TATGATACGACGCCTCTGGGTTATCCGGCTGGAGACAGCGATACCGCCAACTGAGCCCATACTGT
31  TATGATACGACGCCTCTGGGTTATCCGGCTGGAGACAGCGATACCGCCAACTGAGCCCATACTGT
32  TATGATACGACGCCTCTGGGTTATCCGGCTGGAGACAGCGATACCGCCAACTGAGCCCATACTGT
33  TATGATACGACGCCTCTGGGTTATCCGGCTGGAGACAGCGATACCGCCAACTGAGCCCATACTGT
34  TATGATACGACGCCTCTGGGTTATCCGGCTGGAGACAGCGATACCGCCAACTGAGCCCATACTGT
35  TATGATACGACGCCTCTGGGTTATCCGGCTGGAGACAGCGATACCGCCAACTGAGCCCATACTGT
36  TATGATACGACGCCTCTGGGTTATCCGGCTGGAGACAGCGATACCGCCAACTGAGCCCATACTGT
37  TATGATACGACGCCTCTGGGTTATCCGGCTGGAGACAGCGATACCGCCAACTGAGCCCATACTGT
38  TATGATACGACGCCTCTGGGTTATCCGGCTGGAGACAGCGATACCGCCAACTGAGCCCATACTGT
39  TATGATACGACGCCTCTGGGTTATCCGGCTGGAGACAGCGATACCGCCAACTGAGCCCATACTGT
40  TATGATACGACGCCTCTGGGTTATCCGGCTGGAGACAGCGATACCGCCAACTGAGCCCATACTGT
41  TATGATACGACGCCTCTGGGTTATCCGGCTGGAGACAGCGATACCGCCAACTGAGCCCATACTGT
42  TATGATACGACGCCTCTGGGTTATCCGGCTGGAGACAGCGATACCGCCAACTGAGCCCATACTGT
```

	2090 2100 2110 2120 2130 2140
	GCGACAAGGGTTACCTGAAAGAGCTAGGCCTGCGCTGCACCATGGGTAAGGAAGCCAATTGTCGA
1 ➡	GCGACAAGGGTTACCTGAAAGAGCTAGGCCTGCGCTGTACCATGGGCAAGGAAGCCAATTGTCGA
2 ➡	GCGACAAGGGTTACCTGAAAGAGCTAGGCCTGCGCTGTACCATGGGCAAGGAAGCCAATTGTCGA
3 ➡	GCGACAAGGGTTACCTGAAAGAGCTAGGCCTGCGCTGTACCATGGGCAAGGAAGCCAATTGTCGA
4 ➡	GTGACAAGGGTTACCTGAAAGAGCTAGGCCTGCGCTGTACCATGGGCAAGGAAGCCAATTGTCGA
5 ➡	GTGACAAGGGTTACCTGAAAGAGCTAGGCCTGCGCTGTACCATGGGCAAGGAAGCCAATTGTCGA
6 ➡	GCGACAAGGGTTACCTGAAAGAGCTAGGCCTGCGCTGTACCATGGGCAAGGAAGCCAATTGTCGA
7 ➡	GCGACAAGGGTTACCTGAAAGAGCTAGGCCTGCGCTGCACCATGGGTAAGGAAGCCAATTGTCGA
8 ➡	GCGACAAGGGTTACCTGAAAGAGCTAGGCCTGCGCTGCACCATGGGTAAGGAAGCCAATTGTCGA
9 ➡	GCGACAAGGGTTACCTGAAAGAGCTAGGCCTGCGCTGCACCATGGGTAAGGAAGCCAATTGTCGA
10 ➡	GCGACAAGGGTTACCTGAAAGAGCTAGGCCTGCGCTGCACCATGGGTAAGGAAGCCAATTGTCGA
11 ➡	GCGACAAGGGTTACCTGAAAGAGCTAGGCCTGCGCTGCACCATGGGTAAGGAAGCCAATTGTCGA
12 ➡	GCGACAAGGGTTACCTGAAAGAGCTAGGCCTGCGCTGCACCATGGGTAAGGAAGCCAATTGTCGA
13 ➡	GCGACAAGGGTTACCTGAAAGAGCTAGGCCTGCGCTGCACCATGGGTAAGGAAGCCAATTGTCGA
14 ➡	GCGACAAGGGTTACCTGAAAGAGCTAGGCCTGCGCTGCACCATGGGTAAGGAAGCCAATTGTCGA
15 ➡	GCGACAAGGGTTACCTGAAAGAGCTAGGCCTGCGCTGCACCATGGGTAAGGAAGCCAATTGTCGA
16 ➡	GCGACAAGGGTTACCTGAAAGAGCTAGGCCTGCGCTGCACCATGGGTAAGGAAGCCAATTGTCGA
17 ➡	GCGACAAGGGTTACCTGAAAGAGCTAGGCCTGCGCTGCACCATGGGTAAGGAAGCCAATTGTCGA
18 ➡	GCGACAAGGGTTACCTGAAAGAGCTAGGCCTGCGCTGCACCATGGGTAAGGAAGCCAATTGTCGA
19 ➡	GCGACAAGGGTTACCTGAAAGAGCTAGGCCTGCGCTGCACCATGGGTAAGGAAGCCAATTGTCGA
20 ➡	GCGACAAGGGTTACCTGAAAGAGCTAGGCCTGCGCTGCACCATGGGTAAGGAAGCCAATTGTCGA
21 ➡	GCGACAAGGGTTACCTGAAAGAGCTAGGCCTGCGCTGCACCATGGGTAAGGAAGCCAATTGTCGA
22 ➡	GCGACAAGGGTTACCTGAAAGAGCTAGGCCTGCGCTGCACCATGGGTAAGGAAGCCAATTGTCGA
23 ➡	GCGACAAGGGTTACCTGAAAGAGCTAGGCCTGCGCTGCACCATGGGTAAGGAAGCCAATTGTCGA
24 ➡	GCGACAAGGGTTACCTGAAAGAGCTAGGCCTGCGCTGCACCATGGGTAAGGAAGCCAATTGTCGA
25 ➡	GCGACAAGGGTTACCTGAAAGAGCTAGGCCTGCGCTGCACCATGGGTAAGGAAGCCAATTGTCGA
26 ➡	GCGACAAGGGTTACCTGAAAGAGCTAGGCCTGCGCTGCACCATGGGTAAGGAAGCCAATTGTCGA
27 ➡	GCGACAAGGGTTACCTGAAAGAGCTAGGCCTGCGCTGCACCATGGGTAAGGAAGCCAATTGTCGA
28 ➡	GCGACAAGGGTTACCTGAAAGAGCTAGGCCTGCGCTGCACCATGGGTAAGGAAGCCAATTGTCGA
29 ➡	GCGACAAGGGTTACCTGAAAGAGCTAGGCCTGCGCTGCACCATGGGTAAGGAAGCCAATTGTCGA
30 ➡	GCGACAAGGGTTACCTGAAAGAGCTAGGCCTGCGCTGCACCATGGGTAAGGAAGCCAATTGTCGA
31 ➡	GCGACAAGGGTTACCTGAAAGAGCTAGGCCTGCGCTGCACCATGGGTAAGGAAGCCAATTGTCGA
32 ➡	GCGACAAGGGTTACCTGAAAGAGCTAGGCCTGCGCTGCACCATGGGTAAGGAAGCCAATTGTCGA
33 ➡	GCGACAAGGGTTACCTGAAAGAGCTAGGCCTGCGCTGCACCATGGGTAAGGAAGCCAATTGTCGA
34 ➡	GCGACAAGGGTTACCTGAAAGAGCTAGGCCTGCGCTGCACCATGGGTAAGGAAGCCAATTGTCGA
35 ➡	GCGACAAGGGTTACCTGAAAGAGCTAGGCCTGCGCTGCACCATGGGTAAGGAAGCCAATTGTCGA
36 ➡	GCGACAAGGGTTACCTGAAAGAGCTAGGCCTGCGCTGCACCATGGGTAAGGAAGCCAATTGTCGA
37 ➡	GCGACAAGGGTTACCTGAAAGAGCTAGGCCTGCGCTGCACCATGGGTAAGGAAGCCAATTGTCGA
38 ➡	GCGACAAGGGTTACCTGAAAGAGCTAGGCCTGCGCTGCACCATGGGTAAGGAAGCCAATTGTCGA
39 ➡	GCGACAAGGGTTACCTGAAAGAGCTAGGCCTGCGCTGCACCATGGGTAAGGAAGCCAATTGTCGA
40 ➡	GCGACAAGGGTTACCTGAAAGAGCTAGGCCTGCGCTGCACCATGGGTAAGGAAGCCAATTGTCGA
41 ➡	GCGACAAGGGTTACCTGAAAGAGCTAGGCCTGCGCTGCACCATGGGTAAGGAAGCCAATTGTCGA
42 ➡	GCGACAAGGGTTACCTGAAAGAGCTAGGCCTGCGCTGCACCATGGGTAAGGAAGCCAATTGTCGA

	2150 2160 2170 2180 2190 2200 2210
	ACTCACTATCCGGACAGCAAGGTGAAGAGGATTGAGGAGATCTACGAGAGTTTTGCCCACCGCC
1 ➨	ACTCACTACCCGGACAGTAAGGTGAAGAGGATTGAGGAGATCTACGAGAGTTTTGCCCACCGCC
2 ➨	ACTCACTACCCGGACAGTAAGGTGAAGAGGATTGAGGAGATCTACGAGAGTTTTGCCCACCGCC
3 ➨	ACTCACTACCCGGACAGTAAGGTGAAGAGGATTGAGGAGATCTACGAGAGTTTTGCCCACCGCC
4 ➨	ACTCACTACCCGGACAGTAAGGTGAAGAGGATTGAGGAGATCTACGAGAGTTTTGCCCACCGCC
5 ➨	ACTCACTACCCGGACAGTAAGGTGAAGAGGATTGAGGAGATCTACGAGAGTTTTGCCCACCGCC
6 ➨	ACTCACTACCCGGACAGTAAGGTGAAGAGGATTGAGGAGGTCTACGAGAGTTTTGCCCACCGCC
7 ➨	ACTCACTATCCGGACAGCAAGGTGAAGAGGATTGAGGAGATCTACGAGAGTTTTGCCCACCGCC
8 ➨	ACTCACTATCCAGACAGCAAGGTGAAGAGGATTGAGGAGATCTATGAGAGTTTTGCCCACCGCC
9 ➨	ACTCACTATCCGGACAGCAAGGTGAAGAGGATTGAGGAGATCTACGAGAGTTTTGCCCACCGCC
10 ➨	ACTCACTATCCGGACAGCAAGGTGAAGAGGATTGAGGAGATCTACGAGAGTTTTGCCCACCGCC
11 ➨	ACTCACCATCCGGACAGCAAGGTGAGGAGGATTGAGGAGATCTACGAGAGTTTTGCCCACCGCC
12 ➨	ACTCACCATCCGGACAGCAAGGTGAGGAGGATTGAGGAGATCTACGAGAGTTTTGCCCACCGCC
13 ➨	ACTCACCATCCGGACAGCAAGGTGAGGAGGATTGAGGAGATCTACGAGAGTTTTGCCCACCGCC
14 ➨	ACTCACTATCCGGACAGCAAGGTGAAGAGGATTGAGGAGATCTACGAGAGTTTTGCCCACCGCC
15 ➨	ACTCACTATCCGGACAGCAAGGTGAAGAGGATTGAGGAGATCTACGAGAGTTTTGCCCACCGCC
16 ➨	ACTCACTATCCGGACAGCAAGGTGAAGAGGATTGAGGAGATCTACGAGAGTTTTGCCCACCGCC
17 ➨	ACTCACTATCCGGACAGCAAGGTGAAGAGGATTGAGGAGATCTACGAGAGTTTTGCCCACCGCC
18 ➨	ACTCACTATCCGGACAGCAAGGTGAAGAGGATTGAGGAGATCTACGAGAGTTTTGCCCACCGCC
19 ➨	ACTCACTATCCGGACAGCAAGGTGAAGAGGATTGAGGAGATCTACGAGAGTTTTGCCCACCGCC
20 ➨	ACTCACTATCCGGACAGCAAGGTGAAGAGGATTGAGGAGATCTACGAGAGTTTTGCCCACCGCC
21 ➨	ACTCACTATCCGGACAGCAAGGTGAAGAGGATTGAGGAGATCTACGAGAGTTTTGCCCACCGCC
22 ➨	ACTCACTATCCGGACAGCAAGGTGAAGAGGATTGAGGAGATCTACGAGAGTTTTGCCCACCGCC
23 ➨	ACTCACTATCCGGACAGCAAGGTGAAGAGGATTGAGGAGATCTACGAGAGTTTTGCCCACCGCC
24 ➨	ACTCACTATCCGGACAGCAAGGTGAAGAGGATTGAGGAGATCTACGAGAGTTTTGCCCACCGCC
25 ➨	ACTCACTATCCGGACAGCAAGGTGAAGAGGATTGAGGAGATCTACGAGAGTTTTGCCCACCGCC
26 ➨	ACTCACTATCCGGACAGCAAGGTGAAGAGGATTGAGGAGATCTACGAGAGTTTTGCCCACCGCC
27 ➨	ACTCACTATCCGGACAGCAAGGTGAAGAGGATTGAGGAGATCTACGAGAGTTTTGCCCACCGCC
28 ➨	ACTCACTATCCGGACAGCAAGGTGAAGAGGATTGAGGAGATCTACGAGAGTTTTGCCCACCGCC
29 ➨	ACTCACTATCCGGACAGCAAGGTGAAGAGGATTGAGGAGATCTACGAGAGTTTTGCCCACCGCC
30 ➨	ACTCACTATCCGGACAGCAAGGTGAAGAGGATTGAGGAGATCTACGAGAGTTTTGCCCACCGCC
31 ➨	ACTCACTATCCGGACAGCAAGGTGAAGAGGATTGAGGAGATCTACGAGAGTTTTGCCCACCGCC
32 ➨	ACTCACTATCCGGACAGCAAGGTGAAGAGGATTGAGGAGATCTACGAGAGTTTTGCCCACCGCC
33 ➨	ACTCACTATCCGGACAGCAAGGTGAAGAGGATTGAGGAGATCTACGAGAGTTTTGCCCACCGCC
34 ➨	ACTCACTATCCGGACAGCAAGGTGAAGAGGATTGAGGAGATCTACGAGAGTTTTGCCCACCGCC
35 ➨	ACTCACTATCCGGACAGCAAGGTGAAGAGGATTGAGGAGATCTACGAGAGTTTTGCCCACCGCC
36 ➨	ACTCACTATCCGGACAGCAAGGTGAAGAGGATTGAGGAGATCTACGAGAGTTTTGCCCACCGCC
37 ➨	ACTCACTATCCGGACAGCAAGGTGAAGAGGATTGAGGAGATCTACGAGAGTTTTGCCCACCGCC
38 ➨	ACTCACTATCCGGACAGCAAGGTGAAGAGGATTGAGGAGATCTACGAGAGTTTTGCCCACCGCC
39 ➨	ACTCACTATCCGGACAGCAAGGTGAAGAGGATTGAGGAGATCTACGAGAGTTTTGCCCACCGCC
40 ➨	ACTCACTATCCGGACAGCAAGGTGAAGAGGATTGAGGAGATCTACGAGAGTTTTGCCCACCGCC
41 ➨	ACTCACTATCCGGACAGCAAGGTGAAGAGGATTGAGGAGATCTACGAGAGTTTTGCCCACCGCC
42 ➨	ACTCACTATCCGGACAGCAAGGTGAAGAGGATTGAGGAGATCTACGAGAGTTTTGCCCACCGCC

2220 2230 2240 2250 2260 2270

	AAGCCTAAGCTACGTCTTCATTGATGGGTTCCCTGGTTGCATGTTTCCAACTTGGCTATCTTCAG
1	AAGCCTAAGCTACGTCTTTATTGATGGGTTCCCTGGTCGCATGTTTCCAACTTGGCTATCTTTAG
2	AAGCCTAAGCTACGTCTTTATTGATGGGTTCCCTGGTCGCATGTTTCCAACTTGGCTATCTTTAG
3	AAGCCTAAGCTACGTCTTTATTGATGGGTTCCCTGGTCGCATGTTTCCAACTTGGCTATCTTTAG
4	AAGCCTAAGCTACGTCTTTATTGATGGGTTCCCTGGTCGCATGTTTCCAACTTGGCTATCTTTAG
5	AAGCCTAAGCTACGTCTTTATTGATGGGTTCCCTGGTCGCATGTTTCCAACTTGGCTATCTTTAG
6	AAGCCTAAGCTACGTCTTTATTGATGGGTTCCCTGGTCGCATGTTTCCAACTTGGCTATCTTTAG
7	AAGCCTAAGCTACGTCTTCATTGATGGGTTCCCTGGTTGCATGTTTCCAACTTGGCTATCTTCAG
8	AAGCCTAAGCTACGTCTTCATTGATGGGTTCCCTGGTTGCATGTTTCCAACTTGGCTATCTTCAG
9	AAGCCTAAGCTACGTCTTCATTGATGGGTTCCCTGGTTGCATGTTTCCAACTTGGCTATCTTCAG
10	AAGCCTAAGCTACGTCTTCATTGATGGGTTCCCTGGTTGCATGTTTCCAACTTGGCTATCTTCAG
11	AGGCCTAAGCTACGTCTTCATTGATGGGTTCCCTGGTTGCATGTTTCCAACTTGGCTATCTTCAG
12	AGGCCTAAGCTACGTCTTCATTGATGGGTTCCCTGGTTGCATGTTTCCAACTTGGCTATCTTCAG
13	AGGCCTAAGCTACGTCTTCATTGATGGGTTCCCTGGTTGCATGTTTCCAACTTGGCTATCTTCAG
14	AAGCCTAAGCTACGTCTTCATTGATGGGTTCCCTGGTTGCATGTTTCCAACTTGGCTATCTTCAG
15	AAGCCTAAGCTACGTCTTCATTGATGGGTTCCCTGGTTGCATGTTTCCAACTTGGCTATCTTCAG
16	AAGCCTAAGCTACGTCTTCATTGATGGGTTCCCTGGTTGCATGTTTCCAACTTGGCTATCTTCAG
17	AAGCCTAAGCTACGTCTTCATTGATGGGTTCCCTGGTTGCATGTTTCCAACTTGGCTATCTTCAG
18	AAGCCTAAGCTACGTCTTCATTGATGGGTTCCCTGGTTGCATGTTTCCAACTTGGCTATCTTCAG
19	AAGCCTAAGCTACGTCTTCATTGATGGGTTCCCTGGTTGCATGTTTCCAACTTGGCTATCTTCAG
20	AAGCCTAAGCTACGTCTTCATTGATGGGTTCCCTGGTTGCATGTTTCCAACTTGGCTATCTTCAG
21	AAGCCTAAGCTACGTCTTCATTGATGGGTTCCCTGGTTGCATGTTTCCAACTTGGCTATCTTCAG
22	AAGCCTAAGCTACGTCTTCATTGATGGGTTCCCTGGTTGCATGTTTCCAACTTGGCTATCTTCAG
23	AAGCCTAAGCTACGTCTTCATTGATGGGTTCCCTGGTTGCATGTTTCCAACTTGGCTATCTTCAG
24	AAGCCTAAGCTACGTCTTCATTGATGGGTTCCCTGGTTGCATGTTTCCAACTTGGCTATCTTCAG
25	AAGCCTAAGCTACGTCTTCATTGATGGGTTCCCTGGTTGCATGTTTCCAACTTGGCTATCTTCAG
26	AAGCCTAAGCTACGTCTTCATTGATGGGTTCCCTGGTTGCATGTTTCCAACTTGGCTATCTTCAG
27	AAGCCTAAGCTACGTCTTCATTGATGGGTTCCCTGGTTGCATGTTTCCAACTTGGCTATCTTCAG
28	AAGCCTAAGCTACGTCTTCATTGATGGGTTCCCTGGTTGCATGTTTCCAACTTGGCTATCTTCAG
29	AAGCCTAAGCTACGTCTTCATTGATGGGTTCCCTGGTTGCATGTTTCCAACTTGGCTATCTTCAG
30	AAGCCTAAGCTACGTCTTCATTGATGGGTTCCCTGGTTGCATGTTTCCAACTTGGCTATCTTCAG
31	AAGCCTAAGCTACGTCTTCATTGATGGGTTCCCTGGTTGCATGTTTCCAACTTGGCTATCTTCAG
32	AAGCCTAAGCTACGTCTTCATTGATGGGTTCCCTGGTTGCATGTTTCCAACTTGGCTATCTTCAG
33	AAGCCTAAGCTACGTCTTCATTGATGGGTTCCCTGGTTGCATGTTTCCAACTTGGCTATCTTCAG
34	AAGCCTAAGCTACGTCTTCATTGATGGGTTCCCTGGTTGCATGTTTCCAACTTGGCTATCTTCAG
35	AAGCCTAAGCTACGTCTTCATTGATGGGTTCCCTGGTTGCATGTTTCCAACTTGGCTATCTTCAG
36	AAGCCTAAGCTACGTCTTCATTGATGGGTTCCCTGGTTGCATGTTTCCAACTTGGCTATCTTCAG
37	AAGCCTAAGCTACGTCTTCATTGATGGGTTCCCTGGTTGCATGTTTCCAACTTGGCTATCTTCAG
38	AAGCCTAAGCTACGTCTTCATTGATGGGTTCCCTGGTTGCATGTTTCCAACTTGGCTATCTTCAG
39	AAGCCTAAGCTACGTCTTCATTGATGGGTTCCCTGGTTGCATGTTTCCAACTTGGCTATCTTCAG
40	AAGCCTAAGCTACGTCTTCATTGATGGGTTCCCTGGTTGCATGTTTCCAACTTGGCTATCTTCAG
41	AAGCCTAAGCTACGTCTTCATTGATGGGTTCCCTGGTTGCATGTTTCCAACTTGGCTATCTTCAG
42	AAGCCTAAGCTACGTCTTCATTGATGGGTTCCCTGGTTGCATGTTTCCAACTTGGCTATCTTCAG

2280 2290 2300 2310 2320 2330 2340

	AACCGCAGAATAAACTGCCAAACCTGGCTCATATGCACTTCAACGACTGCATATCCTGCCCGAAG
1	AACCGCAGAATAAACTTCCAAACCTGGCTCATATGCACTTCTATGACTGCATATCCTGCCCGAAG
2	AACCGCAGAATAAACTTCCAAACCTGGCTCATATGCACTTCTATGACTGCATATCCTGCCCGAAG
3	AACCGCAGAATAAACTTCCAAACCTGGCTCATATGCACTTCTATGACTGCATATCCTGCCCGAAG
4	AACCGCAGAATAAACTTCCAAACCTGGCTCATATGCACTTCTATGACTGCATATCCTGCCCGAAG
5	AACCGCAGAATAAACTTCCAAACCTGGCTCATATGCACTTCTATGACTGCATATCCTGCCCGAAG
6	AACCGCAGAATAAACTTCCAAACCTGGCTCATATGCACTTCTATGACTGCATATCCTGCCCGAAG
7	AACCGCAGAATAAACTGCCAAACCTGGCTCATATGCACTTCAACGACTGCATATCCTGCCCGAAG
8	AACCGCAGAATAAACTGCCAAACCTGGCTCATATGCACTTCAACGACTGCATATCCTGCCCGAAG
9	AACCGCAGAATAAACTGCCAAACCTGGCTCATATGCACTTCAACGACTGCATATCCTGCCCGAAG
10	AACCGCAGAATAAACTGCCAAACCTGGCTCATATGCACTTCAACGACTGCATATCCTGCCCGAAG
11	AACCGCAGAATAAACTGCCAAGCCTGGCTCATATGCACTTCAACGACTGCATATCCTGCCCGAAG
12	AACCGCAGAATAAACTGCCAAGCCTGGCTCATATGCACTTCAACGACTGCATATCCTGCCCGAAG
13	AACCGCAGAATAAACTGCCAAGCCTGGCTCATATGCACTTCAACGACTGCATATCCTGCCCGAAG
14	AACCGCAGAATAAACTGCCAAACCTGGCTCATATGCACTTCAACGACTGCATATCCTGCCCGAAG
15	AACCGCAGAATAAACTGCCAAACCTGGCTCATATGCACTTCAACGACTGCATATCCTGCCCGAAG
16	AACCGCAGAATAAACTGCCAAACCTGGCTCATATGCACTTCAACGACTGCATATCCTGCCCGAAG
17	AACCGCAGAATAAACTGCCAAACCTGGCTCATATGCACTTCAACGACTGCATATCCTGCCCGAAG
18	AACCGCAGAATAAACTGCCAAACCTGGCTCATATGCACTTCAACGACTGCATATCCTGCCCGAAG
19	AACCGCAGAATAAACTGCCAAACCTGGCTCATATGCACTTCAACGACTGCATATCCTGCCCGAAG
20	AACCGCAGAATAAACTGCCAAACCTGGCTCATATGCACTTCAACGACTGCATATCCTGCCCGAAG
21	AACCGCAGAATAAACTGCCAAACCTGGCTCATATGCACTTCAACGACTGCATATCCTGCCCGAAG
22	AACCGCAGAATAAACTGCCAAACCTGGCTCATATGCACTTCAACGACTGCATATCCTGCCCGAAG
23	AACCGCAGAATAAACTGCCAAACCTGGCTCATATGCACTTCAACGACTGCATATCCTGCCCGAAG
24	AACCGCAGAATAAACTGCCAAACCTGGCTCATATGCACTTCAACGACTGCATATCCTGCCCGAAG
25	AACCGCAGAATAAACTGCCAAACCTGGCTCATATGCACTTCAACGACTGCATATCCTGCCCGAAG
26	AACCGCAGAATAAACTGCCAAACCTGGCTCATATGCACTTCAACGACTGCATATCCTGCCCGAAG
27	AACCGCAGAATAAACTGCCAAACCTGGCTCATATGCACTTCAACGACTGCATATCCTGCCCGAAG
28	AACCGCAGAATAAACTGCCAAACCTGGCTCATATGCACTTCAACGACTGCATATCCTGCCCGAAG
29	AACCGCAGAATAAACTGCCAAACCTGGCTCATATGCACTTCAACGACTGCATATCCTGCCCGAAG
30	AACCGCAGAATAAACTGCCAAACCTGGCTCATATGCACTTCAACGACTGCATATCCTGCCCGAAG
31	AACCGCAGAATAAACTGCCAAACCTGGCTCATATGCACTTCAACGACTGCATATCCTGCCCGAAG
32	AACCGCAGAATAAACTGCCAAACCTGGCTCATATGCACTTCAACGACTGCATATCCTGCCCGAAG
33	AACCGCAGAATAAACTGCCAAACCTGGCTCATATGCACTTCAACGACTGCATATCCTGCCCGAAG
34	AACCGCAGAATAAACTGCCAAACCTGGCTCATATGCACTTCAACGACTGCATATCCTGCCCGAAG
35	AACCGCAGAATAAACTGCCAAACCTGGCTCATATGCACTTCAACGACTGCATATCCTGCCCGAAG
36	AACCGCAGAATAAACTGCCAAACCTGGCTCATATGCACTTCAACGACTGCATATCCTGCCCGAAG
37	AACCGCAGAATAAACTGCCAAACCTGGCTCATATGCACTTCAACGACTGCATATCCTGCCCGAAG
38	AACCGCAGAATAAACTGCCAAACCTGGCTCATATGCACTTCAACGACTGCATATCCTGCCCGAAG
39	AACCGCAGAATAAACTGCCAAACCTGGCTCATATGCACTTCAACGACTGCATATCCTGCCCGAAG
40	AACCGCAGAATAAACTGCCAAACCTGGCTCATATGCACTTCAACGACTGCATATCCTGCCCGAAG
41	AACCGCAGAATAAACTGCCAAACCTGGCTCATATGCACTTCAACGACTGCATATCCTGCCCGAAG
42	AACCGCAGAATAAACTGCCAAACCTGGCTCATATGCACTTCAACGACTGCATATCCTGCCCGAAG

2350 2360 2370 2380 2390 2400

	CTTCCCCCAGCAGGCCAGCTACCGTTTCTGCAGGTTCTTCACGTCAAAGGAGCTGACGCAGTAGT
1	CTTCCCCCAGCAGGCCAGCTACCGTTTCTGCAGGTTCTTCACGTCAAAGGAGCTGATGCAGTGGT
2	CTTCCCCCAGCAGGCCAGCTACCGTTTCTGCAGGTTCTTCACGTCAAAGGAGCTGATGCAGTGGT
3	CTTCCCCCAGCAGGCCAGCTACCGTTTCTGCAGGTTCTTCACGTCAAAGGAGCTGATGCAGTGGT
4	CTTCCCCCAGCAGGCCAGCTACCGTTTCTGCAGGTTCTTCACGTCAAAGGAGCTGATGCAGTGGT
5	CTTCCCCCAGCAGGCCAGCTACCGTTTCTGCAGGTTCTTCACGTCAAAGGAGCTGATGCAGTGGT
6	CTTCCCCCAGCAGGCCAGCTACCGTTTCTGCAGGTTCTTCACGTCAAAGGAGCTGATGCAGTGGT
7	CTTCCCCCAGCAGGCCAGCTACCGTTTCTGCAGGTTCTTCACGTCAAAGGAGCTGACGCAGTAGT
8	CTTCCCCCAGCAGGCCAGCTACCATTTCTGCAGGTTCTTCACGTCAAAGGAGCTGACGCAGTAGT
9	CTTCCCCCAGCAGGCCAGCTACCGTTTCTGCAGGTTCTTCACGTCAAAGGAGCTGACGCAGTAGT
10	CTTCCCCCAGCAGGCCAGCTACCGTTTCTGCAGGTTCTTCACGTCAAAGGAGCTGACGCAGTAGT
11	CTTCCCCCAGCAGGCCAGCTACCGTTTCTGCAGGTTCTTCACGTCAAAGGAGCTGACGCAGTAGT
12	CTTCCCCCAGCAGGCCAGCTACCGTTTCTGCAGGTTCTTCACGTCAAAGGAGCTGACGCAGTAGT
13	CTTCCCCCAGCAGGCCAGCTACCGTTTCTGCAGGTTCTTCACGTCAAAGGAGCTGACGCAGTAGT
14	CTTCCCCCAGCAGGCCAGCTACCGTTTCTGCAGGTTCTTCACGTCAAAGGAGCTGACGCAGTAGT
15	CTTCCCCCAGCAGGCCAGCTACCGTTTCTGCAGGTTCTTCACGTCAAAGGAGCTGACGCAGTAGT
16	CTTCCCCCAGCAGGCCAGCTACCGTTTCTGCAGGTTCTTCACGTCAAAGGAGCTGACGCAGTAGT
17	CTTCCCCCAGCAGGCCAGCTACCGTTTCTGCAGGTTCTTCACGTCAAAGGAGCTGACGCAGTAGT
18	CTTCCCCCAGCAGGCCAGCTACCGTTTCTGCAGGTTCTTCACGTCAAAGGAGCTGACGCAGTAGT
19	CTTCCCCCAGCAGGCCAGCTACCGTTTCTGCAGGTTCTTCACGTCAAAGGAGCTGACGCAGTAGT
20	CTTCCCCCAGCAGGCCAGCTACCGTTTCTGCAGGTTCTTCACGTCAAAGGAGCTGACGCAGTAGT
21	CTTCCCCCAGCAGGCCAGCTACCGTTTCTGCAGGTTCTTCACGTCAAAGGAGCTGACGCAGTAGT
22	CTTCCCCCAGCAGGCCAGCTACCGTTTCTGCAGGTTCTTCACGTCAAAGGAGCTGACGCAGTAGT
23	CTTCCCCCAGCAGGCCAGCTACCGTTTCTGCAGGTTCTTCACGTCAAAGGAGCTGACGCAGTAGT
24	CTTCCCCCAGCAGGCCAGCTACCGTTTCTGCAGGTTCTTCACGTCAAAGGAGCTGACGCAGTAGT
25	CTTCCCCCAGCAGGCCAGCTACCGTTTCTGCAGGTTCTTCACGTCAAAGGAGCTGACGCAGTAGT
26	CTTCCCCCAGCAGGCCAGCTACCGTTTCTGCAGGTTCTTCACGTCAAAGGAGCTGACGCAGTAGT
27	CTTCCCCCAGCAGGCCAGCTACCGTTTCTGCAGGTTCTTCACGTCAAAGGAGCTGACGCAGTAGT
28	CTTCCCCCAGCAGGCCAGCTACCGTTTCTGCAGGTTCTTCACGTCAAAGGAGCTGACGCAGTAGT
29	CTTCCCCCAGCAGGCCAGCTACCGTTTCTGCAGGTTCTTCACGTCAAAGGAGCTGACGCAGTAGT
30	CTTCCCCCAGCAGGCCAGCTACCGTTTCTGCAGGTTCTTCACGTCAAAGGAGCTGACGCAGTAGT
31	CTTCCCCCAGCAGGCCAGCTACCGTTTCTGCAGGTTCTTCACGTCAAAGGAGCTGACGCAGTAGT
32	CTTCCCCCAGCAGGCCAGCTACCGTTTCTGCAGGTTCTTCACGTCAAAGGAGCTGACGCAGTAGT
33	CTTCCCCCAGCAGGCCAGCTACCGTTTCTGCAGGTTCTTCACGTCAAAGGAGCTGACGCAGTAGT
34	CTTCCCCCAGCAGGCCAGCTACCGTTTCTGCAGGTTCTTCACGTCAAAGGAGCTGACGCAGTAGT
35	CTTCCCCCAGCAGGCCAGCTACCGTTTCTGCAGGTTCTTCACGTCAAAGGAGCTGACGCAGTAGT
36	CTTCCCCCAGCAGGCCAGCTACCGTTTCTGCAGGTTCTTCACGTCAAAGGAGCTGACGCAGTAGT
37	CTTCCCCCAGCAGGCCAGCTACCGTTTCTGCAGGTTCTTCACGTCAAAGGAGCTGACGCAGTAGT
38	CTTCCCCCAGCAGGCCAGCTACCGTTTCTGCAGGTTCTTCACGTCAAAGGAGCTGACGCAGTAGT
39	CTTCCCCCAGCAGGCCAGCTACCGTTTCTGCAGGTTCTTCACGTCAAAGGAGCTGACGCAGTAGT
40	CTTCCCCCAGCAGGCCAGCTACCGTTTCTGCAGGTTCTTCACGTCAAAGGAGCTGACGCAGTAGT
41	CTTCCCCCAGCAGGCCAGCTACCGTTTCTGCAGGTTCTTCACGTCAAAGGAGCTGACGCAGTAGT
42	CTTCCCCCAGCAGGCCAGCTACCGTTTCTGCAGGTTCTTCACGTCAAAGGAGCTGACGCAGTAGT

2410 2420 2430 2440 2450 2460 2470

	GAACATAGGTGCTGAGCTCCTCGGAAACAGCATCCCATCCGGAACACATACCACTGCTTTTCCAA
1	GAACATAGGTGCTGAGCTCCTCGGAAACGGCATCCCATCTGGAACACATACCACTGCTTTTCCAA
2	GAACATAGGTGCTGAGCTCCTCGGAAACGGCATCCCATCTGGAACACATACCACTGCTTTTCCAA
3	GAACATAGGTGCTGAGCTCCTCGGAAACGGCATCCCATCTGGAACACATACCACTGCTTTTCCAA
4	GAACATAGGTGCTGAGCTCCTCGGAAACGGCATCCCATCTGGAACACATACCACTGCTTTTCCAA
5	GAACATAGGTGCTGAGCTCCTCGGAAACGGCATCCCATCTGGAACACATACCACTGCTTTTCCAA
6	GAACATAGGTGCTGAGCTCCTCGGAAACGGCATCCCATCTGGAACACATACCACTGCTTTTCCAA
7	GAGGATAGGTGCTGAGCTCCTCGGAAACAGCATCCCATCCGGAACACATACCACTGCTTTTCCAA
8	GAACATAGGTGCTGAGCTCCTCGGAAACAGCATCCCATCCGGAACACATACCACTGCTTTTCCAA
9	GAACATAGGTGCTGAGCTCCTCGGAAACAGCATCCCATCCGGAACACATACCACTGCTTTTCCAA
10	GAACATAGGTGCTGAGCTCCTCGGAAACAGCATCCCATCCGGAACACATACCACTGCTTTTCCAA
11	GAACATAGGTGCTGAGCTCCTCGGAAACAGCATCCCATCCGGAACACATACCACTGCTTTTCCAA
12	GAACATAGGTGCTGAGCTCCTCGGAAACAGCATCCCATCCGGAACACATACCACTGCTTTTCCAA
13	GAACATAGGTGCTGAGCTCCTCGGAAACAGCATCCCATCCGGAACACATACCACTGCTTTTCCAA
14	GAACATAGGTGCTGAGCTCCTCGGAAACAGCATCCCATCCGGAACACATACCACTGCTTTTCCAA
15	GAACATAGGTGCTGAGCTCCTCGGAAACAGCATCCCATCCGGAACACATACCACTGCTTTTCCAA
16	GAACATAGGTGCTGAGCTCCTCGGAAACAGCATCCCATCCGGAACACATACCACTGCTTTTCCAA
17	GAACATAGGTGCTGAGCTCCTCGGAAACAGCATCCCATCCGGAACACATACCACTGCTTTTCCAA
18	GAACATAGGTGCTGAGCTCCTCGGAAACAGCATCCCATCCGGAACACATACCACTGCTTTTCCAA
19	GAACATAGGTGCTGAGCTCCTCGGAAACAGCATCCCATCCGGAACACATACCACTGCTTTTCCAA
20	GAACATAGGTGCTGAGCTCCTCGGAAACAGCATCCCATCCGGAACACATACCACTGCTTTTCCAA
21	GAACATAGGTGCTGAGCTCCTCGGAAACAGCATCCCATCCGGAACACATACCACTGCTTTTCCAA
22	GAACATAGGTGCTGAGCTCCTCGGAAACAGCATCCCATCCGGAACACATACCACTGCTTTTCCAA
23	GAACATAGGTGCTGAGCTCCTCGGAAACAGCATCCCATCCGGAACACATACCACTGCTTTTCCAA
24	GAACATAGGTGCTGAGCTCCTCGGAAACAGCATCCCATCCGGAACACATACCACTGCTTTTCCAA
25	GAACATAGGTGCTGAGCTCCTCGGAAACAGCATCCCATCCGGAACACATACCACTGCTTTTCCAA
26	GAACATAGGTGCTGAGCTCCTCGGAAACAGCATCCCATCCGGAACACATACCACTGCTTTTCCAA
27	GAACATAGGTGCTGAGCTCCTCGGAAACAGCATCCCATCCGGAACACATACCACTGCTTTTCCAA
28	GAACATAGGTGCTGAGCTCCTCGGAAACAGCATCCCATCCGGAACACATACCACTGCTTTTCCAA
29	GAACATAGGTGCTGAGCTCCTCGGAAACAGCATCCCATCCGGAACACATACCACTGCTTTTCCAA
30	GAACATAGGTGCTGAGCTCCTCGGAAACAGCATCCCATCCGGAACACATACCACTGCTTTTCCAA
31	GAACATAGGTGCTGAGCTCCTCGGAAACAGCATCCCATCCGGAACACATACCACTGCTTTTCCAA
32	GAACATAGGTGCTGAGCTCCTCGGAAACAGCATCCCATCCGGAACACATACCACTGCTTTTCCAA
33	GAACATAGGTGCTGAGCTCCTCGGAAACAGCATCCCATCCGGAACACATACCACTGCTTTTCCAA
34	GAACATAGGTGCTGAGCTCCTCGGAAACAGCATCCCATCCGGAACACATACCACTGCTTTTCCAA
35	GAACATAGGTGCTGAGCTCCTCGGAAACAGCATCCCATCCGGAACACATACCACTGCTTTTCCAA
36	GAACATAGGTGCTGAGCTCCTCGGAAACAGCATCCCATCCGGAACACATACCACTGCTTTTCCAA
37	GAACATAGGTGCTGAGCTCCTCGGAAACAGCATCCCATCCGGAACACATACCACTGCTTTTCCAA
38	GAACATAGGTGCTGAGCTCCTCGGAAACAGCATCCCATCCGGAACACATACCACTGCTTTTCCAA
39	GAACATAGGTGCTGAGCTCCTCGGAAACAGCATCCCATCCGGAACACATACCACTGCTTTTCCAA
40	GAACATAGGTGCTGAGCTCCTCGGAAACAGCATCCCATCCGGAACACATACCACTGCTTTTCCAA
41	GAACATAGGTGCTGAGCTCCTCGGAAACAGCATCCCATCCGGAACACATACCACTGCTTTTCCAA
42	GAACATAGGTGCTGAGCTCCTCGGAAACAGCATCCCATCCGGAACACATACCACTGCTTTTCCAA

2480 2490 2500 2510 2520 2530

	AGCTCGAGCTGCTTGAGATCCTCGACATGTACAACTGGCAGAATTGGTCACTAAGCATGGATACC
1	AGCTCGAGCTGCTTGAGATCCTCGACATGTACAACTGGCAGAATTGGTCACTAAGCATGGATACC
2	AGCTCGAGCTGCTTGAGATCCTCGACATGTACAACTGGCAGAATTGGTCACTAAGCATGGATACC
3	AGCTCGAGCTGCTTGAGATCCTCGACATGTACAACTGGCAGAATTGGTCACTAAGCATGGATACC
4	AGCTCGAGCTGCTTGAGATCCTCGACATGTACAACTGGCAGAATTGGTCACTAAGCATGGATACT
5	AGCTCGAGCTGCTTGAGATCCTCGACATGTACAACTGGCAGAATTGGTCACTAAGCATGGATACT
6	AGCTCGAGCTGCTTGAGATCCTCGACATGTACAACTGGCAGAATTGGTCACTAAGCATGGATACC
7	AGCTCGAGCTGCTTGAGATCCTCGACATGTACAACTGGCAGAATTGGTCACTAAGCATGGATACC
8	AGCTCGAGCTGCTTGAGATCCTCGACATGTACAACTGGCAGAATTGGTCACTAAGCATGGATACC
9	AGCTCGAGCTGCTTGAGATCCTCGACATGTACAACTGGCAGAATTGGTCACTAAGCATGGATACC
10	AGCTCGAGCTGCTTGAGATCCTCGACATGTACAACTGGCAGAATTGGTCACTAAGCATGGATACC
11	AGCTCGAGCTGCTTGAGATCCTCGACATGTACAACTGGCAGAATTGGTCACTAAGCATGGATACC
12	AGCTCGAGCTGCTTGAGATCCTCGACATGTACAACTGGCAGAATTGGTCACTAAGCATGGATACC
13	AGCTCGAGCTGCTTGAGATCCTCGACATGTACAACTGGCAGAATTGGTCACTAAGCATGGATACC
14	AGCTCGAGCTGCTTGAGATCCTCGACATGTACAACTGGCAGAATTGGTCACTAAGCATGGATACC
15	AGCTCGAGCTGCTTGAGATCCTCGACATGTACAACTGGCAGAATTGGTCACTAAGCATGGATACC
16	AGCTCGAGCTGCTTGAGATCCTCGACATGTACAACTGGCAGAATTGGTCACTAAGCATGGATACC
17	AGCTCGAGCTGCTTGAGATCCTCGACATGTACAACTGGCAGAATTGGTCACTAAGCATGGATACC
18	AGCTCGAGCTGCTTGAGATCCTCGACATGTACAACTGGCAGAATTGGTCACTAAGCATGGATACC
19	AGCTCGAGCTGCTTGAGATCCTCGACATGTACAACTGGCAGAATTGGTCACTAAGCATGGATACC
20	AGCTCGAGCTGCTTGAGATCCTCGACATGTACAACTGGCAGAATTGGTCACTAAGCATGGATACC
21	AGCTCGAGCTGCTTGAGATCCTCGACATGTACAACTGGCAGAATTGGTCACTAAGCATGGATACC
22	AGCTCGAGCTGCTTGAGATCCTCGACATGTACAACTGGCAGAATTGGTCACTAAGCATGGATACC
23	AGCTCGAGCTGCTTGAGATCCTCGACATGTACAACTGGCAGAATTGGTCACTAAGCATGGATACC
24	AGCTCGAGCTGCTTGAGATCCTCGACATGTACAACTGGCAGAATTGGTCACTAAGCATGGATACC
25	AGCTCGAGCTGCTTGAGATCCTCGACATGTACAACTGGCAGAATTGGTCACTAAGCATGGATACC
26	AGCTCGAGCTGCTTGAGATCCTCGACATGTACAACTGGCAGAATTGGTCACTAAGCATGGATACC
27	AGCTCGAGCTGCTTGAGATCCTCGACATGTACAACTGGCAGAATTGGTCACTAAGCATGGATACC
28	AGCTCGAGCTGCTTGAGATCCTCGACATGTACAACTGGCAGAATTGGTCACTAAGCATGGATACC
29	AGCTCGAGCTGCTTGAGATCCTCGACATGTACAACTGGCAGAATTGGTCACTAAGCATGGATACC
30	AGCTCGAGCTGCTTGAGATCCTCGACATGTACAACTGGCAGAATTGGTCACTAAGCATGGATACC
31	AGCTCGAGCTGCTTGAGATCCTCGACATGTACAACTGGCAGAATTGGTCACTAAGCATGGATACC
32	AGCTCGAGCTGCTTGAGATCCTCGACATGTACAACTGGCAGAATTGGTCACTAAGCATGGATACC
33	AGCTCGAGCTGCTTGAGATCCTCGACATGTACAACTGGCAGAATTGGTCACTAAGCATGGATACC
34	AGCTCGAGCTGCTTGAGATCCTCGACATGTACAACTGGCAGAATTGGTCACTAAGCATGGATACC
35	AGCTCGAGCTGCTTGAGATCCTCGACATGTACAACTGGCAGAATTGGTCACTAAGCATGGATACC
36	AGCTCGAGCTGCTTGAGATCCTCGACATGTACAACTGGCAGAATTGGTCACTAAGCATGGATACC
37	AGCTCGAGCTGCTTGAGATCCTCGACATGTACAACTGGCAGAATTGGTCACTAAGCATGGATACC
38	AGCTCGAGCTGCTTGAGATCCTCGACATGTACAACTGGCAGAATTGGTCACTAAGCATGGATACC
39	AGCTCGAGCTGCTTGAGATCCTCGACATGTACAACTGGCAGAATTGGTCACTAAGCATGGATACC
40	AGCTCGAGCTGCTTGAGATCCTCGACATGTACAACTGGCAGAATTGGTCACTAAGCATGGATACC
41	AGCTCGAGCTGCTTGAGATCCTCGACATGTACAACTGGCAGAATTGGTCACTAAGCATGGATACC
42	AGCTCGAGCTGCTTGAGATCCTCGACATGTACAACTGGCAGAATTGGTCACTAAGCATGGATACC

	2540 2550 2560 2570 2580 2590 2600
	TTGTTCGAGAAAACACAACAGCAATCCCTTATGCCATGCCTTACACGGCTGCGGCTGATAAATTG
1 ➨	TTGTTCGAGAACACACAACAGCAATTCCTTATGCCATGCCTTACACGCCTGCGGCTGATAAATTG
2 ➨	TTGTTCGAGAACACACAACAGCAATTCCTTATGCCATGCCTTACACGCCTGCGGCTGATAAATTG
3 ➨	TTGTTCGAGAACACACAACAGCAATTCCTTATGCCATGCCTTACACGCCTGCGGCTGATAAATTG
4 ➨	TTGTTCGACAACACACAACAGCAATTGTTTATGCCATGCCTTACACGCCTGCGGCTGATAAATTG
5 ➨	TTGTTCGACAACACACAACAGCAATTGTTTATGCCATGCCTTACACGCCTGCGGCTGATAAATTG
6 ➨	TTGTTCGAGAACACACAACAGCAGTTCCTTATGCCATGCCTTACACGCCTGCGGCTGATAAATTG
7 ➨	TTGTTCGAGAAAACACAACAGCAATCCCTTATGCCATGCCTTACACGGCTGCGGCTGATAAATTG
8 ➨	TTGTTCGAGAAAACACAACAGCAATCCCTTATGCCATGCCTTACACGGCTGCGGCTGATAAATTG
9 ➨	TTGTTCGAGAAAACACAACAGCAATCCCTTATGCCATGCCTTACACGGCTGCGGCTGATAAATTG
10 ➨	TTGTTCGAGAAAACACAACAGCAATCCCTTATGCCATGCCTTACACGGCTGCGGCTGATAAATTG
11 ➨	TTGTTCGAGAAAACACAACAGCAATCCCTTATGCCATGCCTTACACGGCTGCGGCTGATAAATTG
12 ➨	TTGTTCGAGAAAACACAACAGCAATCCCTTATGCCATGCCTTACACGGCTGCGGCTGATAAATTG
13 ➨	TTGTTCGAGAAAACACAACAGCAATCCCTTATGCCATGCCTTACACGGCTGCGGCTGATAAATTG
14 ➨	TTGTTCGAGAAAACACAACAGCAATCCCTTATGCCATGCCTTACACGGCTGCGGCTGATAAATTG
15 ➨	TTGTTCGAGAAAACACAACAGCAATCCCTTATGCCATGCCTTACACGGCTGCGGCTGATAAATTG
16 ➨	TTGTTCGAGAAAACACAACAGCAATCCCTTATGCCATGCCTTACACGGCTGCGGCTGATAAATTG
17 ➨	TTGTTCGAGAAAACACAACAGCAATCCCTTATGCCATGCCTTACACGGCTGCGGCTGATAAATTG
18 ➨	TTGTTCGAGAAAACACAACAGCAATCCCTTATGCCATGCCTTACACGGCTGCGGCTGATAAATTG
19 ➨	TTGTTCGAGAAAACACAACAGCAATCCCTTATGCCATGCCTTACACGGCTGCGGCTGATAAATTG
20 ➨	TTGTTCGAGAAAACACAACAGCAATCCCTTATGCCATGCCTTACACGGCTGCGGCTGATAAATTG
21 ➨	TTGTTCGAGAAAACACAACAGCAATCCCTTATGCCATGCCTTACACGGCTGCGGCTGATAAATTG
22 ➨	TTGTTCGAGAAAACACAACAGCAATCCCTTATGCCATGCCTTACACGGCTGCGGCTGATAAATTG
23 ➨	TTGTTCGAGAAAACACAACAGCAATCCCTTATGCCATGCCTTACACGGCTGCGGCTGATAAATTG
24 ➨	TTGTTCGAGAAAACACAACAGCAATCCCTTATGCCATGCCTTACACGGCTGCGGCTGATAAATTG
25 ➨	TTGTTCGAGAAAACACAACAGCAATCCCTTATGCCATGCCTTACACGGCTGCGGCTGATAAATTG
26 ➨	TTGTTCGAGAAAACACAACAGCAATCCCTTATGCCATGCCTTACACGGCTGCGGCTGATAAATTG
27 ➨	TTGTTCGAGAAAACACAACAGCAATCCCTTATGCCATGCCTTACACGGCTGCGGCTGATAAATTG
28 ➨	TTGTTCGAGAAAACACAACAGCAATCCCTTATGCCATGCCTTACACGGCTGCGGCTGATAAATTG
29 ➨	TTGTTCGAGAAAACACAACAGCAATCCCTTATGCCATGCCTTACACGGCTGCGGCTGATAAATTG
30 ➨	TTGTTCGAGAAAACACAACAGCAATCCCTTATGCCATGCCTTACACGGCTGCGGCTGATAAATTG
31 ➨	TTGTTCGAGAAAACACAACAGCAATCCCTTATGCCATGCCTTACACGGCTGCGGCTGATAAATTG
32 ➨	TTGTTCGAGAAAACACAACAGCAATCCCTTATGCCATGCCTTACACGGCTGCGGCTGATAAATTG
33 ➨	TTGTTCGAGAAAACACAACAGCAATCCCTTATGCCATGCCTTACACGGCTGCGGCTGATAAATTG
34 ➨	TTGTTCGAGAAAACACAACAGCAATCCCTTATGCCATGCCTTACACGGCTGCGGCTGATAAATTG
35 ➨	TTGTTCGAGAAAACACAACAGCAATCCCTTATGCCATGCCTTACACGGCTGCGGCTGATAAATTG
36 ➨	TTGTTCGAGAAAACACAACAGCAATCCCTTATGCCATGCCTTACACGGCTGCGGCTGATAAATTG
37 ➨	TTGTTCGAGAAAACACAACAGCAATCCCTTATGCCATGCCTTACACGGCTGCGGCTGATAAATTG
38 ➨	TTGTTCGAGAAAACACAACAGCAATCCCTTATGCCATGCCTTACACGGCTGCGGCTGATAAATTG
39 ➨	TTGTTCGAGAAAACACAACAGCAATCCCTTATGCCATGCCTTACACGGCTGCGGCTGATAAATTG
40 ➨	TTGTTCGAGAAAACACAACAGCAATCCCTTATGCCATGCCTTACACGGCTGCGGCTGATAAATTG
41 ➨	TTGTTCGAGAAAACACAACAGCAATCCCTTATGCCATGCCTTACACGGCTGCGGCTGATAAATTG
42 ➨	TTGTTCGAGAAAACACAACAGCAATCCCTTATGCCATGCCTTACACGGCTGCGGCTGATAAATTG

2610 2620 2630 2640 2650 2660

	CCCCAAGTTGAGAGCTCTCCCTGATCATCTTCACAGAGTTGTCAATCTACAAAGGATCCAAATAG
1 ➨	CCCCAAGTTGAGAGCTCTCCCTGATCATCTTCACAGAGTTGTCAATCTACAAAGGATCCAAATAG
2 ➨	CCCCAAGTTGAGAGCTCTCCCTGATCATCTTCACAGAGTTGTCAATCTACAAAGGATCCAAATAG
3 ➨	CCCCAAGTTGAGAGCTCTCCCTGATCATCTTCACAGAGTTGTCAATCTACAAAGGATCCAAATAG
4 ➨	CCCCAAGTTGAGAGCTCTCCCTGATCATCTTCATAGAGTTGTCAATCTACAGAGGATCCACATAG
5 ➨	CCCCAAGTTGAGAGCTCTCCCTGATCATCTTCATAGAGTTGTCAATCTACAGAGGATCCACATAG
6 ➨	CCCCAAGTTGAGAGCTCTCCCTGATCATCTTCACAGAGTTGTCAATCTACAAAGGATCCAAATAG
7 ➨	CCCCAAGTTGAGAGCTCTCCCTGATCATCTTCACAGAGTTGTCAATCTACAAAGGATCCAAATAG
8 ➨	CCCCAAGTTGAGAGCTCTCCCTGATCATCTTCACAGAGTTGTCAATCTACAAAGGATCCAAATAG
9 ➨	CCCCAAGTTGAGAGCTCTCCCTGATCATCTTCACAGAGTTGTCAATCTACAAAGGATCCAAATAG
10 ➨	CCCCAAGTTGAGAGCTCTCCCTGATCATCTTCACAGAGTTGTCAATCTACAAAGGATCCAAATAG
11 ➨	CCCCAAGTTGAGAGCTCTCCCTGATCATCTTCACAGAGTTGTCAATCTACAAAGGATCCAAATAG
12 ➨	CCCCAAGTTGAGAGCTCTCCCTGATCATCTTCACAGAGTTGTCAATCTACAAAGGATCCAAATAG
13 ➨	CCCCAAGTTGAGAGCTCTCCCTGATCATCTTCACAGAGTTGTCAATCTACAAAGGATCCAAATAG
14 ➨	CCCCAAGTTGAGAGCTCTCCCTGATCATCTTCACAGAGTTGTCAATCTACAAAGGATCCAAATAG
15 ➨	CCCCAAGTTGAGAGCTCTCCCTGATCATCTTCACAGAGTTGTCAATCTACAAAGGATCCAAATAG
16 ➨	CCCCAAGTTGAGAGCTCTCCCTGATCATCTTCACAGAGTTGTCAATCTACAAAGGATCCAAATAG
17 ➨	CCCCAAGTTGAGAGCTCTCCCTGATCATCTTCACAGAGTTGTCAATCTACAAAGGATCCAAATAG
18 ➨	CCCCAAGTTGAGAGCTCTCCCTGATCATCTTCACAGAGTTGTCAATCTACAAAGGATCCAAATAG
19 ➨	CCCCAAGTTGAGAGCTCTCCCTGATCATCTTCACAGAGTTGTCAATCTACAAAGGATCCAAATAG
20 ➨	CCCCAAGTTGAGAGCTCTCCCTGATCATCTTCACAGAGTTGTCAATCTACAAAGGATCCAAATAG
21 ➨	CCCCAAGTTGAGAGCTCTCCCTGATCATCTTCACAGAGTTGTCAATCTACAAAGGATCCAAATAG
22 ➨	CCCCAAGTTGAGAGCTCTCCCTGATCATCTTCACAGAGTTGTCAATCTACAAAGGATCCAAATAG
23 ➨	CCCCAAGTTGAGAGCTCTCCCTGATCATCTTCACAGAGTTGTCAATCTACAAAGGATCCAAATAG
24 ➨	CCCCAAGTTGAGAGCTCTCCCTGATCATCTTCACAGAGTTGTCAATCTACAAAGGATCCAAATAG
25 ➨	CCCCAAGTTGAGAGCTCTCCCTGATCATCTTCACAGAGTTGTCAATCTACAAAGGATCCAAATAG
26 ➨	CCCCAAGTTGAGAGCTCTCCCTGATCATCTTCACAGAGTTGTCAATCTACAAAGGATCCAAATAG
27 ➨	CCCCAAGTTGAGAGCTCTCCCTGATCATCTTCACAGAGTTGTCAATCTACAAAGGATCCAAATAG
28 ➨	CCCCAAGTTGAGAGCTCTCCCTGATCATCTTCACAGAGTTGTCAATCTACAAAGGATCCAAATAG
29 ➨	CCCCAAGTTGAGAGCTCTCCCTGATCATCTTCACAGAGTTGTCAATCTACAAAGGATCCAAATAG
30 ➨	CCCCAAGTTGAGAGCTCTCCCTGATCATCTTCACAGAGTTGTCAATCTACAAAGGATCCAAATAG
31 ➨	CCCCAAGTTGAGAGCTCTCCCTGATCATCTTCACAGAGTTGTCAATCTACAAAGGATCCAAATAG
32 ➨	CCCCAAGTTGAGAGCTCTCCCTGATCATCTTCACAGAGTTGTCAATCTACAAAGGATCCAAATAG
33 ➨	CCCCAAGTTGAGAGCTCTCCCTGATCATCTTCACAGAGTTGTCAATCTACAAAGGATCCAAATAG
34 ➨	CCCCAAGTTGAGAGCTCTCCCTGATCATCTTCACAGAGTTGTCAATCTACAAAGGATCCAAATAG
35 ➨	CCCCAAGTTGAGAGCTCTCCCTGATCATCTTCACAGAGTTGTCAATCTACAAAGGATCCAAATAG
36 ➨	CCCCAAGTTGAGAGCTCTCCCTGATCATCTTCACAGAGTTGTCAATCTACAAAGGATCCAAATAG
37 ➨	CCCCAAGTTGAGAGCTCTCCCTGATCATCTTCACAGAGTTGTCAATCTACAAAGGATCCAAATAG
38 ➨	CCCCAAGTTGAGAGCTCTCCCTGATCATCTTCACAGAGTTGTCAATCTACAAAGGATCCAAATAG
39 ➨	CCCCAAGTTGAGAGCTCTCCCTGATCATCTTCACAGAGTTGTCAATCTACAAAGGATCCAAATAG
40 ➨	CCCCAAGTTGAGAGCTCTCCCTGATCATCTTCACAGAGTTGTCAATCTACAAAGGATCCAAATAG
41 ➨	CCCCAAGTTGAGAGCTCTCCCTGATCATCTTCACAGAGTTGTCAATCTACAAAGGATCCAAATAG
42 ➨	CCCCAAGTTGAGAGCTCTCCCTGATCATCTTCACAGAGTTGTCAATCTACAAAGGATCCAAATAG

2670 2680 2690 2700 2710 2720 2730

	AAGGAGCTGACAGCCTGCAGGAGATTGTCAACCATCCCGGGGTTGTGTGGCTCAAGGTTAAGAAC
1 ➡	AAGGAGCTGACAGCCTGCAGGAGGTTGTCAACCATCCCGGGGTTGTGTGGCTCAAGGTTAAGAAC
2 ➡	AAGGAGCTGACAGCCTGCAGGAGGTTGTCAACCATCCCGGGGTTGTGTGGCTCAAGGTTAAGAAC
3 ➡	AAGGAGCTGACAGCCTGCAGGAGGTTGTCAACCATCCCGGGGTTGTGTGGCTCAAGGTTAAGAAC
4 ➡	AAGGAGCTGACAGCCTGCAGGAGGTTGTCGATCATCCCGGGGTTGTGTGGCTCAAGGTTAAGAAC
5 ➡	AAGGAGCTGACAGCCTGCAGGAGGTTGTCGATCATCCCGGGGTTGTGTGGCTCAAGGTTAAGAAC
6 ➡	AAGGAGCTGACAGCCTGCAGGAGGTTGTCAACCATCCCGGGGTTGTGTGGCTCAAGGCTAAGAAC
7 ➡	AAGGAGCTGACAGCCTGCAGGAGATTGTCAACCATCCCGGGGTTGTGTGGCTCAAGGTTAAGAAC
8 ➡	AAGGAGCTGACAGCCTGCAGGAGATTGTCAACCATCCCAGGGTTGTGTGGCTCAAGGTTAAGAAC
9 ➡	AAGGAGCTGACAGCCTGCAGGAGATTGTCAACCATCCCGGGGTTGTGTGGCTCAAGGTTAAGAAC
10 ➡	AAGGAGCTGACAGCCTGCAGGAGATTGTCAACCATCCCGGGGTTGTGTGGCTCAAGGTTAAGAAC
11 ➡	AAGGAGCTGACAGCCTGCAGGAGATTGTCAACCATCCCGGGGTTGTGTGGCTCAAGGTTAAGAAC
12 ➡	AAGGAGCTGACAGCCTGCAGGAGATTGTCAACCATCCCGGGGTTGTGTGGCTCAAGGTTAAGAAC
13 ➡	AAGGAGCTGACAGCCTGCAGGAGATTGTCAACCATCCCGGGGTTGTGTGGCTCAAGGTTAAGAAC
14 ➡	AAGGAGCTGACAGCCTGCAGGAGATTGTCAACCATCCCGGGGTTGTGTGGCTCAAGGTTAAGAAC
15 ➡	AAGGAGCTGACAGCCTGCAGGAGATTGTCAACCATCCCGGGGTTGTGTGGCTCAAGGTTAAGAAC
16 ➡	AAGGAGCTGACAGCCTGCAGGAGATTGTCAACCATCCCGGGGTTGTGTGGCTCAAGGTTAAGAAC
17 ➡	AAGGAGCTGACAGCCTGCAGGAGATTGTCAACCATCCCGGGGTTGTGTGGCTCAAGGTTAAGAAC
18 ➡	AAGGAGCTGACAGCCTGCAGGAGATTGTCAACCATCCCGGGGTTGTGTGGCTCAAGGTTAAGAAC
19 ➡	AAGGAGCTGACAGCCTGCAGGAGATTGTCAACCATCCCGGGGTTGTGTGGCTCAAGGTTAAGAAC
20 ➡	AAGGAGCTGACAGCCTGCAGGAGATTGTCAACCATCCCGGGGTTGTGTGGCTCAAGGTTAAGAAC
21 ➡	AAGGAGCTGACAGCCTGCAGGAGATTGTCAACCATCCCGGGGTTGTGTGGCTCAAGGTTAAGAAC
22 ➡	AAGGAGCTGACAGCCTGCAGGAGATTGTCAACCATCCCGGGGTTGTGTGGCTCAAGGTTAAGAAC
23 ➡	AAGGAGCTGACAGCCTGCAGGAGATTGTCAACCATCCCGGGGTTGTGTGGCTCAAGGTTAAGAAC
24 ➡	AAGGAGCTGACAGCCTGCAGGAGATTGTCAACCATCCCGGGGTTGTGTGGCTCAAGGTTAAGAAC
25 ➡	AAGGAGCTGACAGCCTGCAGGAGATTGTCAACCATCCCGGGGTTGTGTGGCTCAAGGTTAAGAAC
26 ➡	AAGGAGCTGACAGCCTGCAGGAGATTGTCAACCATCCCGGGGTTGTGTGGCTCAAGGTTAAGAAC
27 ➡	AAGGAGCTGACAGCCTGCAGGAGATTGTCAACCATCCCGGGGTTGTGTGGCTCAAGGTTAAGAAC
28 ➡	AAGGAGCTGACAGCCTGCAGGAGATTGTCAACCATCCCGGGGTTGTGTGGCTCAAGGTTAAGAAC
29 ➡	AAGGAGCTGACAGCCTGCAGGAGATTGTCAACCATCCCGGGGTTGTGTGGCTCAAGGTTAAGAAC
30 ➡	AAGGAGCTGACAGCCTGCAGGAGATTGTCAACCATCCCGGGGTTGTGTGGCTCAAGGTTAAGAAC
31 ➡	AAGGAGCTGACAGCCTGCAGGAGATTGTCAACCATCCCGGGGTTGTGTGGCTCAAGGTTAAGAAC
32 ➡	AAGGAGCTGACAGCCTGCAGGAGATTGTCAACCATCCCGGGGTTGTGTGGCTCAAGGTTAAGAAC
33 ➡	AAGGAGCTGACAGCCTGCAGGAGATTGTCAACCATCCCGGGGTTGTGTGGCTCAAGGTTAAGAAC
34 ➡	AAGGAGCTGACAGCCTGCAGGAGATTGTCAACCATCCCGGGGTTGTGTGGCTCAAGGTTAAGAAC
35 ➡	AAGGAGCTGACAGCCTGCAGGAGATTGTCAACCATCCCGGGGTTGTGTGGCTCAAGGTTAAGAAC
36 ➡	AAGGAGCTGACAGCCTGCAGGAGATTGTCAACCATCCCGGGGTTGTGTGGCTCAAGGTTAAGAAC
37 ➡	AAGGAGCTGACAGCCTGCAGGAGATTGTCAACCATCCCGGGGTTGTGTGGCTCAAGGTTAAGAAC
38 ➡	AAGGAGCTGACAGCCTGCAGGAGATTGTCAACCATCCCGGGGTTGTGTGGCTCAAGGTTAAGAAC
39 ➡	AAGGAGCTGACAGCCTGCAGGAGATTGTCAACCATCCCGGGGTTGTGTGGCTCAAGGTTAAGAAC
40 ➡	AAGGAGCTGACAGCCTGCAGGAGATTGTCAACCATCCCGGGGTTGTGTGGCTCAAGGTTAAGAAC
41 ➡	AAGGAGCTGACAGCCTGCAGGAGATTGTCAACCATCCCGGGGTTGTGTGGCTCAAGGTTAAGAAC
42 ➡	AAGGAGCTGACAGCCTGCAGGAGATTGTCAACCATCCCGGGGTTGTGTGGCTCAAGGTTAAGAAC

2740 2750 2760 2770 2780 2790

	AACAAGTCCTTGAGGAATATATCCAACCTTCCTAAGCTGCGCCTCTTGCTTGCACAAGATTGCCA
1 ➨	AACAAGTCCTTGAGGAATATATCCAACCTTCCTAAGCTGCGCCTCTTGCTTGCACAAGATTGCCA
2 ➨	AACAAGTCCTTGAGGAATATATCCAACCTTCCTAAGCTGCGCCTCTTGCTTGCACAAGATTGCCA
3 ➨	AACAAGTCCTTGAGGAATATATCCAACCTTCCTAAGCTGCGCCTCTTGCTTGCACAAGATTGCCA
4 ➨	AACAAGTCCTTGAGGAATATATCCAACCTTCCTAAGCTGTGCCTCTTGCTTGCACAAGATTGCCA
5 ➨	AACAAGTCCTTGAGGAATATATCCAACCTTCCTAAGCTGTGCCTCTTGCTTGCACAAGATTGCCA
6 ➨	AACAAGTCCTTGAGGAATATATCCAACCTTCCTAAGCTGCGCCTCTTGCTTGCACAAGATTGCCA
7 ➨	AACAAGTCCTTGAGGAATATATCCAACCTTCCTAAGCTGCGCCTCTTGCTTGCACAAGATTGCCA
8 ➨	AACAAGTCCTTGAGGAATATATCCAACCTTCCTAAGCTGCGCCTCTTGCTTGCACAAGATTGCCA
9 ➨	AACAAGTCCTTGAGGAATATATCCAACCTTCCTAAGCTGTGCCTCTTGCTTGCACAAGATTGCCA
10 ➨	AACAAGTCCTTGAGGAATATATCCAACCTTCCTAAGCTGTGCCTCTTGCTTGCACAAGATTGCCA
11 ➨	AACAAGTCCTTGAGGAATATATCCAACCTTCCTAAGCTGCGCCTCTTGCTTGCACAAGATTGCCA
12 ➨	AACAAGTCCTTGAGGAATATATCCAACCTTCCTAAGCTGCGCCTCTTGCTTGCACAAGATTGCCA
13 ➨	AACAAGTCCTTGAGGAATATATCCAACCTTCCTAAGCTGCGCCTCTTGCTTGCACAAGATTGCCA
14 ➨	AACAAGTCCTTGAGGAATATATCCAACCTTCCTAAGCTGCGCCTCTTGCTTGCACAAGATTGCCA
15 ➨	AACAAGTCCTTGAGGAATATATCCAACCTTCCTAAGCTGCGCCTCTTGCTTGCACAAGATTGCCA
16 ➨	AACAAGTCCTTGAGGAATATATCCAACCTTCCTAAGCTGCGCCTCTTGCTTGCACAAGATTGCCA
17 ➨	AACAAGTCCTTGAGGAATATATCCAACCTTCCTAAGCTGCGCCTCTTGCTTGCACAAGATTGCCA
18 ➨	AACAAGTCCTTGAGGAATATATCCAACCTTCCTAAGCTGCGCCTCTTGCTTGCACAAGATTGCCA
19 ➨	AACAAGTCCTTGAGGAATATATCCAACCTTCCTAAGCTGCGCCTCTTGCTTGCACAAGATTGCCA
20 ➨	AACAAGTCCTTGAGGAATATATCCAACCTTCCTAAGCTGCGCCTCTTGCTTGCACAAGATTGCCA
21 ➨	AACAAGTCCTTGAGGAATATATCCAACCTTCCTAAGCTGCGCCTCTTGCTTGCACAAGATTGCCA
22 ➨	AACAAGTCCTTGAGGAATATATCCAACCTTCCTAAGCTGCGCCTCTTGCTTGCACAAGATTGCCA
23 ➨	AACAAGTCCTTGAGGAATATATCCAACCTTCCTAAGCTGCGCCTCTTGCTTGCACAAGATTGCCA
24 ➨	AACAAGTCCTTGAGGAATATATCCAACCTTCCTAAGCTGCGCCTCTTGCTTGCACAAGATTGCCA
25 ➨	AACAAGTCCTTGAGGAATATATCCAACCTTCCTAAGCTGCGCCTCTTGCTTGCACAAGATTGCCA
26 ➨	AACAAGTCCTTGAGGAATATATCCAACCTTCCTAAGCTGCGCCTCTTGCTTGCACAAGATTGCCA
27 ➨	AACAAGTCCTTGAGGAATATATCCAACCTTCCTAAGCTGCGCCTCTTGCTTGCACAAGATTGCCA
28 ➨	AACAAGTCCTTGAGGAATATATCCAACCTTCCTAAGCTGCGCCTCTTGCTTGCACAAGATTGCCA
29 ➨	AACAAGTCCTTGAGGAATATATCCAACCTTCCTAAGCTGCGCCTCTTGCTTGCACAAGATTGCCA
30 ➨	AACAAGTCCTTGAGGAATATATCCAACCTTCCTAAGCTGCGCCTCTTGCTTGCACAAGATTGCCA
31 ➨	AACAAGTCCTTGAGGAATATATCCAACCTTCCTAAGCTGCGCCTCTTGCTTGCACAAGATTGCCA
32 ➨	AACAAGTCCTTGAGGAATATATCCAACCTTCCTAAGCTGCGCCTCTTGCTTGCACAAGATTGCCA
33 ➨	AACAAGTCCTTGAGGAATATATCCAACCTTCCTAAGCTGCGCCTCTTGCTTGCACAAGATTGCCA
34 ➨	AACAAGTCCTTGAGGAATATATCCAACCTTCCTAAGCTGCGCCTCTTGCTTGCACAAGATTGCCA
35 ➨	AACAAGTCCTTGAGGAATATATCCAACCTTCCTAAGCTGCGCCTCTTGCTTGCACAAGATTGCCA
36 ➨	AACAAGTCCTTGAGGAATATATCCAACCTTCCTAAGCTGCGCCTCTTGCTTGCACAAGATTGCCA
37 ➨	AACAAGTCCTTGAGGAATATATCCAACCTTCCTAAGCTGCGCCTCTTGCTTGCACAAGATTGCCA
38 ➨	AACAAGTCCTTGAGGAATATATCCAACCTTCCTAAGCTGCGCCTCTTGCTTGCACAAGATTGCCA
39 ➨	AACAAGTCCTTGAGGAATATATCCAACCTTCCTAAGCTGCGCCTCTTGCTTGCACAAGATTGCCA
40 ➨	AACAAGTCCTTGAGGAATATATCCAACCTTCCTAAGCTGCGCCTCTTGCTTGCACAAGATTGCCA
41 ➨	AACAAGTCCTTGAGGAATATATCCAACCTTCCTAAGCTGCGCCTCTTGCTTGCACAAGATTGCCA
42 ➨	AACAAGTCCTTGAGGAATATATCCAACCTTCCTAAGCTGCGCCTCTTGCTTGCACAAGATTGCCA

	2800 2810 2820 2830 2840 2850 2860
	AGAATTGCAGCAGGCAGAGAACCTTAGCTCACTCAAGGCCTTGTACGTTGTCGATTGCCCTATGG
1 ➡	AGAATTGCAGCAGGCAGAGAACCTTAGCTCACTCAAGGCCTTGTACGTTGTCGATTGCCCTATGG
2 ➡	AGAATTGCAGCAGGCAGAGAACCTTAGCTCACTCAAGGCCTTGTACGTTGTCGATTGCCCTATGG
3 ➡	AGAATTGCAGCAGGCAGAGAACCTTAGCTCACTCAAGGCCTTGTACGTTGTCGATTGCCCTATGG
4 ➡	CGAATTGCAGCAGGCAGAGAACCTTAGCTCACTCAAGGCCTTGTACGTTGTCGATTGCCCTATGG
5 ➡	CGAATTGCAGCAGGCAGAGAACCTTAGCTCACTCAAGGCCTTGTACGTTGTCGATTGCCCTATGG
6 ➡	AGAATTGCAGCAGGCAGAGAACCTTAGCTCACTCAAGGCCTTGTACGTTGTCGATTGCCCTATGG
7 ➡	AGAATTGCAGCAGGCAGAGAACCTTAGCTCACTCAAGGCCTTGTACGTTGTCGATTGCCCTATGG
8 ➡	AGAATTGCAGCAGGCAGAGAACCTTAGCTCACTCAAGGCCTTGTACGTTGTCGATTGCCCTATGG
9 ➡	AGAATTGCAGCAGGCAGAGAACCTTAGCTCACTCAAGGCCTTGTACGTTGTCGATTGCCCTATGG
10 ➡	AGAATTGCAGCAGGCAGAGAACCTTAGCTCACTCAAGGCCTTGTACGTTGTCGATTGCCCTATGG
11 ➡	AGAATTGCAGCAGGCAGAGAACCTTAGCTCACTCAAGGCCTTGTACGTTGTCGATTGCCCTATGG
12 ➡	AGAATTGCAGCAGGCAGAGAACCTTAGCTCACTCAAGGCCTTGTACGTTGTCGATTGCCCTATGG
13 ➡	AGAATTGCAGCAGGCAGAGAACCTTAGCTCACTCAAGGCCTTGTACGTTGTCGATTGCCCTATGG
14 ➡	AGAATTGCAGCAGGCAGAGAACCTTAGCTCACTCAAGGCCTTGTACGTTGTCGATTGCCCTATGG
15 ➡	AGAATTGCAGCAGGCAGAGAACCTTAGCTCACTCAAGGCCTTGTACGTTGTCGATTGCCCTATGG
16 ➡	AGAATTGCAGCAGGCAGAGAACCTTAGCTCACTCAAGGCCTTGTACGTTGTCGATTGCCCTATGG
17 ➡	AGAATTGCAGCAGGCAGAGAACCTTAGCTCACTCAAGGCCTTGTACGTTGTCGATTGCCCTATGG
18 ➡	AGAATTGCAGCAGGCAGAGAACCTTAGCTCACTCAAGGCCTTGTACGTTGTCGATTGCCCTATGG
19 ➡	AGAATTGCAGCAGGCAGAGAACCTTAGCTCACTCAAGGCCTTGTACGTTGTCGATTGCCCTATGG
20 ➡	AGAATTGCAGCAGGCAGAGAACCTTAGCTCACTCAAGGCCTTGTACGTTGTCGATTGCCCTATGG
21 ➡	AGAATTGCAGCAGGCAGAGAACCTTAGCTCACTCAAGGCCTTGTACGTTGTCGATTGCCCTATGG
22 ➡	AGAATTGCAGCAGGCAGAGAACCTTAGCTCACTCAAGGCCTTGTACGTTGTCGATTGCCCTATGG
23 ➡	AGAATTGCAGCAGGCAGAGAACCTTAGCTCACTCAAGGCCTTGTACGTTGTCGATTGCCCTATGG
24 ➡	AGAATTGCAGCAGGCAGAGAACCTTAGCTCACTCAAGGCCTTGTACGTTGTCGATTGCCCTATGG
25 ➡	AGAATTGCAGCAGGCAGAGAACCTTAGCTCACTCAAGGCCTTGTACGTTGTCGATTGCCCTATGG
26 ➡	AGAATTGCAGCAGGCAGAGAACCTTAGCTCACTCAAGGCCTTGTACGTTGTCGATTGCCCTATGG
27 ➡	AGAATTGCAGCAGGCAGAGAACCTTAGCTCACTCAAGGCCTTGTACGTTGTCGATTGCCCTATGG
28 ➡	AGAATTGCAGCAGGCAGAGAACCTTAGCTCACTCAAGGCCTTGTACGTTGTCGATTGCCCTATGG
29 ➡	AGAATTGCAGCAGGCAGAGAACCTTAGCTCACTCAAGGCCTTGTACGTTGTCGATTGCCCTATGG
30 ➡	AGAATTGCAGCAGGCAGAGAACCTTAGCTCACTCAAGGCCTTGTACGTTGTCGATTGCCCTATGG
31 ➡	AGAATTGCAGCAGGCAGAGAACCTTAGCTCACTCAAGGCCTTGTACGTTGTCGATTGCCCTATGG
32 ➡	AGAATTGCAGCAGGCAGAGAACCTTAGCTCACTCAAGGCCTTGTACGTTGTCGATTGCCCTATGG
33 ➡	AGAATTGCAGCAGGCAGAGAACCTTAGCTCACTCAAGGCCTTGTACGTTGTCGATTGCCCTATGG
34 ➡	AGAATTGCAGCAGGCAGAGAACCTTAGCTCACTCAAGGCCTTGTACGTTGTCGATTGCCCTATGG
35 ➡	AGAATTGCAGCAGGCAGAGAACCTTAGCTCACTCAAGGCCTTGTACGTTGTCGATTGCCCTATGG
36 ➡	AGAATTGCAGCAGGCAGAGAACCTTAGCTCACTCAAGGCCTTGTACGTTGTCGATTGCCCTATGG
37 ➡	AGAATTGCAGCAGGCAGAGAACCTTAGCTCACTCAAGGCCTTGTACGTTGTCGATTGCCCTATGG
38 ➡	AGAATTGCAGCAGGCAGAGAACCTTAGCTCACTCAAGGCCTTGTACGTTGTCGATTGCCCTATGG
39 ➡	AGAATTGCAGCAGGCAGAGAACCTTAGCTCACTCAAGGCCTTGTACGTTGTCGATTGCCCTATGG
40 ➡	AGAATTGCAGCAGGCAGAGAACCTTAGCTCACTCAAGGCCTTGTACGTTGTCGATTGCCCTATGG
41 ➡	AGAATTGCAGCAGGCAGAGAACCTTAGCTCACTCAAGGCCTTGTACGTTGTCGATTGCCCTATGG
42 ➡	AGAATTGCAGCAGGCAGAGAACCTTAGCTCACTCAAGGCCTTGTACGTTGTCGATTGCCCTATGG

2870 2880 2890 2900 2910 2920

	AGCAGATACTCTGGAAGTGTTTCCCCATAGAACAACAGAGCACGATTGTCCGTGTTGTCACCACT
1	AGCAGATACTCTGGAAGTGTTTCCCCATAGAACAACAGAGCACGATTGTCCGTGTTGTCACCACT
2	AGCAGATACTCTGGAAGTGTTTCCCCATAGAACAACAGAGCACGATTGTCCGTGTTGTCACCACT
3	AGCAGATACTCTGGAAGTGTTTCCCCATAGAACAACAGAGCACGATTGTCCGTGTTGTCACCACT
4	AGCAGATACTCTGGAAGTGTTTCCCCATAGAACAACAGAGCACAATTGTCCGTGTTGTCACCACT
5	AGCAGATACTCTGGAAGTGTTTCCCCATAGAACAACAGAGCACAATTGTCCGTGTTGTCACCACT
6	AGCAGATACTCTGGAAGTGTTTCCCCATAGAACAACAGAGCACGATTGTCCGTGTTGTCACCACT
7	AGCAGATACTCTGGAAGTGTTTCCCCATAGAACAACAGAGCACAATTGTCCGTGTTGTCACCACT
8	AGCAGATACTCTGGAAGTGTTTCCCCATAGAACAACAGAGCACGATTGTCCGTGTTGTCACCACT
9	AGCAGATACTCTGGAAGTGTTTCCCCATAGAACAACAGAGCACGATTGTCCGTGTTGTCACCACT
10	AGCAGATACTCTGGAAGTGTTTCCCCATAGAACAACAGAGCACGATTGTCCGTGTTGTCACCACT
11	AGCAGATACTCTGGAAGTGTTTCCCCATAGAACAACAGAGCACGATTGTCCGTGTTGTCACCACT
12	AGCAGATACTCTGGAAGTGTTTCCCCATAGAACAACAGAGCACGATTGTCCGTGTTGTCACCACT
13	AGCAGATACTCTGGAAGTGTTTCCCCATAGAACAACAGAGCACGATTGTCCGTGTTGTCACCACT
14	AGCAGATACTCTGGAAGTGTTTCCCCATAGAACAACAGAGCACGATTGTCCGTGTTGTCACCACT
15	AGCAGATACTCTGGAAGTGTTTCCCCATAGAACAACAGAGCACGATTGTCCGTGTTGTCACCACT
16	AGCAGATACTCTGGAAGTGTTTCCCCATAGAACAACAGAGCACGATTGTCCGTGTTGTCACCACT
17	AGCAGATACTCTGGAAGTGTTTCCCCATAGAACAACAGAGCACGATTGTCCGTGTTGTCACCACT
18	AGCAGATACTCTGGAAGTGTTTCCCCATAGAACAACAGAGCACGATTGTCCGTGTTGTCACCACT
19	AGCAGATACTCTGGAAGTGTTTCCCCATAGAACAACAGAGCACGATTGTCCGTGTTGTCACCACT
20	AGCAGATACTCTGGAAGTGTTTCCCCATAGAACAACAGAGCACGATTGTCCGTGTTGTCACCACT
21	AGCAGATACTCTGGAAGTGTTTCCCCATAGAACAACAGAGCACGATTGTCCGTGTTGTCACCACT
22	AGCAGATACTCTGGAAGTGTTTCCCCATAGAACAACAGAGCACGATTGTCCGTGTTGTCACCACT
23	AGCAGATACTCTGGAAGTGTTTCCCCATAGAACAACAGAGCACGATTGTCCGTGTTGTCACCACT
24	AGCAGATACTCTGGAAGTGTTTCCCCATAGAACAACAGAGCACGATTGTCCGTGTTGTCACCACT
25	AGCAGATACTCTGGAAGTGTTTCCCCATAGAACAACAGAGCACGATTGTCCGTGTTGTCACCACT
26	AGCAGATACTCTGGAAGTGTTTCCCCATAGAACAACAGAGCACGATTGTCCGTGTTGTCACCACT
27	AGCAGATACTCTGGAAGTGTTTCCCCATAGAACAACAGAGCACGATTGTCCGTGTTGTCACCACT
28	AGCAGATACTCTGGAAGTGTTTCCCCATAGAACAACAGAGCACGATTGTCCGTGTTGTCACCACT
29	AGCAGATACTCTGGAAGTGTTTCCCCATAGAACAACAGAGCACGATTGTCCGTGTTGTCACCACT
30	AGCAGATACTCTGGAAGTGTTTCCCCATAGAACAACAGAGCACGATTGTCCGTGTTGTCACCACT
31	AGCAGATACTCTGGAAGTGTTTCCCCATAGAACAACAGAGCACGATTGTCCGTGTTGTCACCACT
32	AGCAGATACTCTGGAAGTGTTTCCCCATAGAACAACAGAGCACGATTGTCCGTGTTGTCACCACT
33	AGCAGATACTCTGGAAGTGTTTCCCCATAGAACAACAGAGCACGATTGTCCGTGTTGTCACCACT
34	AGCAGATACTCTGGAAGTGTTTCCCCATAGAACAACAGAGCACGATTGTCCGTGTTGTCACCACT
35	AGCAGATACTCTGGAAGTGTTTCCCCATAGAACAACAGAGCACGATTGTCCGTGTTGTCACCACT
36	AGCAGATACTCTGGAAGTGTTTCCCCATAGAACAACAGAGCACGATTGTCCGTGTTGTCACCACT
37	AGCAGATACTCTGGAAGTGTTTCCCCATAGAACAACAGAGCACGATTGTCCGTGTTGTCACCACT
38	AGCAGATACTCTGGAAGTGTTTCCCCATAGAACAACAGAGCACGATTGTCCGTGTTGTCACCACT
39	AGCAGATACTCTGGAAGTGTTTCCCCATAGAACAACAGAGCACGATTGTCCGTGTTGTCACCACT
40	AGCAGATACTCTGGAAGTGTTTCCCCATAGAACAACAGAGCACGATTGTCCGTGTTGTCACCACT
41	AGCAGATACTCTGGAAGTGTTTCCCCATAGAACAACAGAGCACGATTGTCCGTGTTGTCACCACT
42	AGCAGATACTCTGGAAGTGTTTCCCCATAGAACAACAGAGCACGATTGTCCGTGTTGTCACCACT

	2930 2940 2950 2960 2970
	GGGGCCCATGGTCAGGATATTTATCCTCTTGAATCAGTATTTCATTAA
1	GGGGCCCATGGTCAGGATATTTATCCTCTTGAATCAGTATTTCATTAA
2	GGGGCCCATGGTCAGGATATTTATCCTCTTGAATCAGTATTTCATTAA
3	GGGGCCCATGGTCAGGATATTTATCCTCTTGAATCAGTATTTCATTAA
4	GGG**A**CCCATGGTCAGGATATTTATCCTCTTGAATCAGT**G**TTTCATTAA
5	GGG**A**CCCATGGTCAGGATATTTATCCTCTTGAATCAGT**G**TTTCATTAA
6	GGGGCCCATGGTCAGGATATTTATCCTCTTGAATCAGTATTTCATTAA
7	GGGGCCCATGGTCAGGATATTTATCCTCTTGAATCAGTATTTCATTAA
8	GGGGCCCATGGTCAGGATATTTATCCTCTTGAATCAGTATTTCATTAA
9	GGGGCCCATGGTCAGGATATTTATCCTCTTGAATCAGTATTTCATTAA
10	GGGGCCCATGGTCAGGATATTTATCCTCTTGAATCAGTATTTCATTAA
11	GGGGCCCATGGTCAGGATATTTATCCTCTTGAATCAGTATTTCATTAA
12	GGGGCCCATGGTCAGGATATTTATCCTCTTGAATCAGTATTTCATTAA
13	GGGGCCCATGGTCAGGATATTTATCCTCTTGAATCAGTATTTCATTAA
14	GGGGCCCATGGTCAGGATATTTATCCTCTTGAATCAGTATTTCATTAA
15	GGGGCCCATGGTCAGGATATTTATCCTCTTGAATCAGTATTTCATTAA
16	GGGGCCCATGGTCAGGATATTTATCCTCTTGAATCAGTATTTCATTAA
17	GGGGCCCATGGTCAGGATATTTATCCTCTTGAATCAGTATTTCATTAA
18	GGGGCCCATGGTCAGGATATTTATCCTCTTGAATCAGTATTTCATTAA
19	GGGGCCCATGGTCAGGATATTTATCCTCTTGAATCAGTATTTCATTAA
20	GGGGCCCATGGTCAGGATATTTATCCTCTTGAATCAGTATTTCATTAA
21	GGGGCCCATGGTCAGGATATTTATCCTCTTGAATCAGTATTTCATTAA
22	GGGGCCCATGGTCAGGATATTTATCCTCTTGAATCAGTATTTCATTAA
23	GGGGCCCATGGTCAGGATATTTATCCTCTTGAATCAGTATTTCATTAA
24	GGGGCCCATGGTCAGGATATTTATCCTCTTGAATCAGTATTTCATTAA
25	GGGGCCCATGGTCAGGATATTTATCCTCTTGAATCAGTATTTCATTAA
26	GGGGCCCATGGTCAGGATATTTATCCTCTTGAATCAGTATTTCATTAA
27	GGGGCCCATGGTCAGGATATTTATCCTCTTGAATCAGTATTTCATTAA
28	GGGGCCCATGGTCAGGATATTTATCCTCTTGAATCAGTATTTCATTAA
29	GGGGCCCATGGTCAGGATATTTATCCTCTTGAATCAGTATTTCATTAA
30	GGGGCCCATGGTCAGGATATTTATCCTCTTGAATCAGTATTTCATTAA
31	GGGGCCCATGGTCAGGATATTTATCCTCTTGAATCAGTATTTCATTAA
32	GGGGCCCATGGTCAGGATATTTATCCTCTTGAATCAGTATTTCATTAA
33	GGGGCCCATGGTCAGGATATTTATCCTCTTGAATCAGTATTTCATTAA
34	GGGGCCCATGGTCAGGATATTTATCCTCTTGAATCAGTATTTCATTAA
35	GGGGCCCATGGTCAGGATATTTATCCTCTTGAATCAGTATTTCATTAA
36	GGGGCCCATGGTCAGGATATTTATCCTCTTGAATCAGTATTTCATTAA
37	GGGGCCCATGGTCAGGATATTTATCCTCTTGAATCAGTATTTCATTAA
38	GGGGCCCATGGTCAGGATATTTATCCTCTTGAATCAGTATTTCATTAA
39	GGGGCCCATGGTCAGGATATTTATCCTCTTGAATCAGTATTTCATTAA
40	GGGGCCCATGGTCAGGATATTTATCCTCTTGAATCAGTATTTCATTAA
41	GGGGCCCATGGTCAGGATATTTATCCTCTTGAATCAGTATTTCATTAA
42	GGGGCCCATGGTCAGGATATTTATCCTCTTGAATCAGTATTTCATTAA

Supplemental Figure S16. Alignment of *ARG1* sequences from different genotypes.
Sequences were obtained from our sequencing results and database, generated by SnapGene (The sequence alignment algorithm used is ClustalOmega, https://www.ebi.ac.uk/Tools/msa/clustalw2/). In first page, thick red lines indicate the *ARG1* genes. The small boxes on the lines show single nucleotide polymorphisms (SNPs) alignment of *ARG1* gene. The red boxes indicate the SNPs in page 2 to 50.
